# A cancer vaccine approach for personalized treatment of Lynch Syndrome

**DOI:** 10.1038/s41598-018-30466-x

**Published:** 2018-08-14

**Authors:** Snigdha Majumder, Rakshit Shah, Jisha Elias, Malini Manoharan, Priyanka Shah, Anjali Kumari, Papia Chakraborty, Vasumathi Kode, Yogesh Mistry, Karunakaran Coral, Bharti Mittal, Sakthivel Murugan SM, Lakshmi Mahadevan, Ravi Gupta, Amitabha Chaudhuri, Arati Khanna-Gupta

**Affiliations:** 1 MedGenome Labs Ltd., Bangalore, India; 2 KCHRC, Muni Seva Ashram, Goraj, Gujarat India; 3 MedGenome Inc, Foster City, CA USA

## Abstract

Lynch syndrome (LS) is a cancer predisposition disorder wherein patients have a 70–80% lifetime risk of developing colorectal cancers (CRC). Finding germline mutations in predisposing genes allows for risk assessment of CRC development. Here we report a germline heterozygous frame-shift mutation in the mismatch repair *MLH1* gene which was identified in members of two unrelated LS families. Since defects in DNA mismatch repair genes generate frame-shift mutations giving rise to highly immunogenic neoepitopes, we postulated that vaccination with these mutant peptide antigens could offer promising treatment options to LS patients. To this end we performed whole-exome and RNA seq analysis on the blood and tumour samples from an LS-CRC patient, and used our proprietary neoepitope prioritization pipeline OncoPept*VAC* to select peptides, and confirm their immunogenicity in an *ex vivo* CD8^+^ T cell activation assay. Three neoepitopes derived from the tumour of this patient elicited a potent CD8^+^ T cell response. Furthermore, analysis of the tumour-associated immune infiltrate revealed CD8^+^ T cells expressing low levels of activation markers, suggesting mechanisms of immune suppression at play in this relapsed tumour. Taken together, our study paves the way towards development of a cancer vaccine to treat or delay the onset/relapse of LS-CRC.

## Introduction

Lynch Syndrome (LS) is an inherited heterozygous autosomal dominant disorder which predisposes affected individuals to the risk of developing colorectal cancer (CRC) as well as to endometrial carcinomas, tumours of the stomach, small intestines, ureter, brain, pelvis and prostate among others^1^. It is the most common hereditary CRC syndrome accounting for 2–5% of all CRCs. In the developed world, the estimated disease frequency ranges from 1:370 to 1:2000^2^ but no prevalence details have been officially reported from developing nations to date. In India, while the overall incidence of CRC is comparatively lower than in the west, a large percentage of patients develop CRC before the age of 45 with a higher proportion (10–15%) of LS-CRC cases^3^.

Microsatellite instability (MSI) and deleterious germline mutations in mismatch repair genes (MMR) are the root causes underlying Lynch Syndrome. While LS patients harbour germline mutations in one or more of the MMR genes, patients with sporadic cancers carry somatic mutations in these genes. In either case, the initial heterozygous mutation in an MMR gene appears to be tolerated. A second hit in the same MMR gene or another MMR pathway gene resulting in loss of heterozygosity (LOH), leads to defects in the DNA repair machinery, which in turn result in extensive genome instability. Over 95% of tumours associated with MMR gene mutations in LS demonstrate extensive instability in both coding and non-coding short repeats known as microsatellite sequences, leading to microsatellite instability (MSI). CRC associated with LS therefore presents at a much younger age in comparison to sporadic CRCs, due to the rapid accumulation of mutations in oncogenes and tumour suppressor genes, as a result of MSI. This hyper mutated state leads to an aggressive and rapidly evolving form of CRC (reviewed in ref.^4^).

**Figure 1:**
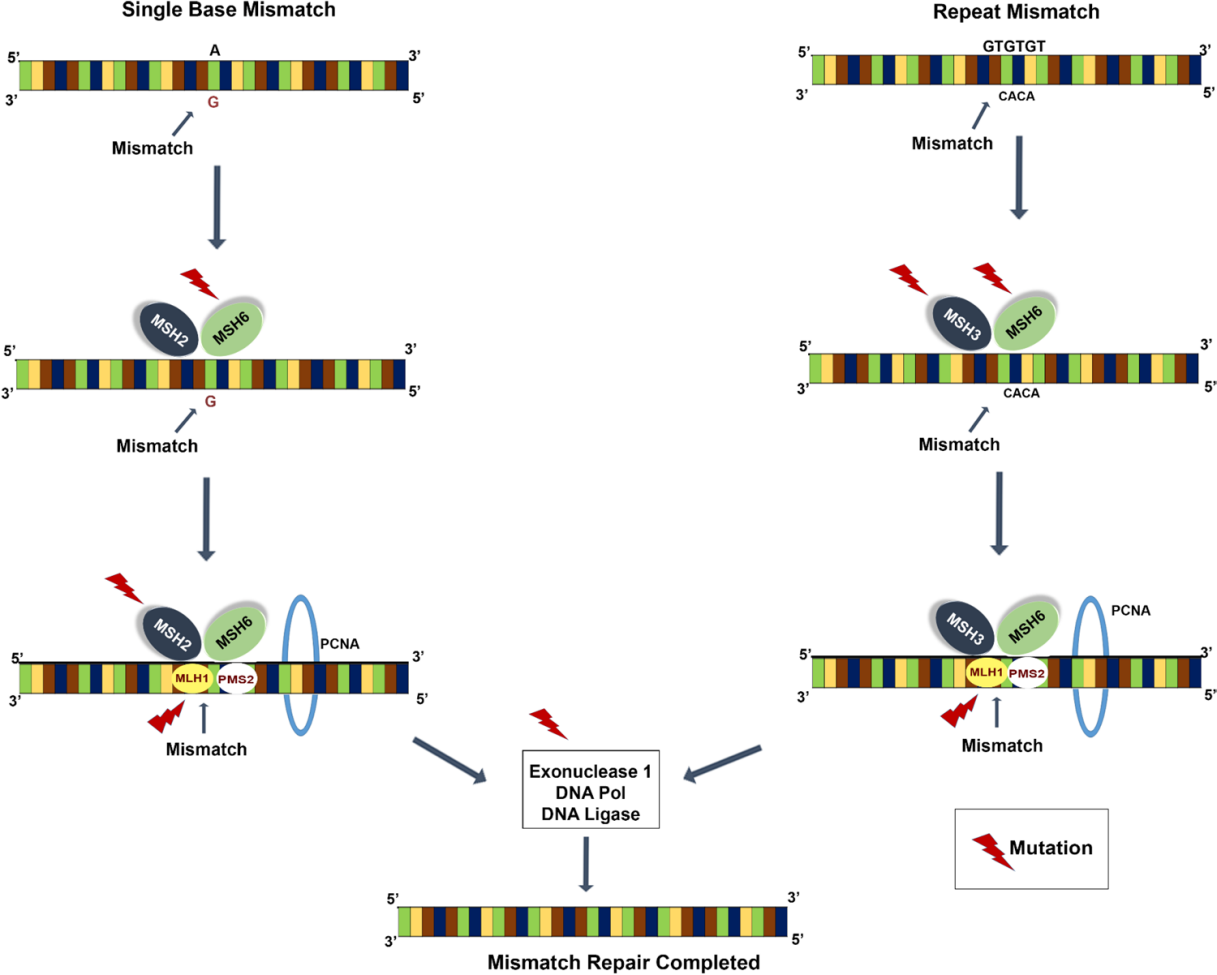
The mismatch repair pathway. Red arrow indicates the proteins of the repair pathway where somatic or germline mutations were found in patient Family 2: II.2; LS^+^ MLH1^mut^.

The fidelity of DNA replication in combination with mechanisms to correct replication errors are evolutionary conserved, vital processes that prevent the development of cancer due to the accumulation of random mutations, particularly in oncogenes and tumour suppressor genes^5,6^. In eukaryotic cells, the replicative DNA polymerases epsilon and delta (ε and δ) make 100,000 replicative errors per cell division, and with their inherent proofreading function, correct the errors to maintain tissue homeostasis. The MMR system functions along with the DNA polymerases to remove mismatched nucleotides to decrease the error rate further by up to one in 10 billion bases per replicative cycle^6,7^. Essentially, a DNA mismatch occurring during replication, if not proofread by the polymerases ε and δ, is recognised by the MSH2/MSH6 heterodimer (for mismatches of 1–2 bases) or by the MSH2/MSH3 heterodimers (for larger insertion/deletion loops). Such mismatches are commonly encountered in the microsatellite domains of the genome^8^. Subsequently, a second heterodimer of MLH1/PMS2 recognizes and binds to the first heterodimer forming a ternary complex at the mismatched site. This ternary complex together with exonuclease 1, proliferating cell nuclear antigen (PCNA) and DNA polymerase δ, remove the mismatched bases and repair the error, thereby contributing to the maintenance of strict DNA replication fidelity (Fig. 1)^8^. Approximately 15% of all CRCs can be attributed to MMR deficiency, with 2–3% contributed by germline mutations in the *MLH1*, *MSH2*, *MSH6*, *PMS2* or *EPCAM* genes. An additional 12% of CRC cases occur due to somatic inactivation of the *MLH1* gene resulting from promoter hypermethylation^9^.

Lynch syndrome associated tumours carry a high burden of insertion/deletion mutations due to the persistence of DNA replication errors in microsatellite rich areas of the genome, leading to the accumulation of a large number of frameshift mutations in the tumours of affected individuals. Additionally, an abundance of tumour infiltrating T-Lymphocytes (TILs) and the so-called Crohn’s-like reaction with prominent aggregates at the periphery of the tumour, suggesting a local inflammatory response, have been reported^10^. Since cancer development is closely associated with the breakdown of immune surveillance mechanisms, the presence of tumour infiltrated leukocytes may represent an unsuccessful attempt to mount an anti-tumour immune response. A recent study showed that the immune microenvironment of MSI-high (MSI-H) colorectal cancers was not only highly infiltrated with activated CD8^+^ cytotoxic T cells, but also showed upregulation of multiple immune checkpoints, including PD-1 (Programmed Death-1), PDL-1 (Programmed Death ligand-1), CTLA-4 (Cytotxic T cell antigen -4), LAG-3 (lymphocyte-activation gene 3), TIM3 (T-cell immunoglobulin and mucin-domain containing-3) and IDO (Indoleamine 2,3-dioxygenase), thereby protecting the tumours from immune-mediated elimination^11^. The observed increase in the response rate of some MSI-H tumours to anti-PD-1 therapy is in concordance with this observation^5,6^. Interestingly, LS patients with MSI-H CRCs have a better prognosis when the tumours have a higher density of tumour infiltrating CTLs compared with MSS (microsatellite stable) CRC, which tend to be CTL depleted^12^.

The recognition of the tumour as non-self is mediated, in part, by tumour-derived neoepitopes, which are immunogenic peptides arising from intracellular proteolytic processing of somatic mutations. Peptides derived from mutated protein coding genes bind HLA Class I (MHC) proteins, which in turn bind T-cell receptors (TCR) on naïve CD8^+^ T-cells, thereby transforming them into cytotoxic T cells, capable of mediating tumour lysis^13,14^. Since almost all tumours in LS patients are hyper-mutated, they are likely to express large numbers of neoantigens, which in turn could elicit an antigen-specific cytotoxic T-cell response^15–17^.

In this study, we analyzed genetic alterations associated with progression of Lynch syndrome to CRC. Our study provides evidence that a valuable source of therapeutic cancer vaccine peptides for hereditary cancers can be harnessed from the multitude of somatic mutations associated with the switch to cancer progression.

## Results

### Identification of a common germline mutation in the *MLH1* gene in two unrelated Lynch syndrome-affected families

**Figure 2:**
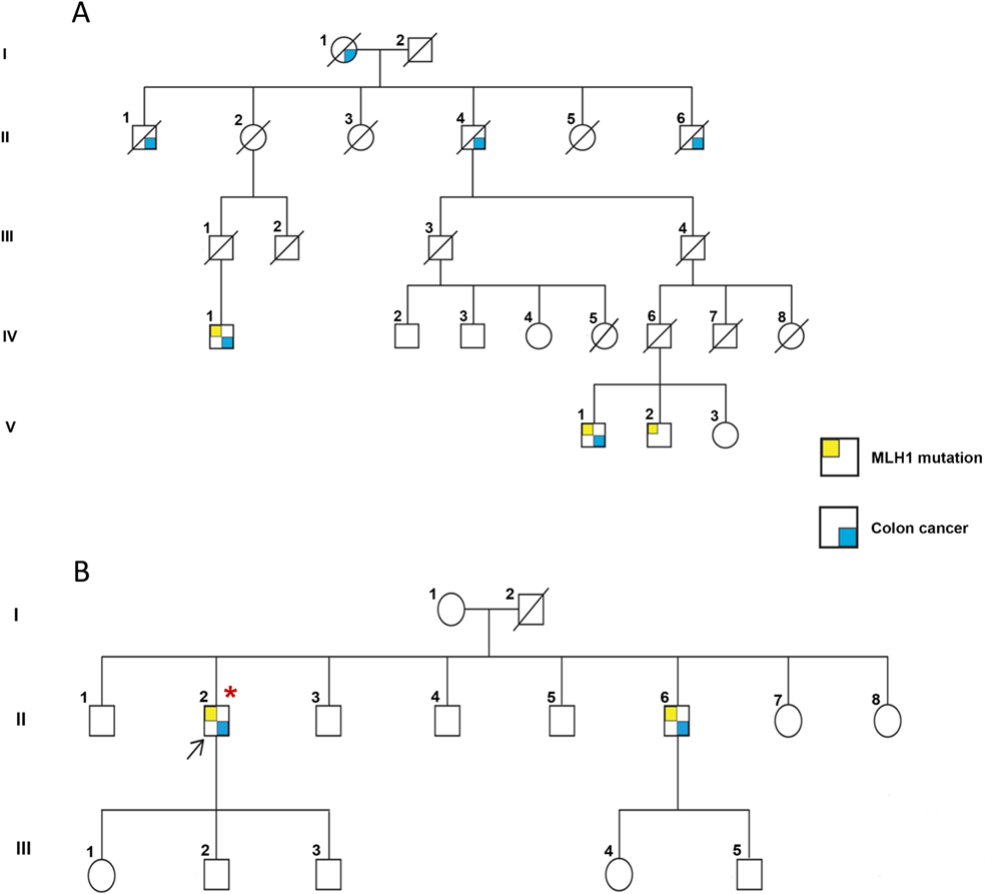
Pedigrees of Lynch syndrome affected families: **(A)** A heterozygous germline *MLH1* mutation was found to be present in 3 members (IV.1, V.1, V.2). 2 members carrying the germline mutation were diagnosed with colorectal cancer and had undergone surgery (IV.1 and V.1). Four other deceased family members were known to have had colorectal cancer. The remaining family members were normal. Note: Samples from individuals IV.4 and V.3 could not be collected. (**B**) The proband of unrelated family 2 (II.2), indicated by a black arrow, was diagnosed with a relapsed colorectal cancer at KCHRC and had undergone surgery. The same heterozygous germline *MLH1* mutation was found in the proband and one other member of the family (II.6) who was also diagnosed with colorectal cancer and had undergone surgery. *All somatic mutation data described in this study were obtained from this patient. All other family members lacked the *MLH1* germline mutation. Color Key:  *MLH1* gene mutation  diagnosed with colorectal cancer. A slash through the shape indicates a deceased member. Roman numerals indicate generations.

**Figure 3:**
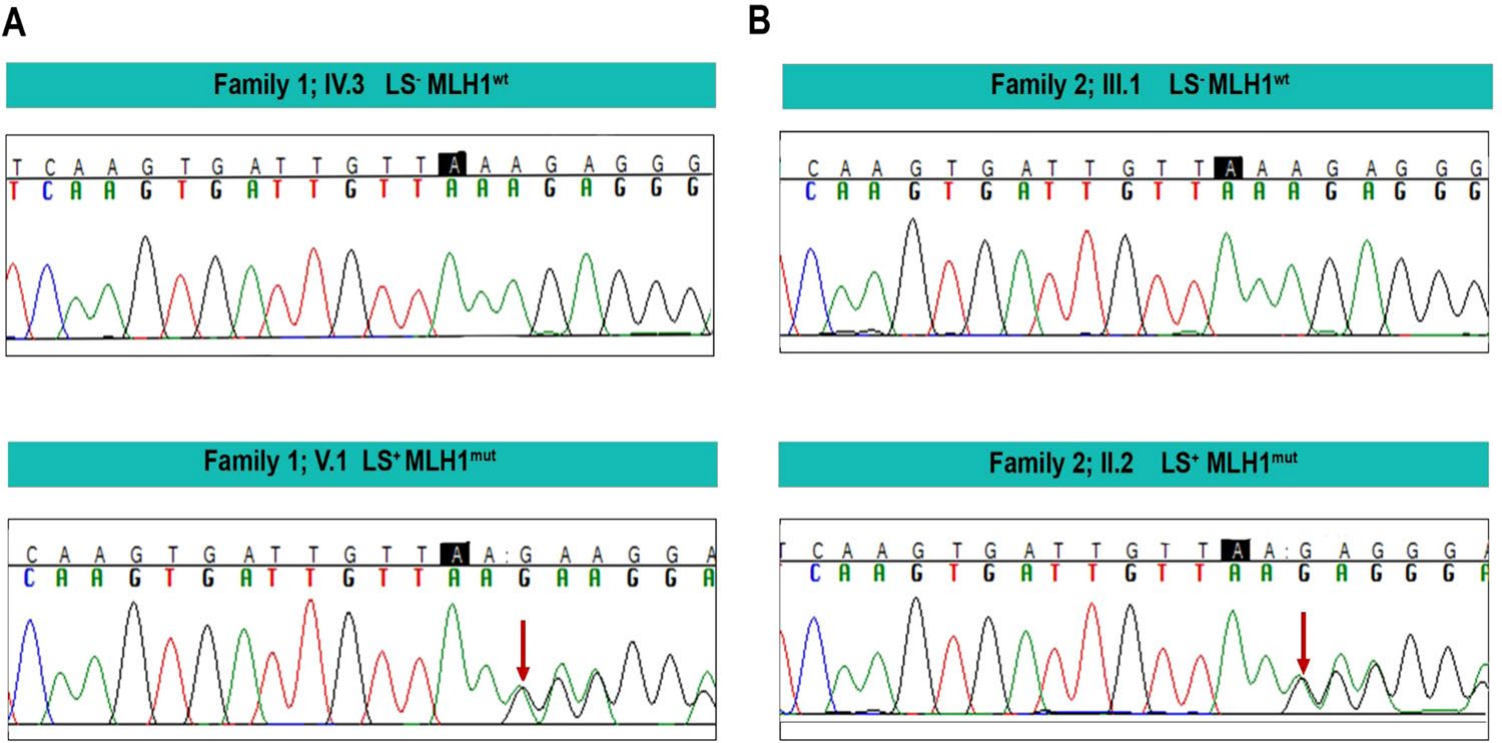
Sanger sequencing confirmation of the germline mutation in the *MLH1* gene: Sanger sequencing analysis was performed on two affected (Family 1: V.1, LS^+^ MLH1^mut^ and Family 2: II.2, LS^+^ MLH1^mut^) and two unaffected (Family 1: IV.3, LS^−^ MLH1^wt^ and Family 2: III.1, LS^−^ MLH1^wt^) members of the two LS families. The presence of *MLH1* mutation (chr3:37038147; c.154delA; p.Glu53ArgfsTer) was confirmed as shown in the example below, in both affected members as indicated by a red arrow, bottom panel.

The unaffected patriarch (Fig. 2A, IV.3) of Family 1 approached us at the Kailash Cancer Hospital and Research Centre (KCHRC) Goraj, in rural India, with the observation that several members of his family were affected with colorectal cancer, many of who were since deceased. Based on his description, a pedigree chart was prepared (Fig. 2A, Suppl. Tables 1 and 2). NGS analysis, followed by confirmation Sanger sequencing of all living family members (seven individuals) identified a heterozygous frameshift mutation leading to premature termination (c.154delA; p.Glu53ArgfsTer) in the *MLH*1 gene in three individuals (Fig. 2A: IV.1, V.1 and V.2; Suppl. Table 1 and Fig. 3, left-hand panel). While one of the three individuals carrying the mutation was unaffected (V.2, aged 51, no progression to CRC), the other two individuals (IV.1 aged 46 and V.1 aged 57) were diagnosed with adenocarcinoma of the colon (mucinous, with extracellular mucin >50%) and were operated to remove the tumour. The *MLH1* mutation was not detected in the remaining four family members (IV.2, IV.3, IV.4 and V.3) (Fig. 2A, Suppl. Table 1, Fig. 3A).

In the second LS family (Fig. 2B), the proband (Fig. 2B, II.2, arrow and asterix) was diagnosed with colon adenocarcinoma and had undergone a right hemi-colonectomy. He returned after ten years with relapsed colon adenocarcinoma that required surgery. The proband’s brother was also diagnosed with rectosigmoidal adenocarcinoma. Following surgery to remove the tumour, he underwent 4 cycles of capecitabine and oxaloplatin therapy and remains cancer-free to date. DNA isolated from the blood of all thirteen members of this family was analysed by Sanger sequencing methods (Fig. 3, right-hand panel; Suppl. Table 2) and the same heterozygous mutation in the *MLH*1 gene identified in Family 1 was also detected in the affected proband (II.2) and his brother (II.6), but not in any of the other unaffected family members (Fig. 2B, Suppl. Table 2, Fig. 3B).

In order to confirm that the *MLH1* mutation was in fact the disease causing mutation, we further analysed the germline NGS data of two affected and two unaffected Lynch Syndrome family members (Family 2) and looked for common mutations in additional MMR genes: *PMS1*, *PMS2*, *MSH2*, *MLH3*, *MSH6*, *PCNA*, *POLD1*, *EXO1*, *POLE*, *EPCAM* (Suppl. Table 3, blue lines). While not all genes had mutations, missense mutations were found in six of the MMR genes in both unaffected and the affected family members examined and were deemed benign by ClinVar. Only the frame-shifted c154delA mutation in the *MLH1* gene (Suppl. Table 3, green line) predicted by ClinVar to be pathogenic, was found exclusively in the affected individuals, making it the most likely disease causing mutation. A point of interest here, is that the common mutations in the MMR genes that were identified in all four individuals tested, are likely to be LS predisposing gene mutations.

### Neoepitope prediction and prioritization in the tumour of the Lynch syndrome patient (II.2)

Immune surveillance mechanisms have been established to play a vital role in purging transformed cells in the early stages of tumour development^18,19^. The recognition of the tumour as non-self is mediated, in part, by tumour-derived neoepitopes, which are immunogenic peptides arising from intracellular proteolytic processing of somatic mutations in protein coding genes.

In the present study, we wished to first establish if the germline mutant MLH1 peptide was immunogenic in unaffected donors and whether individuals carrying the mutant *MLH1* gene were tolarized to the mutation. To address this, we used our proprietary neoepitope prediction algorithm OncoPept*VAC*, to assess the immunogenicity of 9-mer peptides derived *in silico* from the mutant gene. OncoPept*VAC* an in-house ensemble voting-based machine learning algorithm developed to identify immunogenic peptides from somatic mutations present in the tumour of a cancer patient. Our approach combines the physicochemical properties of amino acids in the peptide which favour T-cell receptor (TCR) binding along with factors important for antigen presentation and processing (expression of the mutant gene in the tumour, HLA type, HLA binding affinity and proteasomal cleavage and processing of the peptide). Our algorithm has been validated on unseen peptides and has been shown to provide sensitivity and specificity of 90.23% and 99.14% respectively (manuscript in review). To test whether the MLH-1 mutant-derived peptide was immunogenic, we first determined the HLA type of two LS unaffected and two LS affected family members from each of the LS families (Suppl. Table 4: Family 1: V.2 (LS^−^ MLH1^mut^), IV.3 (LS^−^ MLH1^wt^) and IV.1(LS^+^ MLH1^mut^), V.1 (LS^+^ MLH1^mut^), Family 2: II.1, III.1 (LS^−^ MLH1^wt^), II.2 and II.6 (LS^+^ MLH1^mut^). We next generated three pairs of 9-mer peptides *in silico* from the wildtype and mutant MLH-1 protein (Suppl. Table 5) and their HLA-restricted immunogenicity was predicted using OncoPept*VAC* (Suppl. Table 5). Our analyses revealed that of the three mutant peptides only one peptide (seq. 1: TSIQVIVKR) showed strong HLA binding with the 3 HLA types expressed in members of Family 1 (highlighted in Suppl. Table 4: IC_50_ HLA-A*68:01 = 10.3 mM: HLA-A*33:03 = 79.1 nM and HLA-A*31:01 = 105.42 nM). In contrast, the IC_50_ for the corresponding wildtype peptides for these HLAs showed a relatively weaker binding affinity (Suppl. Table 5) as determined by NetMHCCons^20^. In comparison, none of the three mutant MLH1 peptide pairs demonstrated strong HLA binding affinity with the HLA types identified in Family 2 (Suppl. Table 6). Our algorithm predicted lack of TCR binding for all three peptide pairs (wildtype and mutant), suggesting that even if the mutant peptides were presented on the surface of antigen-presenting cells (APC) in complex with HLA (owing to their strong HLA binding), they would likely fail to interact with the TCR, and would therefore be incapable of eliciting an immune response, rendering them non-immunogenic. To test our prediction that the selected germline MLH1 mutant peptide had no T cell-activating potential, we performed a CD8^+^ T-cell activation assay.

### A neoepitope derived from the germline *MLH1* gene mutation is not immunogenic

In order to examine the immunogenicity of the prioritized MLH1 peptide pair (Suppl. Table 5, Seq. 1), we first performed a CD8^+^ T cell activation assay in a healthy donor with matching HLAs (HLA-A*68:01, Suppl. Table 7, healthy donor 1). Purified naïve CD8^+^ T cells and monocyte-derived dendritic cells were co-cultured in the presence of either synthetic wild type (TSIQVIVKE) or mutant MLH1 (TSIQVIVKR) peptides (Suppl. Table 5). CD8^+^ T cell activation was measured by intracellular interferon gamma (IFNγ) staining using flow cytometry.

**Figure 4:**
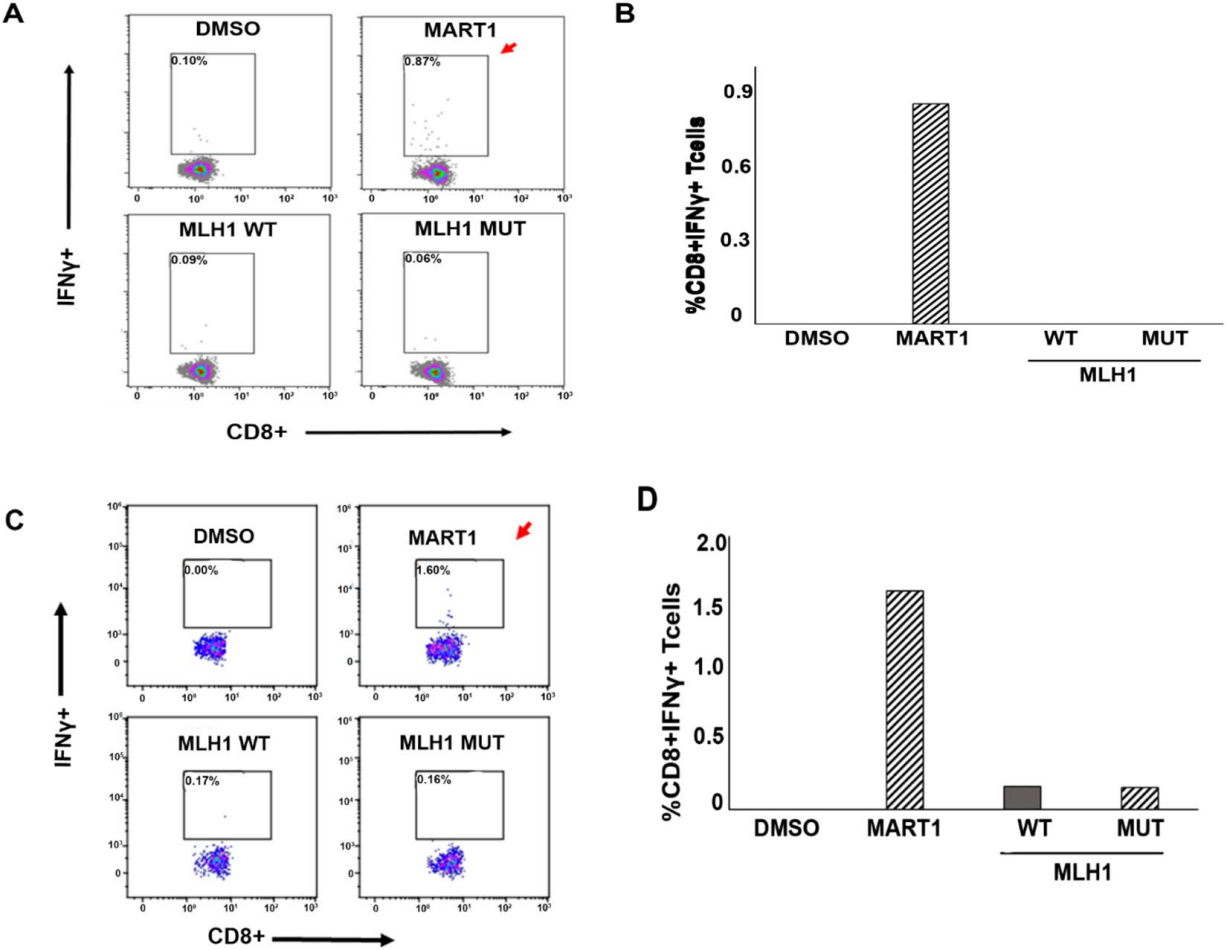
*MLH1*-germline mutation derived peptide tested in a CD8^+^ T cell activation assay in Healthy donor 1 and in a Lynch syndrome patient (Family 2; II.2, LS^+^MLH1^mut^). **(A)** Purified CD8^+^ T cells and monocyte-derived DCs from healthy donor 1 (HLA-A*68:01) was tested for antigen-specific T cell activation using the MART1 peptide and ‘flu peptides (CEF pool, Suppl. Fig. 1) as positive controls and wildtype and mutant *MLH1* peptides (Suppl. Table 5). No increase in percentage of CD8^+^ IFNγ^+^ cells was observed in wildtype or mutant *MLH1* peptide treated cells (bottom panel), whereas the percentage of CD8^+^ IFNγ^+^ cells was increased in MART1 treated cells (positive control, indicated with red arrow, top panel). DMSO was used as negative control. **(B)** Graphical representation of flow-cytometry data in Fig. 4A. **(C)** PBMCs isolated from LS patient: Family 2; II.2, LS^+^ MLH1^mut^, were treated with MART1 (positive control), wildtype and mutant *MLH1* peptides. No increase in percentage of CD8^+^ IFNγ^+^ cells was observed in wildtype or mutant MLH1 peptide-treated cells (bottom panel), whereas the percentage of CD8^+^ IFNγ^+^ cells was increased in both MART1-treated cells (indicated with red arrow, top panel) and ‘flu peptides (CEF pool, Suppl. Fig. 1). DMSO was used as negative control. **(D)** Graphical representation of the Flow cytometry data in Fig. 4C.

No significant increase in the IFNγ producing cell population was observed in three healthy individuals (example: healthy donor 1) in response to either the mutant peptide or wildtype peptides (Fig. 4A,B, and data not shown), suggesting that this MLH-1 germline mutant peptide is incapable of eliciting an immune response in an individual unaffected with LS-CRC. We next tested this peptide using PBMCs from a patient with LS-CRC (Family 2, II.2). Due to the paucity of cells, the CD8^+^ T cell activation assay was performed with patient-derived PBMCs without purifying the cell types (see Methods for details). As is evident in Fig. 4C,D, no significant increase in the IFNγ expressing CD8^+^ T cell population was observed following treatment with the MLH1 wildtype or mutant peptides (Seq. 1; Suppl. Table 5). Our data thus confirm that a peptide derived from the germline mutation in the *MLH-1* gene failed to elicit a CD8^+^ T cell response in both LS unaffected and affected individuals.

### Neoepitopes derived from somatic mutations identified in the tumour of a patient in Family 2 are predicted to be immunogenic

We next considered the possibility that somatic mutations present in the LS-CRC tumour of the patient in Family 2 (II.2) may harbor immunogenic peptides. To this end, targeted deep sequencing (~200X depth) of 6900 genes on a 20MB gene panel (Suppl. Table 8) was performed on DNA isolated from the tumour/blood pair from patient II.2 (Family 2, Fig. 2B). We identified 959 somatic mutations (Suppl. Table 9), of which 663 (69%) were missense, 296 (30%) were INDELs and 30 (<1%) were nonsense mutations (Suppl. Fig. 2). We next screened the somatic mutations for neoepitope prediction and prioritization using OncoPept*VAC*. 289 peptides were predicted to be immunogenic among the somatic missense mutations (TCR binding positive and <1000 nM binding affinity), of which 26% (74) were expressed in the tumour (based on RNA-Seq data). In contrast, a total of 162 immunogenic peptides derived from somatic INDELS (TCR-binding positive and <1000 nM binding affinity) were identified, of which 29 were found to be expressed in the tumour (Suppl. Table 10). Thus, of the total 451 predicted neoantigens in the tumour of patient II.2, 23% (103) were found to be expressed at the transcript level.

Annotation of the identified somatic variants using ClinVar^21^, revealed 20 predicted pathogenic variants (Suppl. Table 11). Of these, 70% (14/20) were found to be frame-shift mutations and 25% were present in known CRC associated genes (*BAX*, *AXIN2*, *MSH6*, *APC and MSH3 genes*). 9-mer peptides from both wildtype and mutant proteins, were theoretically generated for all 20 pathogenic variants and tested *in silico* for their immunogenicity using OncoPept*VAC*. Restricting the mutations to the HLA type of patient II.2 (HLA-A*02:06) our analyses revealed that of all the wildtype and mutant peptide pairs tested, three, (derived from the *AXIN2*, *MSH6* and *PIGO* genes) were predicted to be immunogenic (Suppl. Table 11 highlighted in green and Table 1). All three mutant peptides were predicted to bind HLA with high affinity and two of the three were predicted to be TCR-binding as well (Table 1). Mutations in the three genes were previously detected in stomach and colorectal cancers (Suppl. Table 11; COSMIC and OncoMD databases).

**Table 1:** Peptide binding prediction of three somatic mutations derived from patient II.2 LS^+^ MLH1^mut^.

Gene	Variant	HLA	WT peptide sequence	WT peptide affinity (nM)	Mutant Peptide Sequence	Mutant peptide affinity (nM)	TCR binding prediction	Mutation reported in COSMIC (# of times)
MSH6	p.F1088LfsX5	HLA-A*02:06	LLPEDTPPF	71.02	LLPEDTPPL	8.61	Deprioritized	6
PIGO	p.T788LfsX25	HLA-A*02:06	QADLDYVVP	32787.64	KLTWIMWSL	10.3	High	4
AXIN2	p.G665AfsX24	HLA-A*02:06	HLWGGNSGH	12860.25	HLWGATAGT	425.67	High	46

**Figure 5:**
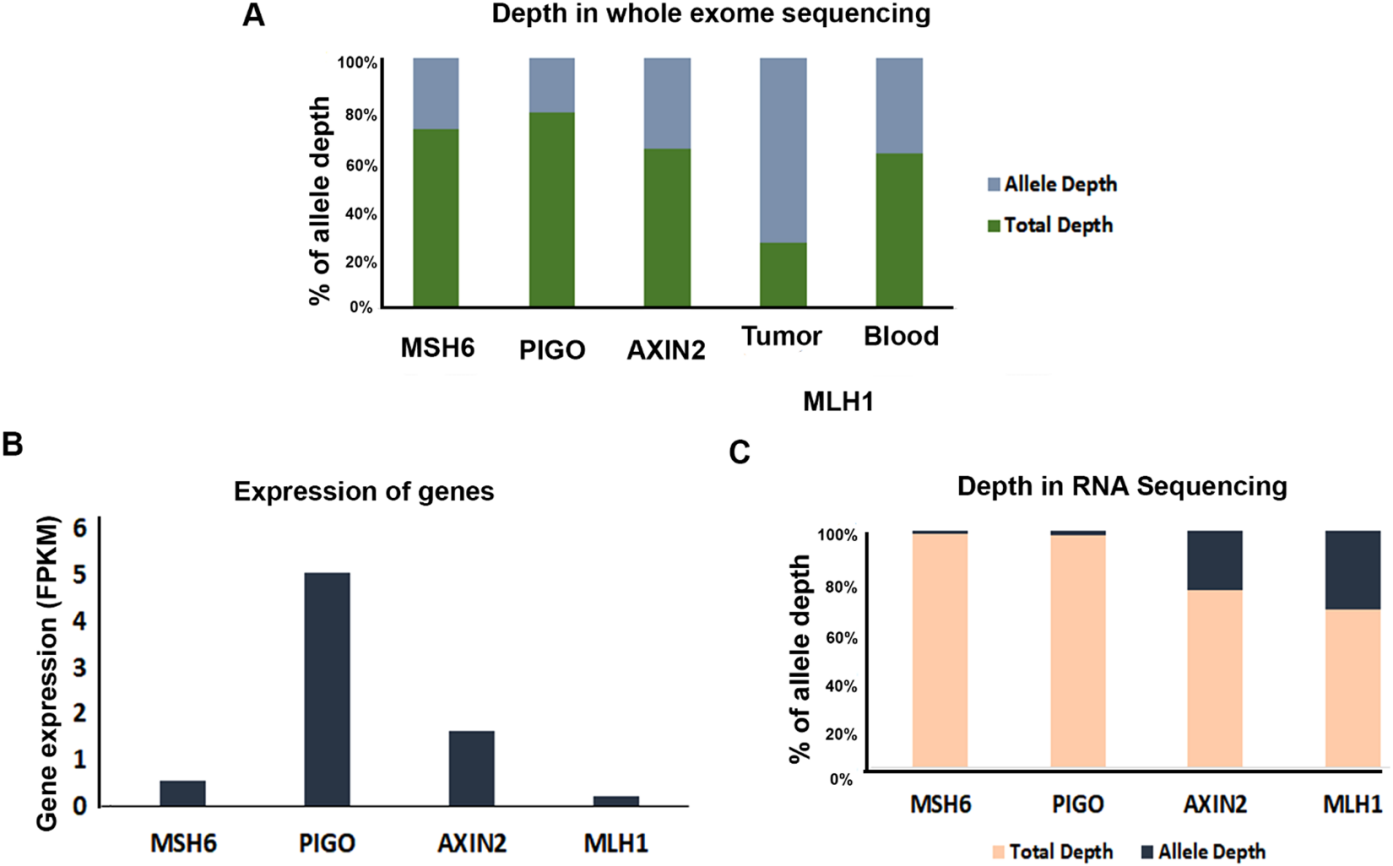
Expression analysis of four genes in the tumour of patient Family 2; II.2 LS^+^MLH1^mut^ **(A)** Whole exome sequencing was performed with the tumour sample collected from patient Family 2; II.2 LS^+^ MLH1^mut^. The bar diagram represents the percentage of wildtype and alternate allele depth of *AXIN2*, *MSH6*, *PIGO* and *MLH1* genes. **(B)** Expression levels of four genes were determined using RNA Seq analysis (FPKM: Fragment per kilobase per million). **(C)** Percentage of wildtype and mutant allele expression determined by RNA seq analysis.

Whole exome sequence (WES) confirmed the presence of the mutant germline *MLH1* and the three somatic mutations as measured by percent allele depth (Fig. 5A). While the allele depth of *MSH6*, *PIGO* and *AXIN2* mutant alleles ranged from 20–39% in the tumour, the allele depth of the *MLH1* mutant in the tumour (73%) was found to be nearly double compared to that in the blood (germline variant, 38%), suggesting a possible second hit in the *MLH1* gene in the tumour cells.

To determine if the three somatic gene variants were in fact expressed in the tumour of patient II.2, we performed RNA-seq analysis from the tumour tissue of this patient. As evidenced in Fig. 5B, expression data indicate that all three genes are expressed in the tumour of the LS patient, with the *PIGO* and *AXIN 2* genes expressing relatively high levels. Surprisingly, expression levels of the germline *MLH1* gene were found to be low (Fig. 5B), possibly due to loss of the gene product from both alleles in the majority of the cells in the tumour. Verification of the presence of each of the mutants in the tumour at the RNA level was gauged by documenting allelic depth of the RNA transcripts. Figure 5C shows that while *AXIN2* and the germline *MLH1* mutant alleles are well represented (20 and 33% respectively); *PIGO* and *MSH6* transcripts were present at low but detectable levels. Taken together, our data confirm the presence of all four mutant genes in the genome of the patient and confirm the RNA expression of the three somatic variants in the tumour of patient II.2, albeit at varying levels.

### Peptides derived from the three mutant genes identified in the tumour of patient II.2 are immunogenic

**Figure 6:**
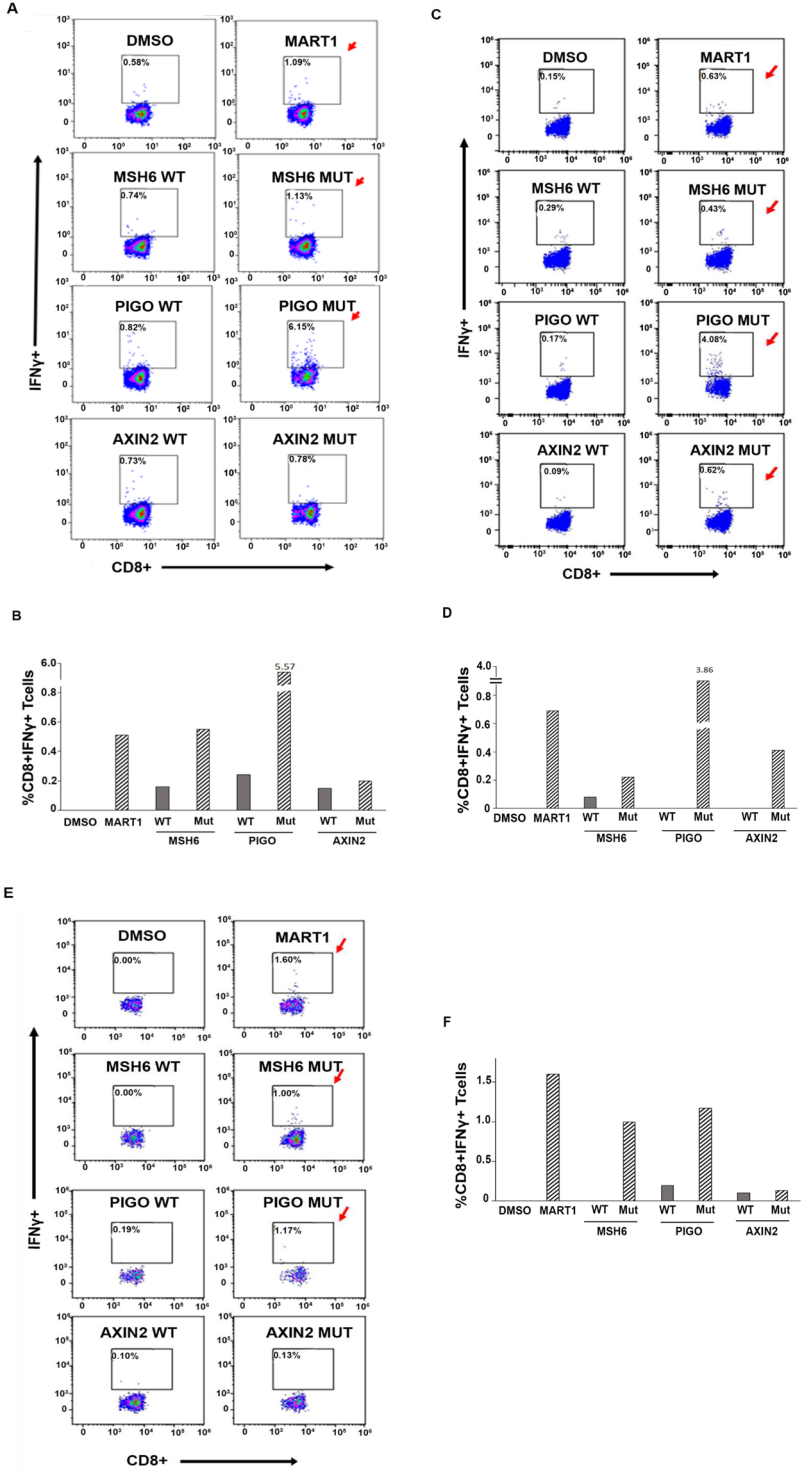
Somatic mutation derived peptides tested in a CD8^+^ T cell activation assay in healthy donor 2, healthy donor 3 and patient Family 2; II.2; LS^+^ MLH1^mut^. **(A)** PBMCs isolated from healthy donor 2 (HLA-A*02:11) were treated with MART1 peptide (positive control), wildtype and mutant peptides derived from frameshift somatic mutations found in *MSH6*, *AXIN2* and *PIGO* genes. Flow-cytometry data revealed that along with the MART1 peptide and ‘flu peptides (Suppl. Fig. 1), *MSH6* and *PIGO* derived mutant peptides also evoked a measurable T-cell activation response (*MSH6:* 1.9-fold and *PIGO*: 7.5-fold above the wildtype peptide) as measured by a fold increase in percentage of CD8^+^ IFNγ^+^ T cells (indicated by the red arrows). No CD8^+^ T cell activation was observed with the mutant *AXIN2* peptide. DMSO was used as negative control. **(B)** Graphical representation of the Flow cytometry data in Fig. 6A. **(C)** PBMCs isolated from healthy donor 3 (HLA-A*02:01) were treated with MART1 (positive control), wildtype and mutant peptides derived frameshift somatic mutations found in *MSH6*, *AXIN2* and *PIGO* genes. Flow-cytometry data revealed that along with the MART1 peptide, *MSH6*, *PIGO* and *AXIN2* mutant peptides also evoked a robust T-cell activation response (*MSH6*: 1.5-fold, *PIGO*: 23.5-fold, *AXIN2*: 6.9-fold above the wildtype peptide) as measured by a fold increase in percentage of CD8^+^ IFNγ^+^ T cells (indicated by red arrows). DMSO was used as negative control. **(D)** Graphical representation of the Flow cytometry data in Fig. 6C. (**E**) PBMCs isolated from Family 2; II.2 LS^+^MLH1^mut^ (HLA-A*02:06) were treated with MART1 (positive control), wildtype and mutant peptides derived frameshift somatic mutation found in *MSH6*, *AXIN2* and *PIGO* genes. Flow cytometry data revealed that along with the MART1 peptide and the ‘flu peptides (Suppl. Fig. 1), *MSH6* and *PIGO* mutant peptides also evoked a robust T-cell activation response (*PIGO*: 6.1-fold) as measured by a fold increase in percentage of CD8^+^ IFNγ^+^ T cells (indicated by red arrows). A moderate CD8^+^ T cell activation was observed with the mutant *AXIN2* peptide (1.3 fold). DMSO was used as negative control. (**F**) Graphical representation of the flow-cytometry data in Fig. 6E.

Next, we assessed the immunogenicity of the wildtype and mutant peptides derived from the three selected genes in patient II.2’s tumour sample. The peptides were first tested on the naive T cell repertoire of two healthy individuals with matching HLAs (Suppl. Table 7). Our analyses revealed that the MSH6 and PIGO mutant peptides evoked a measurable CD8^+^ T-cell activation response (1.9 fold and 7.5 fold above the wildtype peptide) as measured by an increase in IFNγ expressing cells by FACS analysis (Fig. 6A,B). We failed to observe CD8^+^ T cell activation with the mutant AXIN2 peptide in this donor. However, in a second healthy donor, all three mutant peptides showed a robust response (MSH6: 1.5-fold, PIGO: 23.5-fold, AXIN2: 6.9-fold) (Fig. 6C,D) suggesting that all three mutant peptides have immunogenic potential in healthy individuals with the appropriate HLAs.

We next examined whether these mutant peptides were capable of eliciting an immune response in the LS-CRC patient from Family 2, (II.2, Fig. 2B). As shown in Fig. 6E,F, two of the mutant peptides, MSH6 and PIGO significantly increased IFNγ production compared to the corresponding wildtype peptides. A modest, but measurable increase in CD8^+^ T cell activation was also observed in the presence of the mutant AXIN2 peptide (1.3 fold) compared to the wildtype peptide.

Of note, the immunogenic response of these peptides seemed to be inversely proportional to the expression levels of the respective mutant genes. A clear and definitive inverse relationship between CTL responses and the percent allele depth of the mutations in the MSH6, PIGO, AXIN2 and MLH1 genes has been demonstrated in Suppl. Fig. 4, where genes with a low percent allele depth have a larger percentage of activated CTLs (MSH6 and PIGO genes) while genes that have a higher (>20%) allele depth demonstrate a lower percentage of CD8^+^ IFNγ^+^ T cells (AXIN2 and MLH1).

Taken together, our results strongly support the notion that the three mutant peptides derived from the tumour of the Lynch syndrome patient with CRC are immunogenic in the LS-CRC patient as well as in HLA-matched normal healthy donors.

### RNA expression analysis of the Lynch syndrome-CRC patients (II.2) tumour predicts the presence of tumour infiltrating lymphocytes (TILs) and myeloid cells

Having determined the immunogenic potential of three peptides in the Lynch syndrome patient, we analyzed the tumour transcriptome to predict the type and nature of tumour infiltrating immune cells. To this end, we applied our proprietary gene-expression signature-based tumour microenvironment analysis pipeline, OncoPept*TUME* which is a genomic solution that utilizes a cell-type specific minimal gene expression signature for eight different immune cells (manuscript in review). Genes were included in the signature if they satisfied two criteria: 1) if the Average Rank Score (ARS) of the gene was high, that is, the gene was selectively highly expressed in a given cell type compared to all other cell types examined and 2) if the Marker Evaluation Score (MES) of the gene was high, that is, the gene is stably expressed in multiple independent gene expression data sets^22^. The expression of genes for a given signature was transformed using Single Sample Gene Set Enrichment Analysis (ssGSEA)^23^ to generate a cell-type specific immune score, which was used to quantitate the relative proportion of cell types present within the complex tumour microenvironment.

**Figure 7:**
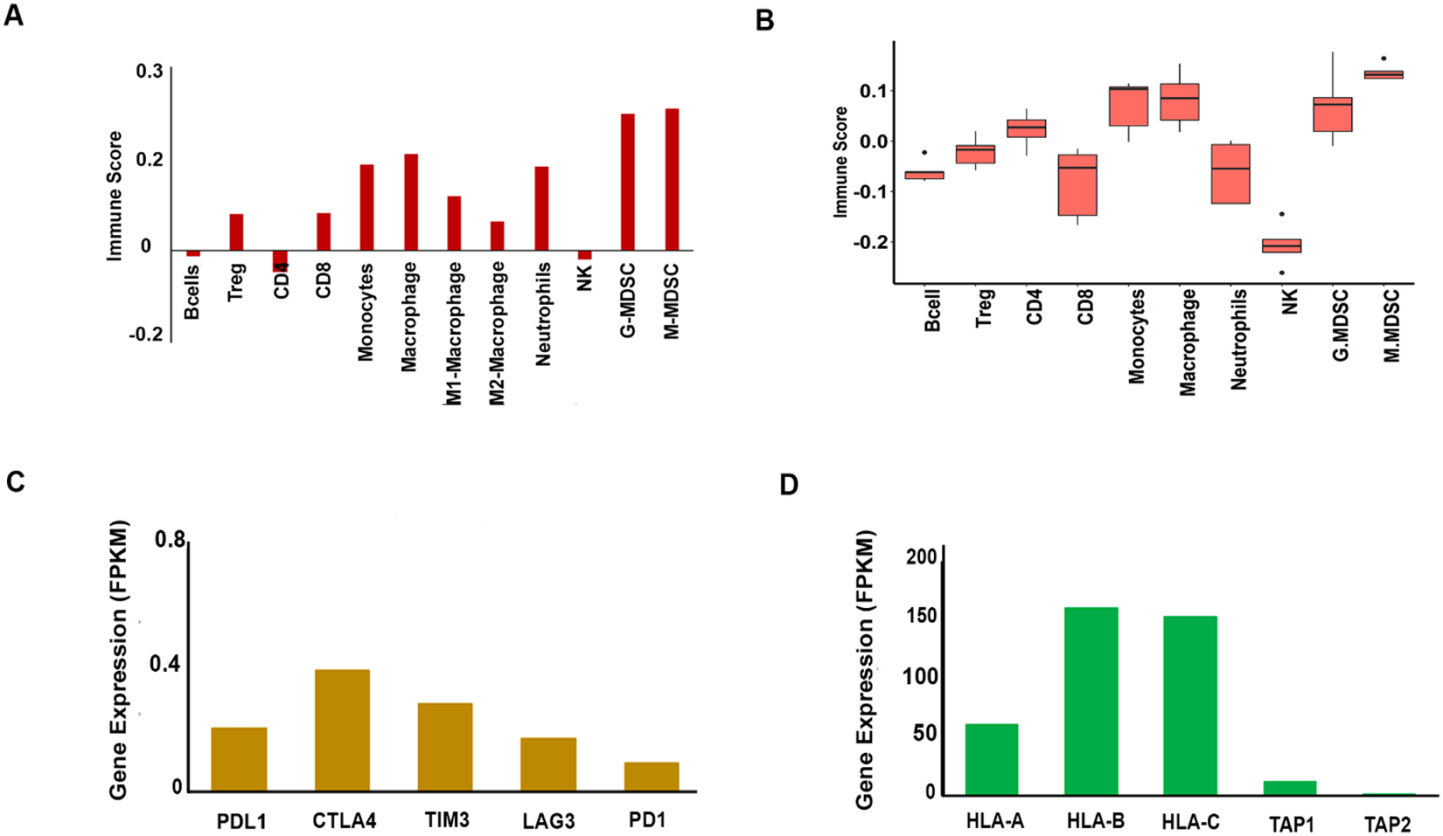
Tumour microenvironment analysis of sample collected from LS patient Family 2; II.2; LS^+^ MLH1^mut^. **(A)** RNA expression data analyzed by the OncoPept*TUME* algorithm shows the presence of various tumour infiltrating cells based on the Immune score distribution. CD8^+^, CD4^+^ T cells, B lymphocytes, NK cells, Macrophage, Monocyte, Neutrophil and Treg cell signature markers were found to be present in the tumour sample of patient Family 2; II.2; LS^+^ MLH1^mut^. **(B)** OncoPept*TUME* analysis of tumour infiltrating cells in 5 MSI-H tumours with MLH-1 mutations from TCGA. **(C)** RNA expression of checkpoint molecules like PD-1, PD-L1, CTLA-4 and LAG-3 were found to be low in the tumour. **(D)** Expression of HLA alleles and transport protein TAP1 and TAP2 in the tumour of patient Family 2: II.2; LS^+^ MLH1^mut^.

Based on our OncoPept*TUME* analysis, we identified a number of tumour infiltrating immune cell types (Fig. 7A,B), including all of the major innate and adaptive immune cell types in the tumour microenvironment of the LS patient. Based on the immune score assigned to each cell type (see Methods), B-cells, CD4^+^ T cells and NK cells were absent in the tumour environment (Fig. 7A), while CD8^+^ T cells, monocytes, M1 and M2 macrophages were present. Significant levels of G-MDSC and M-MDSCs were also noted in this tumour (Fig. 7A). The profile of tumour infiltrated immune cells in the LS-CRC patient’s tumour matched that of five *MLH-1* mutant MSI-H CRC tumours obtained from TCGA (The Cancer Genome Atlas), with the exception of CD8^+^ T cells, which appeared to be higher in the LS-CRC tumour (Fig. 7B).

Many studies have shown that the functional state of CD8^+^ T cells in the tumour microenvironment is indicative of an inflamed tumour phenotype (reviewed in ref.^24^). Analysis of expression of the checkpoint proteins at the RNA level in the LS-CRC tumour indicated low expression of all the markers, including *PD1*, *PD-L1*, *CTLA-4*, *TIM3 and LAG3* (FPKM values <1) (Fig. 7C). These low expression levels correlate with the absence of activated CD8^+^ T cells in the tumour microenvironment. The abundance of Treg and MDSC cells may have contributed to the lack of activated T cells, despite the expression of immunogenic antigens in the tumour^24^. Our analysis also revealed modest expression of the *HLA* genes, including low expression of peptide transporters TAP1 and TAP2, in the LS-CRC tumour (Fig. 7D). Of note, we detected two loss- of- function mutations in the *HLA-A* allele, one resulting in the introduction of a stop codon after amino acid 99 (p.AGr99Ter)) and the other resulting in a frameshifted termination transcript (p.Lys210GlnfsTer11). While the allele frequency of both the HLA-A mutations was found to be 20–30% at the DNA level (Suppl. Fig. 1A), their RNA expression levels were low (<1% allele depth, Suppl. Fig. 1b). Furthermore, the relatively low expression of HLA-A in the tumour (Fig. 7D) could be due to the loss of function mutations in the HLA-A allele in some clones within the tumour, making them unresponsive to neoepitope-driven T-cell activation, thereby contributing to relapse of the disease in LS patient II.2.

## Discussion

A system of MMR proteins, including MLH1, MSH2, MSH3, MSH6, PMS1, PMS2, Exonuclease 1 and the DNA polymerases δ and ε, normally work co-ordinately to orchestrate the process of repairing DNA mismatches in the human genome^25,26^. Mutations in the MMR genes, which give rise to a high mutational burden due to the resultant genomic instability, have been shown to occur in approximately 15% of colorectal cancers, with *MLH1*, the human homolog of the *E*.*coli* DNA mismatch repair (MMR) MutL gene, being the most commonly mutated gene^27^. We identified an *MLH1* heterozygous mutation (chr3:37038147;c.154delA; p.Glu53ArgfsTer) in five individuals in two unrelated LS families, four of whom had progressed to CRC. Analysis of the germline NGS data of two affected and two unaffected LS family members (Family 2) identified common missense mutations in six MMR genes, (Suppl. Table 3, blue lines) in both unaffected and affected family members, all of which were predicted to be benign by ClinVar. Only the frame-shifted c154delA mutation in the *MLH1* gene (Suppl. Table 3, green line), predicted by ClinVar to be pathogenic, was found exclusively in the affected individuals, making it the most likely disease-causing mutation. A point of interest however, is that the common mutations in the MMR genes that were identified in all four individuals tested, are likely to be LS predisposing gene mutations.

Affected LS-family members who progressed to CRC were 40, 42 and 36 years old at the time of diagnosis. Considering that CRC associated with Lynch Syndrome manifests at an early age (mean age 45 years, see ref.^28^), the presence of the *MLH1* gene mutation in a 51 year old member of Family 1 (Fig. 2; V.2) who currently remains cancer free, remains unexplained. It is tempting to speculate that this individual lacks the second hit in the *MLH1* gene or in other predisposition genes, which together with the *MLH1* gene mutation could result in CRC. Counselling and regular colonoscopies have been recommended for this disease- free individual carrying the *MLH1* mutation. Two of the three LS-CRC patients had a history of tobacco chewing and smoking. Whether tobacco abuse may have exacerbated manifestation of carcinoma in the LS-CRC patients remains speculative.

Somatic mutation analyses of the tumour derived from an LS affected family member, revealed a high mutational burden (959 mutations) in a clinical exome panel. Given that LS is an MMR-deficient MSI-H cancer, the exceptionally high number of mutations observed is not surprising. Analysis of the mutant genes in this patient revealed variants in a number of MMR-associated genes, in addition to the germline *MLH-1* gene. These included mutations in *MSH6*, *MSH3*, *MSH2* and *Exonuclease 1* (see Fig. 1, red arrows; Suppl. Table 9). The presence of these mutations in this patient with relapsed LS-CRC, very likely hinders or completely blocks the MMR process, accounting for the high mutational burden in the patient’s tumour. Analysis of this heavy mutational burden has also shed light on progression to CRC. Individuals with germline mutations in the *MLH-1* gene remain disease free, presumably because they have one normal copy of the *MLH-1* gene and can therefore evoke a normal MMR response. Considering the mutant allele depth of the *MLH1* gene in the tumour of the LS-CRC patient (II.2, 73%) is about two fold higher than in the blood (germline, 38%), a second hit in the *MLH-1* allele is likely to have occurred, thereby contributing to CRC progression. Additional mutations in genes associated with the MMR pathway in this patient are likely to have exacerbated genomic instability leading to accelerated transformation to CRC. Furthermore, mutations in genes associated with the Wnt-β catenin pathway, which is commonly affected in CRC, were also observed in this patient, including loss of function mutations in the *APC* and *AXIN2* genes, the protein products of which normally work in concert to stabilize the β-catenin protein and to prevent it from translocating into the nucleus to activate aberrant cell proliferation and cell survival programs^29^. The apparent deregulation of cardinal pathways, namely the MMR and the Wnt-βcatenin pathways undoubtedly contribute to the malignant state in this patient.

In the recent era of immunotherapy, neoepitopes offer opportunities to harness the immune system’s ability to recognize and to ultimately destroy the tumour^30^. The utility of such neoantigens as vaccines for the treatment of both LS-CRC patients as well as LS-mutation carriers offers a promising approach to both treat and to potentially prevent the tumour from relapse^31^. Such a strategy involves the use of a neoantigen-specific effector and memory T-cell response to eliminate tumour cells. We selected predicted immunogenic neoantigens using OncoPept*VAC* and tested wildtype and mutant pairs of peptides for their immunogenicity in a CD8^+^ T cell activation assay. We found that the mutant peptide generated from the germline *MLH1* gene was unable to elicit an immune response in either normal healthy donors or in the LS-CRC affected patient, suggesting that the mutation is intrinsically non-immunogenic.

For a peptide to be considered immunogenic, two conditions must be met: a) it should be presented on the surface of cells in conjunction with the appropriate HLA molecule and b) the peptide should bind to and activate the T-cell receptor (TCR) expressed on CD8^+^ T cells. The peptide derived from the frame-shifted mutation in the MLH1 gene, TSIQVIVKR, was predicted to have a high binding affinity (<150 nM) to HLA-A*02, but did not demonstrate features of robust TCR binding, as judged by OncoPept*VAC*. Therefore, despite the high probability of the peptide being presented on the surface of antigen presenting cells, it appeared to be incapable of engaging and activating the TCR. Our experimental demonstration that this peptide failed to induce an antigen-specific T cell response in unaffected donors, strongly supports the prediction based on our algorithm.

In contrast, mutant peptides derived from three somatic frame-shift mutations in the *AXIN2*, *MSH6 and PI*GO genes evoked T cell activation responses of varying magnitude both in normal healthy donors as well as in the LS-CRC patient. Our findings lead us to conclude that these immunogenic peptides have the ability to generate clonally amplified CD8^+^ T cells against the tumour, and could therefore be used towards the generation of an anti-tumour vaccine. A point of note, while all three mutant peptides in our study were found to be immunogenic in healthy donor 3, only two out of three mutant peptides (PIGO and MSH6) were immunogenic in healthy donor 2. This type of discrepancy has been observed previously, but remains mechanistically unexplained to date^32^.

Recent clinical trials have shown that the mismatch-repair status of a CRC tumour predicted a marked clinical benefit of immune checkpoint blockade therapy^33,34^. In this context it has come to light that the tumour microenvironment plays a vital role in modulating the response of checkpoint blockade therapy and eventually to patient outcomes. Intratumoural heterogeneity driven by genetic changes favor tumours to survive in an environment beset with hostile immune cells actively participating in immune surveillance and tumour clearance. Tumour growth and heterogeneity are known to be driven by immune-editing processes, which impede recognition and destruction of antigen expressing tumour cells by CTLs^24^. We observed a strong inverse correlation between the highly immunogenic PIGO and MSH6 peptides, which evoked a potent CTL response in the patient and the low expression levels of these transcripts. This inverse relationship strongly suggests a mechanism by which tumour cells evade immune attack by down-regulating the expression of highly immunogenic neoepitopes.

A second mechanism that tumour cells employ to evade immune attack is by down-regulating their antigen presenting machinery, including reducing expression of the HLA, β-microglobulin and/or peptide transporter genes. (rev in ref.^35,36^).

Immune-escape involves the establishment of an immunosuppressive tumour microenvironment which blunts the function of CTLs thereby protecting tumours from immune-mediated elimination. The survival benefit of patients with MSI-H tumours strongly correlates with high expression of the *PD1* gene in CD8^+^ T cells, coupled with reduced expression of the anergic or exhaustion markers, TIM1 and LAG3^24^. The LS-CRC tumour, while infiltrated with CD8^+^ T cells, lacked expression of both activation and exhaustion markers. The recruitment of CD8^+^ T cells in the LS-CRC tumour is likely due to the observed elevated expression of a number of CD8^+^ T cell-mobilizing chemokines in the tumour microenvironment (data not shown), coupled with the high neoantigen burden and increased immunogenic peptides noted in this patient (II.2; Suppl. Table 9). It is therefore reasonable to assume that the infiltered CD8^+^ T cells should exhibit properties of activation (expression of PD-1) or activation leading to exhaustion (expression of PD-1, LAG3, TIM3 and CTLA-4). We however, observed neither of these phenotypes exclusively in the tumour-infiltrated CD8^+^ T cells in this relapsed tumour. Based on our observations, we propose two mechanisms that could explain T cell suppression in the LS patient (II.2) tumour: a) the finding of two truncating mutations in the HLA-A*02 allele which may have rendered the tumour partially invisible to the infiltrating CD8^+^ T cells, given that a significant proportion of the predicted immunogenic peptides in this tumour were restricted to HLA-A*02 and b) the high infiltration of Treg and MDSC cells in the tumour microenvironment. Treg cells limit T cell activity through production of TGF-β or by interfering with clonal expansion of T cells^39^. Similarly MDSC cells exert their strong T cell suppressive activity by blocking the expression of L-selectin and producing IL-10, thereby preventing migration to the lymph nodes^40^. Targeting MDSCs in these tumours could thus help release the T cell immunosuppression. In this regard, two very widely used adjuvant chemotherapy drugs, 5-Fluorouracil and Oxaliplatin, trigger MDSC cell death. A combination of Oxaliplatin and IL-2 has not only been shown to reduce MDSCs but also to increase the CD8^+^ T/Treg cell ratio. Therefore using a personalized vaccine approach in combination with adjuvant chemotherapy could serve as a viable approach to gain long term clinical benefit for LS-CRC patients^40^.

In recent clinical trials involving anti-PD1 and anti-CTLA 4, a variable response rate ranging from 26% to 57% has been observed in late stage MSI-H metastatic CRCs^37^. A combinatorial approach with neoantigens vaccines could potentially improve this response rate^38^.

In conclusion, we have identified a germline mutation in the *MLH1* gene in members of two unrelated LS families, the majority of whom have progressed to CRC. While a mutant peptide derived from this germline mutation was nonimmunogenic, a number of neoantigens from the tumour were predicted to be potentially immunogenic. We selected three immunogenic peptides from genes closely associated with CRC - *AXIN2*, *PIGO* and *MSH6* and demonstrated their immunogenicity in a CD8^+^ T cell activation assay, in both healthy donors and in PBMCs derived from an LS-CRC patient. Our study suggests that a cancer vaccine approach could be used either as monotherapy or in combination with established immuno-oncology or chemotherapy drugs, to treat Lynch syndrome patients with mutations in MMR-associated genes and those who have progressed to colon cancer, to prevent relapse and to possibly achieve a cure.

## Methods

### Study subjects

Family 1: The unaffected patriarch of this family approached us at the Kailash Cancer Hospital and Research Centre (KCHRC) Goraj, in rural India, with the observation that several members of his family were affected with colorectal cancer, many of who had since deceased. Following Ethics Committee approval of this study at KCHRC, blood samples were collected by venipuncture in EDTA tubes from all living individuals, in accordance with the norms established by the KCHRC Ethics Committee, Goraj (Gujarat, India), as shown in Fig. 1A. All study participants provided written informed consent prior to the onset of this study.

Family 2: The proband (II.2, Fig. 1B) was diagnosed with Lynch syndrome at KCHRC and was surgically operated for the removal of an adenomatous tumour following a biopsy which confirmed malignancy. He presented at KCHRC after a decade with relapsed disease, with a pT3(2)N1bMx mucinous adenocarcinoma which was surgically treated and followed up by standard of care chemotherapy (Capecitabine (500 mg) and Oxaliplatin (150 mg); 6 cycles at 21 day intervals. No radiotherapy was given). Treatment naïve samples of both blood and tumour were collected from the proband. The tumour sample was flash frozen and preserved at −80 deg C for future use. His brother (Fig. 1B, II.5) had previously been diagnosed and treated for Lynch syndrome-CRC at a different hospital, hence tumour samples from this individual were not available. All thirteen members of this family signed a written informed consent document prior to blood sample/tumour collection. Ethics Committee approval was obtained at KCHRC before this study was initiated.

Blood samples obtained from normal healthy de-identified blood donors at the Narayana Hridayala Hospital (Bangalore, India) following ablation of packed red cell and platelets, were used to isolate PBMCs as a source of control samples, described below. These blood donors had been tested, as part of a routine Blood bank protocol, and found to be negative for HIV, Hepatitis B and C viruses. All experimental protocols conducted as described below on samples collected from human subjects were conducted at MedGenome Labs, and approved by the KCHRC Ethics review committee.

### Mutational analysis

Genomic DNA was extracted from all blood samples using a Qiagen kit (QIAsymphony DNA midi Kit, Cat # 931255) as per protocols provided (Qiagen, Germantown, MD, USA). To identify the causal gene variant in both families, targeted next generation sequencing (NGS) was performed on two affected and two unaffected family members in Family 1 (Fig. 2A, IV.1, IV.3, V.1 and V.2) and two affected and two unaffected members of Family 2 (Fig. 2B II.1, II.2, II.6 and III.1). Libraries were prepared with the extracted DNA using a Kapa Biosystems kit as per manufacturers instruction (Massachusetts, USA), and hybridized on a custom designed 20MB panel (details of this panel are provided in the Suppl. Table 8) following the manufacturer’s protocol. Libraries were then subjected to paired-end sequencing on an Illumina HiSeq. 2500, leading to the identification of a germline mutation in the *MLH1* gene (see above). All family members from both families were then screened for confirmation for the presence of the identified mutation in the *MLH1* gene by Sanger sequencing using standard protocols on an ABI 3730xl instrument using the following oligomers purchased from PxLence (Ghent, Belgium) (Cat #: PXL A0182967; context sequence: 3:36996535–36996928).

### RNA Sequencing of proband from Family 2 (II.2)

RNA was isolated from tumour tissue of the proband using a Qiagen Kit as per manufacturer’s instructions. Library preparation was done using the TruSeq RNA Library Prep Kit V2 (#RS-122–2001/RS-122–2002, Illumina Inc, USA) as instructed by the manufacturer, cDNA was prepared, adapters ligated and PCR amplified to generate a cDNA library. The libraries were quantified and subjected to sequencing on an Illumina HiSeq 4000 with 100 bp paired end chemistry.

### Bioinformatics analysis

Bioinformatics analyses were performed on the raw DNA sequence data obtained. Briefly, adapter trimming using the Fastq-Mcf tool of the raw data obtained was carried out followed by alignment to the human reference genome (hg19) using the BWA aligner. The best practices GATK germline variant calling workflow was used to call SNPs and short INDELs. The aligned reads were then sorted and de-duplicated using Picard. Realignment and recalibration was next performed using GATK. Large insertions and deletions were called using the Pindel program. The Bedtools program was used to calculate target region coverage. All variants obtained were annotated using MedGenome’s in-house variant annotation toolkit (VariMAT) which performs deep annotations including gene, disease and common polymorphism annotation. Disease annotation was performed against HGMD, OncoMD (a proprietary in-house data base), OMIM, GWAS and ClinVar while common polymorphism annotation was obtained against the 1000-Genome, ExAC, dbSNP, ESP, 1000Japanese, dbSNP databases.

### RNA-seq analysis

The paired-end reads were aligned to the reference human genome Feb. 2009 release downloaded from UCSC database (GRCh37/hg19). The chromosome fasta file was downloaded from the following website (http://hgdownload.soe.ucsc.edu/goldenPath/hg19/bigZips/chromFa.tar.gz). GTF file was downloaded from the following website: (ftp://ftp.ensembl.org/pub/release75/gtf/homo_sapiens/Homo_sapiens.GRCh37.75.gtf.gz). Alignment was performed using STAR (2.4.1). The aligned reads were used to estimate expression of the genes and transcripts (or mRNAs) using HTSeq. All expression values are reported as normalized read count units for each of the genes and transcripts.

### OncopeptTUME pipeline analysis of RNA-Seq data

Specific gene signatures were estimated for each of the immune cell types using a robust pipeline, OncoPept*TUME*. Briefly, OncoPept*TUME* uses non-overlapping cell type specific gene expression signatures for eight immune cell types including CD8^+^ T cells, CD4^+^ T cells, Macrophages, Monocytes, Neutrophils, B-cells, Treg and NK cells, to estimate infiltration of these cells in the tumour microenvironment (manuscript in review). Single Sample Gene Set Enrichment Analysis (ssGSEA) is used to score each sample with respect to the eight cell types based on the cell type specific signatures. ssGSEA is a well-known method that uses Empirical Cumulative Distribution Functions (ECDF) of the genes in a given signature and the remaining genes to derive an enrichment score. The score generated is a composite value calculated from the expression level of all genes in the signature. Scores are high if all the genes are coordinately regulated. Thus, the co-expression level of genes belonging to a signature defines the relative abundance of a specific cell type (manuscript in review).

### HLA typing

HLA typing of the following family members was performed on Family 1 IV.1, IV.3, V.1, V.2 (Fig. 2A) and Family 2 II.1, II.2, II.6 and III.1 (Fig. 2B). Genomic DNA isolated from the blood of the indicated individuals was subjected to amplification of the HLA locus-specific regions using long range PCR with proprietary HLA locus-specific primers supplied in the GenDx amplification kit (Utrecht, The Netherlands). Single indexed libraries were prepared from the HLA amplicons using the KAPA Biosystems kit (Massachusetts, USA). The libraries were sequenced on an Illumina HiSeq. 4000 HT system. Data was analysed by the GenDx software to predict the HLA type.

### Neoepitope prediction and peptide synthesis

Neoepitope prediction and prioritization was performed using the MedGenome’s proprietary OncoPept*VAC* neoepitope prediction pipeline (manuscript in review). Briefly, the prediction pipeline combines a novel TCR-binding prediction algorithm with other commercial tools for peptide-MHC binding (NetMHCcons 1.1, http://www.cbs.dtu.dk/services/NetMHCcons/), the peptide-processing module of IEDB (netChop 3.1 http://www.cbs.dtu.dk/services/NetChop/) and peptide TAP binding (http://tools.iedb.org/processing/) to identify peptides that bind to specific HLAs are predicted to be presented by antigen presenting cells and are likely to bind to the T cell receptor (TCR). All synthetic peptides derived from germline or somatic mutations to be tested in the T-cell activation assays described below were synthesized at JPT Peptide Technologies, (Berlin, Germany).

### T cell activation assay

The T-cell activation assay was performed using two methods. Peripheral blood mononuclear cells (PBMCs) were isolated from heparinized blood collected from consented donors (Fig. 2A,B as indicated above) using a standard Ficoll gradient (GE Healthcare, USA). The isolated PBMCs were then frozen and stored in liquid nitrogen for later use for both methods used. In the first method, the naïve CD8^+^ T cell activation assay was performed as described previously^41^. Briefly, monocytes from thawed PBMCs were isolated by the adherence method, differentiated into dendritic cells and pulsed with synthetic wild type or mutant 9-mer peptides. Autologous naïve CD8^+^ T cells were isolated using the MACS cell separation method (Miltenyi Biotec, Cologne, Germany) and co-cultured with peptide- pulsed dendritic cells. After culturing for 10 days, PBMCs pulsed with peptides were used to restimulate the T cell culture. Intracellular cytokine staining was performed using the BD Fast immune CD8 intracellular cytokine kit (Cat. No. 346048) 48 h post stimulation.

The second assay was performed as follows: PBMCs cultured in 0.5 ml RPMI (Gibco) +10% Human AB serum (Sigma) was pulsed with 5 µM of synthetically synthesized peptides (JPT). DMSO treatment was used as a control. 10 ng/ml IL-15 and 10 IU of IL-2 (Peprotech, Rehovot, Israel) was added to each well the next day. The culture media was replenished every three days with fresh media containing 10 IU of IL-2 and 10 ng/ml IL-15. On the 7^th^ and 14^th^ and 21^st^ days of incubation, fresh peptides were added to the respective wells. On day 22, Brefeldin A (BD Biosciences) was added for 6 hrs and cells were fixed, permeabilized using BD Lysis solution and Perm2 solutions respectively and stained with antibodies. Stained cells were analysed in a Beckman Coulter Navios Flow Cytometer (Beckman Coulter, USA) to detect the expression of the T cell activation markers, CD69 and IFNγ. Data was analyzed using Kaluza software (Beckman Coulter, USA).

### Human subjects

Written informed consent was obtained from all human subjects included in this study. All methods and protocols involving human samples were conducted in accordance with guidelines and regulations followed at MedGenome Labs Pvt Ltd, a CAP/CLIA accredited facility. All experimental methods were approved by the KCHRC, Goraj Institutional Ethics Committee prior to implementation.

## Electronic supplementary material

Supplementary Dataset 1

## Data Availability

The datasets generated during and/or analysed during the current study are available from the corresponding author on reasonable request.
