# Supplementary material for: A cancer vaccine approach for personalized treatment of Lynch Syndrome
Source: Sci Rep. 2018 Aug 14;8:12122. doi: 10.1038/s41598-018-30466-x (PMC6092430; doi:10.1038/s41598-018-30466-x)
Supplement: Supplementary file 1 — Supplementary Dataset 1 [file 41598_2018_30466_MOESM1_ESM.pdf]

A cancer vaccine approach for personalized treatment of Lynch Syndrome

Snigdha Majumder*[1], Rakshit Shah*[2], Jisha Elias[1,2], Malini Manoharan[1], Priyanka Shah[1,] Anjali Kumari1, Papia Chakraborty[3], Vasumathi Kode[3], Yogesh Mistry[2], K. Coral [1], Bharti Mittal[1], Sakthivel Murugan SM[1], Lakshmi Mahadevan[1], Ravi Gupta[1], Amitabha Chaudhuri[1,3 **] and Arati Khanna-Gupta[1**]

[1] MedGenome Labs Pvt. Ltd., Bangalore, India. [2] KCHRC, Muni Seva Ashram, Goraj, Gujarat, India

[3]MedGenome Inc., Foster City, CA, USA

* Co-first authors

[**]Corresponding authors

List of supplementary Tables

Suppl. Table 1	Clinical features of family 1
Suppl. Table 2	Clinical features of family 2
Suppl. Table 3	Germline mutations found in MMR genes in the blood samples collected from Family 2 members
Suppl. Table 4	HLA typing of members of Family 1 and 2.
Suppl. Table 5	Analysis of peptides derived from the germline mutation in the MLH1 gene (p.E53RfsX4)
Suppl. Table 6	MLH1 p.E53RfsX4 derived peptide binding prediction with HLA types of Family 2 members
Suppl. Table 7	HLA types of healthy donors tested
Suppl. Table 8	List of genes in the 20MB panel used to screen LS affected individuals by NGS.
Suppl. Table 9	List of somatic mutations found in Family 2, patient II.2, LS+ MLH1mut
Suppl. Table 10	OncopeptVAC prediction of peptides derived from somatic mutations found in Family 2, patient II.2, LS+ MLH1mut
Suppl. Table 11	Pathogenic somatic mutations found in the tumor sample of an LS+MLH1mut patient (Family 2; II.2)

**Suppl. Table 1:** Clinical features of family 1				
Sample ID	Age	Mutation	Habits	Diagnosis
I.1	NA	Deceased (CRC)	NA	CRC
I.2	NA	Deceased	NA	NA
II.1	NA	Deceased (CRC)	NA	CRC
II.2	NA	Deceased	NA	NA
II.3	NA	Deceased	NA	NA
II.4	NA	Deceased (CRC)	No Habit	CRC
II.5	NA	Deceased (CRC)	NA	NA
II.6	NA	Deceased	NA	NA
III.1	NA	Deceased	NA	NA
III.2	NA	Deceased	NA	NA
III.3	NA	Deceased	NA	NA
III.4	NA	Deceased	NA	NA
IV.1	46	Present (Het)	No Habit	CRC
IV.2	65	Absent	No Habit	NA
IV.3	71	Absent	No Habit	NA
IV.4	81	Sample not available	NA	NA
IV.5	NA	Deceased	NA	NA
IV.2	65	Absent	No Habit	NA
IV.7	NA	Deceased	NA	NA
IV.8	NA	Deceased	NA	NA
V.1	57	Present (Het)	Yes (Bidi)	Adenocarcinoma (Mucinous: extracellular mucin >50%)
V.2	51	Present (Het)	No Habit	NA
V.3	54	Sample not available	NA	NA

Suppl. Table 2 Clinical features of family 2

Sample ID	Age	Mutation	Habits	Diagnosis
I.1	75	Absent	No habit	NA
II.1	60	Absent	Panmasala chewing	NA
II.2	52	Present (Het)	Smoking and tobacco chewing	Relapsed Colorectal Carcinoma
II.3	48	Absent	Miraj chewing	NA
II.4	45	Absent	No Habit	NA
II.5	42	Absent	Tobacco chewing	NA
II.6	38	Present (Het)	Tobacco chewing	Recto-sigmoidal Adenocarcinoma
II.7	41	Absent	No Habit	NA
II.8	50	Absent	No Habit	NA
III.1	30	Absent	No Habit	NA
III.2	28	Absent	Panmasala chewing	NA
III.3	21	Absent	No Habit	NA
III.4	11	Absent	No Habit	NA

Suppl. Table 3: Germline mutations found in MMR genes in the blood samples collected from Family 2 members

Status	Family 2 (See Figure 2B)	Chrom	Start	Reference base	Alt base	Gene Symbol	Variant Class	cDNA Change	Amino Acid Change	Ensembl Transcript	Cannoical Transcript	ClinVar Annotation	ExAC Overall Allele Frequency	1000G Overall Allele Frequency	Zygosity	Reference base depth	Alt base depth
Unaffected	II.1	chr7	6022525	C	A	PMS2	MISSENSE	c.2104G>T	p.Ala702Ser	ENST00000265849	Y	NA	NA	NA	Heterozygous	12	12
	II.1	chr7	6026775	T	C	PMS2	MISSENSE	c.1621A>G	p.Lys541Glu	ENST00000265849	Y	Lynch_syndrome [BENIGN]	0.851392367	0.883187	Homozygous	0	117
	II.1	chr7	6026942	G	T	PMS2	MISSENSE	c.1454C>A	p.Thr485Lys	ENST00000265849	Y	Lynch_syndrome [BENIGN]	0.080088649	0.112021	Heterozygous	56	58
	II.1	chr14	75513828	G	A	MLH3	MISSENSE	c.2531C>T	p.Pro844Leu	ENST00000355774	Y	NA	0.412639129	0.363818	Heterozygous	100	86
	II.1	chr14	75513883	T	C	MLH3	MISSENSE	c.2476A>G	p.Asn826Asp	ENST00000355774	Y	NA	0.996795717	0.990415	Homozygous	0	168
	II.1	chr19	50905074	G	A	POLD1	MISSENSE	c.356G>A	p.Arg119His	ENST00000440232	Y	NA	0.115418709	0.188299	Heterozygous	23	22
	II.1	chr1	242030151	A	G	EXO1	MISSENSE	c.1061A>G	p.His354Arg	ENST00000366548	Y	NA	0.592964245	0.636981	Heterozygous	108	90
	II.1	chr1	242042454	C	T	EXO1	MISSENSE	c.1918C>T	p.Pro640Ser	ENST00000366548	Y	NA	0.01299602	0.019369	Heterozygous	78	74
	II.1	chr1	242042545	A	G	EXO1	MISSENSE	c.2009A>G	p.Glu670Gly	ENST00000366548	Y	NA	0.657282364	0.681909	Heterozygous	80	70
	II.1	chr1	242045275	C	T	EXO1	MISSENSE	c.2167C>T	p.Arg723Cys	ENST00000366548	Y	NA	0.956367594	0.915335	Homozygous	0	169
	II.1	chr12	133220526	T	C	POLE	MISSENSE	c.4187A>G	p.Asn1396Ser	ENST00000320574	Y	NA	0.118171481	0.120607	Heterozygous	82	70
	II.1	chr12	133253995	G	A	POLE	MISSENSE	c.755C>T	p.Ala252Val	ENST00000320574	Y	NA	0.085681099	0.0569089	Heterozygous	111	90
	II.1	chr2	47601106	T	C	EPCAM	MISSENSE	c.344T>C	p.Met115Thr	ENST00000263735	Y	Others	0.519785744	0.666134	Homozygous	0	134
Affected	II.6	chr7	6026775	T	C	PMS2	MISSENSE	c.1621A>G	p.Lys541Glu	ENST00000265849	Y	Lynch_syndrome [BENIGN]	0.851392367	0.883187	Homozygous	0	142
	II.6	chr7	6026942	G	T	PMS2	MISSENSE	c.1454C>A	p.Thr485Lys	ENST00000265849	Y	Lynch_syndrome [BENIGN]	0.080088649	0.112021	Heterozygous	74	77
	II.6	chr7	6026988	G	A	PMS2	MISSENSE	c.1408C>T	p.Pro470Ser	ENST00000265849	Y	Lynch_syndrome [BENIGN]	0.385392981	0.358227	Heterozygous	88	70
	II.6	chr14	75513828	G	A	MLH3	MISSENSE	c.2531C>T	p.Pro844Leu	ENST00000355774	Y	NA	0.412639129	0.363818	Heterozygous	117	81
	II.6	chr14	75513883	T	C	MLH3	MISSENSE	c.2476A>G	p.Asn826Asp	ENST00000355774	Y	NA	0.996795717	0.990415	Homozygous	0	170
	II.6	chr1	242030151	A	G	EXO1	MISSENSE	c.1061A>G	p.His354Arg	ENST00000366548	Y	NA	0.592964245	0.636981	Heterozygous	103	79
	II.6	chr1	242042454	C	T	EXO1	MISSENSE	c.1918C>T	p.Pro640Ser	ENST00000366548	Y	NA	0.01299602	0.019369	Heterozygous	124	95
	II.6	chr1	242042545	A	G	EXO1	MISSENSE	c.2009A>G	p.Glu670Gly	ENST00000366548	Y	NA	0.657282364	0.681909	Heterozygous	87	94
	II.6	chr1	242045275	C	T	EXO1	MISSENSE	c.2167C>T	p.Arg723Cys	ENST00000366548	Y	NA	0.956367594	0.915335	Homozygous	0	192
	II.6	chr3	37038146	TA	T	MLH1	FRAMESHIFT-DEL	c.154delA	p.Glu53ArgfsTer4	ENST00000231790	Y	Lynch_syndrome [PATHOGENIC]	NA	NA	Heterozygous	91	86
	II.6	chr12	133220526	T	C	POLE	MISSENSE	c.4187A>G	p.Asn1396Ser	ENST00000320574	Y	NA	0.118171481	0.120607	Heterozygous	64	76
	II.6	chr2	47601106	T	C	EPCAM	MISSENSE	c.344T>C	p.Met115Thr	ENST00000263735	Y	Others	0.519785744	0.666134	Homozygous	1	130
Unaffected	III.1	chr7	6026499	G	A	PMS2	MISSENSE	c.1897C>T	p.His633Tyr	ENST00000265849	Y	NA	NA	NA	Heterozygous	122	114
	III.1	chr7	6026775	T	C	PMS2	MISSENSE	c.1621A>G	p.Lys541Glu	ENST00000265849	Y	Lynch_syndrome [BENIGN]	0.851392367	0.883187	Homozygous	1	112
	III.1	chr7	6026988	G	A	PMS2	MISSENSE	c.1408C>T	p.Pro470Ser	ENST00000265849	Y	Lynch_syndrome [BENIGN]	0.385392981	0.358227	Homozygous	0	128
	III.1	chr14	75513463	A	G	MLH3	MISSENSE	c.2896T>C	p.Ser966Pro	ENST00000355774	Y	Lynch_syndrome [BENIGN]	0.015881781	0.0111821	Heterozygous	98	71
	III.1	chr14	75513828	G	A	MLH3	MISSENSE	c.2531C>T	p.Pro844Leu	ENST00000355774	Y	NA	0.412639129	0.363818	Heterozygous	75	85
	III.1	chr14	75513883	T	C	MLH3	MISSENSE	c.2476A>G	p.Asn826Asp	ENST00000355774	Y	NA	0.996795717	0.990415	Homozygous	0	147
	III.1	chr1	242030151	A	G	EXO1	MISSENSE	c.1061A>G	p.His354Arg	ENST00000366548	Y	NA	0.592964245	0.636981	Homozygous	1	187
	III.1	chr1	242042206	G	A	EXO1	MISSENSE	c.1670G>A	p.Arg557His	ENST00000366548	Y	NA	0.00118665	0.0011981	Heterozygous	94	81
	III.1	chr1	242042301	G	A	EXO1	MISSENSE	c.1765G>A	p.Glu589Lys	ENST00000366548	Y	NA	0.36100173	0.351637	Homozygous	2	220
	III.1	chr1	242045275	C	T	EXO1	MISSENSE	c.2167C>T	p.Arg723Cys	ENST00000366548	Y	NA	0.956367594	0.915335	Homozygous	0	176
	III.1	chr3	37053568	A	G	MLH1	MISSENSE	c.655A>G	p.Ile219Val	ENST00000231790	Y	Lynch_syndrome [BENIGN]	0.23253819	0.129593	Heterozygous	61	69
	III.1	chr12	133253995	G	A	POLE	MISSENSE	c.755C>T	p.Ala252Val	ENST00000320574	Y	NA	0.085681099	0.0569089	Heterozygous	105	77
	III.1	chr2	47601106	T	C	EPCAM	MISSENSE	c.344T>C	p.Met115Thr	ENST00000263735	Y	Others	0.519785744	0.666134	Homozygous	1	118
Affected	II.2	chr7	6013049	C	G	PMS2	MISSENSE	c.2570G>C	p.Gly857Ala	ENST00000265849	Y	Lynch_syndrome [BENIGN]	0.370207538	NA	Heterozygous	19	5
	II.2	chr7	6026775	T	C	PMS2	MISSENSE	c.1621A>G	p.Lys541Glu	ENST00000265849	Y	Lynch_syndrome [BENIGN]	0.851392367	0.883187	Homozygous	0	178
	II.2	chr7	6026988	G	A	PMS2	MISSENSE	c.1408C>T	p.Pro470Ser	ENST00000265849	Y	Lynch_syndrome [BENIGN]	0.385392981	0.358227	Homozygous	1	192
	II.2	chr14	75513828	G	A	MLH3	MISSENSE	c.2531C>T	p.Pro844Leu	ENST00000355774	Y	NA	0.412639129	0.363818	Heterozygous	111	100
	II.2	chr14	75513883	T	C	MLH3	MISSENSE	c.2476A>G	p.Asn826Asp	ENST00000355774	Y	NA	0.996795717	0.990415	Homozygous	0	199
	II.2	chr19	50905074	G	A	POLD1	MISSENSE	c.356G>A	p.Arg119His	ENST00000440232	Y	NA	0.115418709	0.188299	Heterozygous	18	24
	II.2	chr1	242030151	A	G	EXO1	MISSENSE	c.1061A>G	p.His354Arg	ENST00000366548	Y	NA	0.592964245	0.636981	Homozygous	0	198
	II.2	chr1	242042301	G	A	EXO1	MISSENSE	c.1765G>A	p.Glu589Lys	ENST00000366548	Y	NA	0.36100173	0.351637	Heterozygous	194	156
	II.2	chr1	242042545	A	G	EXO1	MISSENSE	c.2009A>G	p.Glu670Gly	ENST00000366548	Y	NA	0.657282364	0.681909	Heterozygous	87	100
	II.2	chr1	242045275	C	T	EXO1	MISSENSE	c.2167C>T	p.Arg723Cys	ENST00000366548	Y	NA	0.956367594	0.915335	Homozygous	0	245
	II.2	chr3	37038146	TA	T	MLH1	FRAMESHIFT-DEL	c.154delA	p.Glu53ArgfsTer4	ENST00000231790	Y	Lynch_syndrome [PATHOGENIC]	NA	NA	Heterozygous	118	75
	II.2	chr3	37053568	A	G	MLH1	MISSENSE	c.655A>G	p.Ile219Val	ENST00000231790	Y	Lynch_syndrome [BENIGN]	0.23253819	0.129593	Heterozygous	107	92
	II.2	chr12	133220526	T	C	POLE	MISSENSE	c.4187A>G	p.Asn1396Ser	ENST00000320574	Y	NA	0.118171481	0.120607	Heterozygous	95	77
	II.2	chr12	133253995	G	A	POLE	MISSENSE	c.755C>T	p.Ala252Val	ENST00000320574	Y	NA	0.085681099	0.0569089	Heterozygous	144	121
	II.2	chr2	47601106	T	C	EPCAM	MISSENSE	c.344T>C	p.Met115Thr	ENST00000263735	Y	Others	0.519785744	0.666134	Homozygous	1	158

Note: No mutations were found in the following MMR genes: MSH2, MSH6, PMS1, PCNA

Suppl. Table 4 HLA typing of members of Family 1 and 2.

Family 1; IV.1, LS+ MLH1mut		
HLA-A	A*31:01:02:01	A*68:01:02:01
HLA-B	B*35:03:01	B*35:03:01
HLA-C	C*04:01:01:01	C*04:01:01:01
DRB1	DRB1*07:01:01:01	DRB1*15:06:01
DQB1	DQB1*02:02:01:01	DQB1*05:02:01
Family 1; IV.3, LS- MLH1wt		
HLA-A	A*01:01:01:01	A*33:03:01
HLA-B	B*15:02:01	B*44:03:02
HLA-C	C*06:02:01:01	C*07:06
DRB1	DRB1*07:01:01:01	DRB1*15:02:01:01
DQB1	DQB1*02:02:01:01	DQB1*06:01:01
Family 1; V.1, LS+ MLH1mut		
HLA-A	A*02:11:01	A*68:01:01:02
HLA-B	B*47:01:01:03	B*52:01:01:01
HLA-C	C*06:02:01:01	C*12:02:02
DRB1	DRB1*04:04:01	DRB1*15:06:01
DQB1	DQB1*03:02:01	DQB1*05:02:01
Family 1; V.2, LS- MLH1mut		
HLA-A	A*03:01:01:01	A*33:03:01
HLA-B	B*15:01:02	B*44:03:02
HLA-C	C*03:03:01	C*07:06
DRB1	DRB1*07:01:01:01	DRB1*14:04:01
DQB1	DQB1*02:02:01:01	DQB1*05:03:01:01
Family 2; II.1, LS- MLH1wt		
HLA-A*	A*03:01:01:01	A*24:17
HLA-B*	B*15:02:01	B*27:05:02
HLA-C*	C*02:02:02:01	C*08:01:01
DRB1*	DRB1*03:01:01:01	DRB1*04:04:01
DQB1*	DQB1*03:02:01:01	DQB1*03:02:01:01
Family 2; II.2, LS+ MLH1mut		
HLA-A*	A*01:01:01:01	A*02:06:01:01
HLA-B*	B*15:02:01	B*35:03:01:01
HLA-C*	C*04:01:01:01	C*08:01:01
DRB1*	DRB1*04:04:01	DRB1*14:04:01
DQB1*	DQB1*03:02:01:01	DQB1*05:03:01:01
Family 2; II.6, LS+ MLH1mut		
HLA-A*	A*01:01:01:01	A*02:06:01:01
HLA-B*	B*15:02:01	B*35:03:01:01
HLA-C*	C*04:01:01:01	C*08:01:01
DRB1*	DRB1*04:04:01	DRB1*14:04:01
DQB1*	DQB1*03:02:01:01	DQB1*05:03:01:01
Family 2; III.1, LS- MLH1wt		
HLA-A*	A*01:01:01:01	A*02:06:01:01
HLA-B*	B*35:03:01:01	B*37:01:01
HLA-C*	C*04:01:01:01	C*06:02:01:01
DRB1*	DRB1*12:02:01	DRB1*14:04:01
DQB1*	DQB1*03:01:01:02	DQB1*05:03:01:01

Suppl. Table 5 Analysis of peptides derived from the germline mutation in the MLH1 gene (p.E53RfsX4)

Wild type protein ...SIQVIVKEGGLKL...
Mutant protein ...SIQVIVKREA **Stop**

predicted neoepitopes
Seq1 TSIQVIVKR
Seq2 SIQVIVKRE
Seq3 IQVIVKREA

HLA	Peptide- Wildtype	Affinity Wildtype (nM)	Peptide-MUT	Affinity MUT (nM)	Proteasome score	Tap score	Processing score	Total score	TCR prediction
HLA-A*68:01	TSIQVIVKE	5997.52	TSIQVIVKR	10.3	1.12	0.68	1.8	0.81	Deprioritized
HLA-A*33:03	TSIQVIVKE	27223.52	TSIQVIVKR	79.1	1.12	0.68	1.8	-0.23	Deprioritized
HLA-A*31:01	TSIQVIVKE	22573.33	TSIQVIVKR	105.42	1.12	0.68	1.8	-0.37	Deprioritized

Suppl. Table 6: MLH1 p.E53RfsX4 derived peptide binding prediction with HLA types of Family 2 members

HLA	Peptide-WT	netMHCcons_Affinity(nM)_WT	Peptide-MUT	netMHCcons_Affinity(nM)_MUT	proteasome_score	tap_score	processing_score	total_score	TCR binding prediction
HLA-A*03:01	TSIQVIVKE	29266.51	TSIQVIVKR	7527.49	1.12	0.68	1.8	-2.08	Deprioritized
HLA-A*03:01	TSIQVIVKE	29266.51	TSIQVIVKR	7527.49	1.12	0.68	1.8	-2.08	Deprioritized
HLA-A*24:17	TSIQVIVKE	36533.83	TSIQVIVKR	20083.19	1.12	0.68	1.8	-2.66	Deprioritized
HLA-A*24:17	TSIQVIVKE	36533.83	TSIQVIVKR	20083.19	1.12	0.68	1.8	-2.66	Deprioritized
HLA-A*02:06	TSIQVIVKE	24614.3	TSIQVIVKR	24882.07	1.12	0.68	1.8	-2.52	Deprioritized
HLA-A*02:06	TSIQVIVKE	24614.3	TSIQVIVKR	24882.07	1.12	0.68	1.8	-2.52	Deprioritized
HLA-A*01:01	TSIQVIVKE	28795.36	TSIQVIVKR	25982.58	1.12	0.68	1.8	-2.71	Deprioritized
HLA-A*01:01	TSIQVIVKE	28795.36	TSIQVIVKR	25982.58	1.12	0.68	1.8	-2.71	Deprioritized
HLA-B*27:05	TSIQVIVKE	30726.73	TSIQVIVKR	20258.44	1.12	0.68	1.8	-2.54	Deprioritized
HLA-B*27:05	TSIQVIVKE	30726.73	TSIQVIVKR	20258.44	1.12	0.68	1.8	-2.54	Deprioritized
HLA-B*15:02	TSIQVIVKE	35176.57	TSIQVIVKR	35367.39	1.12	0.68	1.8	-2.72	Deprioritized
HLA-B*15:02	TSIQVIVKE	35176.57	TSIQVIVKR	35367.39	1.12	0.68	1.8	-2.72	Deprioritized
HLA-B*35:03	TSIQVIVKE	44389.61	TSIQVIVKR	44389.61	1.12	0.68	1.8	-2.86	Deprioritized
HLA-B*35:03	TSIQVIVKE	44389.61	TSIQVIVKR	44389.61	1.12	0.68	1.8	-2.86	Deprioritized
HLA-B*37:01	TSIQVIVKE	39622.53	TSIQVIVKR	47111.36	1.12	0.68	1.8	-2.84	Deprioritized
HLA-B*37:01	TSIQVIVKE	39622.53	TSIQVIVKR	47111.36	1.12	0.68	1.8	-2.84	Deprioritized
HLA-C*02:02	TSIQVIVKE	13356.59	TSIQVIVKR	13575.13	1.12	0.68	1.8	-2.49	Deprioritized
HLA-C*02:02	TSIQVIVKE	13356.59	TSIQVIVKR	13575.13	1.12	0.68	1.8	-2.49	Deprioritized
HLA-C*04:01	TSIQVIVKE	23067.13	TSIQVIVKR	15795.37	1.12	0.68	1.8	-2.71	Deprioritized
HLA-C*04:01	TSIQVIVKE	23067.13	TSIQVIVKR	15795.37	1.12	0.68	1.8	-2.71	Deprioritized
HLA-C*06:02	TSIQVIVKE	37333.42	TSIQVIVKR	34423.54	1.12	0.68	1.8	-2.68	Deprioritized
HLA-C*06:02	TSIQVIVKE	37333.42	TSIQVIVKR	34423.54	1.12	0.68	1.8	-2.68	Deprioritized
HLA-C*08:01	TSIQVIVKE	38979.17	TSIQVIVKR	38843.19	1.12	0.68	1.8	-2.69	Deprioritized
HLA-C*08:01	TSIQVIVKE	38979.17	TSIQVIVKR	38843.19	1.12	0.68	1.8	-2.69	Deprioritized

Suppl. Table 7: HLA types of healthy donors tested

**Healthy donor 1**		
HLA-A	A*02:01	A*68:01
HLA-B	B*40:02	B*51:01
HLA-C	C*03:04	C*15:09
**Healthy donor 2**		
HLA-A	A*02:11	A*11:01
HLA-B	B*35:01	B*35:03
HLA-C	C*04:01	C*12:03
**Healthy donor 3**		
HLA-A	A*02:01	A*11:01
HLA-B	B*07:06	B*52:01
HLA-C	C*07:02	C*12:02

Suppl. Table 8. List of genes in the 20MB panel used to screen LS affected individuals by NGS

A2M	ATP2A2	CDAN1	CYP24A1	ETV6	GNAT2	IL2RA	LPAR6	NCAPD2	PEX12	RAD50	SLC19A1	TAAR9	UBE3A
A4GALT	ATP2A3	CDC25A	CYP26A1	EVC	GNB1L	IL2RG	LPHN2	NCF1	PEX13	RAD51	SLC19A2	TAB2	UBE3B
A4GNT	ATP2B2	CDC42	CYP26B1	EVC2	GNB2	IL3	LPIN1	NCF2	PEX14	RAD51B	SLC19A3	TAC3	UBE3C
AAAS	ATP2B3	CDC42BPB	CYP26C1	EVI5	GNB3	IL31RA	LPIN2	NCF4	PEX16	RAD51C	SLC1A1	TACO1	UBIAD1
AADAC	ATP2B4	CDC5L	CYP27A1	EVI5L	GNB4	IL36B	LPIN3	NCKAP1	PEX19	RAD51D	SLC1A2	TACR3	UBN2
AADACL2	ATP2C1	CDC6	CYP27B1	EWSR1	GNB5	IL36RN	LPL	NCKIPSD	PEX2	RAD51L3-RFFL	SLC1A3	TACSTD2	UBQLN1
AAGAB	ATP5A1	CDC73	CYP2A13	EXD3	GNE	IL4	LPP	NCOA1	PEX26	RAD52	SLC1A5	TAF1	UBQLN2
AANAT	ATP5E	CDCA7	CYP2A6	EXO1	GNG7	IL4I1	LRAT	NCOA2	PEX3	RAD54B	SLC20A2	TAF13	UBR1
AARS	ATP5SL	CDCA7L	CYP2B6	EXO5	GNMT	IL4R	LRBA	NCOA3	PEX5	RAD54L	SLC22A1	TAF15	UBR3
AARS2	ATP6AP2	CDH1	CYP2C18	EXOC4	GNPAT	IL5	LRCH1	NCOA4	PEX6	RAD54L2	SLC22A11	TAF1C	UBR4
AASS	ATP6V0A1	CDH11	CYP2C19	EXOC8	GNPTAB	IL6	LRFN5	NCOA6	PEX7	RAD9A	SLC22A12	TAF1L	UBR5
ABAT	ATP6V0A2	CDH12	CYP2C8	EXOSC3	GNPTG	IL6R	LRGUK	NCOR2	PFAS	RAET1L	SLC22A14	TAF2	UBR7
ABCA1	ATP6V0A4	CDH13	CYP2C9	EXOSC8	GNRH1	IL6ST	LRIG2	NCR3	PFKFB1	RAF1	SLC22A18	TAF4B	UCHL1
ABCA10	ATP6V0C	CDH15	CYP2D6	EXPH5	GNRHR	IL7	LRIT3	NCS1	PFKM	RAG1	SLC22A2	TAF6	UCN
ABCA12	ATP6V1B1	CDH23	CYP2E1	EXT1	GNS	IL7R	LRP1	NCSTN	PFN1	RAG2	SLC22A23	TAF7L	UCP1
ABCA13	ATP6V1B2	CDH3	CYP2F1	EXT2	GOLGA3	IL8	LRP1B	NDE1	PGAM1	RAI1	SLC22A25	TAF9	UCP2
ABCA2	ATP6V1H	CDH5	CYP2J2	EXTL1	GOLGA5	IL9	LRP2	NDN	PGAM2	RALGAPA1	SLC22A3	TAL1	UCP3
ABCA3	ATP7A	CDH7	CYP2R1	EXTL2	GON4L	IL9R	LRP4	NDNL2	PGAM5	RALGDS	SLC22A4	TAL2	UFD1L
ABCA4	ATP7B	CDH8	CYP2U1	EXTL3	GOPC	ILDR1	LRP5	NDOR1	PGAP1	RANBP2	SLC22A5	TALDO1	UFL1
ABCA5	ATP8A2	CDHR1	CYP2W1	EYA1	GORAB	ILK	LRP6	NDP	PGAP2	RANBP6	SLC22A6	TANC1	UFSP2
ABCA7	ATP8B1	CDIP1	CYP3A4	EYA4	GOSR2	ILVBL	LRP8	NDRG1	PGAP3	RANGAP1	SLC22A9	TANC2	UGCG
ABCA8	ATPAF2	CDK11A	CYP3A43	EYS	GOT1	IMMP2L	LRPAP1	NDRG4	PGBD1	RANGRF	SLC23A1	TAP1	UGDH
ABCB1	ATR	CDK12	CYP3A5	EZH2	GP1BA	IMMT	LRPPRC	NDST1	PGBD3	RAP1GDS1	SLC24A1	TAP2	UGGT1
ABCB11	ATRIP	CDK16	CYP3A7	F10	GP1BB	IMPA2	LRRC2	NDST2	PGBD4	RAPGEF1	SLC24A2	TAPBP	UGGT2
ABCB4	ATRN	CDK2	CYP46A1	F11	GP2	IMPAD1	LRRC29	NDST3	PGC	RAPGEF4	SLC24A4	TARDBP	UGT1A1
ABCB5	ATRNL1	CDK4	CYP4A11	F12	GP6	IMPDH1	LRRC4	NDST4	PGD	RAPSN	SLC24A5	TARS2	UGT1A10
ABCB6	ATRX	CDK5	CYP4A22	F13A1	GP9	IMPDH2	LRRC41	NDUFA1	PGK1	RARA	SLC25A1	TAS1R1	UGT1A3
ABCB7	ATXN1	CDK5R1	CYP4B1	F13B	GPAM	IMPG1	LRRC46	NDUFA10	PGM1	RARB	SLC25A12	TAS1R2	UGT1A4
ABCC1	ATXN10	CDK5RAP2	CYP4F12	F2	GPANK1	IMPG2	LRRC6	NDUFA11	PGM3	RARS	SLC25A13	TAS1R3	UGT1A5
ABCC11	ATXN2	CDK5RAP3	CYP4F2	F2R	GPATCH8	INF2	LRRC69	NDUFA12	PGR	RARS2	SLC25A15	TAS2R16	UGT1A6
ABCC12	ATXN3	CDK6	CYP4F22	F2RL1	GPBAR1	ING1	LRRC8A	NDUFA13	PGRMC1	RASA1	SLC25A19	TAS2R19	UGT1A7
ABCC2	ATXN3L	CDK7	CYP4F3	F3	GPC3	ING3	LRRFIP2	NDUFA2	PGS1	RASA2	SLC25A20	TAS2R3	UGT1A8
ABCC3	ATXN7	CDKAL1	CYP4V2	F5	GPC4	INHA	LRRIQ1	NDUFA4	PHACTR1	RASAL1	SLC25A22	TAS2R38	UGT2A1
ABCC4	AUH	CDKL3	CYP51A1	F7	GPC6	INHBA	LRRK1	NDUFA6	PHB	RASGEF1B	SLC25A3	TAS2R43	UGT2A3
ABCC6	AUP1	CDKL5	CYP7A1	F8	GPD1	INHBB	LRRK2	NDUFA7	PHB2	RASGRP1	SLC25A38	TAS2R46	UGT2B10
ABCC8	AURKA	CDKN1A	CYP7B1	F9	GPD1L	INMT	LRRTM1	NDUFA8	PHC1	RASGRP2	SLC25A39	TAS2R50	UGT2B15
ABCC9	AURKB	CDKN1B	CYR61	FA2H	GPD2	INO80	LRRTM4	NDUFA9	PHC2	RASSF1	SLC25A4	TAS2R9	UGT2B17
ABCD1	AURKC	CDKN1C	CYS1	FAAH	GPHN	INO80D	LRSAM1	NDUFAF1	PHEX	RASSF5	SLC25A40	TAT	UGT2B28
ABCD3	AUTS2	CDKN2A	CYSLTR1	FAAH2	GPI	INPP4A	LRTOMT	NDUFAF2	PHF11	RAX	SLC25A43	TAZ	UGT2B4
ABCD4	AVP	CDKN2B	CYSLTR2	FABP1	GPIHBP1	INPP5B	LSM3	NDUFAF3	PHF19	RAX2	SLC25A46	TBC1D1	UGT2B7
ABCG1	AVPR1A	CDKN2C	D2HGDH	FABP2	GPNMB	INPP5D	LSS	NDUFAF4	PHF2	RB1	SLC26A1	TBC1D10B	UGT8
ABCG2	AVPR1B	CDKN2D	DAB2	FABP3	GPR1	INPP5E	LTA	NDUFAF5	PHF20	RB1CC1	SLC26A10	TBC1D20	UHRF1BP1
ABCG5	AVPR2	CDKN3	DACH1	FABP4	GPR101	INPP5K	LTBP1	NDUFAF6	PHF3	RBBP5	SLC26A2	TBC1D23	UIMC1
ABCG8	AXIN1	CDON	DACH2	FABP6	GPR112	INPPL1	LTBP2	NDUFAF7	PHF6	RBBP8	SLC26A3	TBC1D24	ULK4
ABHD12	AXIN2	CDSN	DACT1	FABP7	GPR12	INS	LTBP3	NDUFB1	PHF7	RBCK1	SLC26A4	TBC1D30	UMOD
ABHD14A-ACY1	AXL	CDT1	DAD1	FADD	GPR125	INSIG1	LTBP4	NDUFB11	PHF8	RBFOX1	SLC26A5	TBC1D32	UMPS
ABHD5	B2M	CDX1	DAG1	FADS2	GPR126	INSIG2	LTBR	NDUFB3	PHGDH	RBFOX3	SLC26A6	TBC1D4	UNC119
ABI3BP	B3GALNT1	CDX2	DAGLB	FAH	GPR132	INS-IGF2	LTC4S	NDUFB6	PHIP	RBL1	SLC26A8	TBC1D7	UNC13A
ABL1	B3GALNT2	CDY1	DALRD3	FAM111A	GPR139	INSL3	LTF	NDUFB9	PHKA1	RBL2	SLC26A9	TBC1D9	UNC13B
ABL2	B3GALT1	CDY2A	DAO	FAM111B	GPR143	INSL6	LTK	NDUFC2	PHKA2	RBM10	SLC27A1	TBCD	UNC13C
ABLIM1	B3GALT2	CDYL	DAOA	FAM120A	GPR152	INSR	LTN1	NDUFS1	PHKB	RBM15	SLC27A4	TBCE	UNC13D
ABO	B3GALT5	CEACAM16	DAPK1	FAM120AOS	GPR161	INSRR	LUC7L	NDUFS2	PHKG2	RBM20	SLC27A5	TBCK	UNC45B
ACACA	B3GALT6	CEBPA	DAPK3	FAM120B	GPR174	INTS1	LUM	NDUFS3	PHLDA2	RBM28	SLC28A1	TBK1	UNC5C
ACACB	B3GALTL	CEBPE	DARC	FAM126A	GPR179	INTS5	LUZP1	NDUFS4	PHLDB1	RBM43	SLC28A2	TBL1X	UNC5CL
ACAD10	B3GAT1	CECR1	DARS	FAM134B	GPR33	INTS6	LUZP4	NDUFS5	PHLPP2	RBM8A	SLC28A3	TBL1XR1	UNC79
ACAD11	B3GAT2	CECR2	DARS2	FAM134C	GPR37	INVS	LY6G6F	NDUFS6	PHOX2A	RBMXL2	SLC29A1	TBL1Y	UNC80
ACAD8	B3GAT3	CEL	DAXX	FAM136A	GPR55	IQCB1	LY96	NDUFS7	PHOX2B	RBMY1A1	SLC29A2	TBP	UNC93A
ACAD9	B3GNT1	CELA1	DAZ1	FAM160B1	GPR56	IQCE	LYN	NDUFS8	PHYH	RBP1	SLC29A3	TBR1	UNC93B1
ACADL	B3GNT2	CELF6	DAZ2	FAM160B2	GPR68	IQCG	LYPLAL1	NDUFV1	PHYKPL	RBP3	SLC29A4	TBX1	UNG
ACADM	B3GNT3	CELSR1	DAZ3	FAM161A	GPR75	IQGAP1	LYRM4	NDUFV2	PI15	RBP4	SLC2A1	TBX10	UNKL
ACADS	B3GNT4	CELSR2	DAZ4	FAM166A	GPR98	IQGAP2	LYRM7	NDUFV3	PI3	RBPJ	SLC2A10	TBX15	UPB1
ACADSB	B3GNT5	CELSR3	DAZL	FAM175A	GPRIN1	IQGAP3	LYST	NEB	PI4KA	RC3H1	SLC2A2	TBX18	UPF2
ACADVL	B3GNT6	CENPBD1	DBF4B	FAM177A1	GPRIN3	IQSEC2	LYZ	NEBL	PIAS1	RCAN1	SLC2A3	TBX19	UPF3B
ACAN	B3GNT7	CENPE	DBH	FAM188B	GPS1	IQSEC3	LZTFL1	NECAP1	PICALM	RCBTB1	SLC2A4	TBX2	UPK3A
ACAT1	B3GNT8	CENPF	DBI	FAM189A2	GPSM2	IRAK1	LZTR1	NEDD4	PICK1	RCHY1	SLC2A9	TBX20	UQCC2
ACAT2	B4GALNT1	CENPJ	DBT	FAM205A	GPT	IRAK3	LZTS1	NEDD4L	PIEZO1	RD3	SLC30A10	TBX21	UQCC3
ACBD5	B4GALNT2	CEP120	DCAF11	FAM20A	GPT2	IRAK4	MAB21L2	NEDD9	PIEZO2	RDH11	SLC30A2	TBX22	UQCRB
ACBD6	B4GALNT3	CEP135	DCAF13	FAM20C	GPX1	IRF1	MACC1	NEFH	PIF1	RDH12	SLC30A3	TBX3	UQCRC2
ACCS	B4GALNT4	CEP152	DCAF17	FAM46C	GPX4	IRF2	MACF1	NEFL	PIGA	RDH5	SLC30A5	TBX4	UQCRFS1
ACCSL	B4GALT1	CEP164	DCAF4	FAM47B	GRB10	IRF3	MACROD2	NEFM	PIGF	RDH8	SLC30A8	TBX5	UQCRQ
ACD	B4GALT2	CEP170B	DCAF8	FAM58A	GRB2	IRF4	MAD1L1	NEGR1	PIGL	RDX	SLC31A1	TBX6	URB1
ACE	B4GALT3	CEP19	DCAKD	FAM63B	GREM1	IRF5	MAD2L1	NEIL1	PIGM	RECQL4	SLC33A1	TBXA2R	UROC1
ACHE	B4GALT4	CEP250	DCBLD1	FAM65B	GRHL1	IRF6	MADD	NEIL2	PIGN	REEP1	SLC34A1	TBXAS1	UROD
ACKR1	B4GALT5	CEP290	DCC	FAM71A	GRHL2	IRF7	MAEA	NEK1	PIGO	REEP2	SLC34A2	TCAP	UROS
ACLY	B4GALT6	CEP41	DCDC1	FAM71C	GRHL3	IRF8	MAF	NEK2	PIGQ	REL	SLC34A3	TCEB3	USB1
ACMSD	B4GALT7	CEP57	DCDC2	FAM81B	GRHPR	IRF9	MAFA	NEK8	PIGR	RELN	SLC35A1	TCF12	USF1
ACO1	B9D1	CEP63	DCHS1	FAM83C	GRIA1	IRGM	MAFB	NELFA	PIGS	REN	SLC35A2	TCF20	USH1C
ACO2	B9D2	CEP68	DCHS2	FAM83H	GRIA3	IRS1	MAG	NELL1	PIGT	REPS2	SLC35A3	TCF21	USH1G
ACOX1	BAALC	CEP85L	DCK	FAM86B2	GRID1	IRS2	MAGEC3	NEU1	PIGV	RERE	SLC35B2	TCF3	USH2A
ACP1	BAAT	CER1	DCLK1	FAM8A1	GRID2	IRS4	MAGEE2	NEU2	PIGW	REST	SLC35C1	TCF4	USP1
ACP2	BACE1	CERK	DCLRE1A	FAM91A1	GRIK1	IRX4	MAGEL2	NEUROD1	PIGZ	RET	SLC35D1	TCF7	USP10
ACP5	BAG3	CERKL	DCLRE1C	FAM9C	GRIK2	IRX5	MAGI2	NEUROG3	PIK3AP1	RETN	SLC35D3	TCF7L1	USP15
ACSF3	BAG6	CERS1	DCN	FAN1	GRIK3	ISCA2	MAGT1	NEXN	PIK3C2B	REV3L	SLC35F3	TCF7L2	USP24
ACSL4	BAI3	CERS3	DCP1B	FANCA	GRIK4	ISCU	MAK	NF1	PIK3C2G	RFC2	SLC35G2	TCIRG1	USP26
ACSL5	BAIAP2	CERS6	DCPS	FANCB	GRIN1	ISG15	MAK16	NF2	PIK3C3	RFT1	SLC36A2	TCN1	USP27X
ACSL6	BANF1	CES1	DCST1	FANCC	GRIN2A	ISL1	MAL	NFASC	PIK3CA	RFWD2	SLC37A3	TCN2	USP3
ACSM2B	BANK1	CES2	DCTD	FANCD2	GRIN2B	ISPD	MALT1	NFAT5	PIK3CB	RFX2	SLC37A4	TCOF1	USP30
ACSM3	BAP1	CES4A	DCTN1	FANCD2OS	GRIN3A	ISYNA1	MAMDC4	NFATC1	PIK3CD	RFX5	SLC38A8	TCP1	USP34
ACTA1	BARD1	CETP	DCX	FANCE	GRIP1	ITCH	MAML2	NFATC2	PIK3CG	RFX6	SLC39A12	TCP11L2	USP44
ACTA2	BARX1	CFB	DCXR	FANCF	GRK1	ITGA1	MAMLD1	NFATC3	PIK3R1	RFX8	SLC39A13	TCTE1	USP46
ACTB	BARX2	CFC1	DDAH1	FANCG	GRK4	ITGA11	MAN1A2	NFATC4	PIK3R2	RFXANK	SLC39A4	TCTE3	USP54
ACTC1	BAX	CFD	DDAH2	FANCI	GRK5	ITGA2	MAN1B1	NFE2L1	PIK3R3	RFXAP	SLC39A5	TCTN1	USP6NL
ACTG1	BAZ1B	CFH	DDB2	FANCL	GRM1	ITGA2B	MAN2A1	NFE2L2	PIK3R4	RGL1	SLC3A1	TCTN2	USP7
ACTG2	BBIP1	CFHR1	DDC	FANCM	GRM3	ITGA3	MAN2B1	NFIA	PIK3R5	RGL2	SLC40A1	TCTN3	USP8
ACTL7B	BBS1	CFHR2	DDHD1	FAR1	GRM5	ITGA4	MANBA	NFIB	PIKFYVE	RGMA	SLC41A1	TDGF1	USP9X
ACTN1	BBS10	CFHR3	DDHD2	FARS2	GRM6	ITGA5	MANSC1	NFIX	PIM1	RGR	SLC44A2	TDO2	USP9Y

ACTN2	BBS12	CFHR4	DDIT3	FARSA	GRM7	ITGA6	MAOA	NFKB1	PIN1	RGS12	SLC45A2	TDP1	UST
ACTN3	BBS2	CFHR5	DDOST	FAS	GRM8	ITGA7	MAOB	NFKB2	PINK1	RGS2	SLC46A1	TDP2	UTF1
ACTN4	BBS4	CFI	DDR1	FASLG	GRN	ITGA8	MAP2	NFKBIA	PIP4K2A	RGS5	SLC47A1	TDRD7	UTP14C
ACTR3C	BBS5	CFL2	DDR2	FASN	GRPEL1	ITGA9	MAP2K1	NFKBIL1	PIP5K1B	RGS6	SLC47A2	TEAD1	UTRN
ACVR1	BBS7	CFLAR	DDRGK1	FASTKD2	GRPR	ITGAE	MAP2K2	NFKBIZ	PIP5K1C	RGS7	SLC4A1	TEC	UTS2
ACVR1B	BBS9	CFP	DDX11	FAT1	GRXCR1	ITGAM	MAP2K3	NFS1	PIR	RGS9	SLC4A10	TECPR2	UVSSA
ACVR1C	BCAM	CFTR	DDX20	FAT2	GRXCR2	ITGB1	MAP2K4	NFU1	PITPNA	RGS9BP	SLC4A11	TECR	VAMP1
ACVR2A	BCAP31	CGA	DDX24	FAT3	GSC	ITGB1BP1	MAP2K7	NGF	PITPNM1	RHAG	SLC4A3	TECTA	VAMP7
ACVR2B	BCAT1	CGB	DDX25	FAT4	GSDMA	ITGB2	MAP3K1	NGFR	PITPNM3	RHBDF1	SLC4A4	TEK	VANGL1
ACVRL1	BCAT2	CH25H	DDX39B	FAXDC2	GSDMB	ITGB3	MAP3K14	NGLY1	PITRM1	RHBDF2	SLC4A7	TEKT2	VANGL2
ACY1	BCCIP	CHAF1B	DDX3X	FBLIM1	GSE1	ITGB4	MAP3K15	NHEJ1	PITX1	RHCE	SLC52A1	TELO2	VAPB
ADA	BCHE	CHAMP1	DDX3Y	FBLN1	GSK3B	ITGB6	MAP3K6	NHLRC1	PITX2	RHD	SLC52A2	TENC1	VARS2
ADAM10	BCKDHA	CHAT	DDX47	FBLN2	GSN	ITIH1	MAP3K8	NHP2	PITX3	RHEB	SLC52A3	TENM1	VAV3
ADAM12	BCKDHB	CHCHD10	DDX5	FBLN5	GSPT1	ITIH3	MAP4	NHS	PIWIL3	RHO	SLC5A1	TENM2	VAX1
ADAM17	BCKDK	CHD1	DDX53	FBN1	GSPT2	ITIH4	MAP4K3	NICN1	PIWIL4	RHOA	SLC5A11	TENM3	VCAM1
ADAM19	BCL10	CHD1L	DDX58	FBN2	GSR	ITIH6	MAP4K4	NID1	PKD1	RHOB	SLC5A2	TENM4	VCAN
ADAM22	BCL11A	CHD2	DDX59	FBN3	GSS	ITK	MAP4K5	NIN	PKD1L1	RHOG	SLC5A5	TEP1	VCL
ADAM23	BCL2	CHD3	DEAF1	FBP1	GSTA1	ITM2B	MAP6	NINJ1	PKD2	RHOH	SLC5A7	TERF2IP	VCP
ADAM33	BCL2A1	CHD5	Dec-01	FBXL18	GSTA2	ITM2C	MAP7D1	NIPA1	PKHD1	RHOXF2	SLC6A1	TERT	VCX3A
ADAM7	BCL2L1	CHD6	DECR1	FBXL19	GSTA3	ITPA	MAP7D3	NIPA2	PKHD1L1	RHPN2	SLC6A11	TESK2	VCY
ADAM9	BCL2L11	CHD7	DEF6	FBXL4	GSTK1	ITPK1	MAPK1	NIPAL3	PKLR	RIF1	SLC6A12	TET1	VDAC1
ADAMTS1	BCL2L2	CHD8	DEFB1	FBXL6	GSTM1	ITPKC	MAPK10	NIPAL4	PKM	RIMS1	SLC6A13	TET2	VDR
ADAMTS10	BCL2L2-PABPN1	CHDH	DEFB124	FBXO10	GSTM3	ITPR1	MAPK3	NIPBL	PKN3	RIMS2	SLC6A14	TEX11	VEGFA
ADAMTS13	BCL3	CHEK1	DEFB126	FBXO11	GSTM4	ITPR2	MAPK8	NIPSNAP1	PKNOX1	RIMS3	SLC6A17	TEX13B	VEGFB
ADAMTS16	BCL6	CHEK2	DEFB4A	FBXO18	GSTO1	ITPR3	MAPK8IP1	NIPSNAP3A	PKP1	RIN2	SLC6A18	TEX14	VEGFC
ADAMTS17	BCL7A	CHFR	DENND5A	FBXO2	GSTO2	ITSN1	MAPK9	NISCH	PKP2	RINT1	SLC6A19	TEX15	VHL
ADAMTS18	BCL9	CHGA	DENR	FBXO31	GSTP1	ITSN2	MAPKAP1	NKAIN2	PLA2G10	RIOK2	SLC6A2	TF	VIM
ADAMTS19	BCL9L	CHGB	DEPDC5	FBXO38	GSTT1	IVD	MAPT	NKAIN3	PLA2G2A	RIPK2	SLC6A20	TFAM	VIMP
ADAMTS2	BCLAF1	CHI3L1	DES	FBXO39	GSTT2	IYD	MARS	NKX2-1	PLA2G2D	RIPK3	SLC6A3	TFAP2A	VIP
ADAMTS3	BCMO1	CHI3L2	DFNA5	FBXO45	GSTZ1	JAG1	MARS2	NKX2-2	PLA2G4A	RIPK4	SLC6A4	TFAP2B	VIPAS39
ADAMTS6	BCOR	CHIA	DFNB31	FBXO7	GTF2A1L	JAG2	MARVELD2	NKX2-3	PLA2G4C	RIPPLY2	SLC6A5	TFAP2D	VIPR2
ADAMTSL2	BCORL1	CHIC2	DFNB59	FBXW11	GTF2E1	JAGN1	MASP1	NKX2-5	PLA2G5	RIT1	SLC6A6	TFB1M	VKORC1
ADAMTSL3	BCR	CHIT1	DGAT1	FBXW4	GTF2H1	JAK1	MASP2	NKX2-6	PLA2G6	RIT2	SLC6A8	TFCP2	VLDLR
ADAMTSL4	BCS1L	CHKB	DGAT2L6	FBXW7	GTF2H5	JAK2	MAST1	NKX3-1	PLA2G7	RLBP1	SLC6A9	TFE3	VMA21
ADAR	BDKRB2	CHKB-CPT1B	DGCR14	FCAR	GTF2I	JAK3	MAST4	NKX3-2	PLA2R1	RLIM	SLC7A1	TFF1	VNN1
ADARB1	BDNF	CHL1	DGCR2	FCER1A	GTF2IRD1	JAM3	MASTL	NKX6-1	PLAG1	RMDN2	SLC7A10	TFG	VPREB1
ADAT3	BDP1	CHM	DGCR6	FCER1G	GTF2IRD2	JARID2	MAT1A	NLGN1	PLAGL1	RMI1	SLC7A11	TFPI	VPS13A
ADC	BEAN1	CHMP1A	DGCR8	FCER2	GTF3C2	JMJD1C	MAT2A	NLGN2	PLAT	RMND1	SLC7A14	TFR2	VPS13B
ADCK3	BEND2	CHMP2A	DGKB	FCGR1A	GTPBP3	JMJD7	MATN3	NLGN3	PLAU	RNASE3	SLC7A2	TFRC	VPS13C
ADCK4	BEST1	CHMP2B	DGKD	FCGR2A	GUCA1A	JMJD7-PLA2G4B	MATN4	NLGN4X	PLAUR	RNASE4	SLC7A5	TG	VPS13D
ADCY1	BEX4	CHMP4B	DGKE	FCGR2B	GUCA1B	JPH1	MATR3	NLGN4Y	PLB1	RNASEH1	SLC7A6	TGDS	VPS33B
ADCY10	BFSP1	CHN1	DGKG	FCGR3A	GUCY1A3	JPH2	MAVS	NLRC4	PLCB1	RNASEH2A	SLC7A6OS	TGFB1	VPS35
ADCY3	BFSP2	CHPF	DGKI	FCGR3B	GUCY2C	JPH3	MAX	NLRC5	PLCB4	RNASEH2B	SLC7A7	TGFB2	VPS37A
ADCY5	BGLAP	CHPF2	DGKZ	FCGRT	GUCY2D	JRK	MBD1	NLRP1	PLCD1	RNASEH2C	SLC7A8	TGFB3	VPS39
ADCY6	BGN	CHRD	DGUOK	FCN1	GUCY2F	JUN	MBD3	NLRP10	PLCD4	RNASEL	SLC7A9	TGFBI	VPS45
ADCY8	BHLHA9	CHRDL1	DHCR24	FCN2	GUSB	JUNB	MBD4	NLRP12	PLCE1	RNASET2	SLC8A1	TGFBR1	VPS4A
ADCY9	BHLHE41	CHRFAM7A	DHCR7	FCN3	GXYLT2	JUP	MBD5	NLRP14	PLCG1	RNF113A	SLC8B1	TGFBR2	VPS53
ADCYAP1	BHMT	CHRM1	DHDDS	FCRL3	GYG1	KAL1	MBD6	NLRP2	PLCG2	RNF114	SLC9A1	TGFBR3	VPS54
ADD1	BICC1	CHRM2	DHDH	FCRL6	GYG2	KALRN	MBL2	NLRP3	PLCXD3	RNF125	SLC9A3	TGFBRAP1	VRK1
ADD2	BICD1	CHRM3	DHFR	FDFT1	GYLTL1B	KANK1	MBNL1	NLRP7	PLCZ1	RNF128	SLC9A3R1	TGIF1	VSIG4
ADD3	BICD2	CHRNA1	DHH	FDX1L	GYPA	KANK2	MBOAT7	NLRX1	PLD2	RNF135	SLC9A6	TGM1	VSX1
ADH1B	BIN1	CHRNA2	DHODH	FECH	GYPB	KANSL1	MBP	NMB	PLD3	RNF139	SLC9A9	TGM2	VSX2
ADH1C	BIRC3	CHRNA3	DHTKD1	FEM1A	GYPC	KANSL2	MBTPS2	NME1	PLEC	RNF168	SLC9B1	TGM4	VTN
ADH4	BIRC5	CHRNA4	DHX16	FEM1B	GYPE	KARS	MC1R	NME5	PLEKHA6	RNF170	SLCO1A2	TGM5	VWA3B
ADH5	BIRC6	CHRNA5	DHX36	FEN1	GYS1	KAT2A	MC2R	NME7	PLEKHG4	RNF20	SLCO1B1	TGM6	VWF
ADH7	BIVM-ERCC5	CHRNA7	DHX37	FERMT1	GYS2	KAT2B	MC3R	NME8	PLEKHG5	RNF207	SLCO1B3	TH	WAC
ADIPOQ	BLK	CHRNA9	DHX38	FERMT3	GZMB	KAT6A	MC4R	NMNAT1	PLEKHH1	RNF212	SLCO1B7	THADA	WARS
ADIPOR1	BLM	CHRNB1	DIABLO	FES	H2BFWT	KAT6B	MCC	NMT2	PLEKHH2	RNF213	SLCO1C1	THAP1	WAS
ADK	BLMH	CHRNB2	DIAPH1	FEV	H3F3A	KATNAL2	MCCC1	NMU	PLEKHM1	RNF216	SLCO2A1	THBD	WASF3
ADM	BLNK	CHRNB4	DIAPH2	FEZF1	H6PD	KATNB1	MCCC2	NNT	PLG	RNF32	SLCO2B1	THBS1	WBSCR16
ADNP	BLOC1S3	CHRND	DIAPH3	FEZF2	HABP2	KBTBD13	MCEE	NOBOX	PLIN1	RNF38	SLCO5A1	THBS2	WBSCR17
ADORA1	BLOC1S5	CHRNE	DICER1	FFAR1	HACE1	KCNA1	MCF2	NOD1	PLIN4	RNF43	SLFN5	THBS4	WDFY3
ADORA2A	BLOC1S6	CHRNG	DIO1	FFAR4	HADH	KCNA2	MCF2L2	NOD2	PLK1S1	RNF6	SLIT1	THOC2	WDFY4
ADORA3	BLVRA	CHST14	DIO2	FGA	HADHA	KCNA3	MCFD2	NODAL	PLK4	RNLS	SLIT2	THOC6	WDPCP
ADRA1A	BMI1	CHST3	DIP2A	FGB	HADHB	KCNA4	MCHR1	NOG	PLN	RNPC3	SLIT3	THPO	WDR11
ADRA2A	BMP1	CHST6	DIP2B	FGD1	HAGH	KCNA5	MCIDAS	NOL3	PLOD1	ROBO1	SLITRK1	THRA	WDR13
ADRA2B	BMP10	CHST7	DIP2C	FGD3	HAL	KCNA6	MCL1	NOP10	PLOD2	ROBO2	SLITRK5	THRAP3	WDR19
ADRA2C	BMP15	CHST8	DIRAS3	FGD4	HAMP	KCNAB1	MCM3AP	NOP56	PLOD3	ROBO3	SLITRK6	THRB	WDR31
ADRB1	BMP2	CHSY1	DIRC2	FGF1	HAND1	KCNAB2	MCM4	NOS1	PLP1	ROCK1	SLK	THSD7A	WDR34
ADRB2	BMP2K	CHSY3	DIS3L2	FGF10	HAND2	KCNB1	MCM5	NOS1AP	PLP2	ROCK2	SLMAP	THSD7B	WDR35
ADRB3	BMP4	CHUK	DISC1	FGF14	HAO1	KCNB2	MCM6	NOS2	PLS3	ROGDI	SLMO2	TIA1	WDR36
ADRBK2	BMP5	CIAO1	DISP1	FGF16	HAP1	KCNC1	MCM8	NOS3	PLTP	ROM1	SLURP1	TICAM1	WDR4
ADSL	BMP7	CIB2	DKC1	FGF17	HAPLN1	KCNC3	MCM9	NOTCH1	PLXNA1	ROPN1L	SLX4	TIGD7	WDR45
ADTRP	BMPER	CIC	DKK1	FGF2	HARS	KCND2	MCOLN1	NOTCH2	PLXNA3	ROR2	SMAD1	TIMM44	WDR45B
AFF2	BMPR1A	CIDEA	DKK2	FGF20	HARS2	KCND3	MCPH1	NOTCH3	PLXNA4	RORA	SMAD2	TIMM8A	WDR48
AFF3	BMPR1B	CIDEC	DKK3	FGF22	HAS1	KCNE1	MCTP2	NOTCH4	PLXNB1	RORC	SMAD3	TIMP1	WDR5
AFF4	BMPR2	CIITA	DLAT	FGF23	HAS2	KCNE1L	MDC1	NOX3	PLXNB3	ROS1	SMAD4	TIMP2	WDR60
AFG3L2	BMS1	CILP	DLC1	FGF3	HAUS6	KCNE2	MDGA2	NPAP1	PLXND1	RP1	SMAD5	TIMP3	WDR62
AFP	BNC2	CILP2	DLD	FGF5	HAUS8	KCNE3	MDH1	NPAS2	PMAIP1	RP1L1	SMAD6	TINAG	WDR64
AGA	BOC	CIRH1A	DLEC1	FGF8	HAVCR1	KCNE4	MDK	NPAS3	PMFBP1	RP2	SMAD7	TINF2	WDR65
AGAP2	BOLA3	CISD2	DLG1	FGF9	HAX1	KCNH1	MDM1	NPAT	PML	RP9	SMAD9	TIRAP	WDR66
AGBL1	BPGM	CISH	DLG2	FGFBP1	HBA1	KCNH2	MDM2	NPC1	PMM2	RPA1	SMARCA2	TJP2	WDR72
AGBL4	BPI	CITED2	DLG3	FGFR1	HBA2	KCNH3	MDM4	NPC1L1	PMP22	RPA4	SMARCA4	TK1	WDR73
AGER	BPIFA1	CIZ1	DLG4	FGFR2	HBB	KCNH5	MDN1	NPC2	PMPCA	RPE65	SMARCAD1	TK2	WDR81
AGGF1	BPIFA3	CKAP2L	DLG5	FGFR3	HBD	KCNH6	ME2	NPEPPS	PMS1	RPGR	SMARCAL1	TKT	WDR93
AGK	BPY2	CKM	DLGAP1	FGFR4	HBE1	KCNH7	MECOM	NPFFR2	PMS2	RPGRIP1	SMARCB1	TLK1	WFS1
AGL	BRAF	CLASP1	DLGAP2	FGFRL1	HBEGF	KCNIP4	MECP2	NPHP1	PNKD	RPGRIP1L	SMARCC1	TLK2	WHSC1
AGMO	BRAP	CLCA1	DLGAP3	FGG	HBG1	KCNJ1	MED12	NPHP3	PNKP	RPH3AL	SMARCC2	TLL1	WHSC1L1
AGO1	BRAT1	CLCA2	DLL1	FH	HBG2	KCNJ10	MED12L	NPHP4	PNLIP	RPIA	SMARCE1	TLR1	WIPF1
AGO2	BRCA1	CLCF1	DLL3	FHIT	HBM	KCNJ11	MED13	NPHS1	PNMT	RPL10	SMC1A	TLR10	WISP3
AGPAT2	BRCA2	CLCN1	DLL4	FHL1	HBS1L	KCNJ12	MED13L	NPHS2	PNP	RPL11	SMC1B	TLR2	WIZ
AGPS	BRCC3	CLCN2	DLST	FHL2	HBZ	KCNJ13	MED17	NPL	PNPLA1	RPL15	SMC3	TLR3	WNK1
AGRN	BRD1	CLCN3	DLX3	FHOD3	HCAR1	KCNJ15	MED20	NPM1	PNPLA2	RPL21	SMCHD1	TLR4	WNK4
AGRP	BRD2	CLCN4	DLX5	FIBIN	HCCS	KCNJ16	MED23	NPPA	PNPLA3	RPL24	SMG1	TLR5	WNT1
AGT	BRD3	CLCN5	DLX6	FIG4	HCFC1	KCNJ2	MED25	NPPB	PNPLA6	RPL26	SMG6	TLR6	WNT10A
AGTPBP1	BRF1	CLCN6	DMBT1	FIGF	HCK	KCNJ3	MED29	NPPC	PNPLA8	RPL31	SMIM1	TLR7	WNT10B
AGTR1	BRIP1	CLCN7	DMBX1	FIGLA	HCLS1	KCNJ5	MEF2A	NPR1	PNPO	RPL35A	SMIM3	TLR8	WNT3
AGTR2	BRSK1	CLCNKA	DMC1	FILIP1	HCN1	KCNJ6	MEF2C	NPR2	PNPT1	RPL36	SMN1	TLR9	WNT3A
AGXT	BRSK2	CLCNKB	DMD	FIP1L1	HCN2	KCNJ8	MEFV	NPR3	POC1A	RPL36A-HNRNPH2	SMN2	TLX1	WNT4

AGXT2	BRWD1	CLDN1	DMGDH	FKBP10	HCN3	KCNJ9	MEGF10	NPSR1	POC1B	RPL38	SMNDC1	TLX2	WNT5A
AHCY	BRWD3	CLDN14	DMP1	FKBP14	HCN4	KCNK17	MEGF11	NPTN	PODXL	RPL5	SMO	TLX3	WNT5B
AHDC1	BSCL2	CLDN16	DMPK	FKBP1A	HCRT	KCNK18	MEGF8	NPY	POF1B	RPL6	SMOC1	TM4SF19	WNT7A
AHI1	BSG	CLDN19	DMRT1	FKBP4	HCRTR1	KCNK3	MEIS1	NPY1R	POFUT1	RPN2	SMOC2	TM4SF2	WNT8A
AHNAK2	BSN	CLDN2	DMXL1	FKBP5	HCRTR2	KCNK5	MEIS2	NPY2R	POFUT2	RPS10	SMPD1	TM4SF20	WNT9B
AHR	BSND	CLEC10A	DMXL2	FKBP6	HDAC1	KCNK6	MEN1	NQO1	POGLUT1	RPS10-NUDT3	SMPD3	TM9SF3	WRAP53
AHRR	BST1	CLEC11A	DNA2	FKBP8	HDAC2	KCNK9	MEOX1	NQO2	POGZ	RPS14	SMPX	TMC1	WRN
AHSG	BTAF1	CLEC16A	DNAAF1	FKBPL	HDAC3	KCNMA1	MEP1B	NR0B1	POLB	RPS15	SMS	TMC3	WT1
AHSP	BTBD9	CLEC2D	DNAAF2	FKRP	HDAC4	KCNMB1	MEPE	NR0B2	POLD1	RPS17	SMTNL1	TMC6	WWC1
AICDA	BTC	CLEC3A	DNAAF3	FKTN	HDAC6	KCNMB3	MERTK	NR1H2	POLE	RPS17L	SMUG1	TMC8	WWC2
AIF1	BTD	CLEC3B	DNAH1	FLAD1	HDAC8	KCNMB4	MESDC2	NR1H3	POLE2	RPS19	SMYD3	TMCO1	WWC3
AIFM1	BTK	CLEC4D	DNAH10	FLCN	HDAC9	KCNN2	MESP1	NR1H4	POLG	RPS20	SNAI2	TMEM107	WWOX
AIMP1	BTLA	CLEC4M	DNAH11	FLG	HDC	KCNN3	MESP2	NR1I2	POLG2	RPS24	SNAI3	TMEM114	WWP2
AIP	BTN1A1	CLEC7A	DNAH5	FLG2	HDGFRP2	KCNQ1	MEST	NR1I3	POLH	RPS26	SNAP25	TMEM126A	WWTR1
AIPL1	BTN2A1	CLHC1	DNAH7	FLI1	HDLBP	KCNQ2	MET	NR2E1	POLK	RPS27A	SNAP29	TMEM127	XAF1
AIRE	BTNL2	CLIC2	DNAH8	FLNA	HDX	KCNQ3	METTL14	NR2E3	POLL	RPS28	SNAPC4	TMEM132D	XBP1
AK1	BTRC	CLIC5	DNAH9	FLNB	HEATR2	KCNQ4	METTL23	NR2F1	POLR1A	RPS29	SNAPC5	TMEM132E	XDH
AK2	BUB1	CLIC6	DNAI1	FLNC	HELQ	KCNQ5	METTL2B	NR2F2	POLR1C	RPS3	SNCA	TMEM135	XG
AK7	BUB1B	CLIP2	DNAI2	FLOT1	HEPACAM	KCNS1	MFAP4	NR3C1	POLR1D	RPS4Y2	SNCAIP	TMEM138	XIAP
AK8	BUB3	CLK2	DNAJA4	FLRT1	HEPACAM2	KCNS3	MFAP5	NR3C2	POLR2A	RPS5	SNCB	TMEM139	XIRP1
AK9	BVES	CLMP	DNAJB2	FLRT3	HEPH	KCNT1	MFF	NR4A1	POLR2E	RPS6KA1	SNCG	TMEM165	XIRP2
AKAP10	BZRAP1	CLN3	DNAJB6	FLT1	HERC1	KCNU1	MFGE8	NR4A2	POLR2F	RPS6KA2	SND1	TMEM173	XK
AKAP13	C10ORF11	CLN5	DNAJC13	FLT3	HERC2	KCNV1	MFI2	NR4A3	POLR2M	RPS6KA3	SNIP1	TMEM18	XKR4
AKAP2	C10ORF2	CLN6	DNAJC19	FLT4	HES6	KCNV2	MFN2	NR5A1	POLR3A	RPS6KB1	SNRK	TMEM185A	XKR6
AKAP9	C11ORF40	CLN8	DNAJC3	FLVCR1	HES7	KCTD1	MFRP	NRAS	POLR3B	RPS6KL1	SNRNP200	TMEM187	XKR9
AKR1B1	C12ORF10	CLNK	DNAJC30	FLVCR2	HESX1	KCTD13	MFSD2A	NRCAM	POLR3H	RPS7	SNRPB	TMEM2	XKRY
AKR1C2	C12ORF4	CLNS1A	DNAJC5	FMN1	HEXA	KCTD17	MFSD6L	NRG1	POLRMT	RPSA	SNRPE	TMEM201	XPA
AKR1C3	C12ORF57	CLOCK	DNAJC6	FMN2	HEXB	KCTD6	MFSD8	NRG3	POM121C	RPSAP52	SNRPN	TMEM216	XPC
AKR1C4	C12ORF65	CLP1	DNAL1	FMO1	HEXDC	KCTD7	MGAT1	NRGN	POMC	RPTOR	SNTA1	TMEM219	XPNPEP1
AKR1D1	C14ORF105	CLPB	DNAL4	FMO2	HEY1	KDELC2	MGAT2	NRIP1	POMGNT1	RPUSD3	SNTB1	TMEM231	XPNPEP2
AKR1E2	C14ORF2	CLPP	DNASE1	FMO3	HEY2	KDM1A	MGAT3	NRL	POMGNT2	RRAS	SNTB2	TMEM237	XPNPEP3
AKR7A2	C14ORF28	CLPS	DNASE1L2	FMO4	HFE	KDM3A	MGAT4A	NRP2	POMK	RRAS2	SNTG1	TMEM240	XPO5
AKR7A3	C15ORF41	CLPTM1	DNASE1L3	FMO5	HFE2	KDM3B	MGAT4B	NRTN	POMP	RRH	SNTG2	TMEM256-PLSCR3	XPO7
AKT1	C15ORF62	CLPTM1L	DNASE2	FMO6P	HFM1	KDM4C	MGAT4C	NRXN1	POMT1	RRM1	SNURF	TMEM38B	XPOT
AKT2	C16ORF58	CLPX	DND1	FMOD	HGD	KDM5A	MGAT5	NRXN2	POMT2	RRM2B	SNX1	TMEM39A	XPR1
AKT3	C18ORF8	CLRN1	DNM1	FMR1	HGF	KDM5B	MGAT5B	NRXN3	PON1	RRP1B	SNX10	TMEM43	XRCC1
AKTIP	C19ORF12	CLSPN	DNM1L	FMR1-AS1	HGSNAT	KDM5C	MGEA5	NSD1	PON2	RRP8	SNX14	TMEM5	XRCC2
ALAD	C19ORF18	CLSTN1	DNM2	FN1	HHAT	KDM5D	MGLL	NSDHL	PON3	RS1	SNX19	TMEM67	XRCC3
ALAS1	C19ORF80	CLSTN2	DNMT1	FN3K	HHEX	KDM6A	MGME1	NSMCE2	POP1	RSC1A1	SNX22	TMEM70	XRCC4
ALAS2	C1GALT1	CLTC	DNMT3A	FN3KRP	HHIP	KDM6B	MGMT	NSMF	POR	RSPH1	SNX3	TMEM87B	XRCC5
ALB	C1GALT1C1	CLTCL1	DNMT3B	FNBP4	HIBADH	KDR	MGP	NSUN2	PORCN	RSPH3	SNX31	TMEM8A	XRCC6
ALCAM	C1ORF127	CLU	DNMT3L	FNIP1	HIBCH	KEAP1	MGST2	NSUN7	POSTN	RSPH4A	SNX5	TMEM8C	XRN2
ALDH16A1	C1ORF168	CLUL1	DOC2A	FOCAD	HID1	KEL	MGST3	NT5C1B	POT1	RSPH9	SOBP	TMEM9	XYLT1
ALDH18A1	C1QA	CLYBL	DOCK2	FOLH1	HIF1A	KERA	MIA	NT5C2	POU1F1	RSPO1	SOCS1	TMEM91	XYLT2
ALDH1A1	C1QB	CMA1	DOCK3	FOLR1	HIF1AN	KHDC3L	MIA3	NT5C3A	POU3F4	RSPO4	SOCS2	TMEM92	YAE1D1
ALDH1A2	C1QC	CMPK1	DOCK4	FOS	HIGD2A	KHK	MIB1	NT5DC1	POU4F3	RSRC1	SOCS3	TMEM98	YAP1
ALDH1A3	C1QTNF5	CMPK2	DOCK6	FOXA1	HINT1	KIAA0100	MICA	NT5E	POU5F1	RTEL1	SOD1	TMEM99	YARS
ALDH1B1	C1R	CNBP	DOCK7	FOXA2	HIP1	KIAA0196	MICAL3	NTF3	POU5F1B	RTEL1-TNFRSF6B	SOD2	TMIE	YARS2
ALDH1L2	C1S	CNDP1	DOCK8	FOXA3	HIST1H2AE	KIAA0226	MICALCL	NTF4	POU6F2	RTF1	SOD3	TMLHE	YBEY
ALDH2	C2	CNDP2	DOCK9	FOXC1	HIST1H2BE	KIAA0232	MICB	NTHL1	PPARA	RTN2	SOGA3	TMPO	YBX2
ALDH3A2	C20ORF197	CNGA1	DOK1	FOXC2	HIST1H3B	KIAA0319	MICU1	NTNG1	PPARD	RTN4R	SOHLH1	TMPRSS11A	YTHDC1
ALDH4A1	C21ORF2	CNGA3	DOK2	FOXD3	HIST1H4I	KIAA0430	MID1	NTRK1	PPARG	RTN4RL1	SOHLH2	TMPRSS15	YTHDF2
ALDH5A1	C21ORF59	CNGB1	DOK5	FOXD4	HIST2H2BF	KIAA0513	MID2	NTRK2	PPARGC1A	RTTN	SON	TMPRSS3	YWHAE
ALDH6A1	C21ORF91	CNGB3	DOK7	FOXE1	HIST3H3	KIAA0586	MIF	NTRK3	PPARGC1B	RUNDC3B	SORBS1	TMPRSS4	YWHAZ
ALDH7A1	C2CD3	CNKSR1	DOLK	FOXE3	HIVEP1	KIAA1033	MIIP	NUAK1	PPAT	RUNX1	SORBS2	TMPRSS5	YY1
ALDOA	C2CD5	CNKSR2	DOLPP1	FOXF1	HIVEP2	KIAA1109	MINPP1	NUB1	PPEF2	RUNX1T1	SORCS1	TMPRSS6	ZAN
ALDOB	C2ORF42	CNNM2	DOPEY1	FOXF2	HIVEP3	KIAA1199	MIOS	NUBPL	PPIA	RUNX2	SORL1	TMTC3	ZAP70
ALG1	C2ORF71	CNNM3	DOT1L	FOXG1	HK1	KIAA1210	MIP	NUCB2	PPIB	RUNX3	SORT1	TNC	ZBTB16
ALG10	C3	CNNM4	DPAGT1	FOXH1	HK2	KIAA1217	MIPOL1	NUDC	PPIG	RUVBL1	SOS1	TNF	ZBTB18
ALG10B	C3AR1	CNOT1	DPCD	FOXI1	HLA-A	KIAA1244	MITF	NUDT1	PPM1B	RXFP2	SOS2	TNFAIP1	ZBTB20
ALG11	C4A	CNOT3	DPEP2	FOXJ1	HLA-B	KIAA1279	MKI67	NUDT6	PPM1D	RXRA	SOST	TNFAIP2	ZBTB24
ALG12	C4B	CNOT4	DPH1	FOXK1	HLA-C	KIAA1377	MKKS	NUDT7	PPM1G	RXRG	SOX10	TNFAIP3	ZBTB33
ALG13	C4BPA	CNPY3	DPM1	FOXL1	HLA-DMB	KIAA1429	MKL1	NUMA1	PPM1K	RYK	SOX11	TNFRSF10A	ZBTB40
ALG14	C4ORF19	CNR1	DPM2	FOXL2	HLA-DOA	KIAA1432	MKL2	NUMBL	PPOX	RYR1	SOX17	TNFRSF10B	ZBTB41
ALG2	C4ORF26	CNR2	DPM3	FOXM1	HLA-DPB1	KIAA1462	MKRN3	NUP107	PPP1CB	RYR2	SOX18	TNFRSF11A	ZBTB42
ALG3	C5	CNTF	DPP10	FOXN1	HLA-DPB2	KIAA1549	MKS1	NUP155	PPP1R12B	RYR3	SOX2	TNFRSF11B	ZBTB45
ALG5	C5AR2	CNTN1	DPP3	FOXO1	HLA-DQA1	KIAA1919	MLC1	NUP210	PPP1R13L	S100A14	SOX3	TNFRSF13B	ZC3H10
ALG6	C5ORF42	CNTN2	DPP4	FOXO3	HLA-DQB1	KIAA2018	MLH1	NUP214	PPP1R15B	S100B	SOX5	TNFRSF13C	ZC3H12B
ALG8	C5ORF66	CNTN3	DPP6	FOXP1	HLA-DRA	KIAA2022	MLH3	NUP37	PPP1R17	S1PR1	SOX6	TNFRSF14	ZC3H14
ALG9	C6	CNTN4	DPY19L2	FOXP2	HLA-DRB1	KIF11	MLLT10	NUP62	PPP1R1A	S1PR3	SOX7	TNFRSF1A	ZC3H3
ALK	C6ORF15	CNTN6	DPYD	FOXP3	HLA-DRB5	KIF13B	MLLT11	NUP98	PPP1R3A	SAA1	SOX8	TNFRSF1B	ZC3H6
ALMS1	C6ORF222	CNTNAP1	DPYS	FOXRED1	HLA-E	KIF14	MLLT3	NUS1	PPP1R3C	SAA2	SOX9	TNFRSF21	ZC3HAV1
ALOX12	C7	CNTNAP2	DPYSL2	FPGS	HLA-G	KIF17	MLPH	NVL	PPP2CB	SAA4	SP1	TNFRSF25	ZC4H2
ALOX12B	C7ORF43	CNTNAP3	DRAM2	FPGT-TNNI3K	HLCS	KIF18A	MLXIPL	NXF3	PPP2R1A	SACS	SP100	TNFRSF4	ZCCHC12
ALOX15	C7ORF55	CNTNAP4	DRC1	FPR1	HLX	KIF1A	MLYCD	NXF5	PPP2R1B	SAFB2	SP110	TNFRSF6B	ZCCHC13
ALOX5	C7ORF60	CNTNAP5	DRD1	FPR2	HMBS	KIF1B	MMAA	NXNL1	PPP2R2A	SAG	SP7	TNFRSF9	ZCCHC16
ALOX5AP	C8A	CNTROB	DRD2	FRA10AC1	HMCN1	KIF1C	MMAB	NXPH3	PPP2R2B	SAGE1	SP8	TNFSF10	ZCCHC8
ALOXE3	C8B	COA3	DRD3	FRAS1	HMGA1	KIF21A	MMACHC	NYNRIN	PPP2R2C	SALL1	SPAG1	TNFSF11	ZDHHC15
ALPL	C8ORF37	COA5	DRD4	FREM1	HMGA2	KIF22	MMADHC	NYX	PPP2R5D	SALL2	SPAG16	TNFSF12	ZDHHC17
ALS2	C9	COA6	DRD5	FREM2	HMGB3	KIF23	MMD2	OARD1	PPP3CA	SALL4	SPAG17	TNFSF13B	ZDHHC2
ALS2CL	C9ORF114	COASY	DROSHA	FREM3	HMGCL	KIF24	MME	OAS1	PPP3R1	SAMD9	SPAG9	TNFSF14	ZDHHC24
ALX1	C9ORF172	COCH	DRP2	FRG1	HMGCR	KIF26B	MMEL1	OAS2	PPT1	SAMHD1	SPANXN5	TNFSF15	ZDHHC5
ALX3	C9ORF72	COG1	DSC2	FRK	HMGCS2	KIF27	MMP1	OAS3	PQBP1	SAR1B	SPARC	TNFSF18	ZDHHC8
ALX4	CA1	COG2	DSC3	FRMD5	HMHA1	KIF2A	MMP10	OAT	PRADC1	SARDH	SPAST	TNFSF4	ZDHHC9
AMACR	CA10	COG3	DSCAM	FRMD6	HMMR	KIF2B	MMP12	OAZ1	PRAF2	SARM1	SPATA13	TNFSF8	ZEB1
AMBN	CA12	COG4	DSCR8	FRMD7	HMOX1	KIF3C	MMP13	OBSCN	PRAMEF2	SARS2	SPATA16	TNK2	ZEB2
AMD1	CA2	COG5	DSE	FRMPD4	HMOX2	KIF4A	MMP14	OBSL1	PRB1	SART1	SPATA21	TNKS	ZFAT
AMELX	CA4	COG6	DSG1	FRY	HMSD	KIF5A	MMP19	OCA2	PRB3	SART3	SPATA22	TNNC1	ZFHX3
AMELY	CA5A	COG7	DSG2	FRZB	HMX1	KIF5B	MMP2	OCLN	PRB4	SASS6	SPATA31C1	TNNI2	ZFHX4
AMER1	CA6	COG8	DSG3	FSCB	HMX2	KIF5C	MMP20	OCRL	PRCC	SAT1	SPATA32	TNNI3	ZFP14
AMH	CA8	COL10A1	DSG4	FSCN2	HNF1A	KIF6	MMP3	ODC1	PRCD	SATB2	SPATA5	TNNI3K	ZFP36
AMHR2	CABIN1	COL11A1	DSP	FSD1L	HNF1B	KIF7	MMP7	OFD1	PRCP	SATL1	SPATA5L1	TNNT1	ZFP36L1
AMN	CABP2	COL11A2	DSPP	FSHB	HNF4A	KIFAP3	MMP8	OGDH	PRDM1	SBDS	SPATA6L	TNNT2	ZFP36L2
AMPD1	CABP4	COL12A1	DST	FSHR	HNMT	KIR2DL1	MMP9	OGG1	PRDM12	SBF1	SPATA7	TNNT3	ZFP57
AMPD2	CACHD1	COL14A1	DSTYK	FST	HNRNPA1	KIR2DL3	MMRN2	OGT	PRDM16	SBF2	SPDYC	TNP1	ZFP69
AMPD3	CACNA1A	COL15A1	DTHD1	FSTL1	HNRNPA2B1	KIR2DL4	MN1	OLFM2	PRDM2	SBK3	SPECC1	TNPO2	ZFP90
AMT	CACNA1B	COL17A1	DTNA	FTCD	HNRNPD	KIR3DL1	MNX1	OLIG2	PRDM5	SBNO1	SPECC1L	TNPO3	ZFPL1
AMZ1	CACNA1C	COL18A1	DTNB	FTH1	HNRNPDL	KIR3DL2	MOB4	OLR1	PRDM8	SC5D	SPECC1L-ADORA2A	TNR	ZFPM2
ANAPC1	CACNA1D	COL19A1	DTNBP1	FTHL17	HNRNPH1	KIRREL3	MOCOS	OMG	PRDM9	SCAP	SPEF2	TNRC18	ZFR

ANAPC5	CACNA1E	COL1A1	DTX1	FTL	HNRNPH3	KISS1	MOCS1	OPA1	PRDX1	SCARB1	SPEG	TNRC6B	ZFY
ANG	CACNA1F	COL1A2	DUOX2	FTMT	HNRNPK	KISS1R	MOCS2	OPA3	PRDX2	SCARB2	SPEN	TNRC6C	ZFYVE20
ANGPT1	CACNA1G	COL21A1	DUOXA1	FTO	HNRNPU	KIT	MOG	OPCML	PRDX4	SCARF2	SPERT	TNS3	ZFYVE26
ANGPTL3	CACNA1H	COL25A1	DUOXA2	FTSJ1	HNRNPUL2-BSCL2	KITLG	MOGS	OPHN1	PREPL	SCG2	SPG11	TNXB	ZFYVE27
ANGPTL4	CACNA1I	COL27A1	DUSP15	FUBP1	HOGA1	KL	MOK	OPLAH	PRF1	SCG3	SPG20	TOMM40	ZHX3
ANGPTL5	CACNA1S	COL2A1	DUSP23	FUCA1	HOMER2	KLB	MOV10	OPN1LW	PRG4	SCGB1A1	SPG21	TONSL	ZIC1
ANGPTL6	CACNA2D1	COL3A1	DUSP3	FURIN	HOMEZ	KLC1	MPC1	OPN1MW	PRICKLE1	SCGB1D2	SPG7	TOP1	ZIC2
ANK1	CACNA2D2	COL4A1	DUSP6	FUS	HORMAD1	KLF1	MPDU1	OPN1SW	PRICKLE2	SCGB3A2	SPI1	TOP1MT	ZIC3
ANK2	CACNA2D3	COL4A2	DUX4	FUT1	HOXA1	KLF10	MPDZ	OPN4	PRIMPOL	SCLT1	SPINK1	TOP2A	ZIC4
ANK3	CACNA2D4	COL4A3	DUX4L3	FUT2	HOXA10	KLF11	MPG	OPRD1	PRKAA2	SCN10A	SPINK5	TOPBP1	ZKSCAN5
ANKH	CACNB2	COL4A3BP	DVL1	FUT3	HOXA11	KLF5	MPHOSPH8	OPRK1	PRKACA	SCN11A	SPINT2	TOPORS	ZMPSTE24
ANKK1	CACNB4	COL4A4	DVL2	FUT6	HOXA13	KLF6	MPI	OPRL1	PRKACG	SCN1A	SPOCK1	TOR1A	ZMYM2
ANKRD1	CACNG1	COL4A5	DVL3	FUT7	HOXA2	KLF7	MPL	OPRM1	PRKAG2	SCN1B	SPOP	TOR1AIP1	ZMYM3
ANKRD11	CACNG2	COL4A6	DXO	FUT8	HOXA3	KLF8	MPLKIP	OPTC	PRKAG3	SCN2A	SPP1	TOX3	ZMYM5
ANKRD22	CACNG3	COL5A1	DYM	FUZ	HOXA4	KLHDC1	MPO	OPTN	PRKAR1A	SCN2B	SPP2	TP53	ZMYM6
ANKRD26	CACNG4	COL5A2	DYNAP	FXN	HOXA9	KLHDC8B	MPP3	OR10V1	PRKAR1B	SCN3A	SPR	TP53AIP1	ZMYND10
ANKRD50	CAD	COL6A1	DYNC1H1	FXYD2	HOXB1	KLHL1	MPP4	OR10X1	PRKCA	SCN3B	SPRED1	TP53BP1	ZMYND11
ANKRD6	CADM1	COL6A2	DYNC2H1	FXYD6	HOXB13	KLHL10	MPP6	OR13G1	PRKCB	SCN4A	SPRED2	TP53BP2	ZMYND15
ANKS1A	CADM2	COL6A3	DYRK1A	FYB	HOXB6	KLHL13	MPP7	OR13H1	PRKCD	SCN4B	SPRN	TP53I13	ZNF133
ANKS1B	CADPS	COL6A5	DYRK1B	FYCO1	HOXB8	KLHL15	MPST	OR1B1	PRKCG	SCN5A	SPRR3	TP53I3	ZNF141
ANKS3	CADPS2	COL7A1	DYSF	FZD1	HOXC13	KLHL20	MPV17	OR1J1	PRKCH	SCN7A	SPRTN	TP53RK	ZNF157
ANKS6	CALCA	COL8A2	DYX1C1	FZD3	HOXD10	KLHL3	MPZ	OR2J3	PRKCSH	SCN8A	SPRY1	TP63	ZNF175
ANLN	CALCR	COL9A1	DYX1C1-CCPG1	FZD4	HOXD11	KLHL40	MR1	OR2M2	PRKCZ	SCN9A	SPRY2	TP73	ZNF2
ANO10	CALCRL	COL9A2	E2F1	FZD6	HOXD13	KLHL41	MRAP	OR4M2	PRKD1	SCNN1A	SPRY3	TPCN2	ZNF202
ANO3	CALHM1	COL9A3	E2F4	FZD9	HOXD4	KLHL6	MRAP2	OR51G1	PRKD3	SCNN1B	SPRY4	TPH1	ZNF213
ANO5	CALM1	COLEC11	E2F5	G6PC	HP	KLHL7	MRC1	OR52H1	PRKDC	SCNN1D	SPSB1	TPH2	ZNF224
ANO6	CALM2	COLQ	EARS2	G6PC2	HPCA	KLHL9	MRC2	OR52N4	PRKG1	SCNN1G	SPTA1	TPI1	ZNF236
ANO7	CALM3	COMMD1	EBAG9	G6PC3	HPCAL4	KLK1	MRE11A	OR5AC2	PRKG2	SCO1	SPTAN1	TPK1	ZNF24
ANTXR1	CALR	COMP	EBF4	G6PD	HPD	KLK12	MREG	OR5H6	PRKRA	SCO2	SPTB	TPM1	ZNF276
ANTXR2	CALR3	COMT	EBP	GAA	HPGD	KLK15	MRI1	OR7D4	PRL	SCP2	SPTBN1	TPM2	ZNF292
ANXA1	CAMK2A	COPA	ECE1	GAB1	HPRT1	KLK3	MRPL12	OR8H1	PRLH	SCRIB	SPTBN2	TPM3	ZNF311
ANXA11	CAMK2B	COPS2	ECE2	GAB2	HPS1	KLK4	MRPL3	OR8K3	PRLHR	SCRN1	SPTBN5	TPMT	ZNF326
ANXA5	CAMK2D	COQ2	ECEL1	GABARAPL1	HPS3	KLK7	MRPL44	ORAI1	PRLR	SCT	SPTLC1	TPO	ZNF331
AOAH	CAMK2G	COQ4	ECHS1	GABBR1	HPS4	KLKB1	MRPL48	ORC1	PRM1	SCUBE2	SPTLC2	TPP1	ZNF335
AOC1	CAMK4	COQ5	ECI1	GABBR2	HPS5	KLLN	MRPS16	ORC4	PRM2	SDC3	SQLE	TPP2	ZNF350
AOX1	CAMKK1	COQ6	ECM1	GABRA1	HPS6	KLRC1	MRPS22	ORC6	PRMT10	SDCCAG8	SQSTM1	TPR	ZNF365
AP1S1	CAMKK2	COQ9	ECM2	GABRA2	HPSE2	KLRK1	MRRF	ORMDL3	PRMT3	SDHA	SRC	TPRN	ZNF385B
AP1S2	CAMKMT	CORIN	ECSIT	GABRA5	HR	KMO	MS4A1	OSBPL1A	PRMT7	SDHAF1	SRCAP	TPSB2	ZNF407
AP1S3	CAMP	CORO1A	ECT2L	GABRA6	HRAS	KMT2A	MS4A10	OSBPL2	PRND	SDHAF2	SRCIN1	TPTE	ZNF408
AP2S1	CAMSAP2	CORO7-PAM16	EDA	GABRB2	HRC	KMT2B	MS4A12	OSBPL3	PRNP	SDHB	SRD5A2	TRA2A	ZNF41
AP3B1	CAMTA1	COX10	EDA2R	GABRB3	HRG	KMT2C	MS4A2	OSBPL5	PROC	SDHC	SRD5A3	TRAC	ZNF419
AP3B2	CANT1	COX14	EDAR	GABRD	HRH2	KMT2D	MS4A3	OSER1	PROCR	SDHD	SREBF1	TRADD	ZNF420
AP3D1	CAPN10	COX15	EDARADD	GABRG1	HRH3	KMT2E	MS4A6A	OSGIN1	PRODH	SDR39U1	SREBF2	TRAF3	ZNF423
AP4B1	CAPN12	COX20	EDC3	GABRG2	HS1BP3	KNG1	MS4A6E	OSMR	PROK1	SEC23A	SREK1	TRAF3IP1	ZNF426
AP4B1-AS1	CAPN13	COX4I1	EDEM1	GABRG3	HS6ST1	KPNA1	MSC	OSR1	PROK2	SEC23B	SRGAP1	TRAF3IP2	ZNF433
AP4E1	CAPN15	COX4I2	EDN1	GABRR2	HSD11B1	KPNA7	MSH2	OSTM1	PROKR1	SEC24B	SRGAP2	TRAF3IP2-AS1	ZNF445
AP4M1	CAPN3	COX6A1	EDN2	GAD1	HSD11B2	KPTN	MSH3	OTC	PROKR2	SEC24D	SRGAP3	TRAF6	ZNF45
AP4S1	CAPN5	COX6B1	EDN3	GAD2	HSD17B1	KRAS	MSH4	OTOA	PROM1	SEC31A	SRI	TRAF7	ZNF451
AP5Z1	CAPN9	COX7A1	EDNRA	GADD45A	HSD17B10	KRIT1	MSH5	OTOF	PROP1	SEC63	SRL	TRAK1	ZNF469
APAF1	CAPRIN1	COX7A2	EDNRB	GADD45B	HSD17B2	KRT1	MSH6	OTOG	PROS1	SECISBP2	SRP72	TRAK2	ZNF480
APBA2	CARD11	COX7B	EDRF1	GAK	HSD17B3	KRT10	MSMB	OTOGL	PROX1	SEL1L	SRPK2	TRAP1	ZNF490
APBB1	CARD14	CP	EEA1	GAL	HSD17B4	KRT12	MSMO1	OTOR	PROX2	SELE	SRPX	TRAPPC10	ZNF507
APBB1IP	CARD8	CPA1	EEF1A2	GAL3ST1	HSD3B1	KRT13	MSR1	OTUD4	PROZ	SELL	SRPX2	TRAPPC11	ZNF513
APBB2	CARD9	CPA4	EEF1B2	GAL3ST2	HSD3B2	KRT14	MSRA	OTX2	PRPF3	SELP	SRR	TRAPPC2	ZNF518B
APBB3	CARM1	CPA6	EEF1D	GAL3ST3	HSD3B7	KRT15	MSRB3	OVCH2	PRPF31	SELPLG	SRSF7	TRAPPC9	ZNF526
APC	CARS2	CPB2	EEF2	GAL3ST4	HSF1	KRT16	MST1	OVGP1	PRPF39	SEMA3A	SRY	TRBV9	ZNF532
APC2	CARTPT	CPE	EEF2K	GALC	HSF2	KRT17	MST1R	OXCT1	PRPF4	SEMA3D	SS18L1	TRDN	ZNF536
APCDD1	CASC4	CPLX2	EFCAB13	GALE	HSF4	KRT18	MSTN	OXTR	PRPF6	SEMA3E	SSH1	TRDV1	ZNF551
APEX1	CASC5	CPN1	EFCAB5	GALK1	HSFY1	KRT2	MSX1	P2RX1	PRPF8	SEMA4A	SSH2	TREH	ZNF582
APH1A	CASK	CPOX	EFEMP1	GALNS	HSP90AA1	KRT20	MSX2	P2RX2	PRPH	SEMA4C	SSPN	TREM2	ZNF589
APH1B	CASP1	CPS1	EFEMP2	GALNT11	HSP90B1	KRT25	MT1A	P2RX4	PRPH2	SEMA4G	SSR4	TRERF1	ZNF592
APLNR	CASP10	CPT1A	EFHC1	GALNT12	HSPA1A	KRT3	MT2A	P2RX5	PRPS1	SEMA6D	SST	TREX1	ZNF607
APOA1	CASP12	CPT1B	EFHC2	GALNT13	HSPA1B	KRT31	MTA1	P2RX7	PRRC2A	SEMA7A	SSTR2	TREX2	ZNF627
APOA2	CASP2	CPT1C	EFNA5	GALNT14	HSPA1L	KRT37	MTA2	P2RY11	PRRC2C	SEMG1	SSTR5	TRH	ZNF638
APOA4	CASP3	CPT2	EFNB1	GALNT18	HSPA5	KRT38	MTAP	P2RY12	PRRT2	SEPN1	SSUH2	TRHR	ZNF644
APOA5	CASP5	CPZ	EFR3A	GALNT2	HSPA8	KRT4	MTCH2	P2RY4	PRRX1	SEPP1	SSX1	TRIB1	ZNF668
APOB	CASP8	CR1	EFTUD2	GALNT3	HSPA9	KRT5	MTF1	P4HB	PRSS1	SEPSECS	SSX2	TRIB2	ZNF674
APOBEC1	CASP9	CR1L	EGF	GALNT5	HSPB1	KRT6A	MTFMT	PABPC4L	PRSS12	Sep-12	SSX7	TRIB3	ZNF683
APOBEC3B	CASQ1	CR2	EGFL7	GALNT6	HSPB3	KRT6B	MTG2	PABPN1	PRSS22	Sep-14	ST14	TRIL	ZNF711
APOBEC3G	CASQ2	CRADD	EGFR	GALNT7	HSPB6	KRT6C	MTHFD1	PACRG	PRSS23	Sep-05	ST3GAL1	TRIM17	ZNF721
APOBEC3H	CASR	CRAT	EGLN1	GALNT8	HSPB7	KRT71	MTHFD1L	PACS1	PRSS37	Sep-07	ST3GAL2	TRIM2	ZNF750
APOC1	CAST	CRB1	EGLN2	GALNT9	HSPB8	KRT74	MTHFR	PADI4	PRSS38	Sep-09	ST3GAL3	TRIM21	ZNF75D
APOC2	CAT	CRB2	EGR1	GALNTL5	HSPD1	KRT75	MTHFS	PAFAH1B1	PRSS3P2	SERAC1	ST3GAL4	TRIM22	ZNF77
APOC3	CATSPER1	CRBN	EGR2	GALNTL6	HSPG2	KRT8	MTM1	PAFAH1B2	PRSS56	SERPINA1	ST3GAL5	TRIM24	ZNF780B
APOC4	CATSPER2	CREB1	EGR3	GALP	HSPH1	KRT81	MTMR12	PAFAH1B3	PRSS8	SERPINA10	ST3GAL6	TRIM32	ZNF79
APOC4-APOC2	CATSPER3	CREB3	EHBP1	GALT	HTATSF1	KRT82	MTMR14	PAH	PRTG	SERPINA3	ST5	TRIM33	ZNF80
APOD	CATSPER4	CREB3L3	EHD2	GAMT	HTN3	KRT83	MTMR2	PAK3	PRUNE2	SERPINA5	ST6GAL1	TRIM37	ZNF804A
APOE	CAV1	CREBBP	EHHADH	GAN	HTR1A	KRT85	MTMR8	PAK6	PRX	SERPINA6	ST6GAL2	TRIM5	ZNF81
APOH	CAV3	CRELD1	EHMT1	GAP43	HTR1B	KRT86	MTMR9	PAK7	PRY	SERPINA7	ST6GALNAC1	TRIM54	ZNF813
APOL1	CBFA2T2	CRELD2	EHMT2	GAPDH	HTR2A	KRT9	MTNR1A	PALB2	PRY2	SERPINB11	ST6GALNAC2	TRIM55	ZNF844
APOL3	CBFA2T3	CRH	EIF2AK3	GARS	HTR2B	KRTAP1-1	MTNR1B	PALLD	PSAP	SERPINB3	ST6GALNAC3	TRIM63	ZNHIT6
APOL5	CBFB	CRHR1	EIF2AK4	GAS1	HTR2C	KRTAP4-8	MTO1	PAM16	PSAPL1	SERPINB4	ST6GALNAC4	TRIO	ZNRF1
APOM	CBL	CRIPT	EIF2B1	GAS2L2	HTR3A	KRTAP9-3	MTOR	PAMR1	PSAT1	SERPINB5	ST6GALNAC5	TRIOBP	ZP1
APOPT1	CBLB	CRISP2	EIF2B2	GAS6	HTR3B	KRTCAP3	MTPAP	PANK2	PSCA	SERPINB6	ST6GALNAC6	TRIP11	ZP4
APP	CBLC	CRK	EIF2B3	GATA1	HTR3C	KSR2	MTR	PAPD7	PSEN1	SERPINB7	ST7	TRIP12	ZPBP
APPL1	CBR1	CRKL	EIF2B4	GATA2	HTR3E	KXD1	MTRR	PAPSS2	PSEN2	SERPINC1	ST8SIA1	TRIP13	ZPBP2
APPL2	CBR3	CRLF1	EIF2B5	GATA3	HTR5A	KYNU	MTSS1	PAQR8	PSENEN	SERPIND1	ST8SIA2	TRMT1	ZRSR2
APRT	CBR4	CRLF2	EIF2D	GATA4	HTR6	L1CAM	MTSS1L	PAQR9	PSMA4	SERPINE1	ST8SIA3	TRMT10A	ZSCAN29
APTX	CBS	CRP	EIF2S3	GATA5	HTR7	L2HGDH	MTTP	PARD3B	PSMA6	SERPINF1	ST8SIA4	TRMT44	ZSWIM6
AQP1	CBX2	CRTAP	EIF3B	GATA6	HTRA1	L3MBTL1	MTUS1	PARD6A	PSMA7	SERPINF2	ST8SIA5	TRMT5	
AQP2	CBX4	CRTC1	EIF3C	GATAD1	HTRA2	LACC1	MUC1	PARK2	PSMB8	SERPING1	ST8SIA6	TRMU	
AQP3	CBX5	CRX	EIF3E	GATAD2B	HTT	LAMA1	MUC13	PARK7	PSMB9	SERPINH1	STAC2	TRNT1	
AQP4	CBX8	CRYAA	EIF3G	GATM	HUS1B	LAMA2	MUC17	PARL	PSMC2	SERPINI1	STAC3	TROAP	
AQP5	CBY1	CRYAB	EIF3H	GBA	HUWE1	LAMA3	MUC2	PARM1	PSMC3IP	SERPINI2	STAG1	TROVE2	
AQP7	CC2D1A	CRYBA1	EIF4A3	GBA2	HVCN1	LAMA4	MUC3A	PARN	PSMD2	SERTAD1	STAG3	TRPA1	
AR	CC2D2A	CRYBA2	EIF4E	GBA3	HYAL1	LAMA5	MUC4	PARP1	PSMD7	SESN2	STAMBP	TRPC3	
ARAP1	CCAR2	CRYBA4	EIF4ENIF1	GBE1	HYDIN	LAMB1	MUC5B	PARP2	PSORS1C1	SET	STAMBPL1	TRPC4	
AREL1	CCBE1	CRYBB1	EIF4G1	GBGT1	HYLS1	LAMB2	MUC6	PARS2	PSPC1	SETBP1	STAP1	TRPC5	
ARF4	CCDC103	CRYBB2	EIF4H	GC	IAPP	LAMB3	MUC7	PASK	PSPH	SETD1A	STAR	TRPC6	

ARFGEF2	CCDC107	CRYBB3	ELAC2	GCDH	IARS2	LAMB4	MURC	PAWR	PSPN	SETD2	STARD13	TRPM1
ARG1	CCDC109B	CRYBG3	ELANE	GCGR	IBA57	LAMC1	MUS81	PAX1	PSTPIP1	SETD5	STARD3	TRPM2
ARHGAP18	CCDC11	CRYGA	ELAVL2	GCH1	IBSP	LAMC2	MUSK	PAX2	PSTPIP2	SETD8	STARD9	TRPM3
ARHGAP24	CCDC114	CRYGB	ELF4	GCK	ICAM1	LAMC3	MUT	PAX3	PTAFR	SETD9	STAT1	TRPM4
ARHGAP26	CCDC127	CRYGC	ELK1	GCKR	ICAM4	LAMP1	MUTYH	PAX4	PTCD1	SETDB1	STAT2	TRPM6
ARHGAP29	CCDC136	CRYGD	ELK3	GCLC	ICAM5	LAMP2	MVK	PAX5	PTCH1	SETDB2	STAT3	TRPM7
ARHGAP31	CCDC14	CRYGS	ELMOD2	GCLM	ICK	LAMTOR2	MX1	PAX6	PTCH2	SETX	STAT4	TRPS1
ARHGAP6	CCDC151	CRYM	ELMOD3	GCM2	ICOS	LAPTM4B	MXI1	PAX7	PTCHD1	SEZ6	STAT5B	TRPV1
ARHGAP9	CCDC170	CRYZL1	ELN	GCNT1	ID3	LARGE	MXRA5	PAX8	PTCHD3	SEZ6L	STAT6	TRPV3
ARHGDIA	CCDC176	CSAG1	ELOVL2	GCNT2	ID4	LARP7	MYADML2	PAX9	PTCSC3	SEZ6L2	STEAP3	TRPV4
ARHGEF10	CCDC18	CSDE1	ELOVL4	GCSH	IDE	LARS	MYB	PAXIP1	PTDSS1	SF3B1	STEAP4	TRPV5
ARHGEF11	CCDC19	CSF1	ELOVL5	GDAP1	IDH1	LARS2	MYBBP1A	PBRM1	PTEN	SF3B4	STH	TRRAP
ARHGEF12	CCDC22	CSF1R	ELP2	GDF1	IDH2	LAS1L	MYBL2	PBX1	PTF1A	SFRP1	STIL	TSC1
ARHGEF15	CCDC28B	CSF2	ELP4	GDF15	IDH3B	LAT	MYBPC1	PBX4	PTGDR	SFTPA1	STIM1	TSC2
ARHGEF28	CCDC30	CSF2RA	EMC1	GDF2	IDO1	LATS1	MYBPC3	PC	PTGDR2	SFTPA2	STK10	TSEN15
ARHGEF4	CCDC39	CSF2RB	EMC4	GDF3	IDS	LBP	MYBPHL	PCBD1	PTGDS	SFTPB	STK11	TSEN2
ARHGEF6	CCDC40	CSF3R	EMD	GDF5	IDUA	LBR	MYC	PCBP3	PTGER2	SFTPC	STK11IP	TSEN34
ARHGEF7	CCDC41	CSGALNACT1	EME1	GDF6	IER2	LCA5	MYCBP2	PCCA	PTGER4	SFTPD	STK19	TSEN54
ARHGEF9	CCDC47	CSH1	EMG1	GDF9	IER3IP1	LCAT	MYCL	PCCB	PTGES2	SFXN4	STK3	TSFM
ARID1A	CCDC50	CSMD1	EML1	GDI1	IFI30	LCE3B	MYCN	PCDH10	PTGIR	SGCA	STK32A	TSG101
ARID1B	CCDC65	CSMD2	EMP2	GDNF	IFI44L	LCE3C	MYD88	PCDH11X	PTGIS	SGCB	STK33	TSHB
ARID2	CCDC66	CSMD3	EMR1	GDPD5	IFIH1	LCE5A	MYEF2	PCDH15	PTGS1	SGCD	STK35	TSHR
ARID3C	CCDC78	CSNK1A1L	EMX1	GEMIN2	IFITM3	LCK	MYF5	PCDH18	PTGS2	SGCE	STK36	TSHZ1
ARID4A	CCDC8	CSNK1D	EMX2	GEMIN4	IFITM5	LCN10	MYF6	PCDH19	PTH	SGCG	STK38L	TSHZ2
ARID4B	CCDC85C	CSNK1E	EN1	GFAP	IFNA10	LCT	MYH10	PCDH9	PTH1R	SGCZ	STK39	TSKS
ARID5A	CCDC88C	CSNK1G1	EN2	GFER	IFNA17	LDB1	MYH11	PCDHA1	PTH2R	SGK1	STK4	TSLP
ARIH1	CCDC90B	CSNK1G2	ENAM	GFI1	IFNA2	LDB3	MYH13	PCDHA10	PTHLH	SGK223	STMN1	TSNAX
ARL11	CCHCR1	CSNK2A2	ENDOV	GFI1B	IFNAR1	LDHA	MYH14	PCDHA13	PTK2	SGOL1	STON1-GTF2A1L	TSPAN12
ARL13B	CCK	CSNK2A3	ENG	GFM1	IFNAR2	LDHB	MYH15	PCDHA3	PTK2B	SGSH	STOX1	TSPAN17
ARL14EP	CCKAR	CSPP1	ENO1	GFM2	IFNB1	LDLR	MYH2	PCDHAC2	PTK7	SGSM2	STRA6	TSPAN7
ARL2BP	CCKBR	CSRP3	ENO3	GFOD1	IFNG	LDLRAD4	MYH3	PCDHB16	PTPLA	SGSM3	STRADA	TSPEAR
ARL3	CCL11	CST3	ENOX1	GFPT1	IFNGR1	LDLRAP1	MYH6	PCDHB4	PTPN1	SH2B1	STRC	TSPO
ARL6	CCL13	CST6	ENPP1	GFPT2	IFNGR2	LECT2	MYH7	PCK1	PTPN11	SH2B3	STS	TSPYL1
ARL6IP1	CCL17	CST9	ENPP2	GFRA1	IFNL3	LEF1	MYH7B	PCK2	PTPN12	SH2D1A	STT3A	TSPYL2
ARL6IP5	CCL2	CSTA	ENSA	GFRA2	IFNL4	LEFTY2	MYH8	PCLO	PTPN13	SH3BP2	STT3B	TSR2
ARL6IP6	CCL22	CSTB	ENTPD1	GGCX	IFRD1	LEMD3	MYH9	PCM1	PTPN14	SH3BP4	STUB1	TSSC4
ARMC4	CCL26	CSTF2T	ENTPD5	GGH	IFT122	LENG1	MYL1	PCMT1	PTPN2	SH3GL1	STX11	TSSK2
ARMC5	CCL3	CT45A5	EOGT	GGT5	IFT140	LEP	MYL2	PCNA	PTPN21	SH3PXD2B	STX16	TSSK3
ARMS2	CCL3L1	CTBP2	EOMES	GH1	IFT172	LEPR	MYL3	PCNT	PTPN22	SH3TC2	STX1A	TSSK4
ARNT2	CCL4L1	CTC1	EP300	GH2	IFT27	LEPRE1	MYLIP	PCOLCE	PTPN23	SHANK1	STX1B	TST
ARPC3	CCL5	CTCF	EP400	GHR	IFT43	LEPREL1	MYLK	PCP4	PTPN3	SHANK2	STX3	TTBK2
ARSA	CCL7	CTDP1	EPAS1	GHRH	IFT80	LETM1	MYLK2	PCSK1	PTPN6	SHANK3	STXBP1	TTC14
ARSB	CCM2	CTF1	EPB41	GHRHR	IFT88	LFNG	MYLPF	PCSK2	PTPRA	SHARPIN	STXBP2	TTC19
ARSE	CCNA2	CTGF	EPB41L1	GHRL	IGBP1	LGALS13	MYO15A	PCSK5	PTPRB	SHBG	STXBP3	TTC21B
ARSF	CCND1	CTH	EPB41L2	GHSR	IGF1	LGALS2	MYO18A	PCSK7	PTPRC	SHC1	STXBP5	TTC28
ARSH	CCND2	CTHRC1	EPB41L4A	GIF	IGF1R	LGALS3	MYO18B	PCSK9	PTPRCAP	SHFM1	STXBP5L	TTC37
ARSI	CCND3	CTLA4	EPB42	GIGYF2	IGF2	LGALS9B	MYO1A	PCYT1A	PTPRD	SHH	SUCLA2	TTC4
ART4	CCNE1	CTNNA1	EPC1	GIMAP8	IGF2BP2	LGI1	MYO1C	PDCD1	PTPRF	SHKBP1	SUCLG1	TTC7A
ARV1	CCNO	CTNNA2	EPC2	GIP	IGF2R	LGI4	MYO1D	PDCD10	PTPRG	SHMT1	SUCLG2	TTC8
ARVCF	CCR1	CTNNA3	EPCAM	GIPC1	IGFALS	LGR4	MYO1E	PDCD11	PTPRJ	SHOC2	SUCO	TTF2
ARX	CCR2	CTNNB1	EPG5	GIPC3	IGFBP1	LGR5	MYO1F	PDCD5	PTPRK	SHOX	SUFU	TTI2
AS3MT	CCR3	CTNND1	EPHA2	GIPR	IGFBP3	LHB	MYO3A	PDE10A	PTPRN2	SHOX2	SUGCT	TTK
ASAH1	CCR5	CTNND2	EPHA3	GIT1	IGFBP5	LHCGR	MYO5A	PDE11A	PTPRO	SHROOM1	SULF1	TTLL1
ASAH2	CCR6	CTNS	EPHA4	GJA1	IGFBP7	LHFP	MYO5B	PDE3A	PTPRQ	SHROOM3	SULF2	TTLL11
ASAP1	CCR7	CTPS1	EPHA5	GJA3	IGHA1	LHFPL5	MYO5C	PDE3B	PTPRT	SHROOM4	SULT1A1	TTLL5
ASAP2	CCRL2	CTR9	EPHA7	GJA4	IGHG2	LHX1	MYO6	PDE4B	PTRF	SI	SULT1A3	TTN
ASB1	CCS	CTRC	EPHA8	GJA5	IGHM	LHX3	MYO7A	PDE4C	PTRH2	SIAE	SULT1B1	TTPA
ASB10	CCT5	CTSA	EPHB1	GJA8	IGHMBP2	LHX4	MYO7B	PDE4D	PTS	SIGLEC12	SULT1C2	TTR
ASCC1	CCT7	CTSB	EPHB2	GJB1	IGKC	LHX6	MYO9B	PDE4DIP	PUF60	SIGLEC14	SULT1C3	TUB
ASCC3	CCT8L2	CTSC	EPHB4	GJB2	IGLL1	LHX8	MYOC	PDE5A	PURA	SIGLEC16	SULT1E1	TUBA1A
ASCL1	CD109	CTSD	EPHB6	GJB3	IGSF1	LIAS	MYOCD	PDE6A	PUS1	SIGMAR1	SULT2A1	TUBA3E
ASH1L	CD14	CTSF	EPHX1	GJB4	IGSF22	LIF	MYOD1	PDE6B	PUS3	SIK1	SULT2B1	TUBA4A
ASIC3	CD151	CTSG	EPHX2	GJB6	IGSF3	LIFR	MYOF	PDE6C	PVR	SIK3	SULT4A1	TUBA8
ASIP	CD163	CTSH	EPM2A	GJC1	IGSF8	LIG1	MYOM1	PDE6D	PVRL1	SIL1	SUMF1	TUBB
ASL	CD163L1	CTSK	EPO	GJC2	IHH	LIG3	MYOM2	PDE6G	PVRL2	SIM1	SUMO1	TUBB1
ASMT	CD177	CTSZ	EPOR	GJC3	IKBIP	LIG4	MYOT	PDE6H	PVRL3	SIM2	SUMO4	TUBB2A
ASMTL	CD19	CTTNBP2	EPS8	GJD2	IKBKAP	LILRA3	MYOZ2	PDE7B	PVRL4	SIPA1	SUN1	TUBB2B
ASNS	CD1A	CUBN	EPS8L3	GK	IKBKB	LIM2	MYPN	PDE8B	PWWP2A	SIPA1L1	SUN2	TUBB3
ASPA	CD1E	CUEDC1	EPX	GLA	IKBKG	LIMD1	MYSM1	PDGFA	PXDN	SIRT1	SUOX	TUBB4A
ASPH	CD200	CUL2	EPYC	GLB1	IKZF1	LIMK1	MYT1	PDGFB	PXDNL	SIRT3	SUPT16H	TUBG1
ASPM	CD207	CUL3	ERAP1	GLCCI1	IKZF3	LIN28A	MYT1L	PDGFC	PYCARD	SIRT5	SUPT5H	TUBGCP4
ASPN	CD209	CUL4B	ERAP2	GLDC	IL10	LIN28B	N4BP2L2	PDGFRA	PYCR1	SIX1	SURF1	TUBGCP5
ASPRV1	CD22	CUL5	ERBB2	GLE1	IL10RA	LINS	NAA10	PDGFRB	PYCR2	SIX2	SUV420H1	TUBGCP6
ASPSCR1	CD226	CUL7	ERBB2IP	GLI1	IL10RB	LIPA	NAA15	PDGFRL	PYCRL	SIX3	SUZ12	TUFM
ASS1	CD24	CUX2	ERBB3	GLI2	IL11	LIPC	NAALADL2	PDHA1	PYGL	SIX5	SV2B	TULP1
ASTL	CD244	CUZD1	ERBB4	GLI3	IL11RA	LIPE	NACC1	PDHB	PYGM	SIX6	SVIL	TULP3
ASTN2	CD247	CWF19L1	ERCC1	GLIPR1L2	IL12A	LIPG	NADK2	PDHX	PYGO1	SKI	SYCE1	TUSC3
ASUN	CD27	CX3CR1	ERCC2	GLIS2	IL12B	LIPH	NAGA	PDIA4	PYY	SKIV2L	SYCE2	TWIST1
ASXL1	CD2AP	CXCL10	ERCC3	GLIS3	IL12RB1	LIPI	NAGLU	PDK1	PZP	SKP2	SYCP3	TWIST2
ASXL3	CD320	CXCL11	ERCC4	GLMN	IL12RB2	LIPK	NAGPA	PDK3	QARS	SLA2	SYK	TWSG1
ATCAY	CD33	CXCL12	ERCC5	GLO1	IL13	LIPN	NAGS	PDK4	QDPR	SLBP	SYN1	TXLNB
ATE1	CD36	CXCL16	ERCC6	GLP1R	IL16	LIPT1	NAIP	PDLIM3	QKI	SLC10A1	SYN2	TXN2
ATF1	CD38	CXCL5	ERCC6L2	GLRA1	IL17A	LITAF	NALCN	PDLIM4	QSER1	SLC10A2	SYN3	TXNIP
ATF3	CD3D	CXCR1	ERCC8	GLRA4	IL17F	LLGL1	NAMPT	PDLIM5	RAB10	SLC11A1	SYNCRIP	TXNL4A
ATF5	CD3E	CXCR2	ERF	GLRB	IL17RA	LLGL2	NANOS1	PDP1	RAB11FIP5	SLC11A2	SYNE1	TXNRD2
ATF6	CD3EAP	CXCR3	ERG	GLRX5	IL17RB	LMAN1	NANOS3	PDPK1	RAB18	SLC12A1	SYNE2	TYK2
ATG12	CD3G	CXCR4	ERLIN1	GLS	IL17RC	LMBR1	NANP	PDPR	RAB23	SLC12A3	SYNE4	TYMP
ATG16L1	CD4	CYB5A	ERLIN2	GLTSCR1	IL17RD	LMBRD1	NANS	PDSS1	RAB25	SLC12A4	SYNGAP1	TYMS
ATG5	CD40	CYB5R3	ERMAP	GLUD1	IL17REL	LMF1	NAP1L2	PDSS2	RAB27A	SLC12A5	SYNGR1	TYR
ATG7	CD40LG	CYB5R4	ERMARD	GLUD2	IL18	LMNA	NAPRT1	PDX1	RAB27B	SLC12A6	SYNJ1	TYRO3
ATIC	CD44	CYBA	ERRFI1	GLUL	IL18R1	LMNB1	NARS2	PDXK	RAB28	SLC13A1	SYNM	TYROBP
ATL1	CD46	CYBB	ESAM	GLYCTK	IL18RAP	LMNB2	NAT1	PDYN	RAB2A	SLC13A2	SYNPO	TYRP1
ATL3	CD5	CYBRD1	ESCO2	GM2A	IL19	LMO2	NAT10	PDZD2	RAB33B	SLC13A5	SYNPR	TYW1
ATM	CD55	CYC1	ESPN	GMDS	IL1A	LMO4	NAT2	PDZD7	RAB34	SLC14A1	SYP	U2AF1
ATN1	CD58	CYCS	ESR1	GMIP	IL1B	LMOD3	NAT8	PEAR1	RAB39B	SLC14A2	SYT1	UACA
ATOH7	CD59	CYFIP1	ESR2	GMPPA	IL1R1	LMTK3	NAT8L	PECAM1	RAB3GAP1	SLC15A1	SYT11	UBA1
ATP10A	CD72	CYLD	ESRRA	GMPPB	IL1RAPL1	LMX1B	NAV1	PECR	RAB3GAP2	SLC16A1	SYT14	UBA3
ATP10D	CD74	CYP11A1	ESRRB	GMPS	IL1RAPL2	LNX2	NAV2	PEMT	RAB40AL	SLC16A12	SYT17	UBA7
ATP11B	CD79A	CYP11B1	ESRRG	GNA11	IL1RL1	LONP1	NAV3	PENK	RAB7A	SLC16A2	SYT2	UBAC2

ATP13A2	CD79B	CYP11B2	ETFA	GNA14	IL1RN	LOR	NBAS	PEPD	RAB7L1	SLC16A3	SYTL3	UBAP2
ATP13A4	CD80	CYP17A1	ETFB	GNAI2	IL2	LOX	NBEA	PER1	RABGGTA	SLC17A1	SYTL5	UBB
ATP13A5	CD81	CYP19A1	ETFDH	GNAI3	IL20RA	LOXHD1	NBEAL2	PER2	RABL6	SLC17A3	SYVN1	UBD
ATP1A2	CD82	CYP1A1	ETHE1	GNAL	IL20RB	LOXL1	NBN	PER3	RAC1	SLC17A5	SZT2	UBE2A
ATP1A3	CD86	CYP1A2	ETNPPL	GNAO1	IL21	LOXL2	NBPF1	PET100	RAC2	SLC17A8	T	UBE2B
ATP1B1	CD8A	CYP1B1	ETS1	GNAQ	IL21R	LPA	NCALD	PEX1	RAD21	SLC17A9	TAAR1	UBE2I
ATP1B4	CD96	CYP21A1P	ETS2	GNAS	IL23R	LPAR1	NCAM1	PEX10	RAD21L1	SLC18A1	TAAR2	UBE2NL
ATP2A1	CDA	CYP21A2	ETV4	GNAT1	IL27RA	LPAR3	NCAN	PEX11B	RAD23B	SLC18A2	TAAR6	UBE2T

Suppl. Table 9: List of somatic mutations found in Family 2, patient II.2, LS+ MLH1mut

CHROM	START	REF	ALT	GENE_NAME	VARCLASS	CDNA_CHG	AA_CHG	ClinVar_Significance	ClinVar_Diseas	OncoMD	COSMIC
chr7	1527511	G	A	INTS1	MISSENSE	c.2401C>T	p.Arg801Trp	NA	NA	NA	NA
chr7	1535091	C	T	INTS1	MISSENSE	c.1810G>A	p.Ala604Thr	NA	NA	NA	NA
chr7	2578097	G	A	BRAT1	MISSENSE	c.2072C>T	p.Ala691Val	NA	NA	NA	NA
chr7	20668447	T	C	ABCB5	MISSENSE	c.245T>C	p.Val82Ala	NA	NA	NA	NA
chr7	36483467	G	A	ANLN	MISSENSE	c.3074G>A	p.Arg1025His	NA	NA	NA	NA
chr7	39247001	C	T	POU6F2	MISSENSE	c.293C>T	p.Thr98Met	NA	NA	chr7*39247001*39247001*C*T*1*1*Breast_cancer	chr7*39247001*39247001*COSM163654*C*T*1*NS*1
chr7	50682496	C	T	GRB10	MISSENSE	c.1066G>A	p.Ala356Thr	NA	NA	NA	NA
chr7	55910724	G	A	SEPT14	MISSENSE	c.469C>T	p.Arg157Cys	NA	NA	NA	NA
chr7	73010179	C	T	MLXIPL	MISSENSE	c.2179G>A	p.Ala727Thr	NA	NA	NA	NA
chr7	73752936	G	A	CLIP2	MISSENSE	c.280G>A	p.Val94Met	NA	NA	NA	NA
chr7	82582768	G	A	PCLO	MISSENSE	c.7501C>T	p.His2501Tyr	NA	NA	NA	NA
chr7	84628896	C	T	SEMA3D	MISSENSE	c.2194G>A	p.Glu732Lys	NA	NA	NA	NA
chr7	90894493	C	T	FZD1	NONSENSE	c.298C>T	p.Gln100Ter	NA	NA	NA	NA
chr7	94257615	G	A	SGCE	NONSENSE	c.289C>T	p.Arg97Ter	PATHOGENIC	Myoclonic_dystonia	chr7*94257615*94257615*G*A*1*1*Colorectal_cancer|chr7*94257615*94257615*C*T*1*1*Colon_cancer	chr7*94257615*94257615*COSM287685*G*A*1*large_intestine*1
chr7	97488558	C	T	ASNS	MISSENSE	c.640G>A	p.Ala214Thr	NA	NA	NA	NA
chr7	99786612	G	A	STAG3	MISSENSE	c.688G>A	p.Ala230Thr	NA	NA	NA	NA
chr7	1E+08	T	C	ZAN	MISSENSE	c.2927T>C	p.Leu976Pro	NA	NA	NA	NA
chr7	1.03E+08	A	G	RELN	MISSENSE	c.2119T>C	p.Phe707Leu	NA	NA	NA	NA
chr7	1.05E+08	G	A	KMT2E	MISSENSE	c.2444G>A	p.Cys815Tyr	NA	NA	NA	NA
chr7	1.05E+08	G	A	KMT2E	MISSENSE	c.2888G>A	p.Arg963His	NA	NA	chr7*104747653*104747653*G*A*2*2*Stomach_(gastric)_cancer	chr7*104747653*104747653*COSM1257804*G*A*1*oesophagus*1
chr7	1.08E+08	T	C	LAMB1	MISSENSE	c.4481A>G	p.Asp1494Gly	NA	NA	NA	NA
chr7	1.08E+08	C	T	LAMB1	MISSENSE	c.2744G>A	p.Arg915His	NA	NA	NA	NA
chr7	1.11E+08	A	C	DOCK4	MISSENSE	c.5354T>G	p.Leu1785Arg	NA	NA	NA	NA
chr7	1.12E+08	G	A	IFRD1	MISSENSE	c.945G>A	p.Met315Ile	NA	NA	NA	NA
chr7	1.17E+08	G	T	ST7	MISSENSE	c.1402G>T	p.Gly468Cys	NA	NA	NA	NA
chr7	1.2E+08	G	A	KCND2	MISSENSE	c.419G>A	p.Arg140His	NA	NA	chr7*119915105*119915105*G*G*1*1*Ovarian_cancer|chr7*119915105*119915105*G*A*3*3*Endometrial_cancer	chr7*119915105*119915105*COSM75076*G*A*2*ovary
chr7	1.29E+08	C	T	SMO	MISSENSE	c.2057C>T	p.Ala686Val	UNKNOWN	not_specified	chr7*128851985*128851985*C*T*1*1*Renal_cancer	chr7*128851985*128851985*COSM484810*C*T*1*kidney*1
chr7	1.47E+08	C	T	CNTNAP2	MISSENSE	c.439C>T	p.Arg147Trp	NA	NA	NA	NA
chr7	1.5E+08	C	T	GIMAP8	MISSENSE	c.716C>T	p.Pro239Leu	NA	NA	NA	NA
chr7	1.51E+08	G	A	NUB1	MISSENSE	c.511G>A	p.Val171Met	NA	NA	NA	NA
chr7	1.52E+08	A	G	GALNTL5	MISSENSE	c.836A>G	p.Asn279Ser	NA	NA	NA	NA
chr7	1.52E+08	T	C	GALNT11	MISSENSE	c.37T>C	p.Cys13Arg	NA	NA	NA	NA
chr7	1.52E+08	C	T	KMT2C	MISSENSE	c.4442G>A	p.Arg1481Gln	UNKNOWN	not_specified	NA	NA
chr7	1.52E+08	G	T	KMT2C	MISSENSE	c.2189C>A	p.Ser730Tyr	NA	NA	NA	NA
chr7	1.55E+08	C	T	DPP6	MISSENSE	c.2252C>T	p.Ala751Val	NA	NA	NA	NA
chr7	720280	CG	C	PRKAR1B	FRAMESHIFT-DEL	c.260delC	p.Pro87ArgfsTer5	NA	NA	chr7*720281*720281*C*del*1*1*Colorectal_cancer	chr7*720280*720281*COSM1451886*CG*C*1*large_intestine*1

chr7	5401258	CG	C	TNRC18	FRAMESHIFT-DEL	c.4627delC	p.Arg1543AlafsTer13	NA	NA	chr7*5401258*5401258*ins*G*1*1*Stomach_(gastric)_cancer|chr7*5401258*5401258*ins*C*1*1*Salivary_gland_cancer|chr7*5401258*5401258*C*T*1*1*Endometrial_cancer|chr7*5401259*5401259*G*del*2*2*Stomach_(gastric)_cancer	chr7*5401258*5401258*COSM1090804*C*T*1*endometrium*1|chr7*5401258*5401258*COSM1090805*C*T*1*endometrium*1|chr7*5401258*5401258*COSM1090802*C*T*1*endometrium*1
chr7	16298550	CACAA	C	ISPD	FRAMESHIFT-DEL	c.1017_1020delTTGT	p.Cys340Ter	NA	NA	NA	NA
chr7	17382630	TTCA	T	AHR	INFRAME-DEL	c.2490_2492delTCA	p.His832del	NA	NA	chr7*17382631*17382633*TCA*del*1*1*Lung_cancer	chr7*17382630*17382633*COSM391621*TTCA*T*1*lung*1
chr7	45614573	GC	G	ADCY1	FRAMESHIFT-DEL	c.432delC	p.Ala146ProfsTer40	NA	NA	NA	NA
chr7	70231133	GT	G	AUTS2	FRAMESHIFT-DEL	c.1503delT	p.Leu503TrpfsTer91	NA	NA	NA	NA
chr7	82582943	CT	C	PCLO	FRAMESHIFT-DEL	c.7325delA	p.Lys2442SerfsTer2	NA	NA	chr7*82582943*82582943*C*A*1*1*Myeloma|chr7*82582944*82582944*T*del*2*2*Stomach_(gastric)_cancer|chr7*82582944*82582944*T*G*1*1*Endometrial_cancer	chr7*82582943*82582943*COSM329513*C*A*1*haematopoietic_and_lymphoid_tissue*1|chr7*82582943*82582943*COSM329514*C*A*1*haematopoietic_and_lymphoid_tissue*1|chr7*82582943*82582943*COSM329515*C*A*1*haematopoietic_and_lymphoid_tissue*1|chr7*82582943*82582944*COSM1452371*CT*C*6*pancreas
chr7	82763788	CT	C	PCLO	FRAMESHIFT-DEL	c.3077delA	p.Lys1026SerfsTer11	NA	NA	chr7*82763789*82763789*A*del*1*1*Colorectal_cancer	chr7*82763788*82763789*COSM1452429*CT*C*1*large_intestine*1|chr7*82763788*82763789*COSM1452431*CT*C*1*large_intestine*1|chr7*82763788*82763789*COSM1452430*CT*C*1*large_intestine*1
chr7	87170759	GATC	G	ABCB1	INFRAME-DEL	c.2230_2232delGAT	p.Asp744del	NA	NA	NA	NA

chr7	91732038	T	TG	AKAP9	FRAMESHIFT-INS	c.11228_11229insG	p.Gln3746AlafsTer52	NA	NA	chr7*91732038*91732039*ins*G*3*1*Colorectal_cancer	chr7*91732038*91732038*COSM1452810*T*TG*3*large_intestine*3|chr7*91732038*91732038*COSM1452811*T*TG*3*large_intestine*3|chr7*91732038*91732039*COSM1452813*TG*T*2*large_intestine
chr7	95045559	T	TA	PON2	FRAMESHIFT-INS	c.195dupT	p.Ser66Ter	NA	NA	NA	chr7*95045559*95045560*COSM1453035*TA*T*2*large_intestine*2
chr7	1E+08	CG	C	PCOLCE	FRAMESHIFT-DEL	c.478delG	p.Arg162GlyfsTer9	NA	NA	NA	NA
chr7	1.01E+08	TG	T	AP1S1	FRAMESHIFT-DEL	c.357delG	p.Asp122MetfsTer11	NA	NA	chr7*100802404*100802404*ins*G*2*2*Lung_cancer|chr7*100802404*100802405*ins*GG*1*1*Colorectal_cancer|chr7*100802404*100802405*ins*G*2*2*Breast_cancer|chr7*100802405*100802405*G*del*1*1*Stomach_(gastric)_cancer|chr7*100802405*100802405*G*A*1*1*Ovarian_canc	chr7*100802404*100802404*COSM1446813*T*TGG*1*large_intestine*1|chr7*100802404*100802404*COSM1446814*T*TG*2*large_intestine
chr7	1.07E+08	GA	G	PIK3CG	FRAMESHIFT-DEL	c.3175delA	p.Asn1060MetfsTer48	NA	NA	NA	NA
chr7	1.17E+08	AGTTTGGGG	A	CFTR	FRAMESHIFT-DEL	c.2076_2083delGTTTGGGG	p.Phe693LysfsTer34	UNKNOWN:UNKNOWN:UNCERTAIN_SIGNIFICANCE:UNKNOWN	Cystic_fibrosis:Cystic_fibrosis:not_provided:Cystic_fibrosis	NA	NA
chr7	1.37E+08	T	TAAAAAAAAA	CHRM2	INFRAME-INS	c.1107_1108insAAAAAAAAA	p.Lys370_Gln371insLysLysLys	NA	NA	NA	NA
chr7	1.37E+08	AGCAGCCTGC	A	CHRM2	INFRAME-DEL	c.1110_1118delGCAGCCTGC	p.Gln371_Ala373del	LIKELY_BENIGN	not_specified	NA	NA
chr20	5932719	A	G	MCM8	MISSENSE	c.58A>G	p.Arg20Gly	NA	NA	NA	NA
chr20	10620472	T	C	JAG1	MISSENSE	c.3331A>G	p.Thr1111Ala	NA	NA	NA	NA
chr20	31021316	G	A	ASXL1	MISSENSE	c.1315G>A	p.Ala439Thr	NA	NA	NA	NA
chr20	39795129	A	G	PLCG1	MISSENSE	c.2014A>G	p.Ser672Gly	NA	NA	NA	NA
chr20	42788646	C	T	JPH2	MISSENSE	c.781G>A	p.Ala261Thr	NA	NA	NA	NA
chr20	57484420	C	T	GNAS	MISSENSE	c.2530C>T	p.Arg844Cys	PATHOGENIC|PATHOGENIC|PATHOGENIC|PATHOGENIC	PATHOGENIC	PATHOGENIC|PATHOGENIC|PATHOGENIC|PATHOGENIC	Somatotroph_adenoma
chr20	57599030	A	G	TUBB1	MISSENSE	c.548A>G	p.Tyr183Cys	NA	NA	NA	NA

chr20	19956393	A	AC	RIN2	FRAMESHIFT-INS	c.1871_1872insC	p.Ile627HisfsTer7	PATHOGENIC	Macrocephaly_alopecia_cutis_laxa_and_scoliosis	chr20*19956393*19956394*ins*CC*1*1*Colorectal_cancer|chr20*19956393*19956394*ins*C*1*1*Colorectal_cancer	chr20*19956393*19956393*COSM1410650*A*AC*1*large_intestine*1|chr20*19956393*19956393*COSM1410647*A*ACC*1*large_intestine*1|chr20*19956393*19956393*COSM1410649*A*AC*1*large_intestine*1|chr20*19956393*19956393*COSM1410648*A*ACC*1*large_intestine*1|chr20*19956393*19956394*COSM1410651*AC*A*2*large_intestine*2|chr20*19956393*19956394*COSM1410652*AC*A*2*large_intestine*2
chr20	33588589	CAGA	C	MYH7B	INFRAME-DEL	c.5324_5326delAGA	p.Lys1778del	NA	NA	NA	NA
chr20	43058257	CCT	C	HNF4A	FRAMESHIFT-DEL	c.1378_1379delCT	p.Ser461CysfsTer45	NA	NA	NA	NA
chr20	44047556	TTCA	T	PIGT	INFRAME-DEL	c.431_433delTCA	p.Ile145del	NA	NA	NA	NA
chr20	47989890	CG	C	KCNB1	FRAMESHIFT-DEL	c.2206delC	p.Arg736GlyfsTer9	NA	NA	chr20*47989891*47989891*G*del*1*1*Stomach_(gastric)_cancer	chr20*47989890*47989890*COSM1412372*C*CG*1*large_intestine*1
chr20	51872444	T	TC	TSHZ2	FRAMESHIFT-INS	c.2447_2448insC	p.Met819HisfsTer11	NA	NA	chr20*51872444*51872445*ins*C*1*1*Colorectal_cancer	chr20*51872444*51872444*COSM1412538*T*TC*1*large_intestine*1|chr20*51872444*51872445*COSM1412539*TC*T*2*large_intestine*2
chr22	17589947	C	T	IL17RA	MISSENSE	c.1838C>T	p.Pro613Leu	NA	NA	NA	NA
chr22	18028647	G	A	CECR2	MISSENSE	c.3607G>A	p.Gly1203Arg	NA	NA	chr22*18028647*18028647*G*A*1*1*Lung_cancer	chr22*18028647*18028647*COSM1534793*G*A*1*lung*1|chr22*18028647*18028647*COSM1534794*G*A*1*lung*1
chr22	18029011	C	T	CECR2	MISSENSE	c.3971C>T	p.Thr1324Met	NA	NA	NA	chr22*18029011*18029011*COSM1592833*C*T*1*endometrium*1|chr22*18029011*18029011*COSM1031859*C*T*1*endometrium*1

chr22	20130522	C	G	ZDHHC8	MISSENSE	c.1369C>G	p.Pro457Ala	NA	NA	chr22*20130522*20130522*C*del*1*1*Stomach_(gastric)_cancer	chr22*20130521*20130522*COSM1414944*GC*G*7*large_intestine*7
chr22	20230372	C	T	RTN4R	MISSENSE	c.284G>A	p.Arg95Gln	NA	NA	chr22*20230372*20230372*C*T*1*1*Endometrial_cancer	chr22*20230372*20230372*COSM1032201*C*T*1*endometrium*1
chr22	25627692	C	T	CRYBB2	MISSENSE	c.571C>T	p.Arg191Cys	NA	NA	NA	NA
chr22	26422700	G	A	MYO18B	MISSENSE	c.6760G>A	p.Val2254Met	NA	NA	chr22*26422700*26422700*G*A*2*2*Colorectal_cancer	chr22*26422700*26422700*COSM273716*G*A*1*large_intestine*1
chr22	26422832	G	A	MYO18B	MISSENSE	c.6892G>A	p.Glu2298Lys	NA	NA	NA	NA
chr22	27008042	G	A	CRYBB1	MISSENSE	c.293C>T	p.Ala98Val	NA	NA	chr22*27008042*27008042*C*T*1*1*Prostate_cancer	chr22*27008042*27008042*COSM243859*G*A*1*prostate*1
chr22	28559496	A	G	TTC28	MISSENSE	c.1025T>C	p.Leu342Pro	NA	NA	NA	NA
chr22	28702505	C	T	TTC28	MISSENSE	c.508G>A	p.Ala170Thr	NA	NA	NA	NA
chr22	29668390	G	T	EWSR1	MISSENSE	c.72G>T	p.Gln24His	NA	NA	NA	NA
chr22	32198783	G	A	DEPDC5	MISSENSE	c.1040G>A	p.Arg347His	NA	NA	chr22*32198783*32198783*G*A*1*1*Endometrial_cancer	chr22*32198783*32198783*COSM1033540*G*A*3*large_intestine
chr22	35782925	C	T	HMOX1	MISSENSE	c.392C>T	p.Ala131Val	NA	NA	NA	NA
chr22	37420654	G	A	MPST	MISSENSE	c.458G>A	p.Arg153His	NA	NA	chr22*37420654*37420654*G*T*1*1*Lung_cancer	chr22*37420654*37420654*COSM1193814*G*T*1*lung*1
chr22	38696914	C	T	CSNK1E	MISSENSE	c.380G>A	p.Arg127Gln	NA	NA	NA	NA
chr22	40804309	C	T	SGSM3	MISSENSE	c.1658C>T	p.Thr553Met	NA	NA	chr22*40804309*40804309*C*T*1*1*Lung_cancer	chr22*40804309*40804309*COSM1196136*C*T*1*lung*1
chr22	40804386	C	A	SGSM3	MISSENSE	c.1735C>A	p.Pro579Thr	NA	NA	NA	NA
chr22	46859651	C	T	CELSR1	MISSENSE	c.4136G>A	p.Arg1379His	NA	NA	NA	NA
chr22	50192689	C	T	BRD1	MISSENSE	c.1603G>A	p.Ala535Thr	NA	NA	chr22*50192689*50192689*C*T*1*1*Lung_cancer	chr22*50192689*50192689*COSM1190486*C*T*1*lung*1|chr22*50192689*50192689*COSM1190485*C*T*1*lung*1
chr22	19163947	CCTT	C	SLC25A1	INFRAME-DEL	c.805_807delAAG	p.Lys269del	NA	NA	NA	chr22*19163947*19163950*COSM1414861*CCTT*C*1*large_intestine*1
chr22	19195775	C	CT	CLTCL1	FRAMESHIFT-INS	c.3488dupA	p.Arg1165ProfsTer2	NA	NA	NA	NA
chr22	24134056	TA	T	SMARCB1	FRAMESHIFT-DEL	c.208delA	p.Thr72GlnfsTer13	PATHOGENIC	SCHWANNOMATOSIS_1	NA	NA
chr22	36708256	CG	C	MYH9	FRAMESHIFT-DEL	c.1565delC	p.Pro522ArgfsTer25	NA	NA	chr22*36708257*36708257*G*del*1*1*Stomach_(gastric)_cancer	NA
chr22	40756477	TG	T	ADSL	FRAMESHIFT-DEL	c.774delG	p.Ala260HisfsTer52	NA	NA	NA	NA

chr22	42564715	AG	A	TCF20	FRAMESHIFT-DEL	c.5826delC	p.Leu1943CysfsTer118	NA	NA	chr22*42564715*42564715*ins*G*1*1*Stomach_(gastric)_cancer|chr22*42564716*42564716*C*del*1*1*Colorectal_cancer	chr22*42564715*42564716*COSM1416614*AG*A*1*large_intestine*1
chr22	46931226	GC	G	CELSR1	FRAMESHIFT-DEL	c.1841delG	p.Gly614AlafsTer54	NA	NA	chr22*46931227*46931227*G*del*4*2*Liver_cancer	chr22*46931226*46931227*COSM1416908*GC*G*4*liver
chr14	23862233	G	A	MYH6	MISSENSE	c.3139C>T	p.Arg1047Cys	NA	NA	chr14*23862233*23862233*C*T*1*1*Stomach_(gastric)_cancer	chr14*23862233*23862233*COSM1582268*G*A*1*stomach*1
chr14	24975401	G	A	CMA1	MISSENSE	c.433C>T	p.Arg145Trp	NA	NA	NA	NA
chr14	31355339	T	C	COCH	MISSENSE	c.1298T>C	p.Val433Ala	NA	NA	NA	NA
chr14	50088810	T	C	MGAT2	MISSENSE	c.824T>C	p.Met275Thr	NA	NA	NA	NA
chr14	51387305	C	T	PYGL	MISSENSE	c.809G>A	p.Arg270Gln	NA	NA	chr14*51387305*51387305*G*A*1*1*Brain_cancer	chr14*51387305*51387305*COSM303063*C*T*1*central_nervous_system*1
chr14	59113052	C	T	DACT1	MISSENSE	c.1711C>T	p.Arg571Trp	NA	NA	NA	NA
chr14	64468780	G	A	SYNE2	MISSENSE	c.3767G>A	p.Arg1256His	NA	NA	chr14*64468780*64468780*G*A*1*1*Colorectal_cancer	chr14*64468780*64468780*COSM3419945*G*A*1*large_intestine*1
chr14	64727268	C	T	ESR2	MISSENSE	c.851G>A	p.Arg284His	NA	NA	NA	NA
chr14	64898311	C	T	MTHFD1	MISSENSE	c.1373C>T	p.Ala458Val	NA	NA	NA	NA
chr14	65268057	C	T	SPTB	MISSENSE	c.709G>A	p.Ala237Thr	NA	NA	NA	NA
chr14	67635697	G	A	GPHN	MISSENSE	c.2023G>A	p.Ala675Thr	NA	NA	NA	NA
chr14	68029294	C	T	PLEKHH1	MISSENSE	c.946C>T	p.Arg316Trp	NA	NA	NA	NA
chr14	70252858	C	T	SLC10A1	MISSENSE	c.523G>A	p.Val175Ile	NA	NA	NA	NA
chr14	72939635	G	A	RGS6	MISSENSE	c.592G>A	p.Ala198Thr	NA	NA	NA	NA
chr14	74449813	G	A	ENTPD5	NONSENSE	c.349C>T	p.Arg117Ter	NA	NA	chr14*74449813*74449813*C*T*1*1*Colorectal_cancer	chr14*74449813*74449813*COSM1371031*G*A*1*large_intestine*1
chr14	78709795	G	A	NRXN3	MISSENSE	c.359G>A	p.Arg120His	NA	NA	NA	NA
chr14	80677662	G	A	DIO2	MISSENSE	c.154C>T	p.Arg52Trp	NA	NA	NA	NA
chr14	88938602	C	T	PTPN21	MISSENSE	c.2857G>A	p.Ala953Thr	NA	NA	chr14*88938602*88938602*G*A*1*1*Colorectal_cancer|chr14*88938602*88938602*C*T*1*1*Endometrial_cancer	chr14*88938602*88938602*COSM958457*C*T*1*endometrium*1
chr14	1E+08	C	T	EML1	NONSENSE	c.2047C>T	p.Arg683Ter	NA	NA	NA	NA
chr14	1.02E+08	G	A	DYNC1H1	MISSENSE	c.4133G>A	p.Arg1378Gln	NA	NA	NA	NA
chr14	1.02E+08	C	T	DYNC1H1	MISSENSE	c.6038C>T	p.Ala2013Val	NA	NA	chr14*102476240*102476240*C*T*1*1*Esophageal_cancer	chr14*102476240*102476240*COSM1250827*C*T*1*oesophagus*1
chr14	1.03E+08	A	T	TECPR2	MISSENSE	c.2765A>T	p.Asp922Val	NA	NA	NA	NA
chr14	1.05E+08	G	C	CEP170B	MISSENSE	c.374G>C	p.Gly125Ala	NA	NA	NA	NA
chr14	1.06E+08	C	T	JAG2	MISSENSE	c.584G>A	p.Arg195His	NA	NA	NA	NA

chr14	45645954	CA	C	FANCM	FRAMESHIFT-DEL	c.3998delA	p.Val1336LeufsTer2	NA	NA	chr14*45645954*45645955*ins*A*1*1*Colorectal_cancer|chr14*45645955*45645955*A*del*3*3*Stomach_(gastric)_cancer	chr14*45645954*45645954*COSM1369857*C*CA*1*large_intestine*1|chr14*45645954*45645955*COSM1369858*CA*C*6*large_intestine
chr14	47530770	CT	C	MDGA2	FRAMESHIFT-DEL	c.1206delA	p.Gly403AspfsTer22	NA	NA	chr14*47530771*47530771*T*del*2*2*Stomach_(gastric)_cancer	chr14*47530770*47530771*COSM1369896*CT*C*3*large_intestine*3|chr14*47530770*47530771*COSM1369898*CT*C*3*large_intestine*3|chr14*47530770*47530771*COSM1369897*CT*C*3*large_intestine*3
chr14	47530770	CT	C	MDGA2	FRAMESHIFT-DEL	c.312delA	p.Gly105AspfsTer22	NA	NA	chr14*47530771*47530771*T*del*2*2*Stomach_(gastric)_cancer	chr14*47530770*47530771*COSM1369896*CT*C*3*large_intestine*3|chr14*47530770*47530771*COSM1369898*CT*C*3*large_intestine*3|chr14*47530770*47530771*COSM1369897*CT*C*3*large_intestine*3
chr14	50606660	CA	C	SOS2	FRAMESHIFT-DEL-SS-PRX	c.2784delT	p.Phe928LeufsTer5	NA	NA	chr14*50606661*50606661*T*del*1*1*Stomach_(gastric)_cancer|chr14*50606661*50606661*A*del*2*2*Stomach_(gastric)_cancer	chr14*50606660*50606661*COSM1640096*CA*C*1*stomach*1|chr14*50606660*50606661*COSM1640095*CA*C*1*stomach*1
chr14	50930795	CT	C	MAP4K5	FRAMESHIFT-DEL	c.793delA	p.Arg265AspfsTer8	NA	NA	chr14*50930795*50930795*ins*T*2*2*Stomach_(gastric)_cancer|chr14*50930795*50930796*ins*T*1*1*Melanoma|chr14*50930796*50930796*T*del*1*1*Stomach_(gastric)_cancer|chr14*50930796*50930796*A*del*1*1*Colorectal_cancer	chr14*50930795*50930795*COSM1167791*C*CT*2*skin
chr14	64484386	TA	T	SYNE2	FRAMESHIFT-DEL	c.4962delA	p.Asn1656MetfsTer19	NA	NA	chr14*64484387*64484387*A*del*1*1*Stomach_(gastric)_cancer	chr14*64484386*64484387*COSM1640123*TA*T*1*stomach*1
chr14	75514506	GT	G	MLH3	FRAMESHIFT-DEL	c.1852delA	p.Thr618LeufsTer41	NA	NA	NA	NA

chr14	94517595	GTCT	G	DDX24	INFRAME-DEL	c.2519_2521delAGA	p.Lys840del	NA	NA	chr14*94517595*94517596*ins*TCT*1*1*Stomach_(gastric)_cancer	chr14*94517595*94517598*COSM1371745*GTCT*G*1*large_intestine*1
chr19	613870	G	A	HCN2	MISSENSE	c.1844G>A	p.Arg615Gln	NA	NA	NA	NA
chr19	619647	G	A	POLRMT	MISSENSE	c.3005C>T	p.Thr1002Met	NA	NA	NA	NA
chr19	1046329	C	T	ABCA7	MISSENSE	c.1546C>T	p.Arg516Cys	NA	NA	NA	NA
chr19	1073209	G	A	HMHA1	MISSENSE	c.531G>A	p.Met177Ile	NA	NA	NA	NA
chr19	2199935	C	T	DOT1L	MISSENSE	c.704C>T	p.Thr235Met	NA	NA	NA	NA
chr19	2933602	C	T	ZNF77	MISSENSE	c.1523G>A	p.Arg508His	NA	NA	chr19*2933602*2933602*G*A*1*1*Ovarian_cancer|chr19*2933602*2933602*C*T*1*1*Ovarian_cancer|chr19*2933602*2933602*C*C*1*1*Ovarian_canc	chr19*2933602*2933602*COSM73490*C*T*1*ovary*1
chr19	4154932	A	G	CREB3L3	MISSENSE	c.64A>G	p.Ser22Gly	NA	NA	NA	NA
chr19	6040212	C	T	RFX2	MISSENSE	c.301G>A	p.Ala101Thr	NA	NA	NA	NA
chr19	10738419	C	A	SLC44A2	NONSENSE	c.108C>A	p.Cys36Ter	NA	NA	NA	NA
chr19	11685997	C	T	ACP5	MISSENSE	c.806G>A	p.Arg269Gln	NA	NA	NA	NA
chr19	12984197	G	A	MAST1	MISSENSE	c.3323G>A	p.Ser1108Asn	NA	NA	NA	NA
chr19	13192511	G	A	NFIX	MISSENSE	c.1096G>A	p.Ala366Thr	NA	NA	NA	NA
chr19	15296182	G	A	NOTCH3	MISSENSE	c.2182C>T	p.Arg728Cys	NA	NA	NA	NA
chr19	15538314	C	T	WIZ	MISSENSE	c.560G>A	p.Arg187His	NA	NA	NA	NA
chr19	17212924	C	T	MYO9B	MISSENSE	c.397C>T	p.Arg133Trp	NA	NA	NA	NA
chr19	33482773	C	T	RHPN2	MISSENSE	c.1600G>A	p.Ala534Thr	NA	NA	chr19*33482773*33482773*G*A*1*1*Colon_cancer|chr19*33482773*33482773*C*T*1*1*Colorectal_cancer	chr19*33482773*33482773*COSM187153*C*T*1*large_intestine*1
chr19	33706862	C	A	SLC7A10	MISSENSE	c.169G>T	p.Gly57Cys	NA	NA	NA	NA
chr19	36210426	C	T	KMT2B	MISSENSE	c.419C>T	p.Ala140Val	NA	NA	chr19*36210426*36210426*C*T*1*1*Colorectal_cancer	chr19*36210426*36210426*COSM1392921*C*T*1*large_intestine*1
chr19	36574034	C	T	WDR62	MISSENSE	c.1441C>T	p.Arg481Trp	NA	NA	NA	NA
chr19	36832050	T	G	ZFP14	MISSENSE	c.678A>C	p.Lys226Asn	NA	NA	NA	NA
chr19	39214845	G	A	ACTN4	MISSENSE	c.1741G>A	p.Asp581Asn	NA	NA	NA	NA
chr19	39218649	G	A	ACTN4	MISSENSE	c.2401G>A	p.Val801Met	NA	NA	NA	NA
chr19	40321086	C	T	DYRK1B	MISSENSE	c.301G>A	p.Val101Met	NA	NA	NA	NA
chr19	41888686	T	C	TMEM91	MISSENSE	c.220T>C	p.Ser74Pro	NA	NA	NA	NA
chr19	41931557	C	T	B3GNT8	MISSENSE	c.1127G>A	p.Arg376Gln	NA	NA	NA	NA
chr19	42795851	C	T	CIC	MISSENSE	c.2840C>T	p.Pro947Leu	NA	NA	NA	NA
chr19	42861009	A	G	MEGF8	MISSENSE	c.4505A>G	p.His1502Arg	NA	NA	NA	NA
chr19	44055786	C	T	XRCC1	MISSENSE	c.1136G>A	p.Arg379His	NA	NA	chr19*44055786*44055786*C*T*1*1*Endometrial_cancer	chr19*44055786*44055786*COSM997630*C*T*1*endometrium*1
chr19	45209067	C	T	CEACAM16	MISSENSE	c.869C>T	p.Thr290Met	NA	NA	NA	NA
chr19	46915979	G	T	CCDC8	MISSENSE	c.89C>A	p.Pro30His	NA	NA	NA	NA
chr19	47259005	C	T	FKRP	MISSENSE	c.298C>T	p.Arg100Cys	NA	NA	NA	NA
chr19	47844954	C	T	C5AR2	MISSENSE	c.898C>T	p.Arg300Cys	NA	NA	chr19*47844954*47844954*C*T*1*1*Colorectal_cancer	chr19*47844954*47844954*COSM1394884*C*T*1*large_intestine*1
chr19	48220263	T	C	EHD2	MISSENSE	c.394T>C	p.Phe132Leu	NA	NA	NA	NA
chr19	48229372	G	A	EHD2	MISSENSE	c.806G>A	p.Arg269His	NA	NA	NA	NA
chr19	52272353	G	A	FPR2	MISSENSE	c.442G>A	p.Gly148Arg	NA	NA	NA	NA
chr19	54622003	A	C	PRPF31	MISSENSE	c.228A>C	p.Lys76Asn	NA	NA	NA	NA

chr19	54803070	G	A	LILRA3	MISSENSE	c.607C>T	p.Arg203Cys	NA	NA	chr19*54803070*54803070*G*A*1*1*Endometrial_cancer	chr19*54803070*54803070*COSM1001104*G*A*1*endometrium*1
chr19	55451649	C	T	NLRP7	MISSENSE	c.538G>A	p.Ala180Thr	NA	NA	NA	NA
chr19	613309	A	AC	HCN2	FRAMESHIFT-INS	c.1646_1647insC	p.Asn551GlnfsTer19	NA	NA	NA	NA
chr19	10204185	G	GC	ANGPTL6	FRAMESHIFT-INS	c.1061dupG	p.Arg355ProfsTer8	NA	NA	NA	chr19*10204185*10204186*COSM1389996*GC*G*1*large_intestine*1
chr19	10738663	GA	G	SLC44A2	FRAMESHIFT-DEL	c.229delA	p.Lys77ArgfsTer17	NA	NA	NA	NA
chr19	11097624	G	GC	SMARCA4	FRAMESHIFT-INS	c.804_805insC	p.Gly271ArgfsTer16	NA	NA	NA	chr19*11097624*11097625*COSM30571*GC*G*1*lung*1|chr19*11097624*11097626*COSM30587*GCC*G*1*lung*
chr19	11618820	TG	T	ECSIT	FRAMESHIFT-DEL	c.781delC	p.Gln261SerfsTer5	NA	NA	chr19*11618821*11618821*C*del*2*1*Colorectal_cancer	chr19*11618820*11618821*COSM1390384*TG*T*4*NS
chr19	13008600	TG	T	GCDH	FRAMESHIFT-DEL	c.1167delG	p.Asn392MetfsTer9	UNCERTAIN_SIGNIFICANCE	not_provided	NA	NA
chr19	42730394	AC	A	ZNF526	FRAMESHIFT-DEL	c.1840delC	p.Pro615HisfsTer120	NA	NA	NA	NA
chr19	45910371	TC	T	CD3EAP	FRAMESHIFT-DEL	c.43delC	p.Asn17ThrfsTer19	NA	NA	NA	NA
chr19	46272053	TC	T	SIX5	FRAMESHIFT-DEL	c.49delG	p.Glu17ArgfsTer91	NA	NA	NA	NA
chr19	49458970	TG	T	BAX	FRAMESHIFT-DEL	c.114delG	p.Glu41ArgfsTer19	PATHOGENIC:PATHOGENIC:PATHOGENIC	Carcinoma_of_colon:Carcinoma_of_colon:T-cell_acute_lymphoblastic_leukemia	chr19*49458970*49458970*ins*G*3*2*Stomach_(gastric)_cancer|chr19*49458970*49458971*ins*G*2*1*Breast_cancer|chr19*49458971*49458971*G*del*2*2*Pancreatic_cancer	chr19*49458970*49458970*COSM1481304*T*TG*2*breast*2|chr19*49458970*49458970*COSM1481303*T*TG*2*breast*2|chr19*49458970*49458971*COSM309434*TG*T*16*pancreas
chr8	2021527	G	A	MYOM2	MISSENSE	c.1067G>A	p.Arg356His	NA	NA	NA	NA
chr8	11420564	G	A	BLK	NONSENSE	c.1257G>A	p.Trp419Ter	NA	NA	NA	NA
chr8	21903685	G	A	FGF17	MISSENSE	c.133G>A	p.Asp45Asn	NA	NA	NA	NA
chr8	24775950	T	A	NEFM	MISSENSE	c.2582T>A	p.Ile861Asn	NA	NA	NA	NA
chr8	27255217	G	A	PTK2B	MISSENSE	c.116G>A	p.Arg39His	NA	NA	NA	NA
chr8	29024979	C	T	KIF13B	MISSENSE	c.1069G>A	p.Ala357Thr	NA	NA	NA	NA
chr8	41529890	G	A	ANK1	MISSENSE	c.5201C>T	p.Thr1734Met	NA	NA	chr8*41529890*41529890*C*T*2*2*Pancreatic_cancer	chr8*41529890*41529890*COSM1496904*G*A*2*pancreas
chr8	42742886	A	G	RNF170	MISSENSE	c.122T>C	p.Val41Ala	NA	NA	NA	NA
chr8	56910920	G	A	LYN	MISSENSE	c.1066G>A	p.Ala356Thr	NA	NA	NA	NA
chr8	59409346	A	G	CYP7A1	MISSENSE	c.725T>C	p.Leu242Ser	NA	NA	NA	NA
chr8	71060580	G	A	NCOA2	NONSENSE	c.2533C>T	p.Gln845Ter	NA	NA	NA	NA
chr8	94934711	G	T	PDP1	MISSENSE	c.499G>T	p.Val167Phe	NA	NA	NA	NA
chr8	95538922	A	G	KIAA1429	MISSENSE	c.1550T>C	p.Met517Thr	NA	NA	NA	NA
chr8	1.05E+08	T	C	DPYS	MISSENSE	c.653A>G	p.Glu218Gly	NA	NA	NA	NA
chr8	1.1E+08	T	C	PKHD1L1	MISSENSE	c.5549T>C	p.Val1850Ala	NA	NA	NA	NA
chr8	1.14E+08	A	G	CSMD3	MISSENSE	c.2974T>C	p.Ser992Pro	NA	NA	NA	NA
chr8	1.17E+08	G	T	TRPS1	MISSENSE	c.2461C>A	p.Gln821Lys	NA	NA	NA	NA

chr8	1.34E+08	G	T	TG	MISSENSE	c.1420G>T	p.Gly474Trp	NA	NA	chr8*133899037*133899037*G*C*1*1*Bladder_cancer	NA
chr8	1.41E+08	C	T	TRAPPC9	MISSENSE	c.1528G>A	p.Ala510Thr	NA	NA	NA	NA
chr8	1.45E+08	C	T	NAPRT1	MISSENSE	c.1136G>A	p.Gly379Asp	NA	NA	NA	NA
chr8	1.45E+08	C	T	PLEC	MISSENSE	c.10103G>A	p.Arg3368His	NA	NA	NA	NA
chr8	6302638	CA	C	MCPH1	FRAMESHIFT-DEL	c.1396delA	p.Thr468ProfsTer32	NA	NA	chr8*6302639*6302639*A*del*4*4*Stomach_(gastric)_cancer	chr8*6302638*6302639*COSM312799*CA*C*1*lung*1|chr8*6302638*6302639*COSM179432*CA*C*1*large_intestine*1
chr8	11058233	TG	T	XKR6	FRAMESHIFT-DEL	c.615delC	p.Met206Ter	NA	NA	NA	NA
chr8	17402080	AT	A	SLC7A2	FRAMESHIFT-DEL	c.618delT	p.Phe208LeufsTer9	NA	NA	chr8*17402080*17402080*ins*T*1*1*Stomach_(gastric)_cancer|chr8*17402081*17402081*T*del*1*1*Stomach_(gastric)_cancer	chr8*17402080*17402081*COSM1455837*AT*A*2*large_intestine
chr8	22548711	A	AG	EGR3	FRAMESHIFT-INS	c.438dupC	p.Tyr147LeufsTer47	NA	NA	NA	NA
chr8	28385158	AT	A	FZD3	FRAMESHIFT-DEL	c.882delT	p.Phe296LeufsTer10	NA	NA	chr8*28385158*28385158*ins*T*1*1*Stomach_(gastric)_cancer|chr8*28385158*28385159*ins*T*1*1*Colorectal_cancer	chr8*28385158*28385158*COSM1456490*A*AT*1*large_intestine*1
chr8	30701411	CT	C	TEX15	FRAMESHIFT-DEL	c.5122delA	p.Arg1708GlyfsTer3	NA	NA	chr8*30701411*30701411*ins*T*1*1*Lung_cancer|chr8*30701412*30701412*A*del*1*1*Colorectal_cancer	chr8*30701411*30701412*COSM1456636*CT*C*1*large_intestine*1
chr8	70514025	CT	C	SULF1	FRAMESHIFT-DEL	c.1023delT	p.Phe343LeufsTer7	NA	NA	chr8*70514026*70514026*T*del*1*1*Stomach_(gastric)_cancer	chr8*70514025*70514026*COSM1458028*CT*C*2*large_intestine*2
chr8	70744245	AG	A	SLCO5A1	FRAMESHIFT-DEL	c.663delC	p.Tyr222ThrfsTer6	NA	NA	chr8*70744246*70744246*G*del*1*1*Colorectal_cancer|chr8*70744246*70744246*C*del*1*1*Colon_cancer	chr8*70744245*70744246*COSM195359*AG*A*1*large_intestine*1
chr8	77617336	CA	C	ZFHX4	FRAMESHIFT-DEL	c.1014delA	p.Lys340AsnfsTer15	NA	NA	chr8*77617337*77617337*A*del*2*1*Colorectal_cancer	chr8*77617336*77617337*COSM1458249*CA*C*2*large_intestine*2
chr8	95531562	TA	T	KIAA1429	FRAMESHIFT-DEL	c.2163delT	p.Phe721LeufsTer11	NA	NA	chr8*95531563*95531563*A*del*5*2*Stomach_(gastric)_cancer	chr8*95531562*95531562*COSM1458714*T*TA*1*large_intestine*1|chr8*95531562*95531563*COSM1458715*TA*T*3*large_intestine*3

chr8	1.03E+08	C	CT	UBR5	FRAMESHIFT-INS	c.6360dupA	p.Glu2121ArgfsTer13	NA	NA	chr8*103289348*103289349*ins*T*1*1*Esophageal_cancer	chr8*103289348*103289348*COSM1269667*C*CT*2*NS
chr8	1.44E+08	TC	T	CYP11B2	FRAMESHIFT-DEL	c.546delG	p.Ser183AlafsTer2	NA	NA	chr8*143996511*143996511*G*A*1*1*Lung_cancer	chr8*143996511*143996511*COSM1552039*C*T*1*lung*1
chr8	1.46E+08	T	TG	SLC39A4	FRAMESHIFT-INS	c.1396dupC	p.His466ProfsTer20	NA	NA	NA	NA
chr1	1221591	G	A	SCNN1D	MISSENSE	c.844G>A	p.Ala282Thr	NA	NA	NA	NA
chr1	9030997	G	T	CA6	MISSENSE	c.801G>T	p.Gln267His	NA	NA	NA	NA
chr1	12067291	G	C	MFN2	MISSENSE	c.2054G>C	p.Ser685Thr	NA	NA	NA	NA
chr1	12920018	G	A	PRAMEF2	MISSENSE	c.758G>A	p.Gly253Glu	NA	NA	NA	NA
chr1	16902857	G	A	NBPF1	MISSENSE	c.2024C>T	p.Pro675Leu	NA	NA	chr1*16902857*16902857*G*T*1*1*Lung_cancer	NA
chr1	19992448	C	T	HTR6	MISSENSE	c.202C>T	p.Leu68Phe	NA	NA	NA	NA
chr1	26524819	C	T	CATSPER4	MISSENSE	c.721C>T	p.Pro241Ser	NA	NA	NA	NA
chr1	27107041	A	G	ARID1A	MISSENSE	c.6652A>G	p.Met2218Val	NA	NA	NA	NA
chr1	29314350	C	T	EPB41	MISSENSE	c.401C>T	p.Ala134Val	NA	NA	NA	NA
chr1	34285346	G	A	CSMD2	MISSENSE	c.1172C>T	p.Ala391Val	NA	NA	chr1*34285346*34285346*G*A*1*1*Stomach_(gastric)_cancer	NA
chr1	35250550	G	A	GJB3	MISSENSE	c.187G>A	p.Val63Ile	NA	NA	NA	NA
chr1	41456840	A	C	CTPS1	MISSENSE	c.559A>C	p.Ser187Arg	NA	NA	NA	NA
chr1	46736381	C	T	RAD54L	NONSENSE	c.1093C>T	p.Arg365Ter	NA	NA	NA	NA
chr1	57397545	C	T	C8B	MISSENSE	c.1559G>A	p.Arg520His	NA	NA	chr1*57397545*57397545*G*T*1*1*Breast_cancer	chr1*57397545*57397545*COSM212917*C*A*1*breast*1
chr1	57425814	A	T	C8B	MISSENSE	c.128T>A	p.Val43Asp	NA	NA	NA	NA
chr1	65332823	C	T	JAK1	MISSENSE	c.716G>A	p.Arg239Gln	NA	NA	NA	NA
chr1	65849935	C	A	DNAJC6	NONSENSE	c.726C>A	p.Tyr242Ter	NA	NA	NA	NA
chr1	77510030	G	A	ST6GALNAC5	MISSENSE	c.403G>A	p.Val135Met	NA	NA	chr1*77510030*77510030*G*A*1*1*Lung_cancer	chr1*77510030*77510030*COSM536348*G*A*1*lung*1
chr1	91727847	C	A	HFM1	MISSENSE	c.4189G>T	p.Val1397Leu	NA	NA	NA	chr1*91727847*91727847*COSM1344845*C*CT*1*large_intestine*1
chr1	91846534	T	A	HFM1	NONSENSE	c.808A>T	p.Lys270Ter	NA	NA	NA	NA
chr1	1.1E+08	C	T	CELSR2	MISSENSE	c.5743C>T	p.Pro1915Ser	NA	NA	NA	NA
chr1	1.11E+08	A	G	SLC6A17	MISSENSE	c.757A>G	p.Met253Val	NA	NA	NA	NA
chr1	1.12E+08	C	T	OVGP1	MISSENSE	c.955G>A	p.Val319Ile	NA	NA	NA	NA
chr1	1.12E+08	C	T	KCND3	MISSENSE	c.1703G>A	p.Arg568His	NA	NA	chr1*112319711*112319711*C*T*1*1*Myeloma	chr1*112319711*112319711*COSM329434*C*T*1*haematopoietic_and_lymphoid_tissue*1
chr1	1.13E+08	G	A	KCND3	MISSENSE	c.740C>T	p.Ala247Val	NA	NA	NA	NA
chr1	1.16E+08	G	T	VANGL1	MISSENSE	c.121G>T	p.Gly41Trp	NA	NA	NA	NA
chr1	1.21E+08	C	T	NOTCH2	MISSENSE	c.1462G>A	p.Gly488Ser	NA	NA	NA	NA
chr1	1.51E+08	C	A	ADAMTSL4	MISSENSE	c.788C>A	p.Pro263His	NA	NA	NA	NA
chr1	1.52E+08	C	T	FLG	MISSENSE	c.1076G>A	p.Arg359His	NA	NA	NA	NA
chr1	1.56E+08	G	A	IQGAP3	MISSENSE	c.4315C>T	p.Arg1439Cys	NA	NA	NA	NA
chr1	1.57E+08	G	A	IQGAP3	MISSENSE	c.782C>T	p.Ala261Val	NA	NA	chr1*156532942*156532942*C*T*1*1*Colorectal_cancer	chr1*156532942*156532942*COSM1334867*G*A*1*large_intestine*1
chr1	1.57E+08	T	G	ARHGEF11	MISSENSE	c.1586A>C	p.Glu529Ala	NA	NA	NA	NA

chr1	1.58E+08	C	T	FCRL3	MISSENSE	c.1424G>A	p.Arg475His	NA	NA	chr1*157660311*157660311*G*A*1*1*Colon_cancer|chr1*157660311*157660311*C*T*1*1*Colorectal_cancer	chr1*157660311*157660311*COSM272689*C*T*1*large_intestine*1
chr1	1.6E+08	G	A	CCDC19	MISSENSE	c.1229C>T	p.Ala410Val	NA	NA	NA	NA
chr1	1.61E+08	C	T	B4GALT3	MISSENSE	c.452G>A	p.Arg151His	NA	NA	NA	NA
chr1	1.65E+08	T	C	PBX1	MISSENSE	c.1043T>C	p.Met348Thr	NA	NA	NA	NA
chr1	1.69E+08	G	A	F5	MISSENSE	c.6322C>T	p.Arg2108Cys	NA	NA	NA	NA
chr1	1.7E+08	G	A	SELP	MISSENSE	c.2345C>T	p.Thr782Met	NA	NA	chr1*169562905*169562905*G*A*1*1*Breast_cancer|chr1*169562905*169562905*C*A*1*1*Head_and_neck_cancer	chr1*169562905*169562905*COSM126069*G*T*1*upper_aerodigestive_tract*1|chr1*169562905*169562905*COSM1472958*G*A*1*breast*1
chr1	1.72E+08	G	A	PRRC2C	MISSENSE	c.3947G>A	p.Arg1316Lys	NA	NA	NA	NA
chr1	1.72E+08	C	T	PRRC2C	MISSENSE	c.7718C>T	p.Ala2573Val	NA	NA	NA	NA
chr1	1.74E+08	G	A	SERPINC1	MISSENSE	c.880C>T	p.Arg294Cys	NA	NA	chr1*173878963*173878963*G*A*1*1*Endometrial_cancer	chr1*173878963*173878963*COSM899813*G*A*1*endometrium*1
chr1	1.86E+08	C	T	HMCN1	MISSENSE	c.9545C>T	p.Thr3182Met	NA	NA	NA	NA
chr1	2.07E+08	C	T	PIGR	MISSENSE	c.1646G>A	p.Gly549Asp	NA	NA	NA	NA
chr1	2.07E+08	G	T	PIGR	MISSENSE	c.1108C>A	p.Leu370Ile	NA	NA	NA	NA
chr1	2.2E+08	C	T	RAB3GAP2	MISSENSE	c.37G>A	p.Asp13Asn	NA	NA	NA	NA
chr1	2.23E+08	G	A	DISP1	MISSENSE	c.3367G>A	p.Ala1123Thr	NA	NA	NA	NA
chr1	2.28E+08	G	A	OBSCN	MISSENSE	c.9571G>A	p.Glu3191Lys	NA	NA	NA	NA
chr1	2.28E+08	C	T	OBSCN	MISSENSE	c.9943C>T	p.Arg3315Cys	NA	NA	NA	NA
chr1	2.28E+08	G	A	OBSCN	MISSENSE	c.14129G>A	p.Arg4710Gln	NA	NA	NA	NA
chr1	2.29E+08	C	T	HIST3H3	MISSENSE	c.397G>A	p.Gly133Arg	NA	NA	NA	NA
chr1	2.36E+08	G	A	NID1	MISSENSE	c.3353C>T	p.Ala1118Val	NA	NA	chr1*236143828*236143828*C*A*1*1*Lung_cancer	chr1*236143828*236143828*COSM341192*G*T*1*lung*1
chr1	2.36E+08	G	A	NID1	MISSENSE	c.2612C>T	p.Pro871Leu	NA	NA	NA	NA
chr1	2.38E+08	G	A	RYR2	MISSENSE	c.2374G>A	p.Val792Ile	NA	NA	chr1*237664181*237664181*G*A*1*1*Prostate_cancer	chr1*237664181*237664181*COSM3671676*G*A*1*prostate*1|chr1*237664181*237664181*COSM3671677*G*A*1*prostate*1
chr1	2.38E+08	G	A	RYR2	MISSENSE	c.5149G>A	p.Ala1717Thr	NA	NA	chr1*237777577*237777577*G*T*1*1*Lung_cancer	chr1*237777577*237777577*COSM679911*G*T*1*lung*1
chr1	2.38E+08	G	A	RYR2	MISSENSE	c.13357G>A	p.Ala4453Thr	NA	NA	chr1*237951316*237951316*G*T*1*1*Lung_cancer	NA
chr1	2.4E+08	G	A	CHRM3	MISSENSE	c.214G>A	p.Ala72Thr	NA	NA	NA	NA
chr1	2.42E+08	G	T	EXO1	MISSENSE	c.2487G>T	p.Glu829Asp	NA	NA	NA	NA
chr1	6173035	TCTC	T	CHD5	INFRAME-DEL	c.4933_4935delGAG	p.Glu1645del	NA	NA	NA	NA
chr1	16474976	A	AC	EPHA2	FRAMESHIFT-INS	c.719dupG	p.Glu241Ter	NA	NA	NA	chr1*16474976*16474977*COSM272640*AC*A*1*large_intestine*1

chr1	27099102	TC	T	ARID1A	FRAMESHIFT-DEL	c.3519delC	p.Pro1175HisfsTer5	NA	NA	chr1*27099102*27099102*ins*C*1*1*Stomach_(gastric)_cancer	chr1*27099102*27099103*COSM51476*TC*T*1*ovary*1
chr1	27876438	TC	T	AHDC1	FRAMESHIFT-DEL	c.2188delG	p.Glu730ArgfsTer2	NA	NA	NA	NA
chr1	36636586	AC	A	MAP7D1	FRAMESHIFT-DEL	c.62delC	p.Pro23GlnfsTer33	NA	NA	chr1*36636586*36636587*ins*C*1*1*Colorectal_cancer|chr1*36636587*36636587*C*del*2*1*Colorectal_cancer	chr1*36636586*36636586*COSM1342263*A*AC*1*large_intestine*1|chr1*36636586*36636587*COSM1342264*AC*A*2*large_intestine*2
chr1	44463540	TC	T	SLC6A9	FRAMESHIFT-DEL	c.1912delG	p.Asp638ThrfsTer7	NA	NA	NA	NA
chr1	46521524	AT	A	PIK3R3	FRAMESHIFT-DEL	c.883delA	p.Met295Ter	NA	NA	chr1*46521524*46521524*A*G*1*1*Lung_cancer|chr1*46521525*46521525*T*del*1*1*Stomach_(gastric)_cancer	chr1*46521524*46521524*COSM535457*A*G*1*lung*1|chr1*46521524*46521525*COSM1343109*AT*A*1*large_intestine*1
chr1	82456670	TA	T	LPHN2	FRAMESHIFT-DEL	c.4054delA	p.Ser1353AlafsTer26	NA	NA	NA	NA
chr1	1.01E+08	TAA	T	SASS6	FRAMESHIFT-DEL	c.169_170delTT	p.Leu57IlefsTer2	NA	NA	chr1*100588801*100588801*ins*A*1*1*Stomach_(gastric)_cancer|chr1*100588802*100588802*T*del*1*1*Stomach_(gastric)_cancer|chr1*100588802*100588802*A*del*1*1*Stomach_(gastric)_cancer	chr1*100588801*100588802*COSM1331978*TA*T*8*liver
chr1	1.47E+08	A	AC	BCL9	FRAMESHIFT-INS	c.1539_1540insC	p.Pro517SerfsTer7	NA	NA	chr1*147091500*147091500*ins*C*2*1*Stomach_(gastric)_cancer|chr1*147091500*147091501*ins*C*3*1*Colorectal_cancer	chr1*147091500*147091500*COSM1333664*A*AC*3*large_intestine*3|chr1*147091500*147091501*COSM391451*AC*A*5*large_intestine
chr1	1.55E+08	CT	C	ASH1L	FRAMESHIFT-DEL	c.3665delA	p.Lys1222ArgfsTer11	NA	NA	chr1*155448995*155448995*C*G*1*1*Head_and_neck_cancer|chr1*155448996*155448996*A*del*1*1*Stomach_(gastric)_cancer	chr1*155448995*155448996*COSM1334660*CT*C*3*large_intestine

chr1	1.56E+08	CAGAG	C	GON4L	FRAMESHIFT-DEL	c.3771_3774delCTCT	p.Ser1258LeufsTer15	NA	NA	chr1*155735493*155735493*C*G*1*1*Lung_cancer	chr1*155735493*155735493*COSM344844*G*C*1*lung*1|chr1*155735493*155735493*COSM344843*G*C*1*lung*1|chr1*155735493*155735493*COSM344842*G*C*1*lung*1
chr1	1.57E+08	TC	T	PEAR1	FRAMESHIFT-DEL	c.2860delC	p.Arg956GlyfsTer30	NA	NA	chr1*156883790*156883790*C*del*1*1*Ovarian_carcinoma	chr1*156883789*156883790*COSM253059*TC*T*2*ovary
chr1	1.6E+08	TG	T	PEX19	FRAMESHIFT-DEL	c.711delC	p.Thr238GlnfsTer24	NA	NA	NA	NA
chr1	1.6E+08	TG	T	DCAF8	FRAMESHIFT-DEL	c.270delC	p.Thr91GlnfsTer24	NA	NA	NA	NA
chr1	1.69E+08	TA	T	SLC19A2	FRAMESHIFT-DEL	c.623delT	p.Leu208TyrfsTer20	NA	NA	NA	NA
chr1	1.73E+08	CAG	C	SUCO	FRAMESHIFT-DEL	c.645_646delAG	p.Glu216IlefsTer2	NA	NA	NA	NA
chr1	2.01E+08	AT	A	DDX59	FRAMESHIFT-DEL	c.1730delA	p.Asn577IlefsTer6	NA	NA	NA	chr1*200594041*200594042*COSM252990*AT*A*4*ovary
chr1	2.04E+08	A	AG	PLEKHA6	FRAMESHIFT-INS	c.2110dupC	p.Leu704ProfsTer27	NA	NA	NA	NA
chr1	2.05E+08	TG	T	CNTN2	FRAMESHIFT-DEL	c.273delG	p.Gly93AlafsTer6	NA	NA	chr1*205027366*205027366*G*del*1*1*Colorectal_cancer	chr1*205027365*205027366*COSM1338142*TG*T*1*large_intestine*1
chr1	2.08E+08	TC	T	CR1	FRAMESHIFT-DEL	c.6094delC	p.Pro2033LeufsTer44	NA	NA	NA	NA
chr1	2.24E+08	AG	A	TP53BP2	FRAMESHIFT-DEL	c.275delC	p.Pro92LeufsTer6	NA	NA	chr1*224001956*224001956*C*del*1*1*Colorectal_cancer	chr1*224001955*224001956*COSM1339148*AG*A*1*large_intestine*1
chr1	2.27E+08	CACAA	C	ADCK3	FRAMESHIFT-DEL	c.578_581delACAA	p.Lys194ArgfsTer14	NA	NA	NA	NA
chr1	2.31E+08	T	TG	AGT	FRAMESHIFT-INS	c.407dupC	p.Thr137AsnfsTer20	NA	NA	NA	NA
chr1	2.31E+08	T	TA	SPRTN	FRAMESHIFT-INS	c.930_931insA	p.Asn312LysfsTer10	NA	NA	NA	NA
chr11	1025235	G	A	MUC6	MISSENSE	c.2932C>T	p.Leu978Phe	NA	NA	NA	NA
chr11	1084808	A	G	MUC2	MISSENSE	c.2603A>G	p.Tyr868Cys	NA	NA	NA	NA
chr11	1263467	C	T	MUC5B	MISSENSE	c.5357C>T	p.Pro1786Leu	NA	NA	NA	NA
chr11	1263619	G	A	MUC5B	MISSENSE	c.5509G>A	p.Glu1837Lys	NA	NA	NA	NA
chr11	2424419	C	A	TSSC4	MISSENSE	c.556C>A	p.Leu186Met	NA	NA	NA	NA
chr11	3721877	C	A	NUP98	MISSENSE	c.3705G>T	p.Trp1235Cys	NA	NA	NA	NA
chr11	3744446	T	G	NUP98	MISSENSE	c.2087A>C	p.Glu696Ala	NA	NA	NA	NA
chr11	6292495	C	T	CCKBR	MISSENSE	c.1066C>T	p.Arg356Cys	NA	NA	chr11*6292495*6292495*C*T*3*3*Stomach_(gastric)_cancer	chr11*6292495*6292495*COSM1298345*C*T*2*large_intestine
chr11	6416832	C	T	APBB1	MISSENSE	c.2059G>A	p.Val687Ile	NA	NA	NA	NA
chr11	6662141	C	T	DCHS1	MISSENSE	c.704G>A	p.Arg235Gln	NA	NA	NA	chr11*6662141*6662141*COSM415791*C*T*1*urinary_tract*1|chr11*6662141*6662142*COSM1356293*CG*C*1*large_intestine*1
chr11	10796818	C	T	CTR9	NONSENSE	c.2950C>T	p.Arg984Ter	NA	NA	NA	NA

chr11	12904622	C	T	TEAD1	MISSENSE	c.649C>T	p.Arg217Cys	NA	NA	chr11*12904622*12904622*C*T*1*1*Lung_cancer	chr11*12904622*12904622*COSM687281*C*T*1*lung*1|chr11*12904622*12904622*COSM1646403*C*T*1*lung*1
chr11	14856534	A	G	PDE3B	MISSENSE	c.2213A>G	p.Asn738Ser	NA	NA	NA	NA
chr11	17419239	C	T	ABCC8	MISSENSE	c.3859G>A	p.Ala1287Thr	NA	NA	NA	NA
chr11	17428640	G	A	ABCC8	MISSENSE	c.2957C>T	p.Ser986Leu	NA	NA	NA	NA
chr11	18736130	C	T	IGSF22	MISSENSE	c.1573G>A	p.Ala525Thr	NA	NA	chr11*18736130*18736130*C*T*3*3*Renal_cancer	chr11*18736130*18736130*COSM466642*C*T*1*endometrium*1|chr11*18736130*18736130*COSM466641*C*T*1*endometrium*1
chr11	22271895	T	A	ANO5	MISSENSE	c.991T>A	p.Ser331Thr	NA	NA	NA	NA
chr11	44286499	C	A	ALX4	NONSENSE	c.1141G>T	p.Glu381Ter	NA	NA	chr11*44286499*44286499*C*T*2*2*Endometrial_cancer	chr11*44286499*44286499*COSM926981*C*T*1*endometrium*1
chr11	45827440	G	A	SLC35C1	MISSENSE	c.88G>A	p.Gly30Arg	NA	NA	NA	NA
chr11	47305749	A	G	MADD	MISSENSE	c.1883A>G	p.Tyr628Cys	NA	NA	NA	NA
chr11	47460454	C	T	RAPSN	MISSENSE	c.995G>A	p.Ser332Asn	NA	NA	NA	NA
chr11	57569620	C	T	CTNND1	NONSENSE	c.1372C>T	p.Arg458Ter	NA	NA	chr11*57569620*57569620*C*T*1*1*Pancreatic_cancer	chr11*57569620*57569620*COSM3375851*C*T*1*pancreas*1|chr11*57569620*57569620*COSM3375852*C*T*1*pancreas*1
chr11	61160780	G	A	TMEM216	MISSENSE	c.112G>A	p.Glu38Lys	NA	NA	NA	NA
chr11	64427951	C	T	NRXN2	MISSENSE	c.2242G>A	p.Ala748Thr	NA	NA	NA	NA
chr11	65413719	G	A	SIPA1	MISSENSE	c.1291G>A	p.Asp431Asn	NA	NA	NA	NA
chr11	65792857	G	A	CATSPER1	MISSENSE	c.994C>T	p.Arg332Trp	NA	NA	chr11*65792857*65792857*C*T*2*2*Colorectal_cancer	chr11*65792857*65792857*COSM1199695*G*A*1*large_intestine*1
chr11	66472714	C	T	SPTBN2	MISSENSE	c.2033G>A	p.Arg678His	NA	NA	NA	NA
chr11	67223662	C	T	CABP4	MISSENSE	c.370C>T	p.Arg124Cys	PATHOGENIC	Congenital_stationary_night_blindness_type_2B	NA	NA
chr11	68115597	C	T	LRP5	MISSENSE	c.374C>T	p.Thr125Met	NA	NA	NA	NA
chr11	71724707	G	T	NUMA1	MISSENSE	c.3842C>A	p.Ala1281Asp	NA	NA	NA	NA
chr11	72406885	G	T	ARAP1	MISSENSE	c.3298C>A	p.Gln1100Lys	NA	NA	NA	NA
chr11	75113457	G	A	RPS3	MISSENSE	c.365G>A	p.Arg122His	NA	NA	NA	NA
chr11	76750806	G	A	B3GNT6	MISSENSE	c.211G>A	p.Val71Met	NA	NA	NA	NA
chr11	88780628	C	T	GRM5	MISSENSE	c.413G>A	p.Arg138His	NA	NA	NA	NA
chr11	88780877	C	A	GRM5	MISSENSE	c.164G>T	p.Arg55Met	NA	NA	NA	NA
chr11	1.02E+08	A	G	ANGPTL5	MISSENSE	c.701T>C	p.Val234Ala	NA	NA	NA	NA
chr11	1.03E+08	A	C	MMP3	MISSENSE	c.805T>G	p.Ser269Ala	NA	NA	NA	NA
chr11	1.15E+08	T	C	CADM1	MISSENSE	c.604A>G	p.Thr202Ala	NA	NA	NA	NA
chr11	1.17E+08	A	T	CEP164	MISSENSE	c.2111A>T	p.Glu704Val	NA	NA	NA	NA
chr11	1.18E+08	A	G	IL10RA	MISSENSE	c.122A>G	p.His41Arg	NA	NA	NA	NA
chr11	1.19E+08	G	A	NLRX1	MISSENSE	c.974G>A	p.Arg325His	NA	NA	NA	NA
chr11	1.19E+08	G	A	NLRX1	MISSENSE	c.1694G>A	p.Arg565His	NA	NA	NA	NA
chr11	1.21E+08	G	A	GRIK4	MISSENSE	c.661G>A	p.Ala221Thr	NA	NA	NA	NA
chr11	1.26E+08	G	A	CDON	MISSENSE	c.875C>T	p.Ala292Val	NA	NA	NA	NA

chr11	1.26E+08	G	A	CDON	MISSENSE	c.457C>T	p.Arg153Cys	NA	NA	chr11*125889553*125889553*G*A*1*1*Colorectal_cancer|chr11*125889553*125889553*C*T*1*1*Rectal_cancer	chr11*125889553*125889553*COSM290779*G*A*1*large_intestine*1
chr11	1.29E+08	T	G	KCNJ5	MISSENSE	c.454T>G	p.Tyr152Asp	NA	NA	NA	NA
chr11	5687266	A	AT	TRIM5	FRAMESHIFT-INS	c.812dupA	p.Asn271LysfsTer10	NA	NA	NA	NA
chr11	6643252	TG	T	DCHS1	FRAMESHIFT-DEL	c.9654delC	p.Lys3219SerfsTer176	NA	NA	chr11*6643252*6643252*ins*G*1*1*Stomach_(gastric)_cancer	chr11*6643252*6643253*COSM1356213*TG*T*1*large_intestine*1
chr11	30033562	CA	C	KCNA4	FRAMESHIFT-DEL	c.663delT	p.Phe221LeufsTer21	NA	NA	chr11*30033563*30033563*T*del*1*1*Colon_cancer|chr11*30033563*30033563*A*del*1*1*Colorectal_cancer	chr11*30033562*30033562*COSM1353397*C*CA*1*large_intestine*1|chr11*30033562*30033563*COSM293643*CA*C*1*large_intestine*1
chr11	34482804	CT	C	CAT	FRAMESHIFT-DEL	c.1064delT	p.Phe356LeufsTer20	NA	NA	NA	NA
chr11	35513669	AC	A	PAMR1	FRAMESHIFT-DEL	c.302delG	p.Gly101ValfsTer8	NA	NA	chr11*35513669*35513669*ins*C*1*1*Head_and_neck_cancer|chr11*35513669*35513670*ins*G*1*1*Liver_cancer|chr11*35513670*35513670*ins*G*1*1*Stomach_(gastric)_cancer	chr11*35513669*35513669*COSM1353686*A*AC*3*liver
chr11	36614222	CT	C	RAG2	FRAMESHIFT-DEL	c.1496delA	p.Lys499SerfsTer4	NA	NA	chr11*36614223*36614223*T*del*1*1*Stomach_(gastric)_cancer|chr11*36614223*36614223*A*del*1*1*Lung_cancer	chr11*36614222*36614223*COSM314668*CT*C*3*lung
chr11	62749333	CA	C	SLC22A6	FRAMESHIFT-DEL	c.777delT	p.Phe259LeufsTer27	NA	NA	chr11*62749334*62749334*T*del*1*1*Colorectal_cancer	chr11*62749333*62749334*COSM1355506*CA*C*1*large_intestine*1
chr11	64074719	C	CA	ESRRA	FRAMESHIFT-INS	c.68_69insA	p.Ser26PhefsTer72	NA	NA	NA	NA
chr11	68133123	G	GC	LRP5	FRAMESHIFT-INS	c.968_969insC	p.Thr325HisfsTer13	NA	NA	NA	NA
chr11	68696802	GC	G	IGHMBP2	FRAMESHIFT-DEL	c.1213delC	p.Thr407ProfsTer16	NA	NA	NA	NA
chr11	72424246	GC	G	ARAP1	FRAMESHIFT-DEL	c.721delG	p.Ala241ProfsTer5	NA	NA	NA	NA
chr11	94189472	AT	A	MRE11A	FRAMESHIFT-DEL	c.1532delA	p.Asn511IlefsTer13	NA	NA	chr11*94189473*94189473*A*del*1*1*Stomach_(gastric)_cancer	chr11*94189472*94189473*COSM1357925*AT*A*2*large_intestine

chr11	1.08E+08	C	CT	ATM	FRAMESHIFT-INS	c.633_634insT	p.Ser214PhefsTer40	LIKELY_PATHOGENIC	Ataxia-telangiectasia_syndrome	NA	chr11*108114816*108114817*COSM1350740*CT*C*1*large_intestine*1|chr11*108114816*108114817*COSM1350741*CT*C*1*large_intestine*1
chr11	1.12E+08	GA	G	DLAT	FRAMESHIFT-DEL	c.716delA	p.Val242TrpfsTer5	NA	NA	chr11*111904183*111904183*A*del*4*3*Stomach_(gastric)_cancer	chr11*111904182*111904183*COSM1192764*GA*G*2*large_intestine*2
chr11	1.17E+08	GC	G	BACE1	FRAMESHIFT-DEL	c.107delG	p.Gly36AlafsTer32	NA	NA	NA	NA
chr11	1.19E+08	GC	G	BCL9L	FRAMESHIFT-DEL	c.1442delG	p.Gly481AlafsTer21	NA	NA	chr11*118773010*118773010*G*del*1*1*Colorectal_cancer	chr11*118773009*118773010*COSM1351741*GC*G*1*large_intestine*1|chr11*118773009*118773010*COSM1351740*GC*G*1*large_intestine*1
chr6	12121526	G	A	HIVEP1	MISSENSE	c.1498G>A	p.Ala500Thr	NA	NA	NA	NA
chr6	26459820	G	A	BTN2A1	MISSENSE	c.194G>A	p.Arg65Gln	NA	NA	NA	NA
chr6	26506947	C	T	BTN1A1	MISSENSE	c.746C>T	p.Ala249Val	NA	NA	chr6*26506947*26506947*C*A*1*1*Endometrial_cancer	chr6*26506947*26506947*COSM1076829*C*A*1*endometrium*1
chr6	29910755	C	T	HLA-A	NONSENSE	c.295C>T	p.Arg99Ter	NA	NA	NA	NA
chr6	31084817	G	T	CDSN	MISSENSE	c.575C>A	p.Pro192His	NA	NA	NA	NA
chr6	31611918	G	A	BAG6	MISSENSE	c.1519C>T	p.Pro507Ser	NA	NA	NA	NA
chr6	35423604	C	T	FANCE	MISSENSE	c.329C>T	p.Pro110Leu	NA	NA	NA	NA
chr6	38825323	G	A	DNAH8	NONSENSE	c.5112G>A	p.Trp1704Ter	NA	NA	NA	NA
chr6	38890861	C	T	DNAH8	MISSENSE	c.10039C>T	p.Arg3347Trp	NA	NA	NA	NA
chr6	42236950	C	T	TRERF1	MISSENSE	c.379G>A	p.Ala127Thr	NA	NA	NA	NA
chr6	44371715	G	A	CDC5L	MISSENSE	c.709G>A	p.Ala237Thr	NA	NA	chr6*44371715*44371715*G*A*1*1*Endometrial_cancer	chr6*44371715*44371715*COSM1079786*G*A*1*endometrium*1
chr6	49412367	T	A	MUT	MISSENSE	c.1661A>T	p.Asp554Val	NA	NA	NA	NA
chr6	56418360	T	G	DST	MISSENSE	c.7361A>C	p.Lys2454Thr	NA	NA	NA	NA
chr6	71011709	C	T	COL9A1	MISSENSE	c.83G>A	p.Arg28His	NA	NA	chr6*71011709*71011709*G*A*1*1*Colon_cancer|chr6*71011709*71011709*C*T*1*1*Colorectal_cancer	chr6*71011709*71011709*COSM287366*C*T*2*large_intestine*2
chr6	72892085	C	T	RIMS1	MISSENSE	c.911C>T	p.Ala304Val	NA	NA	NA	NA
chr6	87971031	C	T	ZNF292	MISSENSE	c.7684C>T	p.Arg2562Cys	NA	NA	chr6*87971031*87971031*C*T*1*1*Endometrial_cancer	chr6*87971031*87971031*COSM1082306*C*T*1*endometrium*1|chr6*87971031*87971031*COSM1082307*C*T*1*endometrium*1
chr6	88853777	T	G	CNR1	MISSENSE	c.1217A>C	p.His406Pro	NA	NA	NA	NA
chr6	1.02E+08	A	T	GRIK2	MISSENSE	c.2108A>T	p.Asp703Val	NA	NA	NA	NA

chr6	1.28E+08	C	A	PTPRK	MISSENSE-SS-PRX	c.3405G>T	p.Glu1135Asp	NA	NA	NA	NA
chr6	1.28E+08	C	T	PTPRK	MISSENSE	c.2072G>A	p.Arg691Gln	NA	NA	NA	NA
chr6	1.3E+08	G	A	ARHGAP18	MISSENSE-SS-PRX	c.115C>T	p.Arg39Cys	NA	NA	NA	NA
chr6	1.39E+08	C	T	KIAA1244	MISSENSE	c.416C>T	p.Ala139Val	NA	NA	chr6*138550985*138550985*C*A*1*1*Head_and_neck_cancer	NA
chr6	1.53E+08	T	A	SYNE1	MISSENSE	c.19230A>T	p.Glu6410Asp	NA	NA	NA	NA
chr6	1.53E+08	T	A	SYNE1	MISSENSE	c.19129A>T	p.Ile6377Leu	NA	NA	NA	NA
chr6	1.56E+08	C	A	NOX3	MISSENSE	c.313G>T	p.Val105Phe	NA	NA	NA	NA
chr6	1.56E+08	C	A	NOX3	MISSENSE	c.299G>T	p.Arg100Ile	NA	NA	NA	NA
chr6	1.56E+08	C	A	NOX3	MISSENSE	c.263G>T	p.Arg88Ile	NA	NA	NA	NA
chr6	1.58E+08	C	T	ARID1B	MISSENSE	c.4726C>T	p.Pro1576Ser	NA	NA	chr6*157522454*157522454*C*T*1*1*Endometrial_carcinoma	chr6*157522454*157522454*COSM1075355*C*T*1*endometrium*1|chr6*157522454*157522454*COSM1154612*C*T*1*endometrium*1
chr6	1.61E+08	G	A	LPA	MISSENSE	c.5111C>T	p.Pro1704Leu	NA	NA	NA	NA
chr6	1.64E+08	G	A	PACRG	MISSENSE	c.746G>A	p.Gly249Asp	NA	NA	NA	NA
chr6	1.68E+08	G	T	UNC93A	MISSENSE	c.250G>T	p.Gly84Cys	NA	NA	chr6*167708167*167708167*G*T*1*1*Lung_cancer	chr6*167708167*167708167*COSM741155*G*T*1*lung*1
chr6	1.7E+08	C	T	THBS2	MISSENSE	c.151G>A	p.Gly51Ser	NA	NA	chr6*169648970*169648970*C*A*1*1*Stomach_(gastric)_cancer	NA
chr6	1.71E+08	G	A	DLL1	MISSENSE	c.1174C>T	p.Arg392Cys	NA	NA	NA	NA
chr6	7580880	TA	T	DSP	FRAMESHIFT-DEL	c.4458delA	p.Lys1487AsnfsTer3	NA	NA	NA	NA
chr6	10402828	A	AT	TFAP2A	FRAMESHIFT-INS	c.779dupA	p.Asn260LysfsTer23	NA	NA	NA	NA
chr6	15497225	T	TC	JARID2	FRAMESHIFT-INS	c.1769_1770insC	p.Pro593SerfsTer12	NA	NA	NA	chr6*15497225*15497226*COSM1441795*TC*T*1*large_intestine*1
chr6	29911899	A	AC	HLA-A	FRAMESHIFT-INS-SS-PRX	c.620_621insC	p.Lys210GlnfsTer11	NA	NA	chr6*29911899*29911899*ins*C*1*1*Stomach_(gastric)_cancer	chr6*29911899*29911900*COSM1442957*AC*A*3*large_intestine*3
chr6	31608570	TG	T	BAG6	FRAMESHIFT-DEL-SS-PRX	c.2842delC	p.Gln948SerfsTer85	NA	NA	chr6*31608571*31608571*C*del*2*1*Colorectal_cancer	chr6*31608570*31608571*COSM1443308*TG*T*2*large_intestine*2
chr6	33263964	GC	G	RGL2	FRAMESHIFT-DEL	c.608delG	p.Gly203AlafsTer49	NA	NA	chr6*33263965*33263965*C*del*1*1*Stomach_(gastric)_cancer	chr6*33263964*33263964*COSM1443766*G*GC*5*large_intestine*5|chr6*33263964*33263965*COSM1443767*GC*G*2*large_intestine*2
chr6	41037820	AAT	A	OARD1	FRAMESHIFT-DEL	c.236_237delAT	p.Tyr79LeufsTer15	NA	NA	NA	NA
chr6	64395351	G	GA	PHF3	FRAMESHIFT-INS	c.1728_1729insA	p.Thr579AsnfsTer20	NA	NA	chr6*64395351*64395351*ins*A*1*1*Stomach_(gastric)_cancer	NA

chr6	73902406	GA	G	KCNQ5	FRAMESHIFT-DEL	c.1886delA	p.Lys630AsnfsTer6	NA	NA	NA	NA
chr6	74477920	TA	T	CD109	FRAMESHIFT-DEL	c.1641delA	p.Ile549PhefsTer11	NA	NA	NA	NA
chr6	75866087	TG	T	COL12A1	FRAMESHIFT-DEL	c.3135delC	p.Thr1046GlnfsTer7	NA	NA	chr6*75866088*75866088*C*del*2*2*Colorectal_cancer	chr6*75866087*75866088*COSM196339*TG*T*1*large_intestine*1
chr6	83839079	GA	G	DOPEY1	FRAMESHIFT-DEL	c.2194delA	p.Asn733ThrfsTer2	NA	NA	NA	NA
chr6	87928357	AT	A	ZNF292	FRAMESHIFT-DEL	c.447delT	p.Leu151Ter	NA	NA	chr6*87928358*87928358*T*del*2*2*Colorectal_cancer	chr6*87928357*87928358*COSM295111*AT*A*1*large_intestine*1|chr6*87928357*87928358*COSM295110*AT*A*1*large_intestine*1
chr6	1.07E+08	CT	C	ATG5	FRAMESHIFT-DEL	c.704delA	p.Lys235ArgfsTer4	NA	NA	chr6*106634538*106634538*ins*T*1*1*Stomach_(gastric)_cancer	NA
chr6	1.1E+08	CA	C	FIG4	FRAMESHIFT-DEL	c.1855delA	p.Asn620IlefsTer3	NA	NA	NA	NA
chr6	1.12E+08	G	GC	REV3L	FRAMESHIFT-INS	c.8323dupG	p.Ala2775GlyfsTer2	NA	NA	chr6*111643799*111643799*C*T*2*2*Colorectal_cancer	chr6*111643799*111643799*COSM1223567*G*A*1*large_intestine*1|chr6*111643799*111643799*COSM1223568*G*A*1*large_intestine*1
chr6	1.28E+08	TC	T	KIAA0408	FRAMESHIFT-DEL	c.1868delG	p.Gly623AspfsTer4	NA	NA	chr6*127767596*127767596*G*del*2*1*Colorectal_cancer	chr6*127767595*127767596*COSM1440281*TC*T*3*NS
chr6	1.28E+08	C	CA	SOGA3	FRAMESHIFT-INS	c.2835dupT	p.Val946CysfsTer28	NA	NA	chr6*127794434*127794434*ins*A*1*1*Stomach_(gastric)_cancer	NA
chr6	1.28E+08	C	CA	SOGA3	FRAMESHIFT-INS	c.2835dupT	p.Val946CysfsTer28	NA	NA	chr6*127794434*127794434*ins*A*1*1*Stomach_(gastric)_cancer	NA
chr6	1.64E+08	TA	T	QKI	FRAMESHIFT-DEL	c.394delA	p.Lys134ArgfsTer14	NA	NA	chr6*163899919*163899920*ins*A*1*1*Colorectal_cancer|chr6*163899920*163899920*A*del*8*7*Stomach_(gastric)_cancer|chr6*163899920*163899921*AA*del*1*1*Esophageal_cancer	chr6*163899919*163899919*COSM1442108*T*TA*1*large_intestine*1|chr6*163899919*163899919*COSM1442109*T*TA*1*large_intestine*1|chr6*163899919*163899920*COSM1192820*TA*T*2*pancreas
chr17	2577404	G	A	PAFAH1B1	MISSENSE	c.722G>A	p.Arg241Gln	PATHOGENIC	Subcortical_band_heterotopia	NA	NA
chr17	3445843	C	T	TRPV3	MISSENSE	c.616G>A	p.Ala206Thr	NA	NA	NA	NA
chr17	4713020	C	T	PLD2	MISSENSE	c.649C>T	p.Arg217Cys	NA	NA	NA	NA
chr17	4805243	A	G	CHRNE	MISSENSE	c.484T>C	p.Cys162Arg	NA	NA	NA	NA
chr17	5433872	G	T	NLRP1	MISSENSE	c.3449C>A	p.Pro1150His	NA	NA	NA	NA

chr17	7318310	G	A	NLGN2	MISSENSE	c.880G>A	p.Ala294Thr	NA	NA	chr17*7318310*7318310*G*A*1*1*Colorectal_cancer	chr17*7318310*7318310*COSM3691790*G*A*1*large_intestine*1
chr17	7751827	G	A	KDM6B	MISSENSE	c.2221G>A	p.Ala741Thr	NA	NA	NA	chr17*7751827*7751828*COSM1387267*GC*G*1*large_intestine*1
chr17	8808097	G	T	PIK3R5	MISSENSE	c.409C>A	p.Pro137Thr	NA	NA	NA	NA
chr17	10427113	C	T	MYH2	MISSENSE	c.5264G>A	p.Arg1755His	NA	NA	NA	NA
chr17	12028662	G	C	MAP2K4	MISSENSE	c.865G>C	p.Asp289His	NA	NA	NA	NA
chr17	17701034	G	A	RAI1	MISSENSE	c.4772G>A	p.Arg1591Gln	NA	NA	NA	NA
chr17	18046125	G	A	MYO15A	MISSENSE	c.5881G>A	p.Ala1961Thr	NA	NA	NA	NA
chr17	27446432	G	A	MYO18A	MISSENSE	c.1750C>T	p.Arg584Cys	NA	NA	NA	NA
chr17	27493249	C	T	MYO18A	MISSENSE	c.710G>A	p.Arg237His	NA	NA	chr17*27493249*27493249*G*A*2*2*Colorectal_cancer	chr17*27493249*27493249*COSM1216337*C*T*1*large_intestine*1|chr17*27493249*27493249*COSM1216338*C*T*1*large_intestine*1
chr17	27577276	G	A	CRYBA1	MISSENSE	c.173G>A	p.Arg58His	NA	NA	NA	NA
chr17	27903882	C	T	GIT1	MISSENSE-SS-PRX	c.1253G>A	p.Arg418Gln	NA	NA	chr17*27903882*27903882*G*A*1*1*Head_and_neck_cancer	chr17*27903882*27903882*COSM123363*C*T*1*upper_aerodigestive_tract*1
chr17	28011672	G	A	SSH2	NONSENSE	c.307C>T	p.Arg103Ter	NA	NA	NA	NA
chr17	35538254	C	T	ACACA	MISSENSE	c.4820G>A	p.Gly1607Glu	NA	NA	NA	NA
chr17	36061125	A	G	HNF1B	MISSENSE	c.1397T>C	p.Leu466Pro	NA	NA	NA	NA
chr17	37949015	C	A	IKZF3	MISSENSE	c.335G>T	p.Arg112Met	NA	NA	NA	NA
chr17	39580759	C	T	KRT37	MISSENSE	c.17G>A	p.Ser6Asn	NA	NA	NA	NA
chr17	39661415	C	T	KRT13	MISSENSE	c.388G>A	p.Ala130Thr	NA	NA	chr17*39661415*39661415*G*A*1*1*Lung_cancer	chr17*39661415*39661415*COSM337476*C*T*2*lung
chr17	41720884	G	A	MEOX1	MISSENSE	c.614C>T	p.Ala205Val	NA	NA	chr17*41720884*41720884*C*T*1*1*Colorectal_cancer	chr17*41720884*41720884*COSM1383564*G*A*1*large_intestine*1
chr17	42085916	G	A	NAGS	MISSENSE	c.1552G>A	p.Ala518Thr	NA	NA	chr17*42085916*42085916*G*A*2*2*Colorectal_cancer	chr17*42085916*42085916*COSM257487*G*A*1*large_intestine*1
chr17	42477016	C	T	GPATCH8	MISSENSE	c.2429G>A	p.Arg810Gln	NA	NA	NA	NA
chr17	43552622	G	A	PLEKHM1	MISSENSE	c.767C>T	p.Thr256Met	NA	NA	NA	NA
chr17	45695810	C	T	NPEPPS	MISSENSE	c.2390C>T	p.Thr797Met	NA	NA	NA	NA
chr17	47696652	C	T	SPOP	MISSENSE	c.296G>A	p.Arg99Gln	NA	NA	NA	NA
chr17	48653463	G	A	CACNA1G	MISSENSE	c.1700G>A	p.Arg567His	NA	NA	NA	NA
chr17	56355383	C	T	MPO	MISSENSE	c.1009G>A	p.Val337Met	NA	NA	NA	NA
chr17	56384268	T	C	BZRAP1	MISSENSE	c.5045A>G	p.Tyr1682Cys	NA	NA	NA	NA
chr17	56393826	G	A	BZRAP1	MISSENSE	c.1948C>T	p.Arg650Trp	NA	NA	NA	NA
chr17	57181700	C	T	TRIM37	MISSENSE	c.77G>A	p.Arg26His	NA	NA	NA	NA

chr17	60742200	G	A	MRC2	MISSENSE	c.410G>A	p.Arg137His	NA	NA	chr17*60742200*60742200*G*T*1*1*Endometrial_cancer|chr17*60742200*60742200*G*A*2*2*Colorectal_cancer	chr17*60742200*60742200*COSM982473*G*T*1*endometrium*1|chr17*60742200*60742200*COSM288444*G*A*1*large_intestine*1
chr17	63200369	C	T	RGS9	MISSENSE	c.1153C>T	p.Arg385Cys	NA	NA	chr17*63200369*63200369*C*T*2*2*Colorectal_cancer|chr17*63200369*63200369*C*G*1*1*Lung_cancer	chr17*63200369*63200369*COSM561893*C*G*1*lung*1|chr17*63200369*63200369*COSM294760*C*T*1*large_intestine*1
chr17	73753168	C	T	ITGB4	MISSENSE	c.5198C>T	p.Thr1733Ile	NA	NA	NA	NA
chr17	75493433	G	T	SEPT9	MISSENSE	c.1610G>T	p.Arg537Leu	NA	NA	chr17*75493433*75493433*G*T*1*1*Lung_cancer	chr17*75493433*75493433*COSM377518*G*T*1*lung*1|chr17*75493433*75493433*COSM377517*G*T*1*lung*1
chr17	78155265	G	A	CARD14	MISSENSE	c.28G>A	p.Ala10Thr	NA	NA	chr17*78155265*78155265*G*A*2*2*Colorectal_cancer	chr17*78155265*78155265*COSM1199576*G*A*1*large_intestine*1
chr17	78155316	C	A	CARD14	MISSENSE	c.79C>A	p.Arg27Ser	NA	NA	NA	NA
chr17	78298873	G	A	RNF213	MISSENSE	c.3068G>A	p.Arg1023Gln	NA	NA	chr17*78298873*78298873*G*A*1*1*Endometrial_cancer	chr17*78298873*78298873*COSM1589237*G*A*1*endometrium*1|chr17*78298873*78298873*COSM985596*G*A*1*endometrium*1
chr17	78857650	C	T	RPTOR	MISSENSE	c.1720C>T	p.Arg574Cys	NA	NA	NA	NA
chr17	1631633	AG	A	WDR81	FRAMESHIFT-DEL	c.3381delG	p.Gly1129ValfsTer18	NA	NA	NA	NA
chr17	7193636	TG	T	YBX2	FRAMESHIFT-DEL	c.677delC	p.Pro226HisfsTer22	NA	NA	NA	NA
chr17	7606714	TG	T	WRAP53	FRAMESHIFT-DEL	c.1558delG	p.Ala522ArgfsTer26	NA	NA	chr17*7606714*7606714*ins*G*1*1*Stomach_(gastric)_cancer|chr17*7606715*7606715*G*del*1*1*Colorectal_cancer	chr17*7606714*7606715*COSM1386963*TG*T*1*large_intestine*1
chr17	8110129	C	CG	AURKB	FRAMESHIFT-INS	c.475dupC	p.Arg159ProfsTer15	NA	NA	NA	NA
chr17	18023292	AC	A	MYO15A	FRAMESHIFT-DEL	c.1179delC	p.Glu396ArgfsTer48	PATHOGENIC	Non-syndromic_genetic_deafness	NA	NA
chr17	20108262	TA	T	SPECC1	FRAMESHIFT-DEL	c.901delA	p.Asn303ThrfsTer63	NA	NA	chr17*20108262*20108262*ins*A*1*1*Colorectal_cancer|chr17*20108262*20108263*ins*A*1*1*Colon_cancer|chr17*20108263*20108263*A*del*2*2*Stomach_(gastric)_cancer	chr17*20108262*20108262*COSM297720*T*TA*1*large_intestine*1|chr17*20108262*20108263*COSM1163712*TA*T*4*pancreas

chr17	39975471	TC	T	FKBP10	FRAMESHIFT-DEL	c.738delC	p.Pro248HisfsTer11	NA	NA	chr17*39975472*39975472*C*T*2*2*Brain_cancer	chr17*39975472*39975472*COSM2151943*C*T*1*central_nervous_system*
chr17	44061182	CG	C	MAPT	FRAMESHIFT-DEL	c.1013delG	p.Gly339AlafsTer66	NA	NA	NA	NA
chr17	48356597	AC	A	TMEM92	FRAMESHIFT-DEL	c.409delC	p.Pro138LeufsTer86	NA	NA	chr17*48356598*48356598*C*del*2*1*Colorectal_cancer	chr17*48356597*48356598*COSM1384187*AC*A*2*large_intestine*2
chr17	48431330	AC	A	XYLT2	FRAMESHIFT-DEL	c.476delC	p.Lys161SerfsTer48	NA	NA	NA	NA
chr17	48434108	AC	A	XYLT2	FRAMESHIFT-DEL	c.1720delC	p.Pro575GlnfsTer32	NA	NA	NA	NA
chr17	54921518	CA	C	DGKE	FRAMESHIFT-DEL	c.604delA	p.Thr204GlnfsTer6	PATHOGENIC	Nephrotic_syndrome_type_7	chr17*54921519*54921519*A*del*3*1*Colorectal_cancer	chr17*54921518*54921519*COSM1384590*CA*C*3*large_intestine*3
chr17	56435160	AC	A	RNF43	FRAMESHIFT-DEL	c.1976delG	p.Gly659ValfsTer41	NA	NA	chr17*56435160*56435160*A*del*2*2*Stomach_(gastric)_cancer|chr17*56435161*56435161*G*del*4*4*Colorectal_cancer|chr17*56435161*56435161*C*del*9*5*Stomach_(gastric)_cancer	chr17*56435159*56435160*COSM1734865*GA*G*1*pancreas*1|chr17*56435160*56435161*COSM270052*AC*A*15*large_intestine
chr17	61457169	C	CA	TANC2	FRAMESHIFT-INS	c.2451_2452insA	p.Ala819SerfsTer5	NA	NA	NA	NA
chr17	63532584	GC	G	AXIN2	FRAMESHIFT-DEL	c.1994delG	p.Gly665AlafsTer24	PATHOGENIC	Carcinoma_of_colon	chr17*63532585*63532585*ins*C*3*1*Colorectal_cancer|chr17*63532585*63532585*G*del*10*2*Stomach_(gastric)_cancer	chr17*63532584*63532584*COSM1167822*G*GC*4*skin
chr17	70120159	C	CGAGCCGG	SOX9	FRAMESHIFT-INS	c.1161_1162insGAGCCGG	p.Gln391AlafsTer189	NA	NA	NA	NA
chr17	77808729	TG	T	CBX4	FRAMESHIFT-DEL	c.711delC	p.Glu239ArgfsTer3	NA	NA	NA	NA
chr17	77808750	TG	T	CBX4	FRAMESHIFT-DEL	c.690delC	p.Asn231ThrfsTer3	NA	NA	chr17*77808751*77808751*C*del*2*1*Colorectal_cancer	chr17*77808750*77808750*COSM1386959*T*TG*1*large_intestine*1|chr17*77808750*77808751*COSM1386960*TG*T*2*large_intestine*2
chr21	19642303	G	A	TMPRSS15	NONSENSE	c.3043C>T	p.Gln1015Ter	NA	NA	NA	NA
chr21	30313625	G	T	LTN1	MISSENSE	c.4537C>A	p.Leu1513Met	NA	NA	NA	NA
chr21	43161711	C	T	RIPK4	MISSENSE	c.1642G>A	p.Gly548Arg	NA	NA	NA	chr21*43161711*43161712*COSM1734529*CG*C*1*pancreas*1|chr21*43161711*43161712*COSM1734528*CG*C*1*pancreas*1

chr21	45170412	G	A	PDXK	MISSENSE	c.496G>A	p.Glu166Lys	NA	NA	chr21*45170412*45170412*G*T*1*1*Bladder_cancer	chr21*45170412*45170412*COSM1307854*G*T*1*urinary_tract*1|chr21*45170412*45170412*COSM1307855*G*T*1*urinary_tract*1
chr21	45678572	T	C	DNMT3L	MISSENSE	c.350A>G	p.Tyr117Cys	NA	NA	NA	NA
chr21	45709907	G	A	AIRE	MISSENSE	c.835G>A	p.Val279Ile	NA	NA	NA	NA
chr21	45821604	G	A	TRPM2	MISSENSE	c.2362G>A	p.Ala788Thr	NA	NA	chr21*45821604*45821604*G*A*1*1*Colorectal_cancer	chr21*45821604*45821604*COSM1414386*G*A*1*large_intestine*1
chr21	46320369	C	T	ITGB2	MISSENSE	c.763G>A	p.Val255Ile	NA	NA	NA	NA
chr21	46932197	C	T	COL18A1	MISSENSE	c.4445C>T	p.Thr1482Met	NA	NA	NA	NA
chr21	47409022	C	T	COL6A1	MISSENSE	c.829C>T	p.Pro277Ser	NA	NA	NA	NA
chr21	47662716	C	A	MCM3AP	MISSENSE-SS-PRX	c.5426G>T	p.Arg1809Leu	NA	NA	NA	NA
chr21	47662805	A	T	MCM3AP	MISSENSE	c.5337T>A	p.Phe1779Leu	NA	NA	chr21*47662805*47662805*A*T*1*1*Stomach_(gastric)_cancer	NA
chr21	47974143	C	T	DIP2A	MISSENSE	c.3092C>T	p.Ala1031Val	NA	NA	NA	NA
chr21	16338329	CT	C	NRIP1	FRAMESHIFT-DEL	c.2184delA	p.Glu729ArgfsTer5	NA	NA	chr21*16338330*16338330*T*del*2*2*Stomach_(gastric)_cancer|chr21*16338330*16338330*A*del*2*2*Colorectal_cancer	chr21*16338329*16338329*COSM1413397*C*CT*1*large_intestine*1|chr21*16338329*16338330*COSM1180922*CT*C*9*large_intestine
chr21	30339058	TAGAGGAG	T	LTN1	FRAMESHIFT-DEL	c.1886_1892delCTCCTCT	p.Ser629Ter	NA	NA	chr21*30339060*30339060*C*G*1*1*Head_and_neck_cancer	chr21*30339060*30339060*COSM125893*G*C*1*upper_aerodigestive_tract*1
chr16	2331204	G	A	ABCA3	MISSENSE	c.4183C>T	p.Pro1395Ser	NA	NA	NA	NA
chr16	2569334	C	T	RP11-20I23.1	MISSENSE	c.1081C>T	p.Arg361Cys	NA	NA	NA	NA
chr16	2903158	C	T	PRSS22	MISSENSE	c.890G>A	p.Arg297His	NA	NA	NA	NA
chr16	3722704	G	A	TRAP1	NONSENSE	c.1162C>T	p.Arg388Ter	NA	NA	NA	NA
chr16	4408430	G	A	CORO7-PAM16	MISSENSE	c.2395C>T	p.Arg799Trp	NA	NA	NA	NA
chr16	4408430	G	A	CORO7	MISSENSE	c.2395C>T	p.Arg799Trp	NA	NA	NA	NA
chr16	8866766	G	A	ABAT	MISSENSE	c.946G>A	p.Ala316Thr	NA	NA	chr16*8866766*8866766*G*T*1*1*Lung_cancer	NA
chr16	11145443	C	T	CLEC16A	MISSENSE	c.1940C>T	p.Thr647Met	NA	NA	NA	NA
chr16	15711019	T	A	KIAA0430	MISSENSE	c.2967A>T	p.Glu989Asp	NA	NA	NA	NA
chr16	15730000	C	T	KIAA0430	MISSENSE	c.344G>A	p.Arg115His	NA	NA	NA	NA
chr16	18849370	C	T	SMG1	MISSENSE	c.7379G>A	p.Arg2460His	NA	NA	NA	NA
chr16	20043688	C	T	GPR139	MISSENSE	c.431G>A	p.Arg144His	NA	NA	NA	NA
chr16	20329579	A	G	GP2	MISSENSE	c.1190T>C	p.Val397Ala	NA	NA	NA	NA
chr16	21730757	A	C	OTOA	MISSENSE	c.1738A>C	p.Ser580Arg	NA	NA	NA	NA
chr16	23226482	G	T	SCNN1G	NONSENSE	c.1642G>T	p.Glu548Ter	NA	NA	NA	NA
chr16	24373044	G	A	CACNG3	MISSENSE	c.808G>A	p.Asp270Asn	NA	NA	chr16*24373044*24373044*G*A*1*1*Lung_cancer	NA
chr16	28912098	C	T	ATP2A1	MISSENSE	c.1961C>T	p.Thr654Met	NA	NA	chr16*28912098*28912098*C*T*1*1*Colorectal_cancer	chr16*28912098*28912098*COSM1377198*C*T*1*large_intestine*1

chr16	46943611	C	T	GPT2	MISSENSE	c.592C>T	p.Leu198Phe	NA	NA	NA	NA
chr16	51171222	C	T	SALL1	MISSENSE	c.3776G>A	p.Gly1259Asp	NA	NA	NA	NA
chr16	51174226	G	A	SALL1	MISSENSE	c.1907C>T	p.Thr636Met	NA	NA	NA	NA
chr16	55703516	C	T	SLC6A2	MISSENSE	c.314C>T	p.Ala105Val	NA	NA	chr16*55703516*55703516*C*T*2*2*Colorectal_cancer	chr16*55703516*55703516*COSM1226534*C*T*1*large_intestine*1|chr16*55703516*55703516*COSM1226533*C*T*1*large_intestine*1
chr16	56917997	C	T	SLC12A3	MISSENSE	c.1706C>T	p.Ala569Val	NA	NA	chr16*56917997*56917997*C*T*2*2*Colorectal_cancer	chr16*56917997*56917997*COSM294816*C*T*1*large_intestine*1
chr16	57003422	A	G	CETP	MISSENSE	c.358A>G	p.Thr120Ala	NA	NA	NA	NA
chr16	57060573	G	A	NLRC5	MISSENSE	c.1718G>A	p.Cys573Tyr	NA	NA	NA	NA
chr16	57787374	G	A	KATNB1	MISSENSE	c.1120G>A	p.Ala374Thr	NA	NA	NA	NA
chr16	57921799	G	A	CNGB1	MISSENSE	c.3422C>T	p.Ala1141Val	NA	NA	chr16*57921799*57921799*C*T*2*2*Colon_cancer	chr16*57921799*57921799*COSM1201529*G*A*1*large_intestine*1
chr16	61851566	C	T	CDH8	MISSENSE	c.1094G>A	p.Arg365His	NA	NA	NA	NA
chr16	64984709	C	T	CDH11	MISSENSE	c.1855G>A	p.Ala619Thr	NA	NA	NA	NA
chr16	68371434	C	T	PRMT7	MISSENSE	c.464C>T	p.Ala155Val	NA	NA	chr16*68371434*68371434*C*T*1*1*Lung_cancer	chr16*68371434*68371434*COSM369425*C*T*1*lung*1
chr16	70698128	G	A	MTSS1L	MISSENSE	c.1696C>T	p.Arg566Cys	NA	NA	NA	NA
chr16	71686887	C	T	PHLPP2	MISSENSE	c.2623G>A	p.Ala875Thr	NA	NA	NA	NA
chr16	84214725	C	T	TAF1C	MISSENSE	c.1310G>A	p.Arg437His	NA	NA	NA	NA
chr16	84215650	C	T	TAF1C	MISSENSE	c.736G>A	p.Val246Ile	NA	NA	NA	NA
chr16	86544548	G	A	FOXF1	MISSENSE	c.373G>A	p.Asp125Asn	NA	NA	NA	NA
chr16	88504311	C	T	ZNF469	MISSENSE	c.10349C>T	p.Ala3450Val	NA	NA	NA	NA
chr16	88945758	C	T	CBFA2T3	MISSENSE	c.1582G>A	p.Glu528Lys	NA	NA	chr16*88945758*88945758*G*C*1*1*Colorectal_cancer	chr16*88945758*88945758*COSM1380477*C*G*1*large_intestine*1
chr16	89349945	C	T	ANKRD11	MISSENSE	c.3005G>A	p.Arg1002Gln	NA	NA	NA	NA
chr16	1550678	AG	A	TELO2	FRAMESHIFT-DEL	c.1260delG	p.Pro422LeufsTer3	NA	NA	chr16*1550679*1550679*G*A*1*1*Breast_cancer	NA
chr16	3781420	TG	T	CREBBP	FRAMESHIFT-DEL	c.4944delC	p.Ile1649SerfsTer95	NA	NA	NA	NA
chr16	15694508	AT	A	KIAA0430	FRAMESHIFT-DEL	c.4676delA	p.Asn1559MetfsTer3	NA	NA	NA	NA
chr16	30100077	GC	G	TBX6	FRAMESHIFT-DEL	c.704delG	p.Gly235AlafsTer15	PATHOGENIC	Spondylocostal_dysostosis_5	chr16*30100078*30100078*G*del*1*1*Colorectal_cancer	chr16*30100077*30100077*COSM1377333*G*GC*1*large_intestine*1|chr16*30100077*30100078*COSM1377334*GC*G*1*large_intestine*1
chr16	30736370	G	GC	SRCAP	FRAMESHIFT-INS	c.5625_5626insC	p.Pro1879ThrfsTer21	NA	NA	chr16*30736370*30736370*ins*C*2*2*Stomach_(gastric)_cancer|chr16*30736370*30736371*ins*C*1*1*Small_intestine_cancer	chr16*30736370*30736370*COSM1377474*G*GC*8*large_intestine

chr16	55513515	GC	G	MMP2	FRAMESHIFT-DEL	c.125delC	p.Thr45ArgfsTer10	NA	NA	NA	NA
chr16	57065341	TC	T	NLRC5	FRAMESHIFT-DEL	c.2444delC	p.Pro816ArgfsTer30	NA	NA	chr16*57065342*57065342*C*del*1*1*Colorectal_cancer	chr16*57065341*57065342*COSM1378519*TC*T*1*large_intestine*1
chr16	57088735	TA	T	NLRC5	FRAMESHIFT-DEL	c.3580delA	p.Lys1195ArgfsTer38	NA	NA	NA	NA
chr16	65022076	AC	A	CDH11	FRAMESHIFT-DEL	c.982delG	p.Val328Ter	NA	NA	NA	NA
chr16	67646012	A	AAC	CTCF	FRAMESHIFT-INS	c.940_941insAC	p.Gly318GlnfsTer16	NA	NA	chr16*67646012*67646013*ins*AC*2*1*Endometrial_carcinoma	chr16*67646012*67646012*COSM972481*A*AAC*2*endometrium*2|chr16*67646012*67646014*COSM972482*AAC*A*1*endometrium*1
chr16	68397695	TCTC	T	SMPD3	INFRAME-DEL	c.1627_1629delGAG	p.Glu543del	NA	NA	NA	NA
chr16	87723700	AC	A	JPH3	FRAMESHIFT-DEL	c.1735delC	p.Val581CysfsTer28	NA	NA	NA	NA
chr16	88888999	G	GA	GALNS	FRAMESHIFT-INS	c.1361dupT	p.Ser455GlnfsTer48	NA	NA	NA	NA
chr18	21137149	C	T	NPC1	MISSENSE	c.887G>A	p.Arg296Gln	NA	NA	NA	NA
chr18	44157689	C	T	LOXHD1	MISSENSE	c.1951G>A	p.Val651Met	NA	NA	NA	NA
chr18	44157689	C	T	LOXHD1	MISSENSE	c.1951G>A	p.Val651Met	NA	NA	NA	NA
chr18	44157689	C	T	LOXHD1	MISSENSE	c.1117G>A	p.Val373Met	NA	NA	NA	NA
chr18	56390280	C	T	MALT1	MISSENSE-SS-PRX	c.1019C>T	p.Ala340Val	NA	NA	NA	NA
chr18	72998519	C	T	TSHZ1	MISSENSE	c.1022C>T	p.Ala341Val	NA	NA	NA	NA
chr18	74607032	G	A	ZNF236	MISSENSE	c.1475G>A	p.Arg492His	NA	NA	NA	NA
chr18	10741063	CT	C	PIEZO2	FRAMESHIFT-DEL	c.4598delA	p.Lys1533SerfsTer34	NA	NA	chr18*10741064*10741064*T*del*2*2*Stomach_(gastric)_cancer	NA
chr18	52895519	AG	A	TCF4	FRAMESHIFT-DEL	c.2258delC	p.Pro753LeufsTer32	NA	NA	chr18*52895520*52895520*G*del*1*1*Esophageal_cancer|chr18*52895520*52895520*C*T*1*1*Renal_cancer	chr18*52895519*52895520*COSM1267549*AG*A*2*oesophagus
chr18	67697248	CT	C	RTTN	FRAMESHIFT-DEL-SS-PRX	c.5744delA	p.Lys1915ArgfsTer9	NA	NA	chr18*67697249*67697249*T*del*2*2*Stomach_(gastric)_cancer|chr18*67697249*67697249*A*del*2*2*Colorectal_cancer	chr18*67697248*67697248*COSM1389599*C*CT*1*large_intestine*1|chr18*67697248*67697249*COSM1180860*CT*C*5*large_intestine*5
chr3	10387166	G	A	ATP2B2	MISSENSE	c.2605C>T	p.Arg869Cys	NA	NA	NA	NA
chr3	20215824	A	G	SGOL1	MISSENSE	c.1199T>C	p.Val400Ala	NA	NA	NA	NA
chr3	25777586	T	C	NGLY1	MISSENSE	c.1058A>G	p.Asp353Gly	NA	NA	NA	NA
chr3	38523818	G	A	ACVR2B	MISSENSE	c.1204G>A	p.Ala402Thr	NA	NA	NA	NA
chr3	38763831	C	T	SCN10A	MISSENSE	c.3425G>A	p.Arg1142His	NA	NA	chr3*38763831*38763831*G*A*1*1*Colon_cancer|chr3*38763831*38763831*C*T*1*1*Colorectal_cancer	chr3*38763831*38763831*COSM292451*C*T*1*large_intestine*1
chr3	39230654	G	A	XIRP1	MISSENSE	c.283C>T	p.Arg95Cys	NA	NA	chr3*39230654*39230654*G*A*1*1*Endometrial_cancer	chr3*39230654*39230654*COSM1044502*G*A*1*endometrium*1

chr3	48610322	A	T	COL7A1	MISSENSE	c.6804T>A	p.Asp2268Glu	NA	NA	NA	NA
chr3	48624754	G	A	COL7A1	MISSENSE	c.3008C>T	p.Pro1003Leu	NA	NA	NA	NA
chr3	48718994	T	G	NCKIPSD	MISSENSE	c.818A>C	p.Glu273Ala	NA	NA	NA	NA
chr3	49690185	C	T	BSN	MISSENSE	c.3196C>T	p.Arg1066Cys	NA	NA	chr3*49690185*49690185*C*T*1*1*Colon_cancer	chr3*49690185*49690185*COSM1165499*C*T*1*large_intestine*1
chr3	49694614	C	T	BSN	MISSENSE	c.7625C>T	p.Thr2542Met	NA	NA	NA	NA
chr3	49759419	C	G	GMPPB	MISSENSE	c.930G>C	p.Trp310Cys	NA	NA	NA	NA
chr3	50369170	G	A	RASSF1	MISSENSE	c.592C>T	p.Arg198Trp	NA	NA	NA	NA
chr3	50413407	T	C	CACNA2D2	MISSENSE	c.1760A>G	p.Glu587Gly	NA	NA	NA	NA
chr3	52822007	G	A	ITIH1	MISSENSE	c.1930G>A	p.Val644Met	NA	NA	NA	NA
chr3	53262080	A	G	TKT	MISSENSE	c.1589T>C	p.Leu530Pro	NA	NA	NA	NA
chr3	53531424	G	A	CACNA1D	MISSENSE	c.313G>A	p.Ala105Thr	NA	NA	chr3*53531424*53531424*G*A*5*5*Lung_cancer	chr3*53531424*53531424*COSM1650190*G*A*1*lung*1|chr3*53531424*53531424*COSM88069*G*A*5*oesophagus
chr3	58484500	G	T	KCTD6	MISSENSE	c.18G>T	p.Trp6Cys	NA	NA	NA	NA
chr3	73024213	G	A	GXYLT2	MISSENSE	c.1235G>A	p.Arg412Gln	NA	NA	NA	NA
chr3	77666774	G	A	ROBO2	MISSENSE	c.3452G>A	p.Arg1151Gln	NA	NA	NA	NA
chr3	1.13E+08	C	T	BOC	MISSENSE	c.3310C>T	p.Arg1104Cys	NA	NA	NA	NA
chr3	1.19E+08	C	T	TMEM39A	MISSENSE	c.622G>A	p.Ala208Thr	NA	NA	NA	NA
chr3	1.21E+08	G	A	HCLS1	MISSENSE	c.478C>T	p.Arg160Trp	NA	NA	NA	NA
chr3	1.22E+08	C	A	IQCB1	MISSENSE	c.1205G>T	p.Arg402Met	NA	NA	NA	NA
chr3	1.22E+08	T	G	ILDR1	MISSENSE	c.708A>C	p.Lys236Asn	NA	NA	NA	NA
chr3	1.23E+08	C	T	ADCY5	MISSENSE-SS-PRX	c.1808G>A	p.Arg603His	NA	NA	NA	NA
chr3	1.23E+08	G	A	MYLK	MISSENSE	c.3395C>T	p.Thr1132Met	NA	NA	chr3*123418920*123418920*C*T*1*1*Breast_cancer	NA
chr3	1.26E+08	G	A	UROC1	MISSENSE	c.1172C>T	p.Thr391Met	NA	NA	NA	NA
chr3	1.32E+08	C	A	NPHP3	MISSENSE	c.3634G>T	p.Gly1212Cys	NA	NA	NA	NA
chr3	1.42E+08	C	A	ATR	MISSENSE-SS-PRX	c.7762G>T	p.Ala2588Ser	NA	NA	NA	NA
chr3	1.51E+08	G	A	MED12L	MISSENSE	c.3319G>A	p.Ala1107Thr	NA	NA	chr3*151085530*151085530*G*A*1*1*Colorectal_cancer	chr3*151085530*151085530*COSM1419972*G*A*1*large_intestine*1
chr3	1.51E+08	C	A	MED12L	MISSENSE	c.4916C>A	p.Pro1639His	NA	NA	NA	NA
chr3	1.8E+08	C	T	TTC14	MISSENSE	c.2110C>T	p.Arg704Cys	NA	NA	NA	NA
chr3	1.83E+08	C	A	MCCC1	MISSENSE	c.2165G>T	p.Arg722Met	NA	NA	NA	NA
chr3	1.83E+08	C	T	MCF2L2	MISSENSE-SS-PRX	c.1865G>A	p.Arg622His	NA	NA	chr3*182948803*182948803*G*A*1*1*Colorectal_cancer	chr3*182948803*182948803*COSM32291*C*T*1*large_intestine*1
chr3	1.84E+08	C	T	EIF4G1	MISSENSE	c.2246C>T	p.Ala749Val	NA	NA	NA	NA
chr3	1.88E+08	G	A	LPP	MISSENSE	c.385G>A	p.Ala129Thr	NA	NA	NA	NA
chr3	1.9E+08	C	A	CLDN16	MISSENSE	c.539C>A	p.Pro180Gln	NA	NA	NA	NA
chr3	1.96E+08	C	A	MUC4	MISSENSE	c.12763G>T	p.Asp4255Tyr	NA	NA	NA	NA
chr3	1.96E+08	C	T	MUC4	MISSENSE	c.11272G>A	p.Ala3758Thr	NA	NA	NA	NA
chr3	1.96E+08	T	C	MUC4	MISSENSE	c.9472A>G	p.Thr3158Ala	NA	NA	NA	NA
chr3	1.96E+08	T	G	MUC4	MISSENSE	c.9424A>C	p.Thr3142Pro	NA	NA	NA	NA
chr3	1.96E+08	G	A	MUC4	MISSENSE	c.8858C>T	p.Ala2953Val	NA	NA	NA	NA
chr3	1.96E+08	C	T	MUC4	MISSENSE	c.8296G>A	p.Ala2766Thr	NA	NA	NA	NA

chr3	1.96E+08	T	G	MUC4	MISSENSE	c.5717A>C	p.His1906Pro	NA	NA	NA	chr3*195512734*195512734*COSM225536*T*C*1*NS*1|chr3*195512734*195512734*COSM225537*T*C*1*NS*1
chr3	1.96E+08	G	A	MUC4	MISSENSE	c.3290C>T	p.Ala1097Val	NA	NA	chr3*195515161*195515161*C*T*1*1*Melanoma	chr3*195515161*195515161*COSM226544*G*A*1*skin*1|chr3*195515161*195515161*COSM226543*G*A*1*skin*1
chr3	1.96E+08	C	T	FBXO45	NONSENSE	c.691C>T	p.Arg231Ter	NA	NA	NA	NA
chr3	4854884	T	TG	ITPR1	FRAMESHIFT-INS	c.7482_7483insG	p.Glu2496GlyfsTer22	NA	NA	NA	NA
chr3	7456818	CA	C	GRM7	FRAMESHIFT-DEL	c.1143delA	p.Glu384LysfsTer28	NA	NA	chr3*7456819*7456819*A*del*1*1*Colorectal_cancer	chr3*7456818*7456819*COSM1425298*CA*C*1*large_intestine*1
chr3	13438889	AG	A	NUP210	FRAMESHIFT-DEL	c.403delC	p.Leu135TrpfsTer3	NA	NA	chr3*13438890*13438890*C*del*1*1*Lung_cancer	chr3*13438889*13438890*COSM392248*AG*A*2*lung
chr3	38158477	TG	T	DLEC1	FRAMESHIFT-DEL	c.4192delG	p.Gly1399AlafsTer32	NA	NA	NA	NA
chr3	38949440	CT	C	SCN11A	FRAMESHIFT-DEL-SS-PRX	c.1472delA	p.Lys491SerfsTer5	NA	NA	chr3*38949440*38949440*ins*T*1*1*Stomach_(gastric)_cancer|chr3*38949441*38949441*T*del*3*2*Stomach_(gastric)_cancer|chr3*38949441*38949441*A*del*1*1*Stomach_(gastric)_cancer	chr3*38949440*38949441*COSM1422885*CT*C*4*large_intestine
chr3	48688850	TCA	T	CELSR3	FRAMESHIFT-DEL	c.6126_6127delTG	p.Cys2042Ter	NA	NA	NA	NA
chr3	51664386	GA	G	RAD54L2	FRAMESHIFT-DEL	c.581delA	p.Ser196AlafsTer6	NA	NA	chr3*51664386*51664387*GA*TT*1*1*Prostate_cancer	NA

chr3	52610608	C	CT	PBRM1	FRAMESHIFT-INS	c.3564dupA	p.Glu1189ArgfsTer6	NA	NA	chr3*52610608*52610608*G*T*1*1*Renal_cell_cancer	chr3*52610603*52610618*COSM1659585*TACTTCTTTTTTGTAG*T*1*kidney*1|chr3*52610603*52610618*COSM1659584*TACTTCTTTTTTGTAG*T*1*kidney*1|chr3*52610603*52610618*COSM1659586*TACTTCTTTTTTGTAG*T*1*kidney*1|chr3*52610608*52610608*COSM422819*C*A*1*kidney*1|chr3*52610608*52610608*COSM422820*C*A*1*kidney*1|chr3*52610608*52610608*COSM422821*C*A*1*kidney*1|chr3*52
chr3	53220652	C	CG	PRKCD	FRAMESHIFT-INS	c.1293_1294insG	p.Asp434GlyfsTer13	NA	NA	chr3*53220652*53220653*ins*G*1*1*Colorectal_cancer	chr3*53220652*53220652*COSM1424619*C*CG*1*large_intestine*1|chr3*53220652*53220652*COSM1424620*C*CG*1*large_intestine*1|chr3*53220652*53220653*COSM1424622*CG*C*3*N
chr3	57276920	CA	C	APPL1	FRAMESHIFT-DEL	c.453delA	p.Arg154GlufsTer6	NA	NA	chr3*57276921*57276921*A*del*3*3*Colorectal_cancer	chr3*57276920*57276921*COSM259475*CA*C*4*large_intestine*4
chr3	97806112	GT	G	OR5AC2	FRAMESHIFT-DEL	c.97delT	p.Leu34TrpfsTer14	NA	NA	NA	NA
chr3	1.23E+08	TC	T	MYLK	FRAMESHIFT-DEL	c.472delG	p.Glu158SerfsTer79	NA	NA	chr3*123457860*123457860*G*del*1*1*Colon_cancer|chr3*123457860*123457860*C*del*1*1*Colorectal_ca	chr3*123457859*123457860*COSM296851*TC*T*1*large_intestine*1
chr3	1.26E+08	CG	C	UROC1	FRAMESHIFT-DEL	c.161delC	p.Pro54ArgfsTer32	NA	NA	NA	NA
chr3	1.32E+08	AT	A	ACAD11	FRAMESHIFT-DEL	c.1374delA	p.Lys458AsnfsTer49	NA	NA	NA	NA
chr3	1.42E+08	CT	C	ATR	FRAMESHIFT-DEL	c.5440delA	p.Arg1814GlufsTer10	NA	NA	chr3*142217556*142217557*ins*T*1*1*Esophageal_cancer|chr3*142217557*142217557*T*del*3*1*Endometrial_cancer	chr3*142217556*142217556*COSM1240880*C*CT*1*oesophagus*1|chr3*142217556*142217557*COSM1419630*CT*C*1*large_intestine*1
chr3	1.58E+08	AGGT	A	SHOX2	INFRAME-DEL	c.860_862delACC	p.His287del	NA	NA	NA	NA

chr3	1.58E+08	GT	G	GFM1	FRAMESHIFT-DEL	c.1768delT	p.Leu591Ter	NA	NA	NA	NA
chr3	1.65E+08	C	CA	SI	FRAMESHIFT-INS	c.2025dupT	p.Gly676TrpfsTer8	NA	NA	NA	NA
chr3	1.77E+08	AT	A	TBL1XR1	FRAMESHIFT-DEL	c.377delA	p.Asn126MetfsTer16	NA	NA	chr3*176769342*176769342*T*del*1*1*Stomach_(gastric)_cancer	chr3*176769341*176769342*COSM1420706*AT*A*2*large_intestine*2
chr3	1.84E+08	TCTC	T	ECE2	INFRAME-DEL	c.135_137delCTC	p.Ser47del	NA	NA	NA	NA
chr3	1.84E+08	GC	G	EIF4G1	FRAMESHIFT-DEL	c.27delC	p.Pro11HisfsTer31	NA	NA	NA	NA
chr3	1.87E+08	CG	C	BCL6	FRAMESHIFT-DEL	c.1418delC	p.Pro473ArgfsTer117	NA	NA	chr3*187446269*187446269*ins*G*1*1*Colorectal_cancer|chr3*187446270*187446270*G*A*1*1*Colorectal_cancer|chr3*187446270*187446270*C*T*1*1*Rectal_cancer	chr3*187446269*187446269*COSM293223*C*CG*1*large_intestine*1|chr3*187446270*187446270*COSM174704*G*A*1*large_intestine*1
chr12	7069872	C	T	PTPN6	MISSENSE	c.1720C>T	p.Arg574Trp	NA	NA	NA	NA
chr12	7531774	G	A	CD163L1	MISSENSE	c.2171C>T	p.Ala724Val	NA	NA	NA	NA
chr12	7848093	C	T	GDF3	MISSENSE	c.232G>A	p.Val78Ile	LIKELY_PATHOGENIC	not_provided	NA	NA
chr12	9268379	C	T	A2M	MISSENSE	c.67G>A	p.Ala23Thr	NA	NA	NA	NA
chr12	40757343	G	A	LRRK2	MISSENSE	c.7168G>A	p.Val2390Met	UNCERTAIN_SIGNIFICANCE	Parkinson_disease_8_autosomal_dominant	NA	NA
chr12	45797319	C	A	ANO6	MISSENSE-SS-PRX	c.1943C>A	p.Pro648His	NA	NA	chr12*45797319*45797319*C*T*1*1*Endometrial_cancer	chr12*45797319*45797319*COSM939477*C*T*1*endometrium*1|chr12*45797319*45797319*COSM939478*C*T*1*endometrium*1
chr12	45803162	C	T	ANO6	NONSENSE	c.1966C>T	p.Arg656Ter	NA	NA	NA	NA
chr12	48238764	G	A	VDR	MISSENSE	c.1199C>T	p.Ala400Val	NA	NA	chr12*48238764*48238764*G*G*1*1*Ovarian_cancer|chr12*48238764*48238764*C*T*1*1*Ovarian_cancer	chr12*48238764*48238764*COSM1322492*G*A*1*ovary*1
chr12	49425151	C	T	KMT2D	MISSENSE	c.13337G>A	p.Ser4446Asn	NA	NA	NA	NA
chr12	49447415	C	T	KMT2D	MISSENSE	c.683G>A	p.Arg228His	NA	NA	NA	NA
chr12	51495754	G	A	TFCP2	MISSENSE	c.1115C>T	p.Ala372Val	NA	NA	chr12*51495754*51495754*C*A*1*1*Lung_cancer	chr12*51495754*51495754*COSM1512309*G*T*1*lung*1
chr12	52200945	G	A	SCN8A	MISSENSE	c.5675G>A	p.Arg1892His	NA	NA	NA	NA
chr12	52695788	G	A	KRT86	MISSENSE	c.88G>A	p.Ala30Thr	NA	NA	NA	NA
chr12	53699720	A	G	C12orf10	MISSENSE	c.518A>G	p.Asp173Gly	NA	NA	NA	NA
chr12	53701466	G	A	AAAS	MISSENSE	c.1448C>T	p.Pro483Leu	NA	NA	NA	NA
chr12	53823984	C	T	AMHR2	MISSENSE	c.1343C>T	p.Pro448Leu	NA	NA	chr12*53823984*53823984*C*T*1*1*Lung_cancer	chr12*53823984*53823984*COSM1512469*C*T*1*lung*1
chr12	54333181	A	G	HOXC13	MISSENSE	c.491A>G	p.Asp164Gly	NA	NA	NA	NA
chr12	56481660	C	T	ERBB3	MISSENSE	c.695C>T	p.Ala232Val	NA	NA	chr12*56481660*56481660*C*T*4*3*Stomach_(gastric)_cancer	chr12*56481660*56481660*COSM1242240*C*T*2*oesophagus
chr12	57431731	C	T	MYO1A	MISSENSE	c.1883G>A	p.Arg628His	NA	NA	NA	NA

chr12	57579329	C	T	LRP1	MISSENSE	c.6479C>T	p.Ala2160Val	NA	NA	NA	NA
chr12	58017819	C	T	SLC26A10	MISSENSE	c.1165C>T	p.Arg389Cys	NA	NA	chr12*58017819*58017819*C*T*2*2*Endometrial_cancer	chr12*58017819*58017819*COSM942165*C*T*1*endometrium*1|chr12*58017819*58017819*COSM942164*C*T*1*endometrium*1
chr12	63541203	G	A	AVPR1A	MISSENSE	c.1193C>T	p.Thr398Met	NA	NA	chr12*63541203*63541203*C*G*1*1*Colorectal_cancer	chr12*63541203*63541203*COSM3688389*G*C*1*large_intestine*1
chr12	66800165	T	C	GRIP1	MISSENSE	c.1726A>G	p.Asn576Asp	NA	NA	NA	NA
chr12	70949874	G	A	PTPRB	MISSENSE	c.4769C>T	p.Ala1590Val	NA	NA	NA	NA
chr12	76741292	G	A	BBS10	MISSENSE	c.473C>T	p.Ser158Leu	NA	NA	NA	NA
chr12	80712399	C	A	OTOGL	MISSENSE	c.3681C>A	p.Ser1227Arg	NA	NA	NA	NA
chr12	88566447	A	C	TMTC3	MISSENSE	c.1124A>C	p.Glu375Ala	NA	NA	NA	NA
chr12	89744379	G	A	DUSP6	MISSENSE	c.824C>T	p.Ala275Val	NA	NA	NA	NA
chr12	99102382	T	G	APAF1	MISSENSE	c.2741T>G	p.Val914Gly	NA	NA	NA	NA
chr12	1.02E+08	G	A	GNPTAB	MISSENSE	c.1426C>T	p.Arg476Cys	NA	NA	NA	NA
chr12	1.04E+08	C	T	HSP90B1	MISSENSE-SS-PRX	c.745C>T	p.Leu249Phe	NA	NA	NA	NA
chr12	1.09E+08	C	T	SART3	MISSENSE	c.1111G>A	p.Ala371Thr	NA	NA	chr12*108931931*108931931*G*A*2*2*Colorectal_cancer	chr12*108931931*108931931*COSM1224689*C*T*1*large_intestine*1
chr12	1.12E+08	G	A	CUX2	MISSENSE	c.3421G>A	p.Gly1141Ser	NA	NA	NA	NA
chr12	1.18E+08	T	C	NOS1	MISSENSE	c.2189A>G	p.Lys730Arg	NA	NA	NA	NA
chr12	1.18E+08	C	T	KSR2	MISSENSE	c.1067G>A	p.Arg356His	NA	NA	NA	NA
chr12	1.22E+08	G	T	WDR66	MISSENSE	c.2836G>T	p.Gly946Cys	NA	NA	NA	NA
chr12	1.33E+08	A	T	CHFR	MISSENSE	c.721T>A	p.Ser241Thr	NA	NA	NA	NA
chr12	667809	AC	A	B4GALNT3	FRAMESHIFT-DEL	c.2744delC	p.Gln917SerfsTer9	NA	NA	NA	NA
chr12	7242009	AG	A	C1R	FRAMESHIFT-DEL	c.644delC	p.Pro215LeufsTer100	NA	NA	chr12*7242010*7242010*C*del*2*2*Colorectal_cancer	chr12*7242009*7242010*COSM294194*AG*A*1*large_intestine*1
chr12	7242293	TC	T	C1R	FRAMESHIFT-DEL	c.457delG	p.Glu153ArgfsTer162	NA	NA	NA	NA
chr12	11420243	TG	T	PRB3	FRAMESHIFT-DEL	c.812delC	p.Pro271HisfsTer49	NA	NA	NA	NA
chr12	11506272	TG	T	PRB1	FRAMESHIFT-DEL	c.365delC	p.Pro122HisfsTer91	NA	NA	NA	NA
chr12	14018853	AC	A	GRIN2B	FRAMESHIFT-DEL	c.289delG	p.Val97TrpfsTer36	NA	NA	NA	NA
chr12	20522790	CG	C	PDE3A	FRAMESHIFT-DEL	c.573delG	p.Val193TrpfsTer14	NA	NA	chr12*20522790*20522790*C*T*1*1*Esophageal_cancer|chr12*20522791*20522791*G*A*2*2*Colorectal_cancer	chr12*20522790*20522790*COSM1261744*C*T*1*oesophagus*1|chr12*20522791*20522791*COSM182729*G*A*1*large_intestine*1
chr12	26568291	CA	C	ITPR2	FRAMESHIFT-DEL	c.7250delT	p.Leu2417Ter	NA	NA	NA	NA
chr12	32639069	T	TG	FGD4	FRAMESHIFT-INS	c.2_3insG	p.Cys4LeufsTer25	NA	NA	NA	NA
chr12	52845812	AC	A	KRT6B	FRAMESHIFT-DEL	c.50delG	p.Gly17ValfsTer129	NA	NA	NA	NA

chr12	56092681	TC	T	ITGA7	FRAMESHIFT-DEL	c.822delG	p.Gly276ValfsTer8	OTHER	not_specified	chr12*56092682*56092682*G*A*1*1*Colorectal_cancer	chr12*56092682*56092682*COSM1562007*C*T*1*large_intestine*1|chr12*56092682*56092682*COSM1562006*C*T*1*large_intestine*1
chr12	56094787	TG	T	ITGA7	FRAMESHIFT-DEL	c.565delC	p.Gln189LysfsTer95	NA	NA	NA	NA
chr12	57422572	CT	C	MYO1A	FRAMESHIFT-DEL	c.3098delA	p.Lys1033ArgfsTer8	NA	NA	chr12*57422572*57422572*ins*T*1*1*Stomach_(gastric)_cancer|chr12*57422573*57422573*T*del*3*2*Stomach_(gastric)_cancer|chr12*57422573*57422573*T*C*2*2*Stomach_(gastric)_cancer	chr12*57422571*57422581*COSM250024*CCTTTTTTTTG*C*1*skin*1|chr12*57422572*57422573*COSM1363202*CT*C*5*large_intestine*5|chr12*57422573*57422573*COSM941854*T*C*2*large_intestine
chr12	57598961	TCTC	T	LRP1	INFRAME-DEL	c.11265_11267delCTC	p.Ser3758del	NA	NA	NA	NA
chr12	57603939	G	GC	LRP1	FRAMESHIFT-INS	c.12567_12568insC	p.Asp4193ArgfsTer9	NA	NA	chr12*57603939*57603940*ins*C*2*1*Colorectal_cancer	chr12*57603939*57603939*COSM1363263*G*GC*2*large_intestine*2
chr12	88443167	CT	C	CEP290	FRAMESHIFT-DEL	c.7233delA	p.Glu2412AsnfsTer24	NA	NA	NA	NA
chr12	88487620	AT	A	CEP290	FRAMESHIFT-DEL	c.3235delA	p.Met1079CysfsTer9	NA	NA	NA	NA
chr12	99071206	C	CA	APAF1	FRAMESHIFT-INS	c.1797_1798insA	p.Asn602LysfsTer24	NA	NA	NA	chr12*99071206*99071207*COSM1365084*CA*C*3*large_intestine*3|chr12*99071206*99071208*COSM291512*CAA*C*1*large_intestine*1
chr12	1.1E+08	TG	T	ACACB	FRAMESHIFT-DEL	c.763delG	p.Asp257IlefsTer17	NA	NA	NA	NA
chr12	1.13E+08	CA	C	OAS2	FRAMESHIFT-DEL	c.1271delA	p.Asn426ThrfsTer14	NA	NA	chr12*113442830*113442830*A*del*1*1*Esophageal_cancer	chr12*113442829*113442830*COSM1239661*CA*C*3*oesophagus
chr12	1.2E+08	AC	A	HSPB8	FRAMESHIFT-DEL	c.260delC	p.Pro89HisfsTer12	NA	NA	chr12*119617377*119617377*C*del*1*1*Colorectal_cancer	chr12*119617376*119617377*COSM1359246*AC*A*1*large_intestine*1|chr12*119617376*119617377*COSM1359247*AC*A*1*large_intestine*1

<table>
<tr><td>chr12</td><td>1.21E+08</td><td>GC</td><td>G</td><td>HNF1A</td><td>FRAMESHIFT-DEL</td><td>c.865delC</td><td>p.Pro291GlnfsTer51</td><td>OTHER:GERMLINE</td><td>PATHOGENIC|PATHOGENIC|PATHOGENIC|PATHOGENIC|PATHOGENIC:BENIGN</td><td>NA</td><td>chr12*121432116*121432117*ins*C*1*1*Breast_cancer|chr12*121432117*121432117*ins*C*2*1*Stomach_(gastric)_cancer|chr12*121432117*121432117*G*T*1*1*Brain_cancer|chr12*121432118*121432118*C*del*1*1*Stomach_(gastric)_cancer</td></tr>
<tr><td>chr12</td><td>1.25E+08</td><td>TG</td><td>T</td><td>NCOR2</td><td>FRAMESHIFT-DEL</td><td>c.3921delC</td><td>p.Lys1308SerfsTer6</td><td>NA</td><td>NA</td><td>NA</td><td>NA</td></tr>
<tr><td>chr12</td><td>1.25E+08</td><td>GC</td><td>G</td><td>DHX37</td><td>FRAMESHIFT-DEL</td><td>c.3090delG</td><td>p.Arg1031GlyfsTer62</td><td>NA</td><td>NA</td><td>NA</td><td>NA</td></tr>
<tr><td>chr15</td><td>22855175</td><td>C</td><td>T</td><td>TUBGCP5</td><td>MISSENSE</td><td>c.1636C>T</td><td>p.His546Tyr</td><td>NA</td><td>NA</td><td>NA</td><td>NA</td></tr>
<tr><td>chr15</td><td>22962457</td><td>G</td><td>A</td><td>CYFIP1</td><td>MISSENSE</td><td>c.2177G>A</td><td>p.Arg726Gln</td><td>NA</td><td>NA</td><td>NA</td><td>NA</td></tr>
<tr><td>chr15</td><td>26806222</td><td>A</td><td>G</td><td>GABRB3</td><td>MISSENSE</td><td>c.937T>C</td><td>p.Cys313Arg</td><td>NA</td><td>NA</td><td>NA</td><td>NA</td></tr>
<tr><td>chr15</td><td>27160003</td><td>C</td><td>T</td><td>GABRA5</td><td>MISSENSE</td><td>c.551C>T</td><td>p.Ala184Val</td><td>NA</td><td>NA</td><td>chr15*27160003*27160003*C*T*1*1*Colorectal_cancer|chr15*27160003*27160003*C*A*1*1*Lung_cancer</td><td>chr15*27160003*27160003*COSM1372193*C*T*1*large_intestine*1|chr15*27160003*27160003*COSM554587*C*A*1*lung*1</td></tr>
<tr><td>chr15</td><td>33955073</td><td>C</td><td>T</td><td>RYR3</td><td>MISSENSE</td><td>c.5342C>T</td><td>p.Ala1781Val</td><td>NA</td><td>NA</td><td>NA</td><td>NA</td></tr>
<tr><td>chr15</td><td>34531186</td><td>C</td><td>T</td><td>SLC12A6</td><td>MISSENSE</td><td>c.2612G>A</td><td>p.Arg871His</td><td>NA</td><td>NA</td><td>NA</td><td>NA</td></tr>
<tr><td>chr15</td><td>40915500</td><td>G</td><td>A</td><td>CASC5</td><td>MISSENSE</td><td>c.3116G>A</td><td>p.Ser1039Asn</td><td>NA</td><td>NA</td><td>NA</td><td>NA</td></tr>
<tr><td>chr15</td><td>45392073</td><td>G</td><td>A</td><td>DUOX2</td><td>MISSENSE</td><td>c.3202C>T</td><td>p.Pro1068Ser</td><td>NA</td><td>NA</td><td>NA</td><td>NA</td></tr>
<tr><td>chr15</td><td>51791325</td><td>C</td><td>T</td><td>DMXL2</td><td>MISSENSE</td><td>c.4096G>A</td><td>p.Ala1366Thr</td><td>NA</td><td>NA</td><td>NA</td><td>NA</td></tr>
<tr><td>chr15</td><td>54919088</td><td>G</td><td>A</td><td>UNC13C</td><td>MISSENSE</td><td>c.6422G>A</td><td>p.Cys2141Tyr</td><td>NA</td><td>NA</td><td>NA</td><td>NA</td></tr>
<tr><td>chr15</td><td>62211501</td><td>C</td><td>T</td><td>VPS13C</td><td>MISSENSE</td><td>c.7625G>A</td><td>p.Arg2542His</td><td>NA</td><td>NA</td><td>chr15*62211501*62211501*C*T*1*1*Endometrial_cancer</td><td>chr15*62211501*62211501*COSM963581*C*T*2*large_intestine</td></tr>
<tr><td>chr15</td><td>63944674</td><td>G</td><td>A</td><td>HERC1</td><td>MISSENSE</td><td>c.10357C>T</td><td>p.Arg3453Cys</td><td>NA</td><td>NA</td><td>NA</td><td>NA</td></tr>
<tr><td>chr15</td><td>64056374</td><td>C</td><td>T</td><td>HERC1</td><td>MISSENSE</td><td>c.947G>A</td><td>p.Arg316Gln</td><td>NA</td><td>NA</td><td>NA</td><td>NA</td></tr>
<tr><td>chr15</td><td>73879883</td><td>C</td><td>T</td><td>NPTN</td><td>MISSENSE</td><td>c.688G>A</td><td>p.Ala230Thr</td><td>NA</td><td>NA</td><td>chr15*73879883*73879883*G*A*5*5*Colorectal_cancer</td><td>chr15*73879883*73879883*COSM120973*C*T*4*large_intestine</td></tr>
<tr><td>chr15</td><td>78911140</td><td>G</td><td>A</td><td>CHRNA3</td><td>MISSENSE</td><td>c.200C>T</td><td>p.Ser67Phe</td><td>NA</td><td>NA</td><td>NA</td><td>NA</td></tr>
<tr><td>chr15</td><td>81187442</td><td>C</td><td>T</td><td>KIAA1199</td><td>MISSENSE</td><td>c.1198C>T</td><td>p.Arg400Trp</td><td>NA</td><td>NA</td><td>NA</td><td>NA</td></tr>
<tr><td>chr15</td><td>95013653</td><td>T</td><td>C</td><td>MCTP2</td><td>MISSENSE</td><td>c.2452T>C</td><td>p.Tyr818His</td><td>NA</td><td>NA</td><td>NA</td><td>NA</td></tr>
<tr><td>chr15</td><td>1.02E+08</td><td>G</td><td>A</td><td>LRRK1</td><td>MISSENSE</td><td>c.3424G>A</td><td>p.Asp1142Asn</td><td>NA</td><td>NA</td><td>chr15*101589973*101589973*G*A*1*1*Head_and_neck_cancer</td><td>NA</td></tr>
<tr><td>chr15</td><td>23021238</td><td>CT</td><td>C</td><td>NIPA2</td><td>FRAMESHIFT-DEL</td><td>c.98delA</td><td>p.Lys33ArgfsTer12</td><td>NA</td><td>NA</td><td>chr15*23021239*23021239*T*del*1*1*Stomach_(gastric)_cancer</td><td>chr15*23021238*23021238*COSM1179596*C*T*1*prostate*1|chr15*23021238*23021238*COSM1372050*C*CT*2*large_intestine*2|chr15*23021238*23021239*COSM1372051*CT*C*7*large_intestine*7</td></tr>
</table>

chr15	28200304	CT	C	OCA2	FRAMESHIFT-DEL-SS-PRX	c.1841delA	p.Lys614SerfsTer14	NA	NA	chr15*28200305*28200305*T*del*2*2*Stomach_(gastric)_cancer	chr15*28200304*28200305*COSM1372216*CT*C*2*large_intestine*2
chr15	34549915	TA	T	SLC12A6	FRAMESHIFT-DEL	c.617delT	p.Leu206TyrfsTer28	NA	NA	chr15*34549916*34549916*A*del*1*1*Stomach_(gastric)_cancer	chr15*34549915*34549916*COSM1372503*TA*T*3*large_intestine*3|chr15*34549915*34549916*COSM1372504*TA*T*3*large_intestine*3
chr15	40558459	GC	G	PAK6	FRAMESHIFT-DEL	c.622delC	p.Thr210ArgfsTer8	NA	NA	chr15*40558460*40558460*C*del*1*1*Colorectal_cancer	chr15*40558459*40558460*COSM1372609*GC*G*1*large_intestine*1
chr15	54685301	AT	A	UNC13C	FRAMESHIFT-DEL	c.4770delT	p.Trp1592GlyfsTer7	NA	NA	chr15*54685302*54685302*T*del*1*1*Colorectal_cancer	chr15*54685301*54685302*COSM1373580*AT*A*1*large_intestine*1|chr15*54685301*54685302*COSM1373579*AT*A*1*large_intestine*1
chr15	55652567	CT	C	CCPG1	FRAMESHIFT-DEL	c.1403delA	p.Lys468ArgfsTer29	NA	NA	NA	NA
chr15	91425047	CAG	C	FURIN	FRAMESHIFT-DEL	c.2325_2326delAG	p.Glu776ArgfsTer99	NA	NA	chr15*91425048*91425048*A*T*1*1*Lung_cancer	chr15*91425048*91425048*COSM702115*A*T*1*lung*1
chrX	1419478	G	A	CSF2RA	MISSENSE	c.905G>A	p.Arg302His	NA	NA	NA	NA
chrX	9660220	G	A	TBL1X	MISSENSE	c.817G>A	p.Val273Met	NA	NA	NA	NA
chrX	19380919	G	A	MAP3K15	MISSENSE	c.3616C>T	p.Arg1206Trp	NA	NA	chrX*19380919*19380919*C*A*1*1*Lung_cancer	chrX*19380919*19380919*COSM355485*G*T*1*lung*1|chrX*19380919*19380919*COSM355484*G*T*1*lung*1
chrX	24082419	C	A	EIF2S3	MISSENSE	c.739C>A	p.Pro247Thr	NA	NA	NA	NA
chrX	47307610	T	C	ZNF41	MISSENSE	c.1559A>G	p.Glu520Gly	NA	NA	NA	NA
chrX	48674571	G	A	HDAC6	MISSENSE	c.1517G>A	p.Arg506His	NA	NA	NA	NA
chrX	69424892	A	G	DGAT2L6	MISSENSE	c.950A>G	p.Lys317Arg	NA	NA	NA	NA
chrX	71864265	T	C	PHKA1	MISSENSE	c.1406A>G	p.Tyr469Cys	NA	NA	NA	NA
chrX	91132023	G	A	PCDH11X	MISSENSE	c.784G>A	p.Ala262Thr	NA	NA	NA	NA
chrX	1.1E+08	T	C	PAK3	MISSENSE	c.752T>C	p.Val251Ala	NA	NA	NA	NA
chrX	1.11E+08	C	T	TRPC5	MISSENSE	c.2105G>A	p.Arg702His	NA	NA	NA	NA
chrX	1.2E+08	G	A	ATP1B4	MISSENSE	c.881G>A	p.Arg294His	NA	NA	NA	NA
chrX	1.3E+08	C	T	IGSF1	MISSENSE	c.2728G>A	p.Ala910Thr	NA	NA	chrX*130410118*130410118*C*T*1*1*Endometrial_cancer	chrX*130410118*130410118*COSM1115226*C*T*1*endometrium*1
chrX	1.53E+08	C	T	NAA10	MISSENSE	c.694G>A	p.Asp232Asn	NA	NA	NA	NA
chrX	46433887	CG	C	CHST7	FRAMESHIFT-DEL	c.522delG	p.Asp176ThrfsTer96	NA	NA	chrX*46433887*46433887*C*T*1*1*Bladder_cancer	chrX*46433887*46433887*COSM1315525*C*T*1*urinary_tract*1
chrX	54472571	CG	C	FGD1	FRAMESHIFT-DEL	c.2856delC	p.Glu953AsnfsTer28	NA	NA	NA	NA

chrX	70472962	AG	A	ZMYM3	FRAMESHIFT-DEL	c.143delC	p.Pro48LeufsTer65	NA	NA	chrX*70472962*70472962*A*T*1*1*Lung_cancer|chrX*70472962*70472963*ins*GA*1*1*Non-Hodgkin_lymphoma|chrX*70472962*70472963*ins*G*1*1*Prostate_cancer|chrX*70472962*70472963*ins*C*1*1*Neuroblastoma|chrX*70472963*70472963*G*del*1*1*Pancreatic_cancer	chrX*70472962*70472962*COSM1178926*A*AG*2*autonomic_ganglia
chrX	99662504	CG	C	PCDH19	FRAMESHIFT-DEL	c.1091delC	p.Pro364ArgfsTer4	PATHOGENIC|PATHOGENIC	Early_infantile_epileptic_encephalopathy_9	not_provided	chrX*99662505*99662505*G*del*2*2*Stomach_(gastric)_cancer|chrX*99662505*99662505*C*del*1*1*Colon_cancer
chrX	1.01E+08	AT	A	TAF7L	FRAMESHIFT-DEL	c.1337delA	p.Asn446MetfsTer15	NA	NA	chrX*100530217*100530217*T*del*1*1*Stomach_(gastric)_cancer	NA
chrX	1.35E+08	T	TG	MAP7D3	FRAMESHIFT-INS	c.922dupC	p.Gln308ProfsTer25	NA	NA	chrX*135314193*135314193*ins*G*1*1*Bladder_cancer	chrX*135314193*135314193*COSM1465812*T*TG*1*large_intestine*1|chrX*135314193*135314194*COSM1465813*TG*T*3*NS
chr4	3219666	G	A	HTT	MISSENSE	c.7229G>A	p.Arg2410His	NA	NA	NA	NA
chr4	3237061	C	T	HTT	MISSENSE	c.8507C>T	p.Ala2836Val	NA	NA	NA	NA
chr4	3237102	G	A	HTT	MISSENSE	c.8548G>A	p.Glu2850Lys	NA	NA	NA	NA
chr4	15780159	T	C	CD38	MISSENSE	c.122T>C	p.Val41Ala	NA	NA	NA	NA
chr4	47667154	G	T	CORIN	MISSENSE	c.1484C>A	p.Pro495His	NA	NA	chr4*47667154*47667154*C*A*1*1*Lung_cancer	chr4*47667154*47667154*COSM310286*G*T*1*lung*1
chr4	71347425	A	G	MUC7	MISSENSE	c.964A>G	p.Thr322Ala	NA	NA	NA	NA
chr4	71508032	G	A	ENAM	MISSENSE	c.889G>A	p.Ala297Thr	NA	NA	NA	NA
chr4	79240065	G	A	FRAS1	MISSENSE	c.2062G>A	p.Val688Met	NA	NA	NA	NA
chr4	85414615	C	T	NKX6-1	MISSENSE	c.931G>A	p.Glu311Lys	NA	NA	NA	NA
chr4	88766450	G	A	MEPE	MISSENSE	c.430G>A	p.Ala144Thr	NA	NA	chr4*88766450*88766450*G*A*3*3*Brain_cancer	chr4*88766450*88766450*COSM261977*G*A*1*large_intestine*1
chr4	88903888	A	G	SPP1	MISSENSE	c.785A>G	p.Asp262Gly	NA	NA	NA	NA

chr4	96051097	C	T	BMPR1B	MISSENSE	c.760C>T	p.Arg254Cys	NA	NA	chr4*96051097*96051097*C*T*1*1*Colorectal_cancer	chr4*96051097*96051097*COSM3696786*C*T*1*large_intestine*1|chr4*96051097*96051097*COSM3696785*C*T*1*large_intestine*1|chr4*96051097*96051097*COSM3696784*C*T*1*large_intestine*1
chr4	1.04E+08	A	T	CENPE	MISSENSE	c.4561T>A	p.Leu1521Ile	NA	NA	NA	NA
chr4	1.14E+08	G	A	ANK2	MISSENSE	c.4555G>A	p.Ala1519Thr	LIKELY_BENIGN	not_provided	chr4*114274329*114274329*G*A*2*2*Colorectal_cancer	chr4*114274329*114274329*COSM201680*G*A*1*large_intestine*1
chr4	1.14E+08	A	G	ANK2	MISSENSE	c.5305A>G	p.Lys1769Glu	NA	NA	NA	NA
chr4	1.24E+08	C	T	SPATA5	MISSENSE	c.887C>T	p.Ala296Val	NA	NA	NA	NA
chr4	1.26E+08	C	T	FAT4	MISSENSE	c.341C>T	p.Ala114Val	NA	NA	NA	NA
chr4	1.3E+08	C	T	SCLT1	MISSENSE	c.118G>A	p.Gly40Arg	NA	NA	NA	NA
chr4	1.4E+08	T	A	NAA15	MISSENSE	c.124T>A	p.Cys42Ser	NA	NA	NA	NA
chr4	1.44E+08	A	T	GAB1	MISSENSE	c.1084A>T	p.Ile362Phe	NA	NA	NA	NA
chr4	1.47E+08	G	A	MMAA	MISSENSE	c.730G>A	p.Val244Ile	NA	NA	NA	NA
chr4	1.52E+08	C	T	MAB21L2	MISSENSE	c.481C>T	p.Arg161Cys	NA	NA	NA	NA
chr4	1.55E+08	C	T	DCHS2	MISSENSE	c.4111G>A	p.Ala1371Thr	NA	NA	chr4*155225950*155225950*G*A*1*1*Colon_cancer|chr4*155225950*155225950*C*T*1*1*Colorectal_cancer	chr4*155225950*155225950*COSM206951*C*T*1*large_intestine*1
chr4	1.66E+08	G	A	CPE	MISSENSE	c.418G>A	p.Glu140Lys	NA	NA	NA	NA
chr4	1.78E+08	C	T	VEGFC	MISSENSE	c.439G>A	p.Ala147Thr	NA	NA	chr4*177649045*177649045*G*A*1*1*Colorectal_cancer	chr4*177649045*177649045*COSM3602191*C*T*1*large_intestine*1
chr4	1.86E+08	G	A	CASP3	MISSENSE	c.301C>T	p.Arg101Cys	NA	NA	chr4*185553428*185553428*G*A*1*1*Stomach_(gastric)_cancer	NA
chr4	1.87E+08	C	T	MTNR1A	MISSENSE	c.275G>A	p.Gly92Glu	NA	NA	NA	NA
chr4	3495165	AC	A	DOK7	FRAMESHIFT-DEL	c.1453delC	p.Pro486ArgfsTer15	NA	NA	NA	NA
chr4	5586485	C	CT	EVC2	FRAMESHIFT-INS	c.2921dupA	p.Ala975GlyfsTer29	NA	NA	NA	NA
chr4	38774955	CT	C	TLR10	FRAMESHIFT-DEL	c.2256delA	p.Ala753HisfsTer28	NA	NA	chr4*38774956*38774956*A*del*4*1*Colorectal_cancer	chr4*38774955*38774956*COSM1429552*CT*C*4*large_intestine*4
chr4	70599152	CA	C	SULT1B1	FRAMESHIFT-DEL	c.575delT	p.Leu192CysfsTer6	NA	NA	chr4*70599153*70599153*T*del*1*1*Colorectal_cancer	chr4*70599152*70599153*COSM1430716*CA*C*2*NS
chr4	95173909	T	TA	SMARCAD1	FRAMESHIFT-INS	c.1032_1033insA	p.Asn347LysfsTer4	NA	NA	NA	chr4*95173909*95173910*COSM1431835*TA*T*8*large_intestine*8
chr4	1.14E+08	TA	T	LARP7	FRAMESHIFT-DEL	c.1227delA	p.Thr412GlnfsTer5	NA	NA	chr4*113570754*113570754*A*del*5*4*Stomach_(gastric)_cancer	chr4*113570753*113570754*COSM266809*TA*T*2*lung

chr4	1.44E+08	TC	T	GAB1	FRAMESHIFT-DEL	c.44delC	p.Pro17ArgfsTer5	NA	NA	chr4*144258384*144258385*ins*C*1*1*Colorectal_cancer|chr4*144258385*144258385*C*del*2*1*Colorectal_cancer	chr4*144258384*144258384*COSM1427308*T*TC*1*large_intestine*1|chr4*144258384*144258385*COSM1427309*TC*T*2*large_intestine*2
chr4	1.52E+08	AT	A	LRBA	FRAMESHIFT-DEL	c.303delA	p.Lys101AsnfsTer18	NA	NA	NA	NA
chr4	1.86E+08	GC	G	PDLIM3	FRAMESHIFT-DEL	c.992delG	p.Gly331AlafsTer5	BENIGN|BENIGN|BENIGN	not_specified	Hypertrophic_cardiomyopathy	Primary_dilated_cardiomyopathy
chr4	1.87E+08	CT	C	CYP4V2	FRAMESHIFT-DEL	c.565delT	p.Tyr191ThrfsTer7	NA	NA	chr4*187118245*187118245*T*del*3*1*Colorectal_cancer	chr4*187118244*187118245*COSM1428957*CT*C*3*large_intestine*3
chr2	1652010	G	A	PXDN	MISSENSE	c.3542C>T	p.Ala1181Val	NA	NA	chr2*1652010*1652010*G*A*2*2*Stomach_(gastric)_cancer	chr2*1652010*1652010*COSM441335*G*A*1*breast*1
chr2	6991735	G	A	CMPK2	MISSENSE	c.1072C>T	p.Pro358Ser	NA	NA	NA	NA
chr2	9533650	C	T	ASAP2	MISSENSE	c.2558C>T	p.Thr853Met	NA	NA	NA	NA
chr2	11337711	G	A	ROCK2	MISSENSE	c.3220C>T	p.Arg1074Cys	NA	NA	chr2*11337711*11337711*G*A*1*1*Head_and_neck_cancer	NA
chr2	16085885	C	T	MYCN	MISSENSE	c.1061C>T	p.Ala354Val	NA	NA	NA	NA
chr2	29473986	G	A	ALK	MISSENSE	c.2189C>T	p.Ala730Val	NA	NA	NA	NA
chr2	31609310	C	T	XDH	MISSENSE	c.763G>A	p.Ala255Thr	NA	NA	NA	NA
chr2	32693596	C	T	BIRC6	NONSENSE	c.5872C>T	p.Arg1958Ter	NA	NA	NA	NA
chr2	44970762	G	A	CAMKMT	MISSENSE-SS-PRX	c.625G>A	p.Val209Ile	NA	NA	NA	NA
chr2	47637243	G	A	MSH2	MISSENSE	c.377G>A	p.Gly126Asp	NA	NA	NA	NA
chr2	51255153	C	T	NRXN1	MISSENSE	c.259G>A	p.Gly87Ser	NA	NA	NA	NA
chr2	61275624	C	T	PEX13	MISSENSE	c.931C>T	p.Arg311Cys	NA	NA	chr2*61275624*61275624*C*T*2*2*Colorectal_cancer	chr2*61275624*61275624*COSM289749*C*T*1*large_intestine*1
chr2	61475814	A	C	USP34	MISSENSE	c.6226T>G	p.Cys2076Gly	NA	NA	NA	NA
chr2	71576278	G	A	ZNF638	MISSENSE	c.194G>A	p.Gly65Glu	NA	NA	NA	NA
chr2	71781056	C	T	DYSF	MISSENSE	c.2104C>T	p.Arg702Trp	NA	NA	chr2*71781056*71781056*C*T*1*1*Esophageal_cance	NA
chr2	73456017	C	T	PRADC1	MISSENSE	c.352G>A	p.Ala118Thr	NA	NA	NA	NA
chr2	74760026	G	A	HTRA2	MISSENSE	c.1291G>A	p.Val431Ile	NA	NA	NA	NA
chr2	95847717	C	T	ZNF2	MISSENSE	c.1144C>T	p.Arg382Trp	NA	NA	NA	NA
chr2	1.02E+08	C	T	MAP4K4	MISSENSE	c.1222C>T	p.Arg408Trp	NA	NA	chr2*102460762*102460762*ins*GGCTAGAA*1*1*Endometrial_cancer|chr2*102460762*102460763*ins*GGCTAGAA*1*1*Endometrial_carcinoma	chr2*102460762*102460762*COSM1004591*C*CGGCTAGAA*1*endometrium*1
chr2	1.02E+08	C	T	MAP4K4	NONSENSE	c.1693C>T	p.Arg565Ter	NA	NA	NA	NA
chr2	1.06E+08	C	T	TGFBRAP1	MISSENSE	c.100G>A	p.Gly34Ser	NA	NA	NA	NA
chr2	1.13E+08	C	T	ANAPC1	MISSENSE	c.3686G>A	p.Ser1229Asn	NA	NA	NA	NA
chr2	1.22E+08	T	C	CLASP1	MISSENSE	c.4256A>G	p.His1419Arg	NA	NA	NA	NA
chr2	1.37E+08	G	A	LCT	MISSENSE	c.1568C>T	p.Ala523Val	NA	NA	NA	NA
chr2	1.42E+08	C	A	LRP1B	MISSENSE-SS-PRX	c.2968G>T	p.Asp990Tyr	NA	NA	NA	NA
chr2	1.6E+08	G	T	TANC1	MISSENSE-SS-PRX	c.1069G>T	p.Ala357Ser	NA	NA	NA	NA

chr2	1.7E+08	G	A	LRP2	MISSENSE	c.1243C>T	p.Arg415Trp	NA	NA	NA	NA
chr2	1.77E+08	T	C	HOXD10	MISSENSE	c.391T>C	p.Tyr131His	NA	NA	NA	NA
chr2	1.79E+08	C	T	PDE11A	MISSENSE	c.2320G>A	p.Ala774Thr	NA	NA	NA	NA
chr2	1.79E+08	T	C	TTN	MISSENSE	c.86787A>G	p.Ile28929Met	NA	NA	NA	NA
chr2	1.79E+08	G	A	TTN	MISSENSE	c.78017C>T	p.Ser26006Phe	NA	NA	NA	NA
chr2	1.79E+08	C	T	TTN	MISSENSE	c.54644G>A	p.Arg18215His	NA	NA	NA	NA
chr2	1.79E+08	C	T	TTN	MISSENSE	c.53660G>A	p.Arg17887His	NA	NA	NA	NA
chr2	1.79E+08	C	A	TTN	NONSENSE	c.53170G>T	p.Gly17724Te	NA	NA	NA	NA
chr2	1.8E+08	C	A	TTN	MISSENSE	c.30479G>T	p.Arg10160Ile	NA	NA	NA	NA
chr2	1.8E+08	C	T	TTN	MISSENSE	c.5669G>A	p.Arg1890His	NA	NA	NA	NA
chr2	1.86E+08	A	C	ZNF804A	MISSENSE	c.534A>C	p.Lys178Asn	NA	NA	NA	NA
chr2	1.88E+08	A	G	TFPI	MISSENSE	c.646T>C	p.Trp216Arg	NA	NA	NA	NA
chr2	2.12E+08	G	A	CPS1	MISSENSE	c.4100G>A	p.Gly1367Asp	NA	NA	NA	NA
chr2	2.16E+08	C	T	BARD1	MISSENSE	c.2198G>A	p.Cys733Tyr	NA	NA	NA	NA
chr2	2.24E+08	G	A	SCG2	MISSENSE	c.680C>T	p.Thr227Met	NA	NA	NA	NA
chr2	2.24E+08	T	C	SCG2	MISSENSE	c.611A>G	p.Glu204Gly	NA	NA	NA	NA
chr2	2.28E+08	C	T	COL4A3	NONSENSE	c.2482C>T	p.Gln828Ter	NA	NA	NA	NA
chr2	2.33E+08	G	A	CHRNG	MISSENSE	c.275G>A	p.Arg92Gln	NA	NA	NA	NA
chr2	2.34E+08	G	A	INPP5D	MISSENSE	c.1073G>A	p.Arg358Gln	NA	NA	NA	NA
chr2	2.42E+08	G	A	KIF1A	MISSENSE	c.1862C>T	p.Thr621Met	UNCERTAIN_SIGNIFICANCE	not_specified	chr2*241702670*241702670*G*A*1*1*Bladder_cancer	chr2*241702670*241702670*COSM418792*G*A*1*urinary_tract*1
chr2	2.43E+08	C	T	PDCD1	MISSENSE	c.148G>A	p.Ala50Thr	NA	NA	NA	NA
chr2	1680759	AC	A	PXDN	FRAMESHIFT-DEL	c.787delG	p.Val263CysfsTer24	NA	NA	NA	NA
chr2	9528642	GC	G	ASAP2	FRAMESHIFT-DEL	c.2351delC	p.Pro786ArgfsTer25	NA	NA	chr2*9528642*9528643*ins*C*1*1*Colorectal_cancer|chr2*9528643*9528643*C*del*2*2*Stomach_(gastric)_cancer	chr2*9528642*9528642*COSM1409941*G*GC*2*large_intestine
chr2	27603371	TG	T	ZNF513	FRAMESHIFT-DEL	c.22delC	p.His8ThrfsTer5	NA	NA	NA	NA
chr2	31610691	GA	G	XDH	FRAMESHIFT-DEL	c.636delT	p.Pro214GlnfsTer4	NA	NA	NA	NA
chr2	44145164	CTT	C	LRPPRC	FRAMESHIFT-DEL-SS-PRX	c.3146_3147delAA	p.Lys1049ArgfsTer4	PATHOGENIC	Leigh_syndrome_French_Canadian_type	chr2*44145165*44145165*T*del*4*2*Stomach_(gastric)_cancer	chr2*44145164*44145165*COSM1408154*CT*C*5*pancreas
chr2	48030639	A	AC	MSH6	FRAMESHIFT-INS	c.3253_3254insC	p.Phe1088LeufsTer5	PATHOGENIC|PATHOGENIC|PATHOGENIC:PATHOGENIC|PATHOGENIC|PATHOGENIC:PATHOGENIC|PATHOGENIC|PATHOGENIC:PATHOGENIC|PATHOGENIC|PATHOGENIC	Lynch_syndrome	not_provided	Hereditary_cancer-predisposing_syndrome:Lynch_syndrome
chr2	48808314	CT	C	STON1-GTF2A1L	FRAMESHIFT-DEL	c.543delT	p.Ser183GlnfsTer39	NA	NA	NA	NA
chr2	48808314	CT	C	STON1	FRAMESHIFT-DEL	c.543delT	p.Ser183GlnfsTer39	NA	NA	NA	NA
chr2	48873947	A	AT	STON1-GTF2A1L	FRAMESHIFT-INS	c.2856_2857insT	p.Ser954PhefsTer10	NA	NA	NA	NA
chr2	48873947	A	AT	GTF2A1L	FRAMESHIFT-INS	c.744_745insT	p.Ser250PhefsTer10	NA	NA	NA	NA
chr2	97526593	GC	G	SEMA4C	FRAMESHIFT-DEL	c.2271delG	p.Pro759LeufsTer36	NA	NA	NA	NA
chr2	97529758	AG	A	SEMA4C	FRAMESHIFT-DEL	c.1234delC	p.Leu412CysfsTer3	NA	NA	chr2*97529759*97529759*G*del*1*1*Stomach_(gastric)_cancer	NA
chr2	99778492	T	TA	LIPT1	FRAMESHIFT-INS	c.72_73insA	p.Thr27AsnfsTer14	NA	NA	NA	NA
chr2	1.32E+08	GC	G	ARHGEF4	FRAMESHIFT-DEL	c.1672delC	p.Pro558LeufsTer36	NA	NA	NA	NA

chr2	1.42E+08	GT	G	LRP1B	FRAMESHIFT-DEL	c.2561delA	p.Asn854ThrfsTer18	NA	NA	NA	NA
chr2	1.49E+08	TA	T	ACVR2A	FRAMESHIFT-DEL	c.1303delA	p.Lys437ArgfsTer5	NA	NA	chr2*148683685*148683685*ins*A*1*1*Lung_cancer|chr2*148683685*148683685*T*del*1*1*Colorectal_cancer|chr2*148683685*148683686*ins*A*1*1*Colorectal_cancer|chr2*148683686*148683686*ins*A*1*1*Biliary_tract_cancer|chr2*148683686*148683686*A*del*38*10*Stomach_(gastric)_cancer|chr2*148683686*148683687*ins*A*1*1*Liver_cancer|chr2*148683686*148683693*AAAAAAAA*del*17*2*Colon_cancer|chr2*148683686*148683693*AAA	chr2*148683668*148683688*COSM1613469*BigSeq*C*1*liver*1|chr2*148683684*148683685*COSM1645195*AT*A*1*large_intestine*1|chr2*148683685*148683685*COSM1399832*T*TA*1*large_intestine*1|chr2*148683685*148683686*COSM252949*TA*T*21*large_intestine
chr2	1.66E+08	T	TAAAAAAA	SCN2A	FRAMESHIFT-INS	c.5755_5756insAAAAAAA	p.Tyr1919Ter	NA	NA	NA	NA
chr2	1.66E+08	ACCTCTTG	A	SCN2A	FRAMESHIFT-DEL	c.5757_5763delCCTCTTG	p.Tyr1919Ter	BENIGN	not_specified	NA	NA
chr2	1.7E+08	TG	T	LRP2	FRAMESHIFT-DEL	c.13139delC	p.Pro4380HisfsTer46	PATHOGENIC:LIKELY_PATHOGENIC	Donnai_Barrow_syndrome:Donnai_Barrow_syndrome	chr2*169997024*169997024*ins*G*1*1*Breast_cancer|chr2*169997024*169997025*ins*C*1*1*Liver_cancer|chr2*169997025*169997025*G*del*2*2*Stomach_(gastric)_cancer	chr2*169997024*169997024*COSM1401024*T*TG*4*liver
chr2	1.73E+08	GA	G	ITGA6	FRAMESHIFT-DEL	c.3227delA	p.Lys1078AsnfsTer48	NA	NA	chr2*173368931*173368931*A*del*1*1*Stomach_(gastric)_cancer	chr2*173368930*173368931*COSM1401310*GA*G*2*liver
chr2	1.8E+08	AG	A	TTN	FRAMESHIFT-DEL	c.40214delC	p.Pro13405LeufsTer27	UNCERTAIN_SIGNIFICANCE	not_provided	NA	NA
chr2	1.97E+08	CT	C	DNAH7	FRAMESHIFT-DEL	c.9018delA	p.Ala3007ProfsTer6	NA	NA	NA	NA
chr2	2.03E+08	GA	G	BMPR2	FRAMESHIFT-DEL	c.1742delA	p.Asn583ThrfsTer44	NA	NA	chr2*203420130*203420130*A*del*11*6*Stomach_(gastric)_cancer	chr2*203420129*203420130*COSM1180896*GA*G*18*large_intestine
chr2	2.19E+08	CT	C	SLC11A1	FRAMESHIFT-DEL	c.591delT	p.Phe199LeufsTer5	NA	NA	chr2*219252307*219252307*T*del*2*1*Lung_cancer	chr2*219252306*219252307*COSM391196*CT*C*3*lung
chr2	2.2E+08	GC	G	OBSL1	FRAMESHIFT-DEL	c.4664delG	p.Gly1555AlafsTer14	NA	NA	NA	NA
chr2	2.24E+08	TG	T	KCNE4	FRAMESHIFT-DEL	c.239delG	p.Glu82ArgfsTer3	NA	NA	chr2*223917787*223917787*G*del*1*1*Stomach_(gastric)_cancer	chr2*223917786*223917787*COSM1405822*TG*T*1*large_intestine*1
chr2	2.28E+08	GC	G	IRS1	FRAMESHIFT-DEL	c.2114delG	p.Gly705AlafsTer29	NA	NA	NA	NA

chr2	2.38E+08	T	TG	PRLH	FRAMESHIFT-INS	c.149_150insG	p.Arg52ProfsTer?	NA	NA	NA	NA
chr9	8517952	C	T	PTPRD	MISSENSE	c.1439G>A	p.Gly480Asp	NA	NA	chr9*8517952*8517952*G*C*1*1*Lung_cancer	NA
chr9	19058637	G	A	HAUS6	NONSENSE	c.2128C>T	p.Arg710Ter	NA	NA	NA	NA
chr9	35380501	C	T	UNC13B	MISSENSE	c.1993C>T	p.Arg665Cys	NA	NA	chr9*35380501*35380501*C*T*4*3*Stomach_(gastric)_cancer	chr9*35380501*35380501*COSM1108632*C*T*4*endometrium
chr9	35616683	C	T	CD72	MISSENSE	c.266G>A	p.Arg89His	NA	NA	NA	NA
chr9	35792436	G	A	NPR2	MISSENSE	c.31G>A	p.Ala11Thr	NA	NA	NA	NA
chr9	36246468	C	T	GNE	MISSENSE	c.269G>A	p.Arg90Gln	NA	NA	NA	NA
chr9	73240148	G	A	TRPM3	MISSENSE	c.1732C>T	p.Arg578Cys	NA	NA	chr9*73240148*73240148*G*A*1*1*Colorectal_cancer|chr9*73240148*73240148*C*T*1*1*Colon_cancer	chr9*73240148*73240148*COSM265873*G*A*1*large_intestine*1|chr9*73240148*73240148*COSM265874*G*A*1*large_intestine*1
chr9	86495385	T	C	KIF27	MISSENSE	c.2470A>G	p.Ser824Gly	NA	NA	NA	NA
chr9	91617031	C	T	S1PR3	MISSENSE	c.916C>T	p.Arg306Trp	NA	NA	chr9*91617031*91617031*C*T*1*1*Breast_cancer	NA
chr9	98678619	G	A	ERCC6L2	MISSENSE	c.1094G>A	p.Arg365Gln	NA	NA	NA	NA
chr9	1.01E+08	G	A	ANKS6	MISSENSE	c.2612C>T	p.Ala871Val	NA	NA	chr9*101498805*101498805*C*T*2*2*Colorectal_cancer	chr9*101498805*101498805*COSM1182988*G*A*1*large_intestine*1
chr9	1.03E+08	C	T	NR4A3	MISSENSE	c.1790C>T	p.Ala597Val	NA	NA	NA	NA
chr9	1.08E+08	T	C	ABCA1	MISSENSE	c.2309A>G	p.Tyr770Cys	NA	NA	NA	NA
chr9	1.14E+08	C	T	LPAR1	MISSENSE	c.477G>A	p.Met159Ile	NA	NA	NA	NA
chr9	1.17E+08	G	A	COL27A1	MISSENSE	c.994G>A	p.Ala332Thr	NA	NA	NA	NA
chr9	1.17E+08	C	T	DFNB31	MISSENSE	c.2323G>A	p.Ala775Thr	NA	NA	NA	NA
chr9	1.19E+08	G	A	TRIM32	MISSENSE	c.134G>A	p.Arg45His	NA	NA	NA	NA
chr9	1.19E+08	C	T	ASTN2	MISSENSE	c.2510G>A	p.Ser837Asn	NA	NA	NA	NA
chr9	1.24E+08	G	A	GSN	MISSENSE	c.934G>A	p.Val312Ile	NA	NA	NA	NA
chr9	1.25E+08	G	A	OR1J1	MISSENSE	c.461C>T	p.Ala154Val	NA	NA	chr9*125239745*125239745*G*A*3*3*Endometrial_cancer|chr9*125239745*125239745*C*A*1*1*Lung_cancer	chr9*125239745*125239745*COSM382601*G*T*1*lung*1|chr9*125239745*125239745*COSM1104831*G*A*2*central_nervous_system
chr9	1.31E+08	G	A	SPTAN1	MISSENSE	c.4411G>A	p.Glu1471Lys	NA	NA	chr9*131370475*131370475*G*A*2*2*Colorectal_cancer	chr9*131370475*131370475*COSM267497*G*A*1*large_intestine*1
chr9	1.33E+08	G	A	ASS1	MISSENSE	c.847G>A	p.Glu283Lys	NA	NA	NA	NA
chr9	1.37E+08	C	T	SARDH	MISSENSE	c.1090G>A	p.Ala364Thr	NA	NA	chr9*136582508*136582508*G*A*2*2*Colorectal_cancer|chr9*136582508*136582508*C*T*1*1*Endometrial_cancer	chr9*136582508*136582508*COSM1106507*C*T*3*large_intestine

chr9	1.39E+08	G	A	NOTCH1	MISSENSE-SS-PRX	c.3904C>T	p.Arg1302Cys	NA	NA	NA	chr9*139401089*139401094*COSM1173474*GCCCTA*G*2*oesophagus*2|chr9*139401089*139401094*COSM1173475*GCCCTA*G*2*oesophagus*2
chr9	1.4E+08	G	A	EGFL7	MISSENSE	c.172G>A	p.Gly58Arg	NA	NA	NA	NA
chr9	1.4E+08	C	G	TPRN	MISSENSE	c.1819G>C	p.Glu607Gln	NA	NA	chr9*140087050*140087050*G*C*1*1*Colorectal_cancer	chr9*140087050*140087050*COSM3699590*C*G*1*large_intestine*1|chr9*140087050*140087050*COSM3699591*C*G*1*large_intestine*1
chr9	6604643	C	CA	GLDC	FRAMESHIFT-INS	c.1002dupT	p.Ala335CysfsTer75	LIKELY_PATHOGENIC	Non-ketotic_hyperglycinemia	NA	NA
chr9	8341907	GT	G	PTPRD	FRAMESHIFT-DEL	c.4732delA	p.Thr1578LeufsTer2	NA	NA	NA	NA
chr9	32633024	CT	C	TAF1L	FRAMESHIFT-DEL	c.2553delA	p.Ala852ProfsTer14	NA	NA	chr9*32633025*32633025*A*del*1*1*Ovarian_cancer	NA
chr9	32633655	CT	C	TAF1L	FRAMESHIFT-DEL	c.1922delA	p.Lys641SerfsTer19	NA	NA	NA	NA
chr9	35091522	TG	T	PIGO	FRAMESHIFT-DEL	c.2361delC	p.Thr788LeufsTer25	PATHOGENIC	Hyperphosphatasia_with_mental_retardation_syndrome_2	chr9*35091522*35091522*ins*G*1*1*Ovarian_cancer|chr9*35091523*35091523*ins*C*1*1*Stomach_(gastric)_cancer|chr9*35091523*35091523*C*del*1*1*Colorectal_cancer	chr9*35091522*35091522*COSM112011*T*TG*1*ovary*1|chr9*35091522*35091523*COSM1462196*TG*T*1*large_intestine*1
chr9	96212875	G	GAAAAAAAAA	FAM120AOS	INFRAME-INS	c.569_570insTTTTTTTTT	p.Leu190_Cys191insPhePhePhe	NA	NA	NA	NA
chr9	96422611	CA	C	PHF2	FRAMESHIFT-DEL	c.1468delA	p.Lys492ArgfsTer6	NA	NA	chr9*96422612*96422612*A*del*5*5*Colorectal_cancer	chr9*96422611*96422612*COSM198796*CA*C*2*pancreas
chr9	1.04E+08	CAG	C	RNF20	FRAMESHIFT-DEL	c.1970_1971delAG	p.Asp659GlnfsTer8	NA	NA	NA	NA
chr9	1.34E+08	GC	G	NUP214	FRAMESHIFT-DEL	c.4351delC	p.Pro1452HisfsTer26	NA	NA	chr9*134073232*134073232*C*T*1*1*Endometrial_cancer	chr9*134073232*134073232*COSM1106085*C*T*1*endometrium*1
chr9	1.4E+08	TG	T	RABL6	FRAMESHIFT-DEL	c.1251delG	p.Ala419ProfsTer181	NA	NA	NA	NA
chr13	24797698	C	T	RP11-307N16.6	MISSENSE	c.631C>T	p.Arg211Cys	NA	NA	NA	NA
chr13	24797698	C	T	SPATA13	MISSENSE	c.631C>T	p.Arg211Cys	NA	NA	NA	NA
chr13	32863803	C	T	FRY	MISSENSE	c.8503C>T	p.His2835Tyr	NA	NA	NA	NA
chr13	32912560	G	C	BRCA2	MISSENSE	c.4068G>C	p.Leu1356Phe	OTHER|OTHER|BENIGN|BENIGN|OTHER	UNCERTAIN_SIGNIFICANCE|UNCERTAIN_SIGNIFICANCE	Hereditary_breast_and_ovarian_cancer_syndrome	Breast-ovarian_cancer_familial_2
chr13	35685017	G	A	NBEA	MISSENSE	c.1904G>A	p.Arg635His	NA	NA	NA	NA
chr13	36229015	C	T	NBEA	MISSENSE	c.7996C>T	p.Arg2666Trp	NA	NA	NA	NA
chr13	37567763	C	T	ALG5	MISSENSE	c.332G>A	p.Gly111Asp	NA	NA	NA	NA
chr13	37580099	G	A	EXOSC8	MISSENSE	c.281G>A	p.Arg94Gln	NA	NA	NA	NA

chr13	39433502	C	T	FREM2	MISSENSE	c.7294C>T	p.Arg2432Trp	NA	NA	NA	chr13*39433502*39433502*COSM352487*C*T*1*lung*1
chr13	43930175	G	A	ENOX1	MISSENSE	c.703C>T	p.Arg235Cys	NA	NA	NA	NA
chr13	88328357	T	A	SLITRK5	MISSENSE	c.714T>A	p.Asn238Lys	NA	NA	NA	NA
chr13	1.02E+08	G	A	NALCN	MISSENSE	c.3572C>T	p.Pro1191Leu	NA	NA	chr13*101736073*101736073*C*A*1*1*Lung_cancer	chr13*101736073*101736073*COSM335013*G*T*1*lung*1
chr13	1.11E+08	C	T	COL4A1	MISSENSE	c.4451G>A	p.Gly1484Asp	NA	NA	NA	NA
chr13	1.11E+08	C	A	COL4A2	MISSENSE	c.4549C>A	p.Leu1517Met	NA	NA	NA	NA
chr13	20611022	TA	T	ZMYM2	FRAMESHIFT-DEL	c.2266delA	p.Lys757AsnfsTer31	NA	NA	chr13*20611023*20611023*A*del*1*1*Colorectal_cancer	chr13*20611022*20611023*COSM1365723*TA*T*1*large_intestine*1|chr13*20611022*20611023*COSM1365724*TA*T*1*large_intestine*1|chr13*20611022*20611023*COSM1365725*TA*T*1*large_intestine*1
chr13	20717071	G	GCT	GJA3	FRAMESHIFT-INS	c.355_356dupAG	p.Ser119ArgfsTer58	NA	NA	NA	NA
chr13	28537308	CAG	C	CDX2	FRAMESHIFT-DEL	c.884_885delCT	p.Ser295CysfsTer41	NA	NA	NA	NA
chr13	35733662	TA	T	NBEA	FRAMESHIFT-DEL	c.3355delA	p.Asn1121MetfsTer9	NA	NA	chr13*35733662*35733662*ins*A*1*1*Esophageal_cancer|chr13*35733663*35733663*A*del*4*3*Stomach_(gastric)_cancer	chr13*35733662*35733663*COSM1366580*TA*T*11*pancreas
chr13	36180678	C	CA	NBEA	FRAMESHIFT-INS	c.7412_7413insA	p.Pro2474ThrfsTer2	NA	NA	NA	chr13*36180678*36180679*COSM1366607*CA*C*1*large_intestine*1
chr13	99502311	T	TA	DOCK9	FRAMESHIFT-INS	c.4002dupT	p.Thr1335TyrfsTer4	NA	NA	chr13*99502311*99502311*ins*A*1*1*Colorectal_cancer	NA
chr13	1.14E+08	C	CA	GRK1	FRAMESHIFT-INS	c.1479_1480insA	p.Gly495ArgfsTer5	NA	NA	NA	NA
chr13	1.15E+08	GC	G	GAS6	FRAMESHIFT-DEL	c.449delG	p.Gly150AlafsTer49	NA	NA	chr13*114542717*114542717*ins*C*1*1*Stomach_(gastric)_cancer	NA
chr10	16992011	G	A	CUBN	MISSENSE	c.5069C>T	p.Ala1690Val	NA	NA	chr10*16992011*16992011*C*T*1*1*Myeloma	chr10*16992011*16992011*COSM1236003*G*A*1*haematopoietic_and_lymphoid_tissue*1
chr10	26851354	C	T	APBB1IP	MISSENSE	c.1469C>T	p.Ser490Leu	NA	NA	NA	NA
chr10	27702917	G	A	PTCHD3	MISSENSE	c.263C>T	p.Pro88Leu	NA	NA	NA	chr10*27702893*27702923*COSM1474575*BigSeq*A*1*breast*1
chr10	28413986	G	A	MPP7	MISSENSE	c.482C>T	p.Ala161Val	NA	NA	NA	NA
chr10	29839650	G	A	SVIL	NONSENSE	c.703C>T	p.Arg235Ter	NA	NA	NA	NA
chr10	30336675	G	A	KIAA1462	MISSENSE	c.67C>T	p.Arg23Cys	NA	NA	NA	NA

chr10	32581942	G	A	EPC1	MISSENSE	c.640C>T	p.Arg214Cys	NA	NA	NA	NA
chr10	35351989	C	A	CUL2	MISSENSE-SS-PRX	c.178G>T	p.Asp60Tyr	NA	NA	NA	NA
chr10	68040292	A	G	CTNNA3	MISSENSE	c.1820T>C	p.Val607Ala	NA	NA	NA	NA
chr10	70451288	G	A	TET1	MISSENSE	c.6128G>A	p.Arg2043His	NA	NA	NA	NA
chr10	75277155	C	T	USP54	MISSENSE	c.3029G>A	p.Ser1010Asn	NA	NA	NA	NA
chr10	75289934	A	G	USP54	MISSENSE	c.1795T>C	p.Tyr599His	NA	NA	NA	NA
chr10	76790371	C	T	KAT6B	MISSENSE	c.5789C>T	p.Ala1930Val	NA	NA	chr10*76790371*76790371*C*T*2*2*Colorectal_cancer	chr10*76790371*76790371*COSM272992*C*T*1*large_intestine*1
chr10	79593733	G	A	DLG5	MISSENSE	c.1687C>T	p.Arg563Cys	NA	NA	NA	NA
chr10	89692905	G	A	PTEN	MISSENSE	c.389G>A	p.Arg130Gln	PATHOGENIC:PATHOGENIC|PATHOGENIC|PATHOGENIC|PATHOGENIC	LIKELY_PATHOGENIC	Cowden_syndrome_1:Cowden_syndrome_1	Hereditary_cancer-predisposing_syndrome
chr10	91198854	C	T	SLC16A12	MISSENSE	c.535G>A	p.Ala179Thr	NA	NA	NA	NA
chr10	1.03E+08	G	A	PDZD7	MISSENSE	c.493C>T	p.Arg165Cys	NA	NA	NA	NA
chr10	1.04E+08	G	T	HPS6	MISSENSE	c.1947G>T	p.Trp649Cys	NA	NA	NA	NA
chr10	1.15E+08	A	T	TCF7L2	NONSENSE	c.1402A>T	p.Lys468Ter	NA	NA	chr10*114925317*114925325*ins*AAAAAAAAA*3*2*Colon_cancer|chr10*114925317*114925325*AAAAAAAAA*del*8*2*Rectal_cance	NA
chr10	1.16E+08	T	C	ABLIM1	MISSENSE	c.26A>G	p.Glu9Gly	NA	NA	NA	NA
chr10	1.16E+08	T	C	ABLIM1	MISSENSE	c.26A>G	p.Glu9Gly	NA	NA	NA	NA
chr10	1.16E+08	T	C	ABLIM1	MISSENSE	c.26A>G	p.Glu9Gly	NA	NA	NA	NA
chr10	1.16E+08	T	C	ABLIM1	MISSENSE	c.26A>G	p.Glu9Gly	NA	NA	NA	NA
chr10	55780107	CA	C	PCDH15	FRAMESHIFT-DEL	c.2595delT	p.Phe865LeufsTer13	NA	NA	NA	NA
chr10	60558976	C	CA	BICC1	FRAMESHIFT-INS	c.1689_1690insA	p.Ile566AsnfsTer44	NA	NA	NA	chr10*60558976*60558977*COSM1348387*CA*C*1*large_intestine*1
chr10	61831289	A	AT	ANK3	FRAMESHIFT-INS	c.9349dupA	p.Ile3117AsnfsTer7	NA	NA	NA	chr10*61831289*61831289*COSM1180736*A*AT*1*large_intestine*1|chr10*61831289*61831290*COSM1348429*AT*A*2*large_intestine*2
chr10	64952698	CT	C	JMJD1C	FRAMESHIFT-DEL-SS-PRX	c.6075delA	p.Glu2026LysfsTer45	NA	NA	chr10*64952699*64952699*T*del*1*1*Stomach_(gastric)_cancer|chr10*64952699*64952699*A*del*1*1*Stomach_(gastric)_cancer	chr10*64952698*64952699*COSM1348576*CT*C*3*large_intestine
chr10	69676232	C	CAG	SIRT1	FRAMESHIFT-INS	c.2126_2127insAG	p.Ala712GlufsTer23	NA	NA	NA	NA
chr10	79581395	C	CT	DLG5	FRAMESHIFT-INS	c.2846dupA	p.Ala950GlyfsTer10	NA	NA	NA	NA
chr10	88415981	AC	A	OPN4	FRAMESHIFT-DEL	c.215delC	p.Leu73TrpfsTer4	NA	NA	NA	NA

chr10	98336474	CT	C	TM9SF3	FRAMESHIFT-DEL	c.214delA	p.Ser72ValfsTer31	NA	NA	chr10*98336475*98336475*T*del*3*3*Stomach_(gastric)_cancer|chr10*98336475*98336475*A*del*1*1*Lung_cancer	chr10*98336474*98336475*COSM392624*CT*C*6*NS
chr10	1E+08	TG	T	HPS1	FRAMESHIFT-DEL	c.972delC	p.Met325TrpfsTer6	PATHOGENIC:PATHOGENIC	Hermansky-Pudlak_syndrome_1:Hermansky-Pudlak_syndrome_1	chr10*100186986*100186986*ins*G*2*1*Colorectal_cancer|chr10*100186987*100186987*G*del*1*1*Stomach_(gastric)_cancer	chr10*100186986*100186986*COSM298286*T*TG*2*large_intestine*2|chr10*100186986*100186986*COSM3685965*T*TG*1*large_intestine*1|chr10*100186986*100186987*COSM1345187*TG*T*4*large_intestine*4|chr10*100186986*100186988*COSM1345188*TGG*T*1*large_intestine*1|chr10*100186987*100186987*COSM3685966*G*GG*1*large_intestine*1|chr10*100186987*100186987*COSM3685967*G*GG*1*large
chr10	1.17E+08	AT	A	ATRNL1	FRAMESHIFT-DEL	c.570delT	p.Phe192LeufsTer8	NA	NA	chr10*116887435*116887435*T*del*1*1*Colorectal_cancer	chr10*116887434*116887435*COSM1345965*AT*A*1*large_intestine*1
chr10	1.3E+08	AT	A	MKI67	FRAMESHIFT-DEL	c.5569delA	p.Ile1857TyrfsTer30	NA	NA	chr10*129904535*129904535*A*del*1*1*Colorectal_cancer	chr10*129904534*129904535*COSM1346772*AT*A*1*large_intestine*1
chr10	1.35E+08	TA	T	CYP2E1	FRAMESHIFT-DEL	c.708delA	p.Asn238MetfsTer5	NA	NA	NA	NA
chr5	1294138	G	A	TERT	MISSENSE	c.863C>T	p.Ala288Val	NA	NA	NA	NA
chr5	5306675	A	T	ADAMTS16	MISSENSE	c.3245A>T	p.Tyr1082Phe	NA	NA	NA	NA
chr5	13714660	T	A	DNAH5	NONSENSE	c.12979A>T	p.Lys4327Ter	NA	NA	NA	NA
chr5	13751362	A	G	DNAH5	MISSENSE	c.11036T>C	p.Val3679Ala	NA	NA	NA	NA
chr5	14359492	C	T	TRIO	MISSENSE	c.2243C>T	p.Thr748Ile	NA	NA	NA	NA
chr5	22078649	C	T	CDH12	MISSENSE	c.137G>A	p.Arg46Gln	NA	NA	chr5*22078649*22078649*C*A*1*1*Lung_cancer	NA
chr5	32101307	C	T	PDZD2	MISSENSE	c.8315C>T	p.Thr2772Met	NA	NA	NA	NA
chr5	36171770	C	T	SKP2	MISSENSE	c.836C>T	p.Ala279Val	NA	NA	NA	NA
chr5	37834919	C	T	GDNF	MISSENSE	c.31G>A	p.Ala11Thr	NA	NA	chr5*37834919*37834919*G*A*1*1*Colorectal_cancer	chr5*37834919*37834919*COSM1437402*C*T*1*large_intestine*1
chr5	42719162	C	T	GHR	MISSENSE	c.1553C>T	p.Pro518Leu	NA	NA	chr5*42719162*42719162*C*T*1*1*Lung_cancer	chr5*42719162*42719162*COSM311440*C*T*1*lung*1

chr5	63257353	C	T	HTR1A	MISSENSE	c.194G>A	p.Arg65His	NA	NA	chr5*63257353*63257353*G*A*1*1*Colorectal_cancer	chr5*63257353*63257353*COSM1438123*C*T*1*large_intestine*1
chr5	64569234	C	T	ADAMTS6	MISSENSE	c.1553G>A	p.Arg518His	NA	NA	chr5*64569234*64569234*C*T*2*2*Endometrial_cancer	chr5*64569234*64569234*COSM1069333*C*T*2*oesophagus
chr5	66459128	T	C	MAST4	MISSENSE	c.4121T>C	p.Ile1374Thr	NA	NA	NA	NA
chr5	73218966	C	T	ARHGEF28	MISSENSE	c.5003C>T	p.Thr1668Ile	NA	NA	NA	NA
chr5	77311318	T	A	AP3B1	MISSENSE	c.3047A>T	p.Asn1016Ile	NA	NA	NA	NA
chr5	90052365	G	T	GPR98	MISSENSE	c.11675G>T	p.Arg3892Leu	NA	NA	NA	NA
chr5	90077306	G	A	GPR98	MISSENSE	c.13142G>A	p.Gly4381Glu	NA	NA	NA	NA
chr5	92923764	G	A	NR2F1	MISSENSE	c.605G>A	p.Arg202His	NA	NA	NA	NA
chr5	96086336	C	T	CAST	MISSENSE	c.1351C>T	p.Arg451Trp	NA	NA	NA	NA
chr5	1.12E+08	G	T	APC	MISSENSE-SS-PRX	c.422G>T	p.Arg141Met	NA	NA	chr5*112103086*112103087*AG*del*2*1*Colorectal_cancer	chr5*112103085*112103087*COSM25153*GAG*G*2*large_intestine*2
chr5	1.12E+08	C	T	APC	NONSENSE	c.2626C>T	p.Arg876Ter	PATHOGENIC|PATHOGENIC	Familial_adenomatous_polyposis_1	not_provided	chr5*112173912*112173917*CAAAGC*del*1*1*Colorectal_cancer|chr5*112173917*112173917*C*T*82*24*Duodenal_cancer
chr5	1.22E+08	C	T	SNCAIP	MISSENSE	c.179C>T	p.Thr60Met	NA	NA	chr5*121758611*121758611*C*T*1*1*Esophageal_cancer	chr5*121758611*121758611*COSM1266333*C*T*1*oesophagus*1|chr5*121758611*121758611*COSM1266332*C*T*1*oesophagus*1|chr5*121758611*121758611*COSM1266331*C*T*1*oesophagus*1
chr5	1.27E+08	G	A	MEGF10	MISSENSE	c.212G>A	p.Arg71Gln	NA	NA	NA	NA
chr5	1.31E+08	C	T	FNIP1	MISSENSE	c.2161G>A	p.Ala721Thr	NA	NA	NA	NA
chr5	1.41E+08	G	A	HDAC3	MISSENSE	c.487C>T	p.Arg163Trp	NA	NA	NA	NA
chr5	1.51E+08	G	A	SLC36A2	MISSENSE	c.293C>T	p.Ala98Val	NA	NA	NA	NA
chr5	1.53E+08	C	T	GRIA1	MISSENSE	c.2276C>T	p.Ala759Val	NA	NA	NA	NA
chr5	1.67E+08	C	T	TENM2	MISSENSE	c.854C>T	p.Pro285Leu	NA	NA	NA	NA
chr5	1.75E+08	T	C	HRH2	MISSENSE	c.533T>C	p.Val178Ala	NA	NA	NA	NA
chr5	1.77E+08	C	T	FGFR4	MISSENSE	c.1693C>T	p.Arg565Trp	NA	NA	NA	NA
chr5	1.79E+08	C	T	SQSTM1	MISSENSE	c.257C>T	p.Ala86Val	NA	NA	NA	NA
chr5	14477003	CT	C	TRIO	FRAMESHIFT-DEL-SS-PRX	c.6085delT	p.Leu2031Ter	NA	NA	chr5*14477004*14477004*T*del*1*1*Esophageal_cancer	chr5*14477003*14477004*COSM1241852*CT*C*3*oesophagus
chr5	35867540	CA	C	IL7R	FRAMESHIFT-DEL	c.355delA	p.Ile121Ter	PATHOGENIC	Severe_combined_immunodeficiency_autosomal_recessive_T_cell-negative_B_cell-positive_NK_cell-positive	NA	NA
chr5	68565038	C	CTTTTTTTTT	CDK7	INFRAME-INS	c.632_633insTTTTTTTTT	p.Phe212_Leu213insPhePhePhe	NA	NA	NA	NA
chr5	68736315	GA	G	MARVELD2	FRAMESHIFT-DEL	c.1543delA	p.Lys517ArgfsTer16	NA	NA	NA	NA

chr5	73154018	T	TG	ARHGEF28	FRAMESHIFT-INS	c.2021_2022insG	p.Lys676GlufsTer8	NA	NA	NA	NA
chr5	76358966	CA	C	AGGF1	FRAMESHIFT-DEL	c.2035delA	p.Asn681ThrfsTer29	NA	NA	chr5*76358966*76358967*ins*A*1*1*Colorectal_cancer|chr5*76358967*76358967*A*del*1*1*Stomach_(gastric)_cancer	chr5*76358966*76358966*COSM1438673*C*CA*1*large_intestine*1|chr5*76358966*76358967*COSM1438674*CA*C*4*large_intestine*4
chr5	79970914	CA	C	MSH3	FRAMESHIFT-DEL	c.1141delA	p.Lys383ArgfsTer32	PATHOGENIC	Endometrial_carcinoma	chr5*79970915*79970915*ins*A*1*1*Pancreatic_cancer|chr5*79970915*79970915*A*del*8*3*Stomach_(gastric)_cancer|chr5*79970915*79970916*AA*del*1*1*Colorectal_cancer|chr5*79970915*79970922*AAAAAAAA*del*11*1*Colon_cancer	chr5*79970914*79970915*COSM1642798*CA*C*3*NS
chr5	1.09E+08	CA	C	MAN2A1	FRAMESHIFT-DEL	c.1910delA	p.Asn639IlefsTer2	NA	NA	NA	NA
chr5	1.12E+08	AAG	A	APC	FRAMESHIFT-DEL	c.4385_4386delAG	p.Ser1465TrpfsTer3	UNCERTAIN_SIGNIFICANCE:PATHOGENIC|PATHOGENIC|PATHOGENIC|PATHOGENIC:PATHOGENIC	not_provided:Gardner_syndrome	Periampullary_adenoma	not_provided
chr5	1.19E+08	TGGACACAA	T	DMXL1	FRAMESHIFT-DEL	c.5005_5012delGGACACAA	p.Gly1669PhefsTer2	NA	NA	NA	NA
chr5	1.32E+08	CT	C	SLC22A4	FRAMESHIFT-DEL	c.1514delT	p.Phe507SerfsTer7	NA	NA	chr5*131676327*131676327*ins*T*1*1*Ovarian_carcinoma|chr5*131676327*131676327*T*del*2*2*Stomach_(gastric)_can	chr5*131676326*131676327*COSM1433027*CT*C*2*NS

Suppl. Table 10: OncopeptVAC prediction of peptides derived from somatic mutations found in Family 2, patient II.2, LS+ MLH1mut

Mutation		#Unique Peptides	HLA-A*01:01	HLA-A*02:06	HLA-B*15:02	HLA-B*35:03	HLA-C*04:01	HLA-C*08:01
Missense	Total number of unique peptides	6104						
	Total number of mutant peptides <1000 nM	548 (9%)	42	406	116	18	1	25
	Total number of immunogenic mutant peptide <1000 nM binding affinity	289 (53%)	21	214	62	13	1	8
	Total number of immunogenic peptides <1000nM expressed at the RNA level (>10 Alt reads)	74 (26%)	4	56	18	1	0	1
Indel	Total number of unique peptides	6379						
	Total number of peptides <1000 nM binding	369 (6%)	8	307	54	18	1	15
	Total number of immunogenic peptides <1000 nM binding	162 (44%)	3	134	23	15	0	4
	Total number of immunogenic peptides <1000 nM expressed at the RNA level (>10 alt read)	29 (18%)	0	23	3	4	0	1

Suppl. Table 11: Pathogenic somatic mutations found in the tumor sample of an LS+MLH1mut patient (Family 2; II.2)

GENE_NAME	VARCLASS	CDNA_CHG	AA_CHG	ClinVar_Significance	ClinVar_Disease
SGCE	NONSENSE	c.289C>T	p.Arg97Ter	PATHOGENIC	Myoclonic_dystonia
GNAS	MISSENSE	c.2530C>T	p.Arg844Cys	PATHOGENIC	Pancreatic cancer and other cancer
RIN2	FRAMESHIFT-INS	c.1871_1872insC	p.Ile627HisfsTer7	PATHOGENIC	Macrocephaly_alopecia_cutis_laxa_and_scoliosis
SMARCB1	FRAMESHIFT-DEL	c.208delA	p.Thr72GlnfsTer13	PATHOGENIC	SCHWANNOMATOSIS_1
BAX	FRAMESHIFT-DEL	c.114delG	p.Glu41ArgfsTer19	PATHOGENIC	Carcinoma_of_colon:Carcinoma_of_colon:T-cell_acute_lymphoblastic_leukemia
CABP4	MISSENSE	c.370C>T	p.Arg124Cys	PATHOGENIC	Congenital_stationary_night_blindness_type_2B
PAFAH1B1	MISSENSE	c.722G>A	p.Arg241Gln	PATHOGENIC	Subcortical_band_heterotopia
MYO15A	FRAMESHIFT-DEL	c.1179delC	p.Glu396ArgfsTer48	PATHOGENIC	Non-syndromic_genetic_deafness
DGKE	FRAMESHIFT-DEL	c.604delA	p.Thr204GlnfsTer6	PATHOGENIC	Nephrotic_syndrome_type_7
AXIN2	FRAMESHIFT-DEL	c.1994delG	p.Gly665AlafsTer24	PATHOGENIC	Carcinoma_of_colon
TBX6	FRAMESHIFT-DEL	c.704delG	p.Gly235AlafsTer15	PATHOGENIC	Spondylocostal_dysostosis_5
PCDH19	FRAMESHIFT-DEL	c.1091delC	p.Pro364ArgfsTer4	PATHOGENIC	Early_infantile_epileptic_encephalopathy_9
LRPPRC	FRAMESHIFT-DEL-SS-PRX	c.3146_3147delAA	p.Lys1049ArgfsTer4	PATHOGENIC	Leigh_syndrome_French_Canadian_type
MSH6	FRAMESHIFT-INS	c.3253_3254insC	p.Phe1088LeufsTer5	PATHOGENIC	Lynch_syndrome
PIGO	FRAMESHIFT-DEL	c.2361delC	p.Thr788LeufsTer25	PATHOGENIC	Hyperphosphatasia_with_mental_retardation_syndrome_2
PTEN	MISSENSE	c.389G>A	p.Arg130Gln	PATHOGENIC	Endometrium, colorectal and other cancer
HPS1	FRAMESHIFT-DEL	c.972delC	p.Met325TrpfsTer6	PATHOGENIC	Hermansky-Pudlak_syndrome_1:Hermansky-Pudlak_syndrome_1
APC	NONSENSE	c.2626C>T	p.Arg876Ter	PATHOGENIC	Familial_adenomatous_polyposis_1
IL7R	FRAMESHIFT-DEL	c.355delA	p.Ile121Ter	PATHOGENIC	Severe_combined_immunodeficiency_autosomal_recessive_T_cell-negative_B_cell-positive_NK_cell-positive
MSH3	FRAMESHIFT-DEL	c.1141delA	p.Lys383ArgfsTer32	PATHOGENIC	Endometrial_carcinoma

Suppl Fig 1

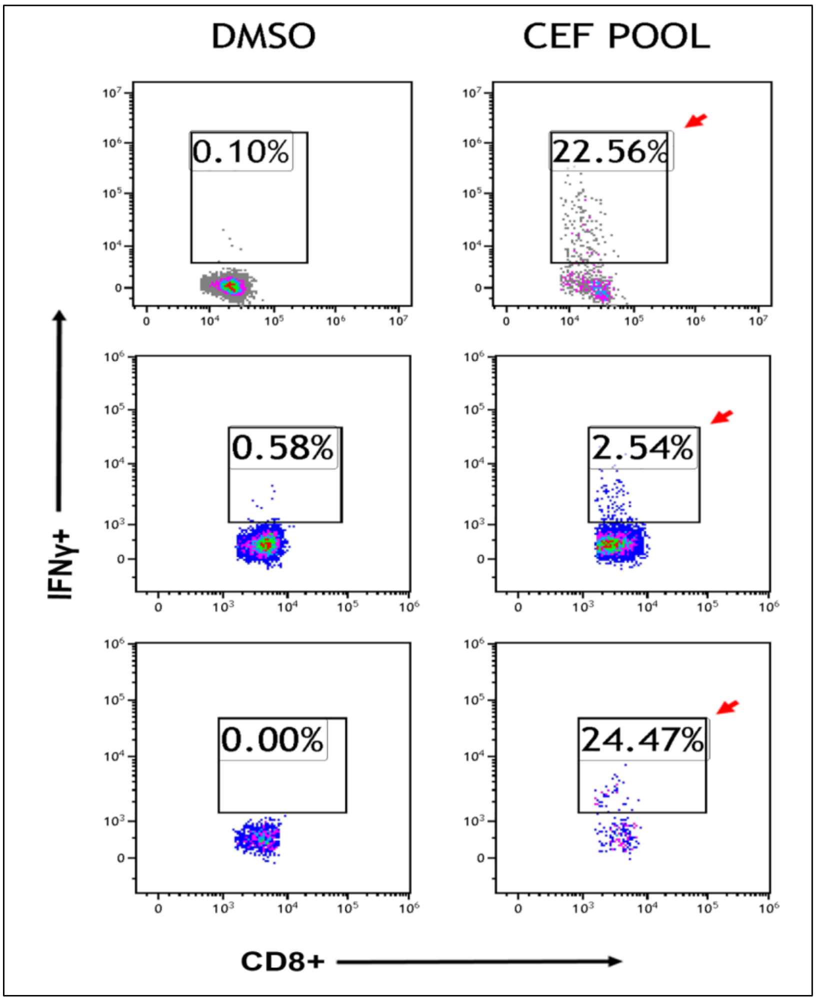

Suppl Fig 2

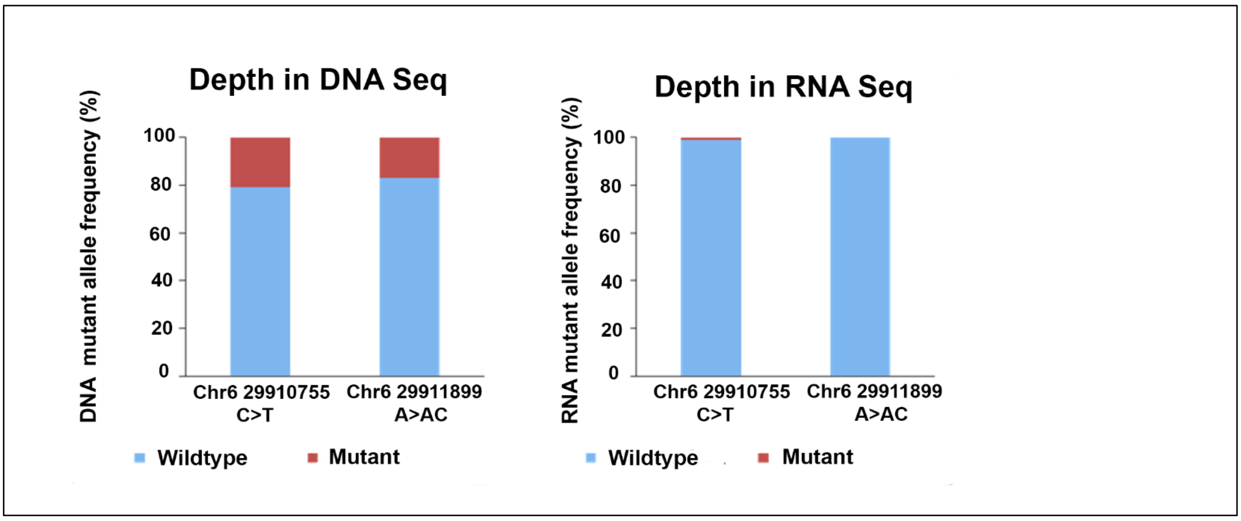

Suppl Fig 3

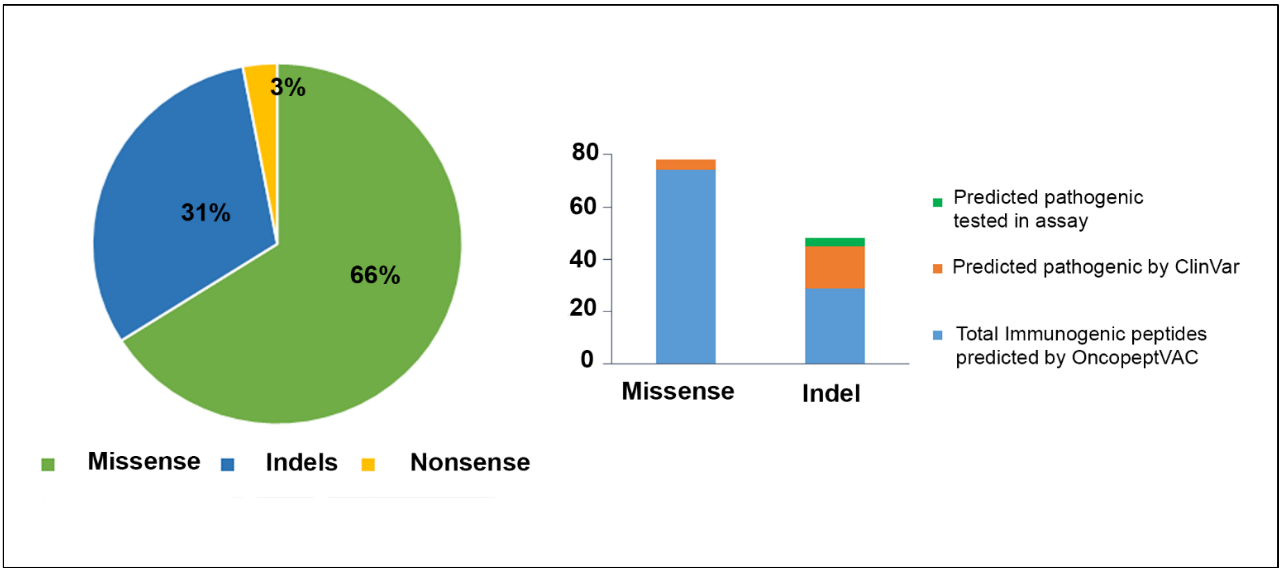

Suppl Fig 4

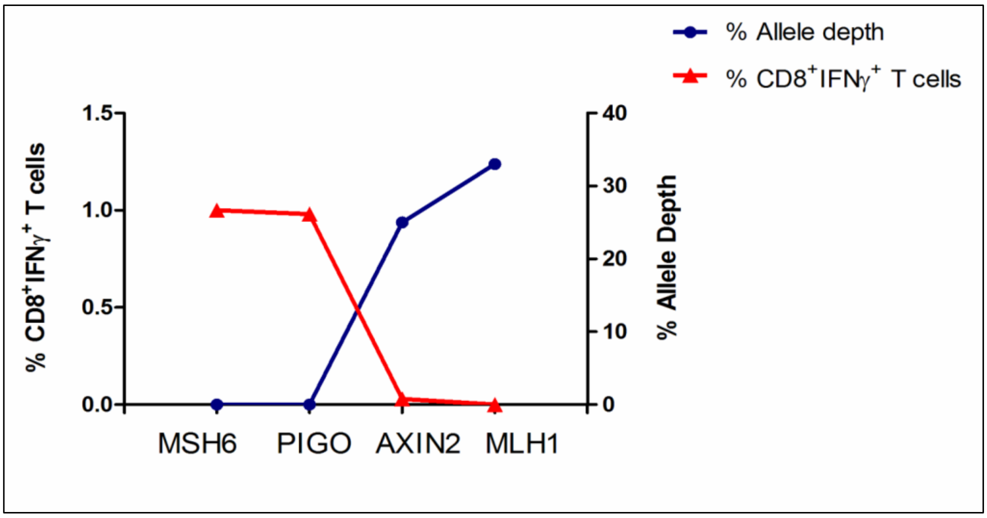

SUPPLEMENTARY TABLES:

Suppl. Table 1. Clinical features of Family 1.

Suppl. Table 2. Clinical features of Family 2.

Suppl. Table 3. Germline mutations found in MMR genes in the blood samples collected from two affected and two unaffected members of Family 2.

Suppl. Table 4. A. HLA typing of members of Family 1 and 2. HLA typing was performed by NGS methods in four members from each of the two LS families (Fig 1A and 1B). The HLAs highlighted in yellow were predicted to have a strong binding affinity to the germline *MLH1* mutation derived peptide (TSIQVIVKR) (See text). **B.** Top panel: Protein sequence of wildtype and mutant *MLH1* gene with predicted neoepitopes. Bottom panel: Only one mutant peptide (TSIQVIVKR) was predicted to have a strong HLA binding affinity but was deprioritized based on the TCR binding criteria (see methods). None of the *MLH1* germline mutation derived peptides were predicted to have strong binding affinity with HLAs found in members of family 2.

Suppl. Table 5: Analysis of peptides derived from the germline mutation in the MLH1 gene (p.E53RfsX4)

Suppl. Table 6. MLH1 p.E53RfsX4 derived peptide binding prediction with HLA types of Family 2 members.

Suppl. Table 7. HLA types of healthy donors tested.

Suppl. Table 8. List of genes in the 20MB panel used to screen LS affected individuals by NGS.

Suppl. Table 9. List of somatic mutations found in Family 2, patient II.2, LS^+ $MLH1^{mut}$

Suppl. Table 10. Oncopept*VAC* prediction of peptides derived from somatic mutations found in Family 2, patient II.2, LS^+ $MLH1^{mut}$

Suppl. Table 11. Pathogenic somatic mutations found in the tumour sample of an LS+MLH1mut patient (Family 2; II.2) Peptides derived from genes highlighted in green were tested in CD8+ T cell activation assay (see text).

SUPPLEMENTARY FIGURES:

Suppl. Fig. 1. CEF peptide pool (PM-CEF-E, JPT Peptide Technologies) used as a positive control in CD8+ T cell activation assay in healthy donor 1 (top panel), healthy donor 2 (middle panel) and Lynch syndrome patient (Family 2; II.2, $LS^{+}MLH1^{mut}$) (bottom panel).

Suppl. Fig. 2. DNA and RNA depth of HLA-A variants found in Family 2, patient II.2, LS^{+} $MLH1^{mut}$

Suppl. Fig. 3. A. Pie chart showing the breakup of different types of mutations observed in the tumour of the LS-CRC (II.2) patient. **B.** Predicted pathogenic variants determined by OncoPept*VAC* and COSMIC. Empirically tested peptides are marked in green.

Suppl. Fig. 4. Inverse relationship between the percent allele depth and the percent $CD8^{+}IFN\gamma^{+}$ T cells for *MSH6, PIGO, AXIN2* and *MLH1* genes.
